# A Network Pharmacology Study: Reveal the Mechanisms of Palovarotene Against Heterotopic Ossification

**DOI:** 10.3389/fmed.2022.897392

**Published:** 2022-05-13

**Authors:** Junchao Huang, Dachuan Liu, Jingwei Zhang, Haijun Xiao

**Affiliations:** ^1^Department of Orthopedics, Shanghai Fenxian District Central Hospital/Anhui University of Science and Technology Affiliated Fengxian Hospital, Shanghai, China; ^2^Department of Orthopaedic Surgery, Orthopaedic Institute, The First Affiliated Hospital of Soochow University, Suzhou, China

**Keywords:** Palovarotene, heterotopic ossification, network pharmacology, module-based network analysis, topological analysis, Gene-Phenotype Correlation Analysis

## Abstract

Heterotopic ossification (HO) occurs when bone forms within non-ossifying tissues, such as in muscle. Palovarotene, an activator of retinoic acid receptor γ (RAR-γ), has been shown to inhibit the formation of ectopic bone in HO model mice, but its specific mechanism of action remains unclear. This study will explore the target and molecular mechanism of Palovarotene's action on HO by network pharmacology study. We collected the relevant targets of Palovarotene and HO from the database, obtained the potential targets of Palovarotene acting on HO through Venn analysis, and constructed the protein-protein interaction (PPI) network. Then, Gene Ontology (GO) and KEGG (Kyoto Encyclopedia of Genes and Genomes) enrichment Analysis and Module-based Network Analysis were performed for potential targets, and in addition, PPI Network Topology Analysis and Gene-Phenotype Correlation Analysis were performed. The results suggested that MAPK1, MDM2, and other targets as well as P53 signaling pathway and PI3K–Akt signaling pathway may be closely related to Palovarotene treatment of HO. We carried out verification experiments to confirm our finding, alkaline phosphatase and alizarin red staining *in vitro* and Micro-CT as well as hematoxylin-eosin staining *in vivo* were performed to verify treatment for HO of Palovarotene, reverse transcription polymerase chain reaction was also used to explore the transcription changes of MAPK1, MDM2, and osteogenic genes. This study systematically elucidated the possible mechanism of Palovarotene in the treatment of HO through network pharmacology study, revealing a new direction for the further application of Palovarotene in the treatment of HO.

## Introduction

Heterotopic ossification (HO) refers to bone formation in tissues that do not have ossification properties under normal conditions (1). Heterotopic ossification can be either congenital or acquired. The latter is usually related to trauma (2). Patients with early heterotopic ossification have obvious local swelling and pain, the range of motion of the joints gradually narrows, and movement is restricted; in the late stage, due to the loss of more soft tissue and the formation of bone tissue, the joints may even lose the ability to move (3). Because heterotopic ossification brings serious consequences to patients. Therefore, the search for drugs to prevent and treat heterotopic ossification has become a contemporary research hotspot.

Palovarotene is a retinoic acid receptor-gamma (RAR-γ) agonist that inactivates activin receptor-like kinase 2 (Alk2) receptors. This receptor usually interacts with bone morphogenetic protein (BMP), which can inhibit the formation of new bone tissue outside the bone (4). According to research, the combined use of corticosteroids and retinoic acid receptor gamma agonist Palovarotene can significantly inhibit heterotopic ossification (5). It has broad prospects in the treatment of heterotopic ossification.

As the requirements for drug research and development continue to increase, innovative drug research and development are facing huge difficulties. The development of highly selective single-target drugs has shown obvious limitations. In order to change this deep-rooted concept of “one gene, one drug, one disease,” network pharmacology was proposed by Hopkins (6, 7). Since the human body is a dynamic and interactive complex environment, if any organ's homeostasis is disrupted, it will cause the change in the body environment, and this dynamic network will be destroyed (8). Network pharmacology emphasizes the analysis of the molecular association rules between drugs and treatment targets from the perspective of biological networks, providing new scientific and technological support for clinical rational drug use and new drug research and development.

**Figure 1:**
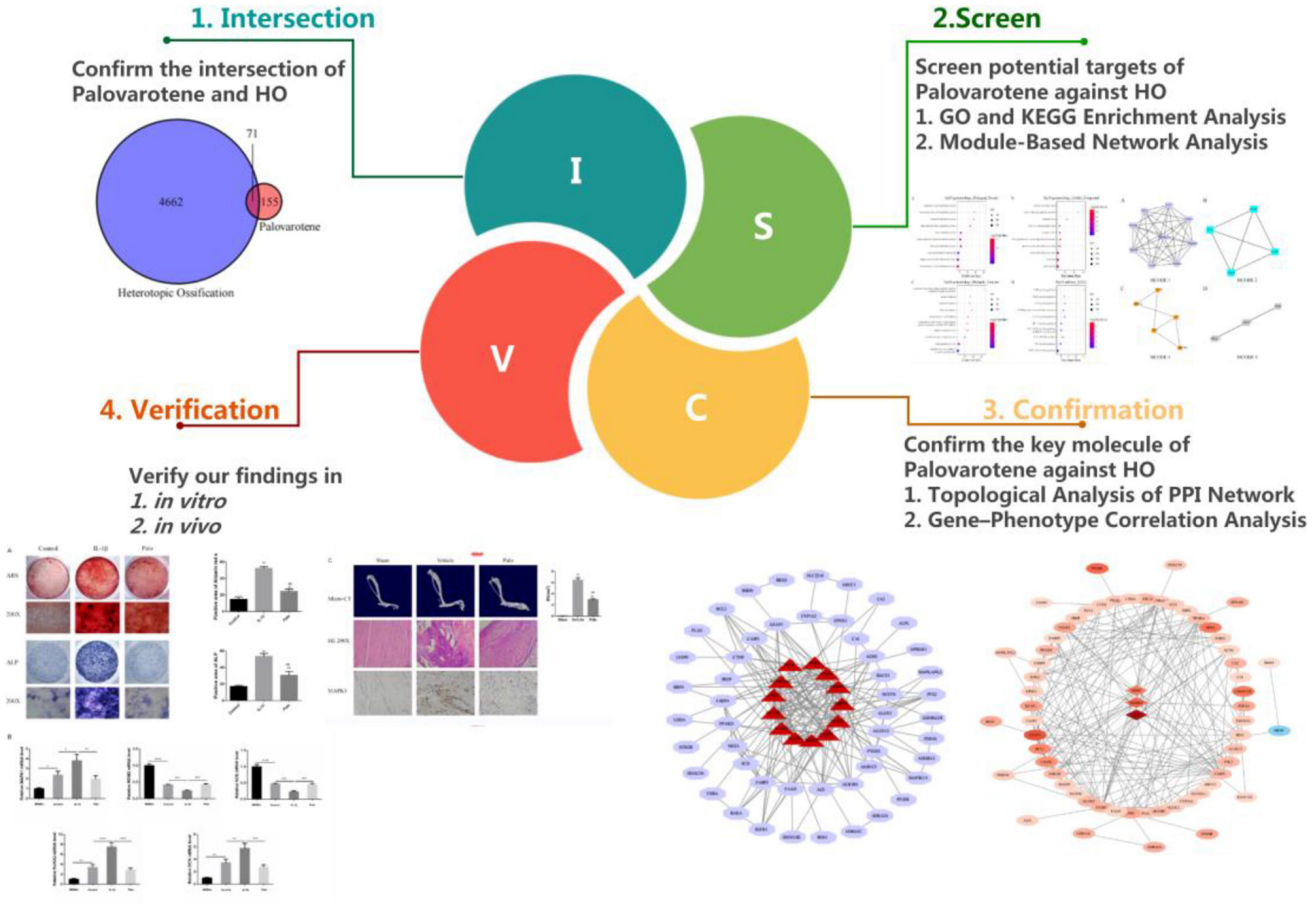
Workflow diagram of method.

Currently, various studies have been conducted on the mechanism of Palovarotene in treating HO, but these views are one-sided and static. The mechanism of Palovarotene's action on HO should be multi-signal pathway and multi-target. Therefore, in order to explore as much and comprehensive mechanisms by which Palovarotene acts on HO as possible, we used the method of network pharmacology. In addition, we also carry out conceptual verification by a series of experiments (Figure 1).

## Methods

### Software and Database

The database and related analysis platform used in this study are shown in Table 1.

**Table 1:** The database and related analysis platform used in this study.

**Names**	**URL**	**Purpose**
PubChem	https://pubchem.ncbi.nlm.nih.gov	Get the Palovarotene structure
ChEMBL	http://www.ebi.ac.uk/chembl/	Obtain the Palovarotene target
SwissTarget Prediction	http://www.swisstargetprediction.ch	Obtain the Palovarotene target
UniProt	http://www.uniprot.org/	Target name standardization
DisGeNET	http://www.disgenet.org/	Obtain related genes of HO
Comparative Toxicogenomics Database	http://ctdbase.org/	Obtain related genes of HO
STRING	https://www.string-db.org/	Construction of PPI network
Metascape	www.metascape.org/	Perform module-based network analysis
VarElect	http://ve.genecards.org	Analyze the relationship between hub genes and disease

### Target Gene Screening of Palovarotene

PubChem can provide structural information and bioactivity information about chemical substances. We collected potential protein targets of Palovarotene through PubChem. Using Palovarotene as the search term, the 3D molecular structure of Palovarotene can be obtained through the PubChem database. The obtained information was input into ChEMBL and SWisStar prediction database to predict palovarotene-related targets. Finally, the English names of the screened target proteins were input into Uniprot database for standardization (Supplementary Table 1).

### Acquisition of Palovarotene and HO Intersection Targets

Search DisGeNET Database and Comparative Genomics Database by search terms for heterotopic ossification (9). The disease target of HO was obtained by integration after elimination of repetitions, detailed information of genes was shown in Supplementary Table 2. Then we performed Venny analysis on HO disease targets and Palovarotene-related targets. Intersection targets of Palovarotene and HO in Venn diagram were regarded as potential targets.

### PPI Network Construction

In order to clarify the interaction between intersection targets, drug-disease intersection target data was imported into STRING database (10). Homo sapiens was set as species, a PPI (protein-protein interaction) network of intersection targets was obtained. The network analysis function of Cytoscape 3.7.2 was utilized to analyze the obtained protein interaction information. It uses a confidence range to define PPI (Low confidence: Score < 0.4; Moderate: 0.4–0.7; Height: > 0.7). Take into account these scores, this study retained a PPI with a comprehensive score > 0.4. Visualize the PPI network by using Cytoscape 3.7.2.

### GO and KEGG Enrichment Analysis

R software package WebGestaltR (V0.4.4) was used for KEGG pathway analysis and GO functional enrichment analysis of differential genes (11), *P* < 0.05. The biological process (BP), cell component (MF), molecular function (MF), and pathways were screened in descending order according to the enrichment degree of the target. Finally, the data was visualized.

### Module-Based Network Analysis

Metascape was used for modular network analysis (12). Mature MCODE algorithm was used to find a few closely connected protein groups in a large and complex target network, and the biological functions of each protein group were also labeled.

### Topological Analysis of PPI Network

We used the Network Analyzer function in CytoScape3.7.2 to conduct topological analysis on PPI Network (13), its Node degree distribution, Betweenness centrality and Closeness centrality were calculated, the node with the highest ranking among the three parameters can be identified as the core target.

### Gene–Phenotype Correlation Analysis

To further defined the relationship between genes and phenotypes, we used the VarElec tool (10). VarElect's algorithm can verify the direct or indirect relationship between genes and phenotypes, and it can select the genes with the highest correlation with phenotypes from a group of genes. We were input genes screened from Palovarotene's potential therapeutic targets against HO and phenotype of HO into VarElect to obtain the results.

### Preparation of Bone Mesenchymal Stem Cells

Bone marrow mesenchymal stromal cells (BMSCs) were selected as our validation cells in subsequent experiments. BMSCs have been found to have osteogenic potential, which is one of HO progenitor cells (14), BMSCs (Procell, #CP-M131) were purchased from Wuhan Procell. The cells were cultured in an incubator at 37°C and 5% CO_2_. BMSCs were cultured in osteogenic induction medium for 14 to 21 days to induce osteogenic differentiation. The osteogenic induction medium used in this study consisted of 10 nM Dexamethasone, 50 μg/mL ascorbic acid, 10 mM B-GP disodium, 10% fetal bovine serum, and high-glucose Dulbecco's Modified Eagle Medium DMEM (Gibco, MA, USA). We used IL-1β to mimic inflammatory stimuli. The dosage of IL-1 β is 10 ng/mL *in vitro*. And the dosage of Palovarotene is 0.25 μM *in vitro*. These above concentrations were supported by our preliminary experimental verification. In *in vitro* experiments, we are grouped into: Control group (Control), IL-1β group (IL-1β), IL-1β group plus Palovarotene group (Palo).

### Alizarin Red and Alkaline Phosphatase Staining

After 14 days of osteogenesis, alkaline phosphatase (ALP) staining was performed; 40 uL of reagent A was added to 1 mL of reaction buffer, and 40 uL of reagent B was added to the mix, that is, the reaction working solution (ready for use) was prepared (the reagents were purchased from Beyotime, #C3206). Subsequently, 1 mL of PBS was added to each well and was removed after 1 min, and the cells were washed twice. Subsequently, 500 μL of fixing solution was added to each well, and the cells were fixed at 37°C (or room temperature) for 30 min. Add the prepared reaction solution into each well, and the cells were dyed at 37°C (or room temperature) for 30 min. Lastly, 1 mL of washing solution was added to each well; the cells were washed twice, The cells were washed twice and examined under a microscope.

Then, after 21 days of osteogenesis, alizarin red staining (ARS) was performed as follows: 1 mL of PBS was added to each well and was removed after 1 min. Furthermore, 1 mL of 70% ethanol or 10% neutral formaldehyde was added to each well, and the cells were fixed at 37°C (or room temperature) for 30 min. After discarding the fixed solution in the well, 1 mL washing solution was added to each well. The cells were washed thrice, and 3 mL of alizarin red S staining solution (purchased from Sigma, #A5533) was added to each well; the stained cells were incubated for 15–20 min at 37°C. The cells were washed twice with the washing solution and were observed under a microscope.

### RT-PCR

After 14 days of osteogenesis, the transcription level of osteogenic genes was detected by reverse transcription PCR (RT-PCR). We added an additional group of BMSCs that were cultured without osteogenic induction medium but with normal medium. The reaction system used was SYBR Green Mix (Takara, RR420A), and the fluorescence signal was obtained by a detecting instrument (Roche, Light Cycler 480). The primer sequences used in this study shown in Table 2.

**Table 2:** Primer sequences.

**Gene**	**Primers**
OCN-mouse-F	CTGACCTCACAGATCCCAAGC
OCN-mouse-R	TGGTCTGATAGCTCGTCACAAG
Runx2-mouse-F	CCAACTTCCTGTGCTCCGTG
Runx2-mouse-R	TCTTGCCTCGTCCGCTCC
MAPK1-mouse-F	ATGGTTTGCTCTGCTTAT
MAPK1-mouse-R	TGATGCCAATGATGTTCT
MDM2-mouse-F	GGTCTATCGGGTCACAGT
MDM2-mouse-R	TTATCTTTCCCCTTATCGT
ACE-mouse-F	CGTTACCCGACAACTATC
ACE-mouse-R	CGTTACCCGACAACTATC
Actin-mouse-F	GTCCCTCACCCTCCCAAAAG
Actin-mouse-R	GCTGCCTCAACACCTCAACCC

### Construction of HO Animal Model

The experimental rats were 24 males SD rats (4 weeks, weight 200 ± 5 grams), eight in each group and three groups in total according to our experimental requirements. Rats are purchased from Shanghai Jiesjie Experimental Animal Co., LTD. In our animal experiments, we are grouped into: sham-operated group (Sham), HO model group (Vehicle), HO model and administered Palovarotene group (Palo). Anesthesia was performed by intraperitoneal injection of 10% chloral hydration, control drug flow per minute. After fixation, hair removal and disinfection, a 0.5–1 cm longitudinal incision was made along the lateral superficial skin groove between tibiofibular bone and the Achilles tendon according to the marking point of the Achilles tendon. Cut the skin and subcutaneous tissue, step by step after exposure of the Achilles tendon tissue fully, to identify the middle point of the Achilles tendon, The Achilles tendon was clamped repeatedly with vascular forceps five times, once at the midpoint of the Achilles tendon, and then twice above and below the midpoint caused considerable trauma, in Model group and Palov group, we pricked the outer sheath membrane, to the middle point of tendon and surrounding tissues thoroughly after separation, use eye or knife cut transverse form completely cut off the Achilles tendon, then on both sides of the Achilles tendon end in normal tissue, in the sham operation group, only the Achilles tendon was exposed and then sutured. Penicillin was injected intramuscularly for 3 consecutive days after surgery at a dose of 800,000 IU/ days to prevent infection in the surgical field. Palovarotene was dissolved in DMSO solvent, and 100 ul oral solution was obtained by mixing 30 ul drug solution and 70 ul corn oil during administration. The Sham group and HO Model group were given DMSO+ corn oil solution in the same proportion, Palovarotene concentration was 1 mg/kg/ day, and Palovarotene was orally administered by no. 20 gavage needle. Throughout the 21-day period, the Palovarotene was administered continuously.

### Micro-CT

Micro-CT analysis was performed after 12 weeks of feeding. Achilles tendons with lower tibia and calcaneus from mice were fixed in 10 % formalin overnight. The X-ray tube settings were 50 kV and 60 uA and images were acquired at 50-um resolution. A 0.5 rotation step through a 360 angular range with a 50 ms exposure per step was used. The images were reconstructed and analyzed with Skysan 1275 software.

### Hematoxylin-Eosin Staining

The rats were sacrificed by excessive intraperitoneal injection of 10% chloral hydrate, and then the tissues below the bilateral knee joints of rats were immediately severed with a scalpel. Residual blood stains were washed with physiological saline for several time, then fixed in a 50 mL centrifuge tube containing 4% paraformaldehyde solution. After being fixed in 4% paraformaldehyde for 24–48 h, the Achilles tendon tissue were decalcified, dehydrated and embedded. The tissue sections required by the experiment were dewaxed and hydrated. The original hematoxylin solution and alcohol-soluble eosin solution were successively covered on the tissue surface for staining. After staining, the slices were sealed. Observed them under a microscope 24 h later.

### Statistical Analysis

Statistical analysis was performed using SPSS 25.0 software. Results are presented as mean ± standard deviation (x ± s). The comparison of multiple groups of independent data was performed by one-way analysis of variance. Pairwise comparisons between multiple groups were performed using the LSD-*t* test. *P* < 0.05 indicated that the difference was statistically significant.

## Results

### Intersection Target of Palovarotene and HO

**Figure 2:**
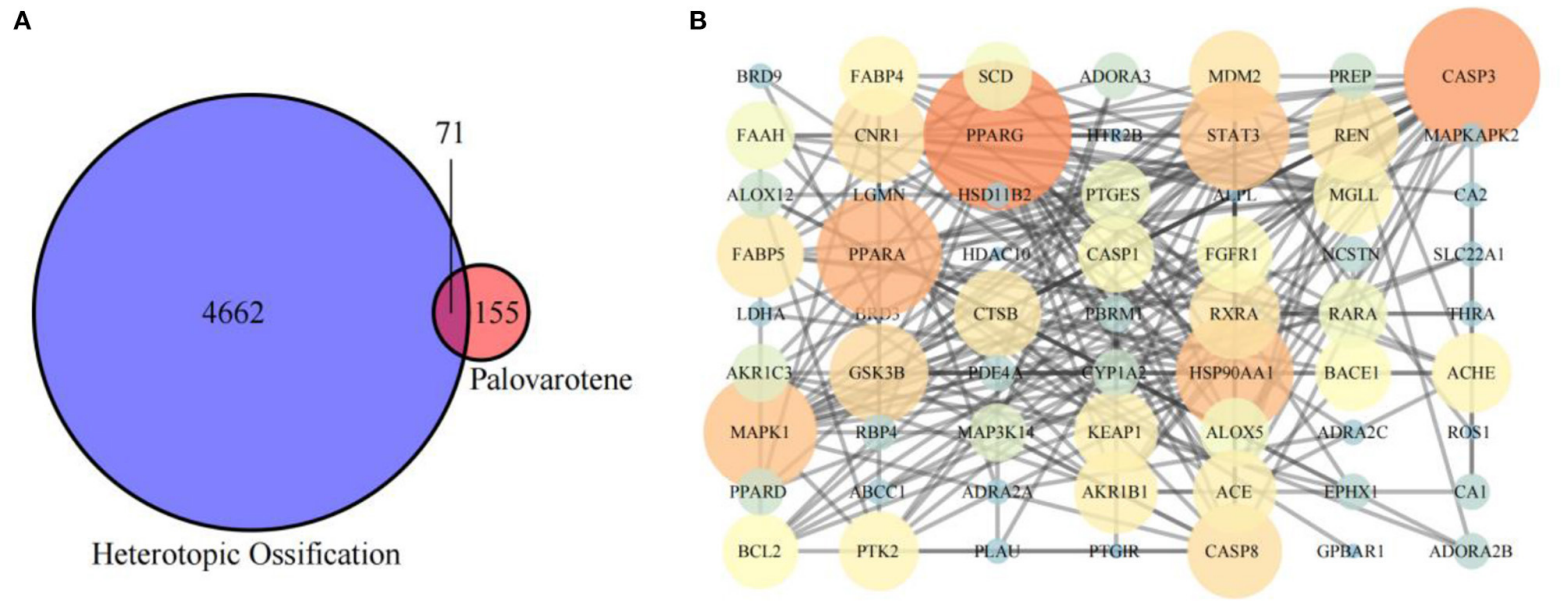
**(A)** Intersection of Palovarotene-related genes and heterotopic ossification related genes; **(B)** PPI network of potential targets of Palovarotene to treat heterotopic ossification.

We obtained 226 Palovarotene-related genes from ChEMBL and SwissTargetPrediction databases. 4733 HO related genes were obtained from DisGeNET and Comparative Toxicogenomics Database. Then, their intersection was taken to obtain 71 common targets of Palovarotene and HO, and this set is the possible target of Palovarotene for HO (Figure 2A).

### Construction of Palovarotene and HO Intersection Target Network and Target Protein-Protein Interaction Analysis

Intersection targets of Palovarotene-HO were imported into Cytoscape software to construct the compound-target interaction network diagram of Palovarotene's direct or indirect action against HO, and then the intersection targets were imported into String database (15). Seventy-one targets were reserved with a confidence score > 0.4 out of all the intersection targets. Acquire protein-protein interaction (PPI) relationships. Then Cytoscap was utilized to draw the network diagram of the relationship between targets. After deleting the nodes with fewer edges in PPI network, 63 targets were finally reserved for follow-up studies (Figure 2B and Supplementary Table 3).

### GO and KEGG Enrichment Analysis of Potential Targets

**Figure 3:**
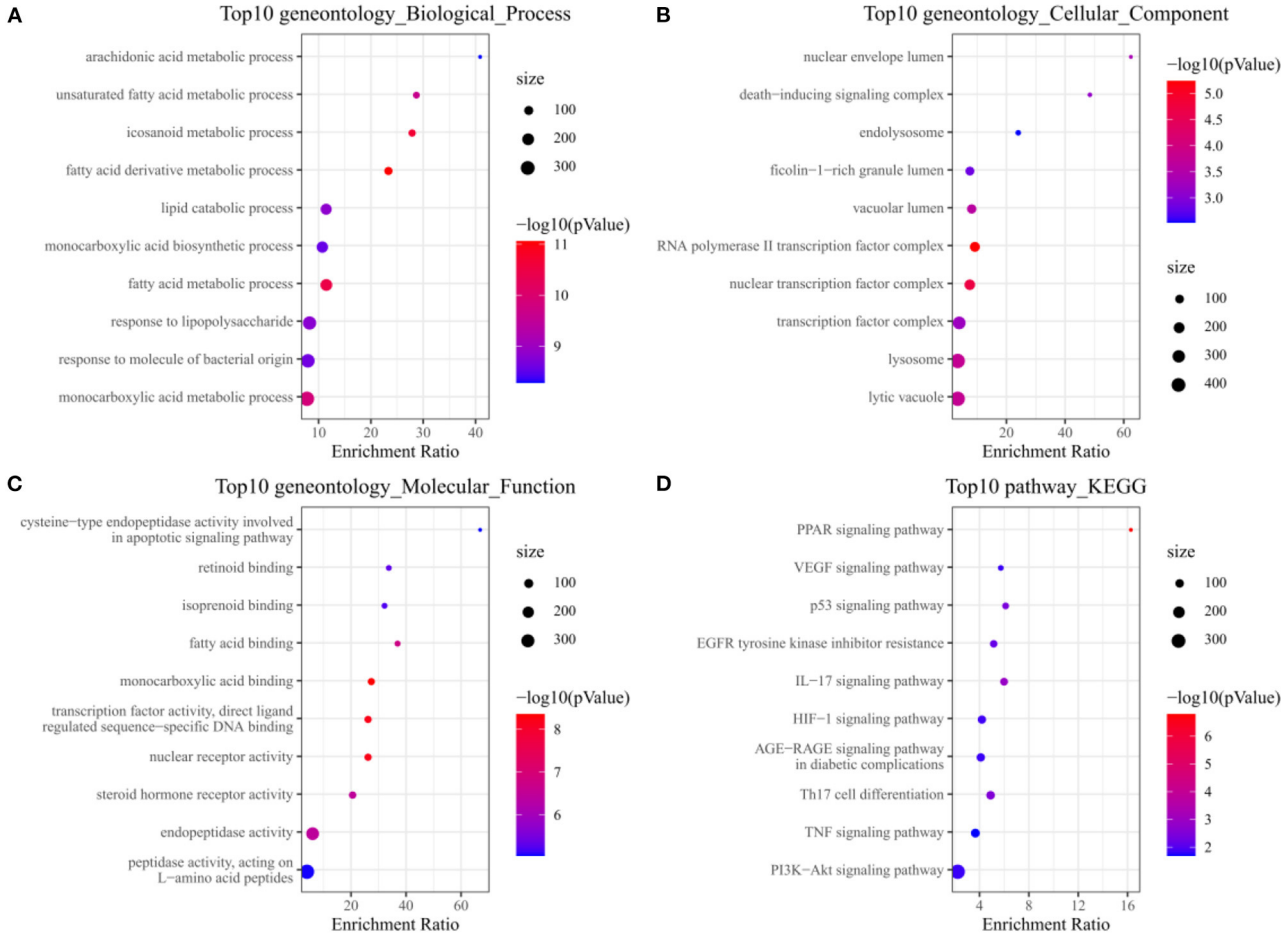
**(A)** Annotation diagram of biological process; **(B)** Annotation diagram of cellular component; **(C)** Annotation diagram of molecular function; **(D)** Annotation diagram of KEGG.

Analysis of GO and KEGG enrichment analysis on 63 targets was performed using WebGestaltR After systematic analysis, it was found that in the biological process of Palovarotene treatment of HO, the top 10 correlation of biological process GO entries (*p* < 0.05), including arachidonic acid metabolic process, unsaturated fatty acid metabolic process, icosanoid metabolic process, monocarboxylic acid metabolic process. Molecular function GO entries (*p* < 0.05), concluding cysteine-type endopeptidase activity involved in apoptotic signaling pathway, retinoid binding, fatty acid binding, peptidase activity, acting on L-amino acid peptides and cellular components GO entries (*p* < 0.05), including nuclear envelope lumen, death–inducing signaling complex, endolysosome, lytic vacuole. KEGG pathway enrichment found that P53 signaling pathway, PI3K-Akt signaling pathway, PPAR signaling pathway, VEGF signaling pathway, and AGE-RAGE and other signaling pathways were significantly enriched (Figure 3, original data can be found in Supplementary Material 1).

### Module-Based Network Analysis

**Figure 4:**
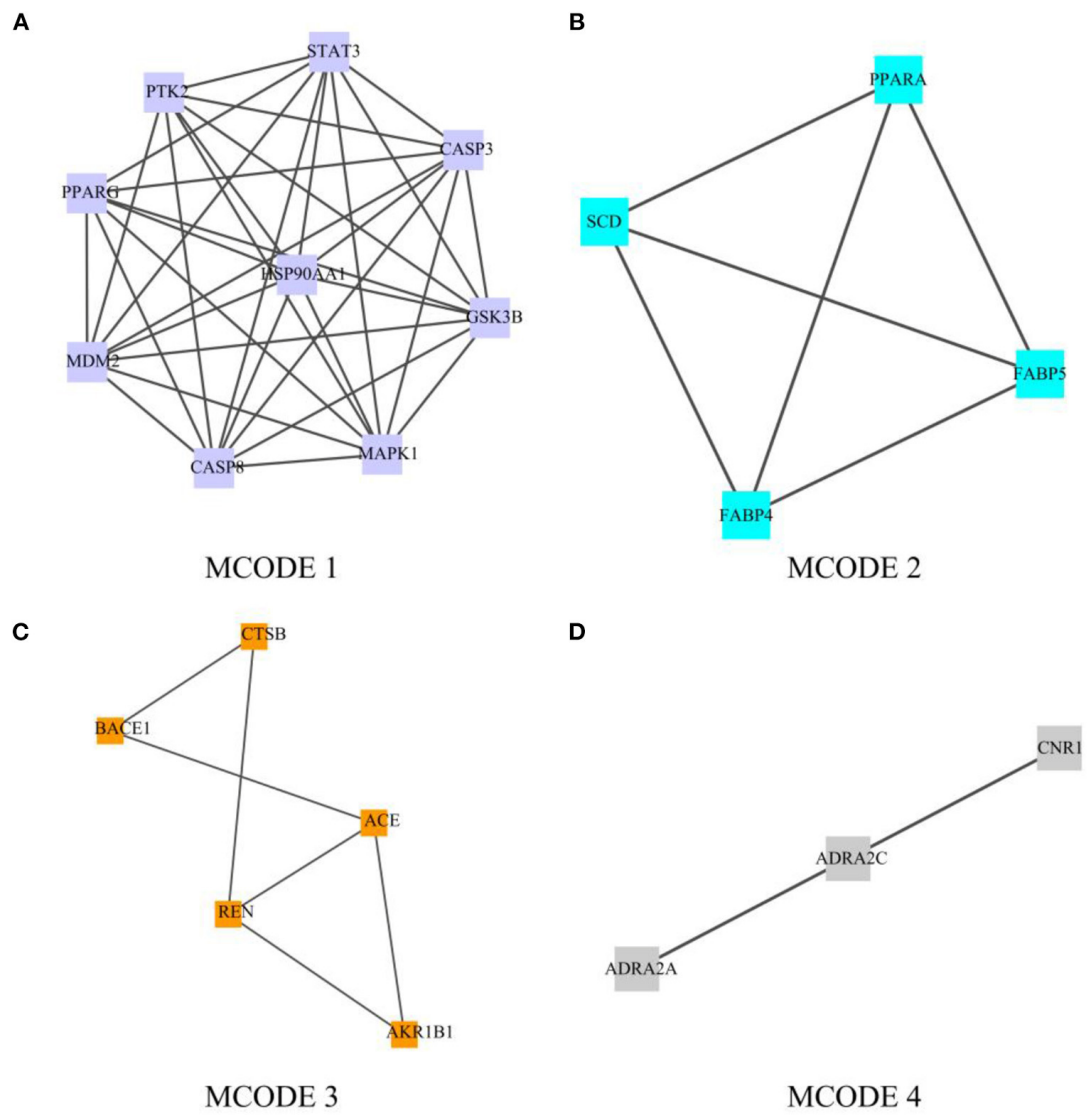
Module-based network analysis of potential targets of Palovarotene against heterotopic ossification. **(A–D)** Different functional modules in targets of Palovarotene against heterotopic ossification.

In order to screen out the 63 intersection targets highly associated targets of Palovarotene against HO, we performed a module-based network analysis using Metascape, and used the MCODE algorithm to screen these 63 intersecting targets with their respective mutual. The linked targets are called protein groups (Supplementary Material 2). These screened protein groups often have their own biological functions. GO and KEGG analysis of these protein groups found that these protein groups are related to p53 signaling pathway, PI3K-Akt signaling pathway, PPAR signaling pathway, cGMP-PKG signaling pathway, FoxO signaling pathway, etc. are significantly related (Figure 4, original data can be found in Supplementary Material 3).

### Topological Analysis of PPI Network

**Figure 5:**
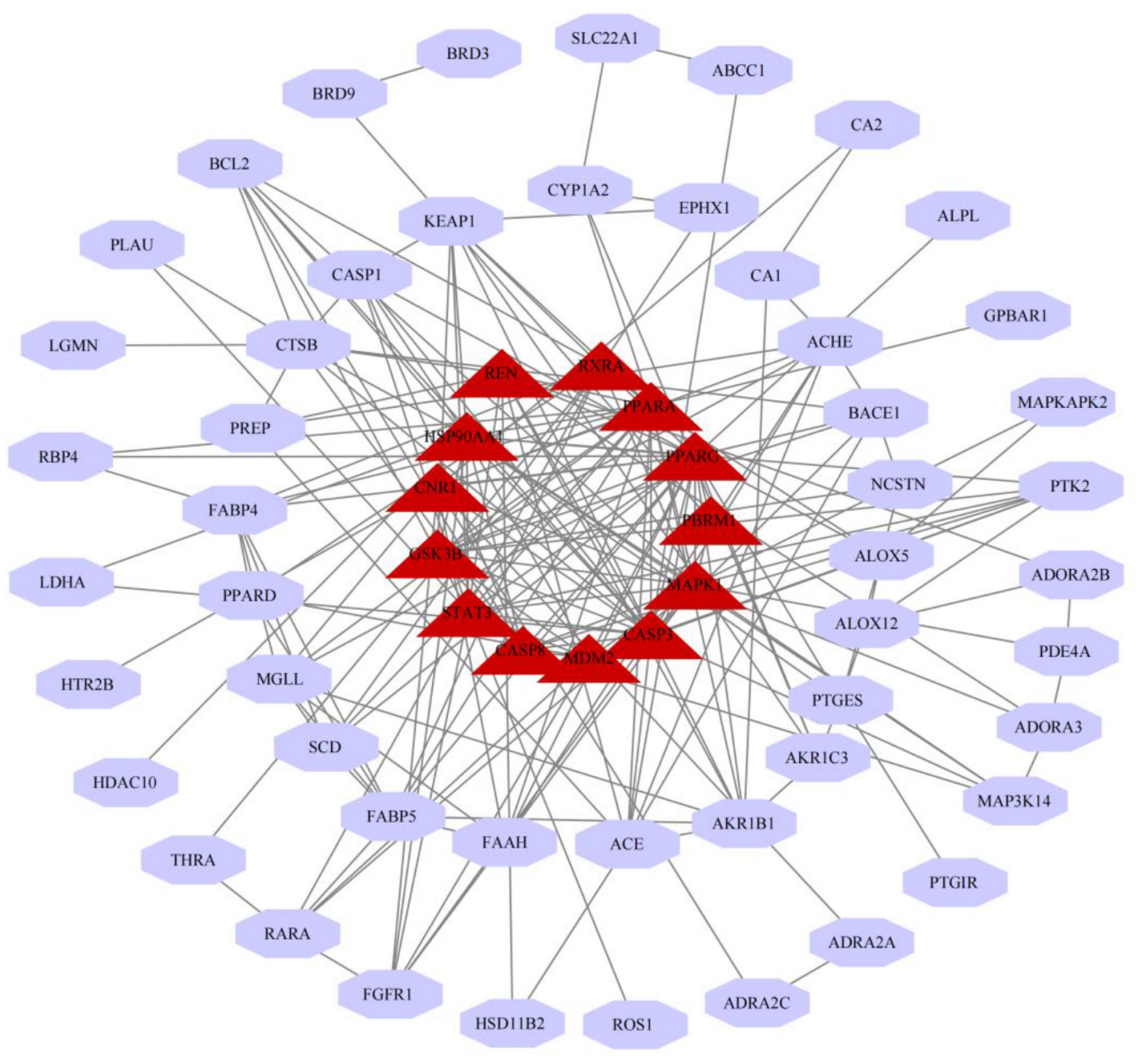
Topological analysis of PPI network. Red nodes on the inner circle indicate hub genes.

We have constructed a PPI network, and 63 targets were initially screened, and the 63 key targets were used to communicate with Cytoscape and visualized. Sort by topological parameters of degree, betweenness central, and closeness central, the highest number of nodes as Hub Genes (Figure 5). The more important of the rank is more important in the PPI network. In this network, PPARG, CASP3, PPARA, HSP90AA1, MAPK1, STAT3, GSK3B, CNR1, CASP8, REN, RXRA, MDM2, and PBRM and other genes were HUB Gene (Table 3).

**Table 3:** Topological parameters of hub genes in the PPI network.

**Number**	**Genes**	**Degree**	**Betweenness centrality**	**Closeness centrality**
1	PPARG	26	0.25539816	0.60784314
2	CASP3	23	0.15038421	0.58490566
3	PPARA	20	0.15050133	0.56363636
4	HSP90AA1	18	0.08021818	0.52542373
5	MAPK1	17	0.10085056	0.52542373
6	STAT3	16	0.06892525	0.51666667
7	GSK3B	13	0.02914279	0.496
8	CNR1	12	0.13782789	0.48062016
9	CASP8	12	0.00986503	0.484375
10	REN	11	0.06854053	0.48062016
11	RXRA	11	0.04907326	0.45588235
12	MDM2	11	0.02154882	0.47328244
13	PBRM1	3	0.06345849	0.41610738

### Gene-Phenotype Correlation Analysis of Anti-heterotopic Ossification Targets of Palovarotene

**Figure 6:**
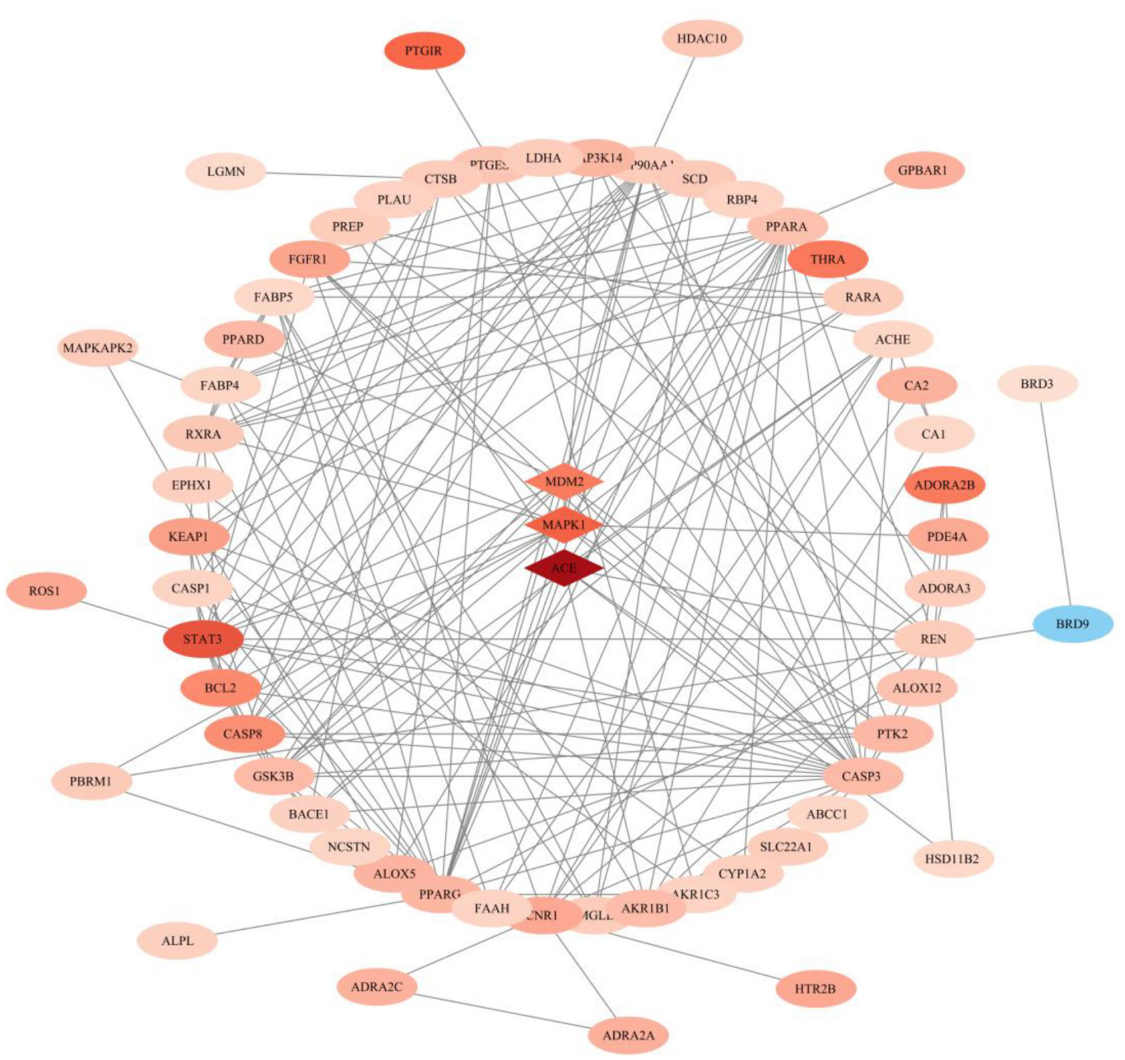
Gene–phenotype correlation analysis of anti- heterotopic ossification targets of Palovarotene. Intersection genes were categorized as directly (the diamond in the middle) or indirectly (outer ellipse) associated with the HO phenotype. The darker the color, the larger the score value, the lighter the color, the smaller the score value.

After performing Topological Analysis on the PPI network, we screened out the most relevant hub genes. For identifying the most core genes, we employed VarElect to perform Gene-Phenotype Correlation Analysis. The VarElect algorithm can verify the relationship between genes and phenotypes. The direct or indirect relationship can be used to screen out the genes with the highest phenotype correlation in a group of genes. The results showed that, of the 63 potential targets, three were directly related to the HO phenotype and 60 were indirectly related (Table 4). Directly related targets are Angiotensin I Converting Enzyme (ACE), Mitogen-Activated Protein Kinase 1 (MAPK1), and MDM2 Proto-Oncogene (MDM2) (Figure 6).

**Table 4:** Top 20 targets directly or indirectly associated with the HO phenotype.

**Number**	**Symbol**	**Description**	**Direct/indirect**	**Score**	**Average disease causing likelihood**
1	ACE	Angiotensin I Converting Enzyme	Direct	2.38	15.8
2	STAT3	Signal Transducer And Activator Of Transcription 3	Indirect	1.97	78.9
3	PTGIR	Prostaglandin I2 Receptor	Indirect	1.36	43.3
4	MAPK1	Mitogen-Activated Protein Kinase 1	Direct	1.31	73.8
5	THRA	Thyroid Hormone Receptor Alpha	Indirect	1.17	89.8
6	ADORA2B	Adenosine A2b Receptor	Indirect	1.16	58.2
7	CASP8	Caspase 8	Indirect	1.13	68.6
8	BCL2	BCL2 Apoptosis Regulator	Indirect	1.1	48.6
9	MDM2	MDM2 Proto-Oncogene	Direct	1.03	86.2
10	FGFR1	Fibroblast Growth Factor Receptor 1	Indirect	1.01	76.3
11	ROS1	ROS Proto-Oncogene 1, Receptor Tyrosine Kinase	Indirect	0.89	22.3
12	PDE4A	Phosphodiesterase 4A	Indirect	0.82	57.4
13	CA2	Carbonic Anhydrase 2	Indirect	0.8	60.1
14	KEAP1	Kelch Like ECH Associated Protein 1	Indirect	0.8	86.2
15	PPARG	Peroxisome Proliferator Activated Receptor Gamma	Indirect	0.73	58.4
16	CNR1	Cannabinoid Receptor 1	Indirect	0.7	87.5
17	HTR2B	5-Hydroxytryptamine Receptor 2B	Indirect	0.7	20.5
18	PTK2	Protein Tyrosine Kinase 2	Indirect	0.68	83
19	ADRA2A	Adrenoceptor Alpha 2A	Indirect	0.65	43.2
20	CASP3	Caspase 3	Indirect	0.63	64.2

### After Administration of Palovarotene, the Osteogenic Differentiation of BMSCs Was Inhibited and the Volume of Heterotopic Bone Was Significantly Reduced in Animal Models

**Figure 7:**
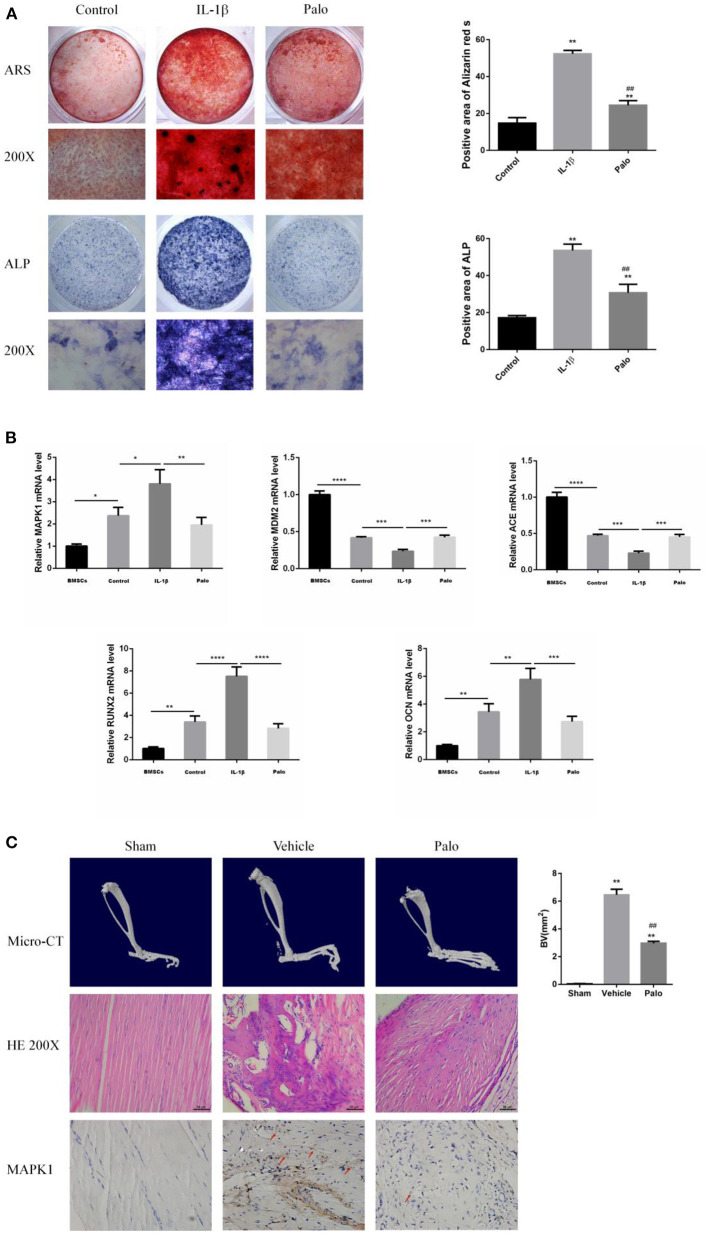
Screening target validation and Palovarotene against HO validation experiments. **(A)** ALP and ARS staining in BMSCs after culture with osteogenesis induction. Data are means ± SD (*n* = 3) *P < 0.05, **P < 0.01, * compared with the Control group. ^#^*P* < 0.05, ^##^*P* < 0.01, and # compared with the IL-1β group. Scale bar: 100 μm; **(B)** The mRNA expression and relevant quantitative analysis of MAPK1, MDM2, ACE, Runx2, and OCN. Data are means ± SD (*n* = 3) *P < 0.05, **p < 0.01, ***P < 0.001, and ****P < 0.0001; **(C)** images of Micro-CT, HE staining and IHC. Data are means ± SD (*n* = 8) *P < 0.05, **p < 0.01, and * compared with the Sham group. ^#^*P* < 0.05, ^##^*P* < 0.01, and # compared with the Vehicle group. Scale bar =50 μm.

In *in vitro* experiments, we are grouped into: Control group (Control), IL-1β group (IL-1β), IL-1β group plus Palovarotene group (Palo), in the PCR experiments performed *in vitro*, we added an additional group of BMSCs that were cultured without osteogenic induction medium but with normal medium. In animal experiments, we are grouped into: sham-operated group (Sham), HO model group (Vehicle), HO model, and administered Palovarotene group (Palo). *In vitro*, after culturing BMSCs for 14–21 days after osteogenic induction, we performed ARS, ALP staining, and RT-PCR experiments *in vitro*. The results showed that under the osteogenic induction and the stimulation of IL-1β, the calcium deposition and alkaline phosphatase content of BMSCs were significantly increased, and the calcium deposition and alkaline phosphatase are classic markers of osteogenic differentiation, and the expression and transcription levels of osteogenic-related molecules OCN and RUNX2 were significantly up-regulated. This indicated that BMSCs differentiated into osteoblasts in the context of osteogenic induction. The PCR results also showed that the transcription levels of the key molecules MAPK1 and MDM2 we screened were significantly regulated, MAPK1 is a positive regulator of PI3K–Akt signaling pathway, while MDM2 is a key negative regulator of p53 signaling pathway. The results showed that MAPK1 was significantly up-regulated, while MDM2 was significantly down-regulated. After administration of Palovarotene, the above trend was reversed, the calcium deposition and alkaline phosphatase contents were significantly reduced, and the osteogenesis-related molecules and the screened key molecules transcriptions were down-regulated. In our animal model, Micro-CT showed that the model group produced obvious ectopic bone, while the ectopic bone formation in the sham-operated group and the Palovarotene-administered group was significantly inhibited. A large number of disorganized ectopic bones were generated between tissues, while in the sham-operated group and the Palovarotene-administered group, the trend of the model group was suppressed, and there was no obvious abnormality except for the partial tissue destruction caused by modeling. Immunohistochemical results showed that the positive rate of MAPK1, a positive regulator of PI3K-Akt signaling pathway, was higher in Vehicle group, but lower in Sham group and Palo Group. The above results suggest that the PI3K-Akt signaling pathway is activated when HO occurs, and the administration of Palovarotene can effectively inhibit the activation of PI3K-Akt signaling pathway, which is consistent with our cell validation experiment (Figure 7).

## Discussion

Clinically, heterotopic ossification is currently mainly divided into two types: one is hereditary heterotopic ossification, with low incidence but severe symptoms and high mortality; the other is acquired heterotopic ossification, with mild symptoms and death. The incidence is low, but the morbidity is high. The severity of symptoms in patients is often proportional to the severity of the trauma, and is closely related to the site of the trauma and the source of the trauma (3). Because the pathogenesis of heterotopic ossification is complex, it involves heredity and gene mutation, the participation of osteogenic precursor cells, the up-regulation of BMP signal transduction pathway, and the overexpression of BMP (16). At present, the treatment methods of heterotopic ossification include drug therapy, radiotherapy, surgery, rehabilitation therapy, etc. (17). Since the exact mechanism of the formation of heterotopic ossification is still unclear, there is no clear drug for the treatment of heterotopic ossification (18). The reason is that the targets of the currently isolated and purified drugs are various, and it is impossible to truly screen a drug against heterotopic ossification through clinical practice. Palovarotene is a retinoic acid receptor agonist. Studies have shown that this receptor interacts with bone morphogenetic protein (BMP), which is shown to prevent ectopic bone and blood vessel formation, and inhibit osteogenic gene expression (19), it can also be used in combination with corticosteroids to treat heterotopic ossification (20). Through the above studies, it can be found that Palovarotene can improve disease progression through multiple pathways and targets, which is a feature of drugs for complex diseases.

Network pharmacology explains the occurrence and development of diseases from the perspective of systems biology and biological network balance, understands the interaction between drugs and the body from the overall perspective of improving or restoring biological network balance, and guides the discovery of new drugs (21). It emphasizes the analysis of molecular associations between drugs and treatment of diseases from the perspective of the system level and the overall biological network (22). Considering that Palovarotene has the characteristics of being multi-target, and multi-pathways for treating diseases, it shows great promise for treating HO. Compared with traditional pharmacology, network pharmacology can comprehensively explain the potential mechanism of drug action by scientific analysis methods. The potential mechanism of action in the treatment of heterotopic ossification provides new scientific and technological support for clinical rational drug use, interpretation of the overall mechanism of action, and analysis of the law of drug combination (23).

To evaluate the efficacy of Palovarotene in the treatment of heterotopic ossification, a network pharmacology analysis was performed. First, 71 potential targets of Palovarotene for the treatment of heterotopic ossification were obtained through Venn analysis by searching different databases. After constructing PPI, edge nodes were removed, and 63 targets were obtained for follow-up research. Afterwards, to elucidate the multiple mechanisms by which Palovarotene treats HO, we performed GO and KEGG enrichment analysis on these targets. The GO results indicated that the potential targets of Palovarotene in the treatment of heterotopic ossification are closely related to the following molecular functions: cysteine-type endopeptidase activity involved in apoptotic signaling pathway, retinoid binding, fatty acid binding, peptidase activity, acting on L-amino acid peptides; closely related to the following biological processes: arachidonic acid metabolic process, unsaturated fatty acid metabolic process, icosanoid metabolic process, monocarboxylic acid metabolic process. It is closely related to the following cellular components: nuclear envelope lumen, death-inducing signaling complex, endolysosome, and lytic vacuole. The results suggest that many molecules, cellular components, and biological processes may play a role in the signal transduction and metabolism of Palovarotene-treated HO, and these molecules, cellular components, and biological processes are highly correlated with the results of our screening of core targets. In addition, the results of KEGG enrichment analysis showed that the signaling pathways involving these targets in heterotopic ossification diseases mainly include PI3K-Akt signaling pathway, PPAR signaling pathway, P53 signaling pathway, and VEGF signaling pathway. Dong et al. demonstrated that the PTEN/PI3K/AKT signaling pathway plays a key role in the development of ectopic bone formation (24). Targeting the PI3K/AKT pathway with inhibitors may be a potential molecular therapy for HO (25). Chen et al. confirmed that the activation of PI3K-Akt signaling pathway by leptin combined with mechanical stress stimulation plays an important role in the ossification of the posterior longitudinal ligament (26). Hwang et al. found that mesenchymal-derived VEGFA is an important expression signal in the occurrence of heterotopic ossification, and the treatment of VEGF pathway may reduce the occurrence of heterotopic ossification (27). AGE/RAGE signaling heavily influences both cellular and systemic responses to increase bone matrix proteins through PKC, p38 MAPK, fetuin-A, TGF-β, NFκB, and ERK1/2 signaling pathways in both hyperglycemic and calcification conditions (28) and researcher found that the Advanced Glycation End-Products (AGE)/Receptor for AGEs (RAGE) signaling pathway exacerbates diabetes-mediated vascular calcification (VC) in vascular smooth muscle cells (VSMCs), so the relationship between age-rage signaling pathway and osteogenesis was confirmed (29). In recent years, more and more experimental studies have shown that retinoic acid receptors play a negative regulatory role in the process of osteogenesis and chondrogenesis, and chondrogenic differentiation under physiological conditions requires a low concentration of endogenous retinoic acid (30). The researchers used a retinoic acid receptor agonist to effectively prevent heterotopic ossification in mice and found that the effect continued even after the mice were no longer taking the agonist (31). In additions, the nuclear transcription factor peroxisome-proliferator-activated receptor-gamma (PPAR-γ) has been found expressed in both osteoblasts and adipocytes, as well as in mesenchymal stem cells, suggesting its crucial role in regulating adipocyte formation and osteoblast development. In addition, Ju et al. found that in models of heterotopic ossification such as traumatic brain injury/burn/tenotomy, the NF-kB/p53 signaling pathway plays an important role in the occurrence and development of heterotopic ossification, and ammonium pyrrolidine dithiocarbamate Pharmacological inhibition of NF-kB signaling pathway (PDTC) can significantly reduce the expression level of p53 and the size of heterotopic ossification (32).

Further targeting of Palovarotene in the treatment of heterotopic ossification, we performed a modular network analysis of parovatine's anti-heterotopic ossification targets. The result of the modular network analysis is likely to be a protein group that plays a role in the treatment of HO with Palovarotene, so we propose a modular network analysis method to highlight the protein group of parovarotene in the treatment of heterotopic ossification. The results show that these protein groups are closely related to p53 signaling pathway, PI3K-Akt signaling pathway, PPAR signaling pathway, cGMP-PKG signaling pathway, FoxO signaling pathway, etc., which are very similar to our KEGG results. Next, we further conducted topological analysis of the PPI network, and we screened out PPARG, PPARA, MDM2, MAPK1, and other genes as hub genes that are highly related to the HO process of Palovarotene treatment, which is consistent with the above GO and KEGG enrichment analysis results and Module-Based. The results of Network Analysis are consistent, both PPARG and PPARA are related to PPAR signaling pathway; MDM2 is a key negative regulator of p53 signaling pathway; MAPK1 is an important component of PI3K-Akt signaling pathway. Finally, in order to confirm the relationship between the screened key genes and the HO phenotype, we carried out Gene–Phenotype Correlation Analysis, the results showed that PPARG, MAPK1, MDM2, and other targets are directly related to the HO phenotype, among which the role of MAPK1 and MDM2 most closely, our research has gradually confirmed that MAPK1 and MDM2 may be the core targets of Palovarotene in the treatment of heterotopic ossification, and the corresponding PI3K-Akt signaling pathway and p53 signaling pathway are likely to be involved in the treatment process play an important role in. To verify our point, we conduct a series of verification experiments.

The verification experiment found that in the osteogenic induction environment, the calcium deposition and alkaline phosphatase content of BMSCs were significantly increased, and the expression levels of osteogenesis-related molecules OCN and RUNX2 were significantly up-regulated. The results indicated that BMSCs differentiated into osteoblasts, which confirmed the validity of our *in vitro* model. The PCR results showed that the transcription levels of the key molecules MAPK1 and MDM2 we screened were significantly up-regulated. After administration of Palovarotene, the above trend was reversed, the calcium deposition and alkaline phosphatase contents were significantly reduced, and the osteogenesis-related molecules and the screened key molecules were transcribed level down. In our animal model, Micro-CT showed that the model group produced obvious ectopic bone, while the ectopic bone formation in the sham-operated group and the Palovarotene-administered group was significantly inhibited, and the volume of the ectopic bone was significantly reduced. Consistent with Mircro-CT, in the model group, the muscle tissue was disordered, angiogenesis was formed, and a large number of disorganized ectopic bones were generated between tissues, while in the sham-operated group and the administered Palovarotene group, the trend of the model group was suppressed, except for some tissues caused by modeling. No obvious abnormality was found outside the damage. Our results show that when BMSCs differentiate into osteoblasts, MAPK1 and MDM2 play a role and their transcription levels are up-regulated. After administration of Palovarotene, the transcription levels are decreased, indicating that Palovarotene is likely to act on MAPK1, MDM2 targets and their corresponding PI3K- Akt signaling pathway and p53 signaling pathway play a role.

This study attempted to clarify a key target and mechanism of Palovarotene in the treatment of heterotopic ossification, screened out the possible core targets and related signaling pathways by means of network pharmacology, and carried out verification experiments. Our study confirmed the role of the screened core targets in the treatment of HO with Palovarotene, and confirmed the effectiveness of Palovarotene in the treatment of HO. Nevertheless, there are some limitations to our study. Although we have collected a large database for parovatine-related targets and heterotopic ossification-related targets, the data are certainly incomplete and some of the latest studies have not been uncovered. Secondly, our study found that the mechanism of Palovarotene in the treatment of HO is complex and multi-factorial. It's not sufficiently investigated for the details of its relevant molecular mechanism, and there is a lack of further experimental verification. More experiments are needed to verify these goals and key points path.

## Data Availability Statement

The datasets presented in this study can be found in online repositories. The names of the repository/repositories and accession number(s) can be found in the article/Supplementary Material.

## Ethics Statement

The animal study was reviewed and approved by the Bioethics Committee of the Shanghai Sixth People's Hospital.

## Author Contributions

JH and HX: conceptualization. JH: methodology, formal analysis, resources, data curation, writing—original draft preparation, and visualization. JH and DL: software and writing—review and editing. JZ: validation. DL and JZ: investigation. HX: supervision, project administration, and funding acquisition. All authors have read and agreed to the published version of the manuscript.

## Funding

This research was funded by Shanghai Fengxian District Health Committee, grant number fxlczlzx-a-202103.

## Conflict of Interest

The authors declare that the research was conducted in the absence of any commercial or financial relationships that could be construed as a potential conflict of interest.

## Publisher's Note

All claims expressed in this article are solely those of the authors and do not necessarily represent those of their affiliated organizations, or those of the publisher, the editors and the reviewers. Any product that may be evaluated in this article, or claim that may be made by its manufacturer, is not guaranteed or endorsed by the publisher.
