# Supplementary material for: A Network Pharmacology Study: Reveal the Mechanisms of Palovarotene Against Heterotopic Ossification
Source: Front Med (Lausanne). 2022 May 13;9:897392. doi: 10.3389/fmed.2022.897392 (PMC9136101; doi:10.3389/fmed.2022.897392)
Supplement: Supplementary file 1 [file Supplementary_Material_1.docx]

geneSet description link size overlap expect enrichmentRatio pValue FDR overlapId userId DB

hsa03320 PPAR signaling pathway http://www.kegg.jp/kegg-bin/show_pathway?hsa03320+2167+2171+5465+5467+5468+6256+6319 44 7 0.428958295721249 16.3186026936027 1.62825467775463e-07 5.11271968814953e-05 2167;2171;5465;5467;5468;6256;6319 PPARG;PPARA;FABP5;RXRA;FABP4;PPARD;SCD pathway_KEGG

hsa05215 Prostate cancer http://www.kegg.jp/kegg-bin/show_pathway?hsa05215+2260+2932+3320+4193+5328+5594+596 94 7 0.916410904495396 7.63849487785658 2.99251672977174e-05 0.00420805099429026 2260;2932;3320;4193;5328;5594;596 GSK3B;BCL2;MAPK1;HSP90AA1;MDM2;FGFR1;PLAU pathway_KEGG

hsa05200 Pathways in cancer http://www.kegg.jp/kegg-bin/show_pathway?hsa05200+2260+2932+3320+4193+5467+5468+5594+5747+5914+596+6256+6774+836+841+9817 487 15 4.74778840946019 3.15936573123431 4.02043088626458e-05 0.00420805099429026 2260;2932;3320;4193;5467;5468;5594;5747;5914;596;6256;6774;836;841;9817 CASP3;PPARG;GSK3B;KEAP1;PTK2;BCL2;MAPK1;STAT3;HSP90AA1;CASP8;RXRA;MDM2;FGFR1;PPARD;RARA pathway_KEGG

hsa00590 Arachidonic acid metabolism http://www.kegg.jp/kegg-bin/show_pathway?hsa00590+239+240+8644+9536 32 4 0.311969669615454 12.8217592592593 0.00023702800934311 0.015116565401358 239;240;8644;9536 ALOX12;AKR1C3;PTGES;ALOX5 pathway_KEGG

hsa01524 Platinum drug resistance http://www.kegg.jp/kegg-bin/show_pathway?hsa01524+4193+5594+596+836+841 61 5 0.594692182704459 8.4077109896782 0.000286751344313219 0.015116565401358 4193;5594;596;836;841 CASP3;BCL2;MAPK1;CASP8;MDM2 pathway_KEGG

hsa04625 C-type lectin receptor signaling pathway http://www.kegg.jp/kegg-bin/show_pathway?hsa04625+4193+5594+834+841+9020+9261 95 6 0.926159956670879 6.47836257309942 0.000288851568178816 0.015116565401358 4193;5594;834;841;9020;9261 MAP3K14;MAPKAPK2;MAPK1;CASP8;CASP1;MDM2 pathway_KEGG

hsa05145 Toxoplasmosis http://www.kegg.jp/kegg-bin/show_pathway?hsa05145+240+5594+596+6774+836+841 111 6 1.08214479147861 5.54454454454454 0.000667926632958116 0.0299612803926926 240;5594;596;6774;836;841 CASP3;ALOX5;BCL2;MAPK1;STAT3;CASP8 pathway_KEGG

hsa00790 Folate biosynthesis http://www.kegg.jp/kegg-bin/show_pathway?hsa00790+231+249+8644 20 3 0.194981043509659 15.3861111111111 0.000888103952784602 0.0348580801467956 231;249;8644 AKR1B1;AKR1C3;ALPL pathway_KEGG

hsa04614 Renin-angiotensin system http://www.kegg.jp/kegg-bin/show_pathway?hsa04614+1636+5550+5972 21 3 0.204730095685142 14.6534391534392 0.00102900226897618 0.0359007458287244 1636;5550;5972 ACE;REN;PREP pathway_KEGG

hsa04657 IL-17 signaling pathway http://www.kegg.jp/kegg-bin/show_pathway?hsa04657+2932+3320+5594+836+841 84 5 0.818920382740567 6.10559964726631 0.00125603741669056 0.0386378740303651 2932;3320;5594;836;841 CASP3;GSK3B;MAPK1;HSP90AA1;CASP8 pathway_KEGG

hsa04210 Apoptosis http://www.kegg.jp/kegg-bin/show_pathway?hsa04210+1508+5594+596+836+841+9020 127 6 1.23812962628633 4.84601924759405 0.00135355609660515 0.0386378740303651 1508;5594;596;836;841;9020 CASP3;MAP3K14;CTSB;BCL2;MAPK1;CASP8 pathway_KEGG

hsa05205 Proteoglycans in cancer http://www.kegg.jp/kegg-bin/show_pathway?hsa05205+2260+4193+5328+5594+5747+6774+836 183 7 1.78407654811338 3.92359846184983 0.00182303048557375 0.0430663199923119 2260;4193;5328;5594;5747;6774;836 CASP3;PTK2;MAPK1;STAT3;MDM2;FGFR1;PLAU pathway_KEGG

hsa05010 Alzheimer disease http://www.kegg.jp/kegg-bin/show_pathway?hsa05010+23385+23621+2932+5594+836+841 136 6 1.32587109586568 4.52532679738562 0.00192430672081134 0.0430663199923119 23385;23621;2932;5594;836;841 CASP3;BACE1;GSK3B;NCSTN;MAPK1;CASP8 pathway_KEGG

hsa04726 Serotonergic synapse http://www.kegg.jp/kegg-bin/show_pathway?hsa04726+239+240+3357+5594+836 93 5 0.906661852319913 5.5147351652728 0.00197896860443558 0.0430663199923119 239;240;3357;5594;836 CASP3;ALOX12;ALOX5;MAPK1;HTR2B pathway_KEGG

hsa05203 Viral carcinogenesis http://www.kegg.jp/kegg-bin/show_pathway?hsa05203+4193+5594+6774+836+83933+841+9261 190 7 1.85231991334176 3.77904483430799 0.00225955680281931 0.0430663199923119 4193;5594;6774;836;83933;841;9261 CASP3;MAPKAPK2;MAPK1;STAT3;CASP8;MDM2;HDAC10 pathway_KEGG

hsa04621 NOD-like receptor signaling pathway http://www.kegg.jp/kegg-bin/show_pathway?hsa04621+1508+3320+5594+596+834+841 142 6 1.38436540891858 4.33411580594679 0.00239581069330508 0.0430663199923119 1508;3320;5594;596;834;841 CTSB;BCL2;MAPK1;HSP90AA1;CASP8;CASP1 pathway_KEGG

hsa05206 MicroRNAs in cancer http://www.kegg.jp/kegg-bin/show_pathway?hsa05206+4193+5328+5594+596+6774+836 144 6 1.40386351326954 4.27391975308642 0.00257090918176961 0.0430663199923119 4193;5328;5594;596;6774;836 CASP3;BCL2;MAPK1;STAT3;MDM2;PLAU pathway_KEGG

hsa04080 Neuroactive ligand-receptor interaction http://www.kegg.jp/kegg-bin/show_pathway?hsa04080+1268+136+140+150+152+3357+5739+7067 249 8 2.42751399169525 3.29555258069314 0.00257234425756603 0.0430663199923119 1268;136;140;150;152;3357;5739;7067 CNR1;ADORA2B;ADORA3;ADRA2A;ADRA2C;HTR2B;PTGIR;THRA pathway_KEGG

hsa04928 Parathyroid hormone synthesis, secretion and action http://www.kegg.jp/kegg-bin/show_pathway?hsa04928+2260+5141+5594+596+6256 99 5 0.965156165372811 5.1805087916199 0.00260592382119085 0.0430663199923119 2260;5141;5594;596;6256 PDE4A;BCL2;MAPK1;RXRA;FGFR1 pathway_KEGG

hsa04659 Th17 cell differentiation http://www.kegg.jp/kegg-bin/show_pathway?hsa04659+3320+5594+5914+6256+6774 102 5 0.99440332189926 5.02814088598402 0.00296852679305815 0.0466058706510129 3320;5594;5914;6256;6774 MAPK1;STAT3;HSP90AA1;RXRA;RARA pathway_KEGG

hsa05160 Hepatitis C http://www.kegg.jp/kegg-bin/show_pathway?hsa05160+2932+5465+5594+6256+6774 106 5 1.03339953060119 4.83839972047519 0.00350720509768609 0.0471938720997162 2932;5465;5594;6256;6774 PPARA;GSK3B;MAPK1;STAT3;RXRA pathway_KEGG

hsa04215 Apoptosis http://www.kegg.jp/kegg-bin/show_pathway?hsa04215+596+836+841 32 3 0.311969669615454 9.61631944444444 0.00355776592160817 0.0471938720997162 596;836;841 CASP3;BCL2;CASP8 pathway_KEGG

hsa04115 p53 signaling pathway http://www.kegg.jp/kegg-bin/show_pathway?hsa04115+4193+596+836+841 66 4 0.643437443581874 6.21661054994388 0.00371924728356965 0.0471938720997162 4193;596;836;841 CASP3;BCL2;CASP8;MDM2 pathway_KEGG

hsa05221 Acute myeloid leukemia http://www.kegg.jp/kegg-bin/show_pathway?hsa05221+5467+5594+5914+6774 66 4 0.643437443581874 6.21661054994388 0.00371924728356965 0.0471938720997162 5467;5594;5914;6774 MAPK1;STAT3;PPARD;RARA pathway_KEGG

hsa05163 Human cytomegalovirus infection http://www.kegg.jp/kegg-bin/show_pathway?hsa05163+2932+4193+5594+5747+6774+836+841 208 7 2.02780285250045 3.45201210826211 0.00375747389328951 0.0471938720997162 2932;4193;5594;5747;6774;836;841 CASP3;GSK3B;PTK2;MAPK1;STAT3;CASP8;MDM2 pathway_KEGG

hsa04919 Thyroid hormone signaling pathway http://www.kegg.jp/kegg-bin/show_pathway?hsa04919+2932+4193+5594+6256+7067 110 5 1.07239573930312 4.66245791245791 0.00411311124590119 0.0496737281235759 2932;4193;5594;6256;7067 GSK3B;MAPK1;RXRA;MDM2;THRA pathway_KEGG

hsa05216 Thyroid cancer http://www.kegg.jp/kegg-bin/show_pathway?hsa05216+5468+5594+6256 37 3 0.360714930492869 8.31681681681682 0.0053854016233037 0.0626302262858282 5468;5594;6256 PPARG;MAPK1;RXRA pathway_KEGG

hsa05167 Kaposi sarcoma-associated herpesvirus infection http://www.kegg.jp/kegg-bin/show_pathway?hsa05167+2932+5594+6774+836+841+9261 170 6 1.6573388698321 3.6202614379085 0.0058305468756682 0.0653854185342791 2932;5594;6774;836;841;9261 CASP3;GSK3B;MAPKAPK2;MAPK1;STAT3;CASP8 pathway_KEGG

hsa05202 Transcriptional misregulation in cancer http://www.kegg.jp/kegg-bin/show_pathway?hsa05202+4193+5328+5468+5747+5914+6256 174 6 1.69633507853403 3.53703703703704 0.00652081043086805 0.0706046370790541 4193;5328;5468;5747;5914;6256 PPARG;PTK2;RXRA;MDM2;PLAU;RARA pathway_KEGG

hsa01521 EGFR tyrosine kinase inhibitor resistance http://www.kegg.jp/kegg-bin/show_pathway?hsa01521+2932+5594+596+6774 78 4 0.760426069687669 5.26020892687559 0.00675443096010331 0.0706963773824146 2932;5594;596;6774 GSK3B;BCL2;MAPK1;STAT3 pathway_KEGG

hsa04932 Non-alcoholic fatty liver disease (NAFLD) http://www.kegg.jp/kegg-bin/show_pathway?hsa04932+2932+5465+6256+836+841 128 5 1.24787867846182 4.00679976851852 0.00779142134252631 0.0789195581146213 2932;5465;6256;836;841 CASP3;PPARA;GSK3B;CASP8;RXRA pathway_KEGG

hsa05169 Epstein-Barr virus infection http://www.kegg.jp/kegg-bin/show_pathway?hsa05169+4193+596+6774+836+841+9020 184 6 1.79382560028886 3.34480676328502 0.00850607797535008 0.0834658901331227 4193;596;6774;836;841;9020 CASP3;MAP3K14;BCL2;STAT3;CASP8;MDM2 pathway_KEGG

hsa05161 Hepatitis B http://www.kegg.jp/kegg-bin/show_pathway?hsa05161+5594+596+6774+836+841 132 5 1.28687488716375 3.88538159371493 0.00884634600292289 0.0841743225732663 5594;596;6774;836;841 CASP3;BCL2;MAPK1;STAT3;CASP8 pathway_KEGG

hsa05210 Colorectal cancer http://www.kegg.jp/kegg-bin/show_pathway?hsa05210+2932+5594+596+836 86 4 0.838418487091533 4.770887166236 0.00949632754629204 0.0877013779275206 2932;5594;596;836 CASP3;GSK3B;BCL2;MAPK1 pathway_KEGG

hsa04217 Necroptosis http://www.kegg.jp/kegg-bin/show_pathway?hsa04217+3320+596+6774+834+841 137 5 1.33562014804116 3.74357934576913 0.010299775902208 0.0924037038083802 3320;596;6774;834;841 BCL2;STAT3;HSP90AA1;CASP8;CASP1 pathway_KEGG

hsa05014 Amyotrophic lateral sclerosis (ALS) http://www.kegg.jp/kegg-bin/show_pathway?hsa05014+596+834+836 49 3 0.477703556598664 6.28004535147392 0.0117634305692464 0.101592752094544 596;834;836 CASP3;BCL2;CASP1 pathway_KEGG

hsa01522 Endocrine resistance http://www.kegg.jp/kegg-bin/show_pathway?hsa01522+4193+5594+5747+596 92 4 0.89691280014443 4.4597423510467 0.0119711204697392 0.101592752094544 4193;5594;5747;596 PTK2;BCL2;MAPK1;MDM2 pathway_KEGG

hsa05222 Small cell lung cancer http://www.kegg.jp/kegg-bin/show_pathway?hsa05222+5747+596+6256+836 93 4 0.906661852319913 4.41178813221824 0.0124202059468317 0.10263012282382 5747;596;6256;836 CASP3;PTK2;BCL2;RXRA pathway_KEGG

hsa00140 Steroid hormone biosynthesis http://www.kegg.jp/kegg-bin/show_pathway?hsa00140+1544+8644 18 2 0.175482939158693 11.3971193415638 0.0129166883257392 0.103867087979301 1544;8644 AKR1C3;CYP1A2 pathway_KEGG

hsa04066 HIF-1 signaling pathway http://www.kegg.jp/kegg-bin/show_pathway?hsa04066+3939+5594+596+6774 95 4 0.926159956670879 4.31890838206628 0.0133505916041421 0.103867087979301 3939;5594;596;6774 BCL2;MAPK1;STAT3;LDHA pathway_KEGG

hsa04933 AGE-RAGE signaling pathway in diabetic complications http://www.kegg.jp/kegg-bin/show_pathway?hsa04933+5594+596+6774+836 97 4 0.945658061021845 4.22985872470409 0.0143245044535376 0.103867087979301 5594;596;6774;836 CASP3;BCL2;MAPK1;STAT3 pathway_KEGG

hsa04151 PI3K-Akt signaling pathway http://www.kegg.jp/kegg-bin/show_pathway?hsa04151+2260+2932+3320+4193+5594+5747+596+6256 333 8 3.24643437443582 2.46424201979758 0.0144135444189283 0.103867087979301 2260;2932;3320;4193;5594;5747;596;6256 GSK3B;PTK2;BCL2;MAPK1;HSP90AA1;RXRA;MDM2;FGFR1 pathway_KEGG

hsa04370 VEGF signaling pathway http://www.kegg.jp/kegg-bin/show_pathway?hsa04370+5594+5747+9261 53 3 0.516699765300596 5.80607966457023 0.0145546237932779 0.103867087979301 5594;5747;9261 PTK2;MAPKAPK2;MAPK1 pathway_KEGG

hsa05134 Legionellosis http://www.kegg.jp/kegg-bin/show_pathway?hsa05134+834+836+841 53 3 0.516699765300596 5.80607966457023 0.0145546237932779 0.103867087979301 834;836;841 CASP3;CASP8;CASP1 pathway_KEGG

hsa04976 Bile secretion http://www.kegg.jp/kegg-bin/show_pathway?hsa04976+6256+6580+760 55 3 0.536197869651562 5.5949494949495 0.0160792948935571 0.112197746590598 6256;6580;760 SLC22A1;RXRA;CA2 pathway_KEGG

hsa04924 Renin secretion http://www.kegg.jp/kegg-bin/show_pathway?hsa04924+1508+1636+5972 56 3 0.545946921827045 5.49503968253968 0.0168741686902452 0.11518454279863 1508;1636;5972 ACE;REN;CTSB pathway_KEGG

hsa04920 Adipocytokine signaling pathway http://www.kegg.jp/kegg-bin/show_pathway?hsa04920+5465+6256+6774 59 3 0.575194078353493 5.21563088512241 0.0193896173325658 0.129539145583525 5465;6256;6774 PPARA;STAT3;RXRA pathway_KEGG

hsa04668 TNF signaling pathway http://www.kegg.jp/kegg-bin/show_pathway?hsa04668+5594+836+841+9020 108 4 1.05289763495216 3.79903978052126 0.0204868806382896 0.134018344175478 5594;836;841;9020 CASP3;MAP3K14;MAPK1;CASP8 pathway_KEGG

hsa05230 Central carbon metabolism in cancer http://www.kegg.jp/kegg-bin/show_pathway?hsa05230+2260+3939+5594 61 3 0.594692182704459 5.04462659380692 0.0211760100706155 0.135699329840271 2260;3939;5594 MAPK1;FGFR1;LDHA pathway_KEGG

hsa04722 Neurotrophin signaling pathway http://www.kegg.jp/kegg-bin/show_pathway?hsa04722+2932+5594+596+9261 116 4 1.13089005235602 3.53703703703704 0.025860127983508 0.157736236653783 2932;5594;596;9261 GSK3B;MAPKAPK2;BCL2;MAPK1 pathway_KEGG

hsa05223 Non-small cell lung cancer http://www.kegg.jp/kegg-bin/show_pathway?hsa05223+5594+6256+6774 66 3 0.643437443581874 4.66245791245791 0.0260261561406683 0.157736236653783 5594;6256;6774 MAPK1;STAT3;RXRA pathway_KEGG

hsa05165 Human papillomavirus infection http://www.kegg.jp/kegg-bin/show_pathway?hsa05165+2932+4193+5594+5747+836+83933+841 302 7 2.94421375699585 2.37754476330635 0.0261219245413908 0.157736236653783 2932;4193;5594;5747;836;83933;841 CASP3;GSK3B;PTK2;MAPK1;CASP8;MDM2;HDAC10 pathway_KEGG

hsa00561 Glycerolipid metabolism http://www.kegg.jp/kegg-bin/show_pathway?hsa00561+11343+231 27 2 0.263224408738039 7.59807956104253 0.0280250554127467 0.166035233954763 11343;231 AKR1B1;MGLL pathway_KEGG

hsa04550 Signaling pathways regulating pluripotency of stem cells http://www.kegg.jp/kegg-bin/show_pathway?hsa04550+2260+2932+5594+6774 121 4 1.17963531323344 3.39087848178757 0.0296131806444817 0.169902852971132 2260;2932;5594;6774 GSK3B;MAPK1;STAT3;FGFR1 pathway_KEGG

hsa04612 Antigen processing and presentation http://www.kegg.jp/kegg-bin/show_pathway?hsa04612+1508+3320+5641 70 3 0.682433652283806 4.39603174603175 0.0303011457528134 0.169902852971132 1508;3320;5641 CTSB;HSP90AA1;LGMN pathway_KEGG

hsa04917 Prolactin signaling pathway http://www.kegg.jp/kegg-bin/show_pathway?hsa04917+2932+5594+6774 70 3 0.682433652283806 4.39603174603175 0.0303011457528134 0.169902852971132 2932;5594;6774 GSK3B;MAPK1;STAT3 pathway_KEGG

hsa05418 Fluid shear stress and atherosclerosis http://www.kegg.jp/kegg-bin/show_pathway?hsa05418+3320+5747+596+9817 123 4 1.1991334175844 3.33574224631135 0.0312007791293252 0.170125974371559 3320;5747;596;9817 KEAP1;PTK2;BCL2;HSP90AA1 pathway_KEGG

hsa05218 Melanoma http://www.kegg.jp/kegg-bin/show_pathway?hsa05218+2260+4193+5594 71 3 0.692182704459289 4.33411580594679 0.0314245430367848 0.170125974371559 2260;4193;5594 MAPK1;MDM2;FGFR1 pathway_KEGG

hsa04915 Estrogen signaling pathway http://www.kegg.jp/kegg-bin/show_pathway?hsa04915+3320+5594+5914+596 124 4 1.20888246975988 3.30884109916368 0.0320132094438322 0.170375385853616 3320;5594;5914;596 BCL2;MAPK1;HSP90AA1;RARA pathway_KEGG

hsa04723 Retrograde endocannabinoid signaling http://www.kegg.jp/kegg-bin/show_pathway?hsa04723+11343+1268+2166+5594 125 4 1.21863152193537 3.28237037037037 0.032838095002052 0.171852697177406 11343;1268;2166;5594 FAAH;CNR1;MGLL;MAPK1 pathway_KEGG

hsa05133 Pertussis http://www.kegg.jp/kegg-bin/show_pathway?hsa05133+5594+834+836 73 3 0.711680808810255 4.21537290715373 0.0337366299501105 0.173660685316962 5594;834;836 CASP3;MAPK1;CASP1 pathway_KEGG

hsa05170 Human immunodeficiency virus 1 infection http://www.kegg.jp/kegg-bin/show_pathway?hsa05170+5594+5747+596+836+841 187 5 1.82307275681531 2.74262230144583 0.0345689199980423 0.175074852893311 5594;5747;596;836;841 CASP3;PTK2;BCL2;MAPK1;CASP8 pathway_KEGG

hsa05226 Gastric cancer http://www.kegg.jp/kegg-bin/show_pathway?hsa05226+2932+5594+596+6256 138 4 1.34536920021665 2.97316156736447 0.0447030419443586 0.222805637627438 2932;5594;596;6256 GSK3B;BCL2;MAPK1;RXRA pathway_KEGG

hsa04012 ErbB signaling pathway http://www.kegg.jp/kegg-bin/show_pathway?hsa04012+2932+5594+5747 83 3 0.809171330565084 3.70749665327979 0.0465863757050972 0.228564405803133 2932;5594;5747 GSK3B;PTK2;MAPK1 pathway_KEGG

GO:1901568 fatty acid derivative metabolic process http://amigo.geneontology.org/amigo/term/GO:1901568 78 10 0.422868408082799 23.6480186480186 8.81850148459762e-12 7.05656488797501e-08 239;240;1544;2166;2171;6319;8644;9261;9536;11343 FAAH;ALOX12;MGLL;FABP5;AKR1C3;CYP1A2;PTGES;ALOX5;MAPKAPK2;SCD geneontology_Biological_Process

GO:0006690 icosanoid metabolic process http://amigo.geneontology.org/amigo/term/GO:0006690 59 9 0.319862000985707 28.1371340523883 2.08253414513138e-11 8.33221911467064e-08 239;240;1544;2166;2171;8644;9261;9536;11343 FAAH;ALOX12;MGLL;FABP5;AKR1C3;CYP1A2;PTGES;ALOX5;MAPKAPK2 geneontology_Biological_Process

GO:0006631 fatty acid metabolic process http://amigo.geneontology.org/amigo/term/GO:0006631 201 13 1.08969935929029 11.9298959746721 3.43705064409505e-11 9.16775975134954e-08 239;240;1268;1544;2166;2171;5465;5467;5468;6319;8644;9536;11343 PPARG;PPARA;FAAH;CNR1;ALOX12;MGLL;FABP5;AKR1C3;CYP1A2;PTGES;ALOX5;PPARD;SCD geneontology_Biological_Process

GO:0032787 monocarboxylic acid metabolic process http://amigo.geneontology.org/amigo/term/GO:0032787 332 15 1.79990142927551 8.33378970427163 1.40274902804549e-10 2.80619943060501e-07 239;240;1268;1544;2166;2171;3939;5465;5467;5468;6319;6774;8644;9536;11343 PPARG;PPARA;FAAH;CNR1;ALOX12;MGLL;FABP5;AKR1C3;CYP1A2;PTGES;ALOX5;STAT3;LDHA;PPARD;SCD geneontology_Biological_Process

GO:0033559 unsaturated fatty acid metabolic process http://amigo.geneontology.org/amigo/term/GO:0033559 51 8 0.276490882207984 28.9340463458111 2.33878916233721e-10 3.74299817540447e-07 239;1544;2166;2171;6319;8644;9536;11343 FAAH;ALOX12;MGLL;FABP5;AKR1C3;CYP1A2;PTGES;SCD geneontology_Biological_Process

GO:0016042 lipid catabolic process http://amigo.geneontology.org/amigo/term/GO:0016042 171 11 0.927057663873829 11.8654970760234 1.42344758202739e-09 1.76373344618064e-06 150;1268;1544;2166;2167;2171;5465;5467;7067;8644;11343 PPARA;FAAH;CNR1;ADRA2A;MGLL;FABP5;AKR1C3;CYP1A2;FABP4;PPARD;THRA geneontology_Biological_Process

GO:0032496 response to lipopolysaccharide http://amigo.geneontology.org/amigo/term/GO:0032496 273 13 1.4800394282898 8.78354978354978 1.54288104514677e-09 1.76373344618064e-06 249;834;836;841;1268;1544;5467;5594;5739;5914;5972;9261;9536 CASP3;REN;CNR1;CYP1A2;PTGES;MAPKAPK2;ALPL;MAPK1;CASP8;CASP1;PPARD;RARA;PTGIR geneontology_Biological_Process

GO:0002237 response to molecule of bacterial origin http://amigo.geneontology.org/amigo/term/GO:0002237 283 13 1.53425332676195 8.47317699967877 2.39273412194763e-09 2.39333230547811e-06 249;834;836;841;1268;1544;5467;5594;5739;5914;5972;9261;9536 CASP3;REN;CNR1;CYP1A2;PTGES;MAPKAPK2;ALPL;MAPK1;CASP8;CASP1;PPARD;RARA;PTGIR geneontology_Biological_Process

GO:0072330 monocarboxylic acid biosynthetic process http://amigo.geneontology.org/amigo/term/GO:0072330 182 11 0.986692952193199 11.1483516483516 2.75877454214424e-09 2.45285709847091e-06 239;240;1544;2171;3939;5465;6319;6774;8644;9536;11343 PPARA;ALOX12;MGLL;FABP5;AKR1C3;CYP1A2;PTGES;ALOX5;STAT3;LDHA;SCD geneontology_Biological_Process

GO:0019369 arachidonic acid metabolic process http://amigo.geneontology.org/amigo/term/GO:0019369 27 6 0.146377525874815 40.989898989899 5.20180942764625e-09 4.16248790400253e-06 239;1544;2166;8644;9536;11343 FAAH;ALOX12;MGLL;AKR1C3;CYP1A2;PTGES geneontology_Biological_Process

GO:0006633 fatty acid biosynthetic process http://amigo.geneontology.org/amigo/term/GO:0006633 86 8 0.466239526860522 17.1585623678647 1.68606468786692e-08 1.1691474782453e-05 239;240;1544;2171;6319;8644;9536;11343 ALOX12;MGLL;FABP5;AKR1C3;CYP1A2;PTGES;ALOX5;SCD geneontology_Biological_Process

GO:0001676 long-chain fatty acid metabolic process http://amigo.geneontology.org/amigo/term/GO:0001676 56 7 0.303597831444061 23.0568181818182 1.75328289664378e-08 1.1691474782453e-05 239;240;1544;2166;8644;9536;11343 FAAH;ALOX12;MGLL;AKR1C3;CYP1A2;PTGES;ALOX5 geneontology_Biological_Process

GO:0048608 reproductive structure development http://amigo.geneontology.org/amigo/term/GO:0048608 350 13 1.89748644652538 6.85116883116883 3.0721040156223e-08 1.81311670219249e-05 596;836;841;1508;5467;5468;5594;5747;5914;5950;5972;6256;8644 CASP3;REN;PPARG;AKR1C3;PTK2;CTSB;BCL2;MAPK1;CASP8;RXRA;PPARD;RBP4;RARA geneontology_Biological_Process

GO:0019216 regulation of lipid metabolic process http://amigo.geneontology.org/amigo/term/GO:0019216 288 12 1.56136027599803 7.68560606060606 3.21370667855092e-08 1.81311670219249e-05 150;1268;2171;3357;5465;5467;5468;5747;6256;6319;7067;8644 PPARG;PPARA;CNR1;ADRA2A;FABP5;AKR1C3;PTK2;RXRA;HTR2B;PPARD;SCD;THRA geneontology_Biological_Process

GO:0061458 reproductive system development http://amigo.geneontology.org/amigo/term/GO:0061458 353 13 1.91375061606703 6.79294360030904 3.39874413057828e-08 1.81311670219249e-05 596;836;841;1508;5467;5468;5594;5747;5914;5950;5972;6256;8644 CASP3;REN;PPARG;AKR1C3;PTK2;CTSB;BCL2;MAPK1;CASP8;RXRA;PPARD;RBP4;RARA geneontology_Biological_Process

GO:0010038 response to metal ion http://amigo.geneontology.org/amigo/term/GO:0010038 294 12 1.59388861508132 7.52875695732839 4.0376818999377e-08 2.01934566020634e-05 596;760;836;841;1544;2167;4193;5594;5641;8644;9536;23621 CASP3;BACE1;AKR1C3;CYP1A2;PTGES;BCL2;MAPK1;CASP8;CA2;MDM2;LGMN;FABP4 geneontology_Biological_Process

GO:0009617 response to bacterium http://amigo.geneontology.org/amigo/term/GO:0009617 436 14 2.3637259733859 5.92285237698082 5.14997613443313e-08 2.42412406045494e-05 249;834;836;841;1268;1544;2167;5467;5594;5739;5914;5972;9261;9536 CASP3;REN;CNR1;CYP1A2;PTGES;MAPKAPK2;ALPL;MAPK1;CASP8;CASP1;FABP4;PPARD;RARA;PTGIR geneontology_Biological_Process

GO:0010035 response to inorganic substance http://amigo.geneontology.org/amigo/term/GO:0010035 453 14 2.45588960078857 5.70058197872767 8.2983685922855e-08 3.68908585974825e-05 231;596;760;836;841;1544;2167;3939;4193;5594;5641;8644;9536;23621 CASP3;AKR1B1;BACE1;AKR1C3;CYP1A2;PTGES;BCL2;MAPK1;CASP8;CA2;MDM2;LDHA;LGMN;FABP4 geneontology_Biological_Process

GO:0016053 organic acid biosynthetic process http://amigo.geneontology.org/amigo/term/GO:0016053 256 11 1.38787580088714 7.92578125 9.54987754431258e-08 3.82090600547946e-05 239;240;1544;2171;3939;5465;6319;6774;8644;9536;11343 PPARA;ALOX12;MGLL;FABP5;AKR1C3;CYP1A2;PTGES;ALOX5;STAT3;LDHA;SCD geneontology_Biological_Process

GO:0046394 carboxylic acid biosynthetic process http://amigo.geneontology.org/amigo/term/GO:0046394 256 11 1.38787580088714 7.92578125 9.54987754431258e-08 3.82090600547946e-05 239;240;1544;2171;3939;5465;6319;6774;8644;9536;11343 PPARA;ALOX12;MGLL;FABP5;AKR1C3;CYP1A2;PTGES;ALOX5;STAT3;LDHA;SCD geneontology_Biological_Process

GO:0044283 small molecule biosynthetic process http://amigo.geneontology.org/amigo/term/GO:0044283 461 14 2.49926071956629 5.60165647801223 1.03130503914528e-07 3.92976329678121e-05 231;239;240;1544;2171;3939;5141;5465;5950;6319;6774;8644;9536;11343 AKR1B1;PPARA;ALOX12;PDE4A;MGLL;FABP5;AKR1C3;CYP1A2;PTGES;ALOX5;STAT3;LDHA;RBP4;SCD geneontology_Biological_Process

GO:0008015 blood circulation http://amigo.geneontology.org/amigo/term/GO:0008015 405 13 2.19566288812223 5.92076318742985 1.69975270303269e-07 5.88162019130359e-05 136;140;150;152;1268;1636;3357;4193;5465;5467;5468;5972;7067 ACE;REN;PPARG;PPARA;CNR1;ADORA2B;ADORA3;ADRA2A;ADRA2C;MDM2;HTR2B;PPARD;THRA geneontology_Biological_Process

GO:1901570 fatty acid derivative biosynthetic process http://amigo.geneontology.org/amigo/term/GO:1901570 47 6 0.254805322819123 23.5473887814313 1.7366042748268e-07 5.88162019130359e-05 239;240;2171;6319;8644;9536 ALOX12;FABP5;AKR1C3;PTGES;ALOX5;SCD geneontology_Biological_Process

GO:0044242 cellular lipid catabolic process http://amigo.geneontology.org/amigo/term/GO:0044242 117 8 0.634302612124199 12.6122766122766 1.90572761127328e-07 5.88162019130359e-05 1268;2166;2167;2171;5465;5467;8644;11343 PPARA;FAAH;CNR1;MGLL;FABP5;AKR1C3;FABP4;PPARD geneontology_Biological_Process

GO:0033002 muscle cell proliferation http://amigo.geneontology.org/amigo/term/GO:0033002 163 9 0.883686545096106 10.1846068042387 1.93182525864977e-07 5.88162019130359e-05 231;2260;4193;5467;5468;5739;5950;6256;6774 AKR1B1;PPARG;STAT3;RXRA;MDM2;FGFR1;PPARD;RBP4;PTGIR geneontology_Biological_Process

GO:0048545 response to steroid hormone http://amigo.geneontology.org/amigo/term/GO:0048545 341 12 1.84869393790044 6.4910690482538 2.04662773839459e-07 5.88162019130359e-05 249;596;760;836;4193;5465;5467;5468;5914;6256;7067;8644 CASP3;PPARG;PPARA;AKR1C3;ALPL;BCL2;RXRA;CA2;MDM2;PPARD;RARA;THRA geneontology_Biological_Process

GO:0046677 response to antibiotic http://amigo.geneontology.org/amigo/term/GO:0046677 276 11 1.49630359783144 7.35144927536232 2.04702283790326e-07 5.88162019130359e-05 249;596;836;841;1268;3320;3939;4193;5914;5950;6774 CASP3;CNR1;ALPL;BCL2;STAT3;HSP90AA1;CASP8;MDM2;LDHA;RBP4;RARA geneontology_Biological_Process

GO:0008610 lipid biosynthetic process http://amigo.geneontology.org/amigo/term/GO:0008610 412 13 2.23361261705274 5.82016769638129 2.07266498408742e-07 5.88162019130359e-05 43;231;239;240;1544;2171;2260;3357;5467;6319;8644;9536;11343 AKR1B1;ACHE;ALOX12;MGLL;FABP5;AKR1C3;CYP1A2;PTGES;ALOX5;FGFR1;HTR2B;PPARD;SCD geneontology_Biological_Process

GO:0003013 circulatory system process http://amigo.geneontology.org/amigo/term/GO:0003013 413 13 2.23903400689995 5.80607528065155 2.13155443073987e-07 5.88162019130359e-05 136;140;150;152;1268;1636;3357;4193;5465;5467;5468;5972;7067 ACE;REN;PPARG;PPARA;CNR1;ADORA2B;ADORA3;ADRA2A;ADRA2C;MDM2;HTR2B;PPARD;THRA geneontology_Biological_Process

GO:0003006 developmental process involved in reproduction http://amigo.geneontology.org/amigo/term/GO:0003006 491 14 2.66190241498275 5.25939640807258 2.24402897042175e-07 5.98557327377162e-05 249;596;836;841;1508;5467;5468;5594;5747;5914;5950;5972;6256;8644 CASP3;REN;PPARG;AKR1C3;PTK2;ALPL;CTSB;BCL2;MAPK1;CASP8;RXRA;PPARD;RBP4;RARA geneontology_Biological_Process

GO:0006636 unsaturated fatty acid biosynthetic process http://amigo.geneontology.org/amigo/term/GO:0006636 26 5 0.1409561360276 35.472027972028 2.34599558446291e-07 6.05569569899104e-05 239;2171;6319;8644;9536 ALOX12;FABP5;AKR1C3;PTGES;SCD geneontology_Biological_Process

GO:0045862 positive regulation of proteolysis http://amigo.geneontology.org/amigo/term/GO:0045862 289 11 1.56678166584524 7.02076124567474 3.25163793224448e-07 8.13112710431885e-05 150;834;836;841;2932;4193;5468;5641;5747;6774;9817 CASP3;PPARG;GSK3B;ADRA2A;KEAP1;PTK2;STAT3;CASP8;CASP1;MDM2;LGMN geneontology_Biological_Process

GO:0048871 multicellular organismal homeostasis http://amigo.geneontology.org/amigo/term/GO:0048871 363 12 1.96796451453918 6.0976709241172 4.01534917204849e-07 9.7366133559794e-05 43;231;239;596;760;1268;2167;2171;5950;6319;6774;7067 AKR1B1;ACHE;CNR1;ALOX12;FABP5;BCL2;STAT3;CA2;FABP4;RBP4;SCD;THRA geneontology_Biological_Process

GO:0030522 intracellular receptor signaling pathway http://amigo.geneontology.org/amigo/term/GO:0030522 236 10 1.27944800394283 7.81587057010786 4.47967417938067e-07 0.000105430449362953 841;2171;5465;5467;5468;5914;6256;6774;7067;8644 PPARG;PPARA;FABP5;AKR1C3;STAT3;CASP8;RXRA;PPARD;RARA;THRA geneontology_Biological_Process

GO:0010817 regulation of hormone levels http://amigo.geneontology.org/amigo/term/GO:0010817 378 12 2.04928536224741 5.85569985569986 6.19108002641866e-07 0.000141545778204006 43;150;152;231;1268;1636;2260;5467;5950;5972;6580;8644 SLC22A1;ACE;REN;AKR1B1;ACHE;CNR1;ADRA2A;ADRA2C;AKR1C3;FGFR1;PPARD;RBP4 geneontology_Biological_Process

GO:0044057 regulation of system process http://amigo.geneontology.org/amigo/term/GO:0044057 457 13 2.47757516017743 5.24706584444002 6.79816169912861e-07 0.000151108027545631 136;140;150;152;1636;2171;2260;4193;5465;5747;5972;7067;11343 ACE;REN;PPARA;ADORA2B;ADORA3;ADRA2A;ADRA2C;MGLL;FABP5;PTK2;MDM2;FGFR1;THRA geneontology_Biological_Process

GO:0046456 icosanoid biosynthetic process http://amigo.geneontology.org/amigo/term/GO:0046456 32 5 0.173484475110892 28.8210227272727 7.006810096577e-07 0.000151536471331917 239;240;2171;8644;9536 ALOX12;FABP5;AKR1C3;PTGES;ALOX5 geneontology_Biological_Process

GO:0071407 cellular response to organic cyclic compound http://amigo.geneontology.org/amigo/term/GO:0071407 465 13 2.52094627895515 5.15679374389052 8.2763840247857e-07 0.000174283223595619 249;836;841;4193;5465;5467;5468;5594;5914;6256;6774;7067;8644 CASP3;PPARG;PPARA;AKR1C3;ALPL;MAPK1;STAT3;CASP8;RXRA;MDM2;PPARD;RARA;THRA geneontology_Biological_Process

GO:1901615 organic hydroxy compound metabolic process http://amigo.geneontology.org/amigo/term/GO:1901615 324 11 1.75653031049778 6.26234567901234 1.01309326860921e-06 0.000205709132484877 231;239;596;2260;3939;5467;5641;5950;6256;6319;8644 AKR1B1;ALOX12;AKR1C3;BCL2;RXRA;FGFR1;LDHA;LGMN;PPARD;RBP4;SCD geneontology_Biological_Process

GO:0043627 response to estrogen http://amigo.geneontology.org/amigo/term/GO:0043627 63 6 0.341547560374569 17.5670995670996 1.02828859027682e-06 0.000205709132484877 760;3939;4193;5468;5594;5914 PPARG;MAPK1;CA2;MDM2;LDHA;RARA geneontology_Biological_Process

GO:0016485 protein processing http://amigo.geneontology.org/amigo/term/GO:0016485 204 9 1.10596352883194 8.13770053475936 1.2864686145253e-06 0.000245837554623321 834;836;841;1636;4193;5328;5641;5972;23385 CASP3;ACE;REN;NCSTN;CASP8;CASP1;MDM2;PLAU;LGMN geneontology_Biological_Process

GO:0050435 amyloid-beta metabolic process http://amigo.geneontology.org/amigo/term/GO:0050435 36 5 0.195170034499754 25.6186868686869 1.29032458062728e-06 0.000245837554623321 836;1636;5972;23385;23621 CASP3;ACE;REN;BACE1;NCSTN geneontology_Biological_Process

GO:0009991 response to extracellular stimulus http://amigo.geneontology.org/amigo/term/GO:0009991 414 12 2.24445539674717 5.34650856389987 1.62055411623285e-06 0.000301573814839425 136;249;596;1268;3939;4193;5465;5467;5468;5594;5914;8644 PPARG;PPARA;CNR1;ADORA2B;AKR1C3;ALPL;BCL2;MAPK1;MDM2;LDHA;PPARD;RARA geneontology_Biological_Process

GO:0007565 female pregnancy http://amigo.geneontology.org/amigo/term/GO:0007565 157 8 0.851158206012814 9.39895773016792 1.80811023553495e-06 0.000328829502380698 152;231;596;1268;1508;5467;5914;6256 AKR1B1;CNR1;ADRA2C;CTSB;BCL2;RXRA;PPARD;RARA geneontology_Biological_Process

GO:0099177 regulation of trans-synaptic signaling http://amigo.geneontology.org/amigo/term/GO:0099177 352 11 1.90832922621981 5.76420454545454 2.28116008371693e-06 0.000405640955331174 43;136;760;1268;2171;2932;5594;5641;5914;6774;23621 BACE1;ACHE;GSK3B;CNR1;ADORA2B;FABP5;MAPK1;STAT3;CA2;LGMN;RARA geneontology_Biological_Process

GO:0009266 response to temperature stimulus http://amigo.geneontology.org/amigo/term/GO:0009266 163 8 0.883686545096106 9.05298382598996 2.39693256975926e-06 0.000416962052678556 841;2932;3320;3357;5468;5594;7067;9261 PPARG;GSK3B;MAPKAPK2;MAPK1;HSP90AA1;CASP8;HTR2B;THRA geneontology_Biological_Process

GO:0007507 heart development http://amigo.geneontology.org/amigo/term/GO:0007507 431 12 2.33661902414983 5.13562539548618 2.46832328776314e-06 0.000420245169120865 836;2260;3357;4193;5465;5467;5468;5594;5747;5914;5950;6256 CASP3;PPARG;PPARA;PTK2;MAPK1;RXRA;MDM2;FGFR1;HTR2B;PPARD;RBP4;RARA geneontology_Biological_Process

GO:0010887 negative regulation of cholesterol storage http://amigo.geneontology.org/amigo/term/GO:0010887 6 3 0.0325283390832923 92.2272727272727 2.98136903231772e-06 0.000497019062429299 5465;5467;5468 PPARG;PPARA;PPARD geneontology_Biological_Process

GO:0035094 response to nicotine http://amigo.geneontology.org/amigo/term/GO:0035094 43 5 0.233119763430261 21.4482029598309 3.20117369745354e-06 0.000522771263816801 596;836;1268;5465;5594 CASP3;PPARA;CNR1;BCL2;MAPK1 geneontology_Biological_Process

GO:0001890 placenta development http://amigo.geneontology.org/amigo/term/GO:0001890 120 7 0.650566781665845 10.7598484848485 3.44652011785573e-06 0.000551581079661632 841;1508;5467;5468;5594;5747;6256 PPARG;PTK2;CTSB;MAPK1;CASP8;RXRA;PPARD geneontology_Biological_Process

GO:0019233 sensory perception of pain http://amigo.geneontology.org/amigo/term/GO:0019233 81 6 0.439132577624446 13.6632996632997 4.55806260524216e-06 0.000715168960140153 152;1268;2171;5594;11343;23621 BACE1;CNR1;ADRA2C;MGLL;FABP5;MAPK1 geneontology_Biological_Process

GO:0044706 multi-multicellular organism process http://amigo.geneontology.org/amigo/term/GO:0044706 180 8 0.975850172498768 8.1979797979798 5.02270557289908e-06 0.000772917115275739 152;231;596;1268;1508;5467;5914;6256 AKR1B1;CNR1;ADRA2C;CTSB;BCL2;RXRA;PPARD;RARA geneontology_Biological_Process

GO:0048241 epinephrine transport http://amigo.geneontology.org/amigo/term/GO:0048241 7 3 0.0379497289305076 79.0519480519481 5.19734362303126e-06 0.000784700823990493 150;152;6580 SLC22A1;ADRA2A;ADRA2C geneontology_Biological_Process

GO:0015850 organic hydroxy compound transport http://amigo.geneontology.org/amigo/term/GO:0015850 182 8 0.986692952193199 8.10789210789211 5.45110758265466e-06 0.000807773386600048 150;152;1268;5468;5950;5972;6256;6580 SLC22A1;REN;PPARG;CNR1;ADRA2A;ADRA2C;RXRA;RBP4 geneontology_Biological_Process

GO:0007584 response to nutrient http://amigo.geneontology.org/amigo/term/GO:0007584 183 8 0.992114342040414 8.0635866865375 5.67667365491964e-06 0.000825904410666672 249;1268;3939;4193;5467;5468;5914;8644 PPARG;CNR1;AKR1C3;ALPL;MDM2;LDHA;PPARD;RARA geneontology_Biological_Process

GO:0031667 response to nutrient levels http://amigo.geneontology.org/amigo/term/GO:0031667 388 11 2.10349926071957 5.22938144329897 5.8351483438468e-06 0.00083380101870468 249;596;1268;3939;4193;5465;5467;5468;5594;5914;8644 PPARG;PPARA;CNR1;AKR1C3;ALPL;BCL2;MAPK1;MDM2;LDHA;PPARD;RARA geneontology_Biological_Process

GO:0051604 protein maturation http://amigo.geneontology.org/amigo/term/GO:0051604 247 9 1.3390832922622 6.72101582627898 6.21612626228174e-06 0.000872656883346991 834;836;841;1636;4193;5328;5641;5972;23385 CASP3;ACE;REN;NCSTN;CASP8;CASP1;MDM2;PLAU;LGMN geneontology_Biological_Process

GO:0042445 hormone metabolic process http://amigo.geneontology.org/amigo/term/GO:0042445 135 7 0.731887629374076 9.56430976430976 7.53513419771146e-06 0.00102522797217847 43;231;1636;2260;5950;5972;8644 ACE;REN;AKR1B1;ACHE;AKR1C3;FGFR1;RBP4 geneontology_Biological_Process

GO:0060135 maternal process involved in female pregnancy http://amigo.geneontology.org/amigo/term/GO:0060135 51 5 0.276490882207984 18.0837789661319 7.55916650319044e-06 0.00102522797217847 231;1268;1508;5467;6256 AKR1B1;CNR1;CTSB;RXRA;PPARD geneontology_Biological_Process

GO:0014855 striated muscle cell proliferation http://amigo.geneontology.org/amigo/term/GO:0014855 52 5 0.2819122720552 17.736013986014 8.32902477554409e-06 0.0011108142708984 2260;5467;5950;6256;6774 STAT3;RXRA;FGFR1;PPARD;RBP4 geneontology_Biological_Process

GO:0001659 temperature homeostasis http://amigo.geneontology.org/amigo/term/GO:0001659 138 7 0.748151798915722 9.35638998682477 8.70972089561661e-06 0.00114254404273318 43;1268;2167;2171;6319;6774;7067 ACHE;CNR1;FABP5;STAT3;FABP4;SCD;THRA geneontology_Biological_Process

GO:0009636 response to toxic substance http://amigo.geneontology.org/amigo/term/GO:0009636 408 11 2.21192705766387 4.97303921568627 9.41438210599888e-06 0.00121506267116456 596;836;841;1268;3939;4193;5465;5594;5914;5950;6774 CASP3;PPARA;CNR1;BCL2;MAPK1;STAT3;CASP8;MDM2;LDHA;RBP4;RARA geneontology_Biological_Process

GO:0008202 steroid metabolic process http://amigo.geneontology.org/amigo/term/GO:0008202 197 8 1.06801379990143 7.49053991693586 9.76800984042736e-06 0.00124069229750952 231;1544;2260;5467;5641;6256;6319;8644 AKR1B1;AKR1C3;CYP1A2;RXRA;FGFR1;LGMN;PPARD;SCD geneontology_Biological_Process

GO:0035265 organ growth http://amigo.geneontology.org/amigo/term/GO:0035265 141 7 0.764415968457368 9.15731785944552 1.00326267081785e-05 0.00125439185810694 596;2260;5465;5747;5914;5950;6256 PPARA;PTK2;BCL2;RXRA;FGFR1;RBP4;RARA geneontology_Biological_Process

GO:0035902 response to immobilization stress http://amigo.geneontology.org/amigo/term/GO:0035902 26 4 0.1409561360276 28.3776223776224 1.05789659659017e-05 0.00130235208706377 1544;4193;5468;5972 REN;PPARG;CYP1A2;MDM2 geneontology_Biological_Process

GO:0001101 response to acid chemical http://amigo.geneontology.org/amigo/term/GO:0001101 268 9 1.45293247905372 6.19436906377205 1.201700497655e-05 0.00141539595754103 231;836;5468;5914;5950;6256;6319;8644;9536 CASP3;AKR1B1;PPARG;AKR1C3;PTGES;RXRA;RBP4;SCD;RARA geneontology_Biological_Process

GO:0071496 cellular response to external stimulus http://amigo.geneontology.org/amigo/term/GO:0071496 268 9 1.45293247905372 6.19436906377205 1.201700497655e-05 0.00141539595754103 136;596;834;841;4193;5468;5594;8644;9020 PPARG;ADORA2B;MAP3K14;AKR1C3;BCL2;MAPK1;CASP8;CASP1;MDM2 geneontology_Biological_Process

GO:0014910 regulation of smooth muscle cell migration http://amigo.geneontology.org/amigo/term/GO:0014910 56 5 0.303597831444061 16.4691558441558 1.20419479241463e-05 0.00141539595754103 596;1636;4193;5328;5467 ACE;BCL2;MDM2;PLAU;PPARD geneontology_Biological_Process

GO:0048638 regulation of developmental growth http://amigo.geneontology.org/amigo/term/GO:0048638 269 9 1.45835386890094 6.17134166948293 1.23816192236781e-05 0.00141539595754103 596;2260;2932;5465;5467;5641;5747;5950;6774 PPARA;GSK3B;PTK2;BCL2;STAT3;FGFR1;LGMN;PPARD;RBP4 geneontology_Biological_Process

GO:0062012 regulation of small molecule metabolic process http://amigo.geneontology.org/amigo/term/GO:0062012 269 9 1.45835386890094 6.17134166948293 1.23816192236781e-05 0.00141539595754103 136;1268;2171;2932;5465;5468;6319;6774;8644 PPARG;PPARA;GSK3B;CNR1;ADORA2B;FABP5;AKR1C3;STAT3;SCD geneontology_Biological_Process

GO:0001655 urogenital system development http://amigo.geneontology.org/amigo/term/GO:0001655 271 9 1.46919664859537 6.12579671251258 1.31393243698597e-05 0.00148085737475517 231;596;760;1636;2260;5914;5950;5972;6256 ACE;REN;AKR1B1;BCL2;RXRA;CA2;FGFR1;RBP4;RARA geneontology_Biological_Process

GO:0090407 organophosphate biosynthetic process http://amigo.geneontology.org/amigo/term/GO:0090407 429 11 2.3257762444554 4.72960372960373 1.51083157448051e-05 0.00167912142486014 43;136;2171;2260;3357;3939;5465;5467;5594;6319;6774 PPARA;ACHE;ADORA2B;FABP5;MAPK1;STAT3;FGFR1;LDHA;HTR2B;PPARD;SCD geneontology_Biological_Process

GO:1904951 positive regulation of establishment of protein localization http://amigo.geneontology.org/amigo/term/GO:1904951 350 10 1.89748644652538 5.27012987012987 1.53861913689823e-05 0.0016865794977342 43;596;834;841;2932;3357;4193;5467;5594;5950 ACHE;GSK3B;BCL2;MAPK1;CASP8;CASP1;MDM2;HTR2B;PPARD;RBP4 geneontology_Biological_Process

GO:0050804 modulation of chemical synaptic transmission http://amigo.geneontology.org/amigo/term/GO:0050804 351 10 1.9029078363726 5.25511525511525 1.57723293341405e-05 0.00170554296394314 43;136;760;1268;2932;5594;5641;5914;6774;23621 BACE1;ACHE;GSK3B;CNR1;ADORA2B;MAPK1;STAT3;CA2;LGMN;RARA geneontology_Biological_Process

GO:0006869 lipid transport http://amigo.geneontology.org/amigo/term/GO:0006869 215 8 1.16559881715131 6.86342494714588 1.84667415339401e-05 0.00197027821006118 1636;2167;5465;5467;5468;5950;5972;6256 ACE;REN;PPARG;PPARA;RXRA;FABP4;PPARD;RBP4 geneontology_Biological_Process

GO:0015718 monocarboxylic acid transport http://amigo.geneontology.org/amigo/term/GO:0015718 104 6 0.563824544110399 10.6416083916084 1.93713068199486e-05 0.00203959469964774 1636;2167;5465;5467;5468;6256 ACE;PPARG;PPARA;RXRA;FABP4;PPARD geneontology_Biological_Process

GO:0014909 smooth muscle cell migration http://amigo.geneontology.org/amigo/term/GO:0014909 62 5 0.336126170527353 14.875366568915 1.99029579239784e-05 0.00206835674425553 596;1636;4193;5328;5467 ACE;BCL2;MDM2;PLAU;PPARD geneontology_Biological_Process

GO:0045860 positive regulation of protein kinase activity http://amigo.geneontology.org/amigo/term/GO:0045860 445 11 2.41251848201084 4.55955056179775 2.12698452555227e-05 0.00218206797095759 136;150;152;1636;2260;3320;3357;5594;5747;9020;9261 ACE;ADORA2B;MAP3K14;ADRA2A;ADRA2C;PTK2;MAPKAPK2;MAPK1;HSP90AA1;FGFR1;HTR2B geneontology_Biological_Process

GO:0035296 regulation of tube diameter http://amigo.geneontology.org/amigo/term/GO:0035296 110 6 0.596352883193692 10.0611570247934 2.66711980344558e-05 0.00266778658339644 136;150;152;1636;3357;5467 ACE;ADORA2B;ADRA2A;ADRA2C;HTR2B;PPARD geneontology_Biological_Process

GO:0097746 regulation of blood vessel diameter http://amigo.geneontology.org/amigo/term/GO:0097746 110 6 0.596352883193692 10.0611570247934 2.66711980344558e-05 0.00266778658339644 136;150;152;1636;3357;5467 ACE;ADORA2B;ADRA2A;ADRA2C;HTR2B;PPARD geneontology_Biological_Process

GO:0019395 fatty acid oxidation http://amigo.geneontology.org/amigo/term/GO:0019395 66 5 0.357811729916215 13.9738292011019 2.70393979384664e-05 0.00267122546053838 239;1268;5465;5467;5468 PPARG;PPARA;CNR1;ALOX12;PPARD geneontology_Biological_Process

GO:0023061 signal release http://amigo.geneontology.org/amigo/term/GO:0023061 380 10 2.06012814194184 4.85406698564593 3.12676919014265e-05 0.00297988522607417 136;150;152;1268;2260;2932;5467;5950;5972;23621 REN;BACE1;GSK3B;CNR1;ADORA2B;ADRA2A;ADRA2C;FGFR1;PPARD;RBP4 geneontology_Biological_Process

GO:0034440 lipid oxidation http://amigo.geneontology.org/amigo/term/GO:0034440 68 5 0.368654509610646 13.5628342245989 3.12809746301212e-05 0.00297988522607417 239;1268;5465;5467;5468 PPARG;PPARA;CNR1;ALOX12;PPARD geneontology_Biological_Process

GO:0015908 fatty acid transport http://amigo.geneontology.org/amigo/term/GO:0015908 68 5 0.368654509610646 13.5628342245989 3.12809746301212e-05 0.00297988522607417 1636;2167;5465;5467;5468 ACE;PPARG;PPARA;FABP4;PPARD geneontology_Biological_Process

GO:0009895 negative regulation of catabolic process http://amigo.geneontology.org/amigo/term/GO:0009895 232 8 1.25776244455397 6.36050156739812 3.19393578069871e-05 0.00300680871966483 150;596;1268;3357;5465;5747;6774;9261 PPARA;CNR1;ADRA2A;PTK2;MAPKAPK2;BCL2;STAT3;HTR2B geneontology_Biological_Process

GO:1901653 cellular response to peptide http://amigo.geneontology.org/amigo/term/GO:1901653 306 9 1.65894529324791 5.42513368983957 3.4443378304605e-05 0.00318056509896116 231;760;2932;4193;5468;5641;5747;6774;23621 AKR1B1;PPARG;BACE1;GSK3B;PTK2;STAT3;CA2;MDM2;LGMN geneontology_Biological_Process

GO:0071417 cellular response to organonitrogen compound http://amigo.geneontology.org/amigo/term/GO:0071417 469 11 2.54263183834401 4.32622601279318 3.45800004510899e-05 0.00318056509896116 231;760;836;2932;4193;5468;5594;5641;5747;6774;23621 CASP3;AKR1B1;PPARG;BACE1;GSK3B;PTK2;MAPK1;STAT3;CA2;MDM2;LGMN geneontology_Biological_Process

GO:0009409 response to cold http://amigo.geneontology.org/amigo/term/GO:0009409 35 4 0.189748644652538 21.0805194805195 3.57339343883822e-05 0.00320439847749488 841;3320;5468;7067 PPARG;HSP90AA1;CASP8;THRA geneontology_Biological_Process

GO:0035150 regulation of tube size http://amigo.geneontology.org/amigo/term/GO:0035150 116 6 0.628881222276984 9.54075235109718 3.6040472753629e-05 0.00320439847749488 136;150;152;1636;3357;5467 ACE;ADORA2B;ADRA2A;ADRA2C;HTR2B;PPARD geneontology_Biological_Process

GO:0050880 regulation of blood vessel size http://amigo.geneontology.org/amigo/term/GO:0050880 116 6 0.628881222276984 9.54075235109718 3.6040472753629e-05 0.00320439847749488 136;150;152;1636;3357;5467 ACE;ADORA2B;ADRA2A;ADRA2C;HTR2B;PPARD geneontology_Biological_Process

GO:0010876 lipid localization http://amigo.geneontology.org/amigo/term/GO:0010876 239 8 1.29571217348448 6.17421072651198 3.94938070422679e-05 0.00342356398061405 1636;2167;5465;5467;5468;5950;5972;6256 ACE;REN;PPARG;PPARA;RXRA;FABP4;PPARD;RBP4 geneontology_Biological_Process

GO:0072001 renal system development http://amigo.geneontology.org/amigo/term/GO:0072001 239 8 1.29571217348448 6.17421072651198 3.94938070422679e-05 0.00342356398061405 231;596;760;1636;2260;5914;5950;5972 ACE;REN;AKR1B1;BCL2;CA2;FGFR1;RBP4;RARA geneontology_Biological_Process

GO:0019748 secondary metabolic process http://amigo.geneontology.org/amigo/term/GO:0019748 36 4 0.195170034499754 20.4949494949495 4.00393856143655e-05 0.00342356398061405 231;596;1544;8644 AKR1B1;AKR1C3;CYP1A2;BCL2 geneontology_Biological_Process

GO:0033674 positive regulation of kinase activity http://amigo.geneontology.org/amigo/term/GO:0033674 477 11 2.58600295712173 4.25366876310273 4.0388473774744e-05 0.00342356398061405 136;150;152;1636;2260;3320;3357;5594;5747;9020;9261 ACE;ADORA2B;MAP3K14;ADRA2A;ADRA2C;PTK2;MAPKAPK2;MAPK1;HSP90AA1;FGFR1;HTR2B geneontology_Biological_Process

GO:0030855 epithelial cell differentiation http://amigo.geneontology.org/amigo/term/GO:0030855 478 11 2.59142434696895 4.24476987447699 4.11707330481015e-05 0.00342356398061405 836;1508;2260;2932;5468;5914;6098;6256;7067;8644;9817 CASP3;PPARG;GSK3B;AKR1C3;KEAP1;CTSB;RXRA;FGFR1;RARA;THRA;ROS1 geneontology_Biological_Process

GO:0010878 cholesterol storage http://amigo.geneontology.org/amigo/term/GO:0010878 13 3 0.0704780680137999 42.5664335664336 4.15003381804002e-05 0.00342356398061405 5465;5467;5468 PPARG;PPARA;PPARD geneontology_Biological_Process

GO:0010885 regulation of cholesterol storage http://amigo.geneontology.org/amigo/term/GO:0010885 13 3 0.0704780680137999 42.5664335664336 4.15003381804002e-05 0.00342356398061405 5465;5467;5468 PPARG;PPARA;PPARD geneontology_Biological_Process

GO:0014812 muscle cell migration http://amigo.geneontology.org/amigo/term/GO:0014812 74 5 0.401182848693938 12.463144963145 4.71628374112409e-05 0.0038120911612601 596;1636;4193;5328;5467 ACE;BCL2;MDM2;PLAU;PPARD geneontology_Biological_Process

GO:0006766 vitamin metabolic process http://amigo.geneontology.org/amigo/term/GO:0006766 74 5 0.401182848693938 12.463144963145 4.71628374112409e-05 0.0038120911612601 2260;5467;5641;6256;8644 AKR1C3;RXRA;FGFR1;LGMN;PPARD geneontology_Biological_Process

GO:0033273 response to vitamin http://amigo.geneontology.org/amigo/term/GO:0033273 75 5 0.406604238541153 12.2969696969697 5.03245164880095e-05 0.00402696780937052 249;4193;5467;5468;5914 PPARG;ALPL;MDM2;PPARD;RARA geneontology_Biological_Process

GO:0006820 anion transport http://amigo.geneontology.org/amigo/term/GO:0006820 322 9 1.74568753080335 5.15556182947487 5.12965975687507e-05 0.00406411261133805 760;1636;2167;2260;5465;5467;5468;6098;6256 ACE;PPARG;PPARA;RXRA;CA2;FGFR1;FABP4;PPARD;ROS1 geneontology_Biological_Process

GO:0033604 negative regulation of catecholamine secretion http://amigo.geneontology.org/amigo/term/GO:0033604 14 3 0.0758994578610153 39.525974025974 5.2615869287731e-05 0.00412776652980807 150;152;1268 CNR1;ADRA2A;ADRA2C geneontology_Biological_Process

GO:0120162 positive regulation of cold-induced thermogenesis http://amigo.geneontology.org/amigo/term/GO:0120162 76 5 0.412025628388369 12.1351674641148 5.36477354008413e-05 0.00416785610366536 43;2167;2171;6319;7067 ACHE;FABP5;FABP4;SCD;THRA geneontology_Biological_Process

GO:1901214 regulation of neuron death http://amigo.geneontology.org/amigo/term/GO:1901214 250 8 1.35534746180384 5.90254545454545 5.43595870727831e-05 0.00418255207458087 596;836;841;2932;5465;5641;6774;23621 CASP3;BACE1;PPARA;GSK3B;BCL2;STAT3;CASP8;LGMN geneontology_Biological_Process

GO:0002066 columnar/cuboidal epithelial cell development http://amigo.geneontology.org/amigo/term/GO:0002066 39 4 0.2114342040414 18.9184149184149 5.52382037393162e-05 0.00420967720306675 2260;2932;5914;6098 GSK3B;FGFR1;RARA;ROS1 geneontology_Biological_Process

GO:0032526 response to retinoic acid http://amigo.geneontology.org/amigo/term/GO:0032526 78 5 0.422868408082799 11.8240093240093 6.0801553732448e-05 0.00457158191704275 5468;5914;5950;6256;9536 PPARG;PTGES;RXRA;RBP4;RARA geneontology_Biological_Process

GO:0050994 regulation of lipid catabolic process http://amigo.geneontology.org/amigo/term/GO:0050994 40 4 0.216855593888615 18.4454545454545 6.11296257340133e-05 0.00457158191704275 150;1268;5465;7067 PPARA;CNR1;ADRA2A;THRA geneontology_Biological_Process

GO:0042180 cellular ketone metabolic process http://amigo.geneontology.org/amigo/term/GO:0042180 128 6 0.693937900443568 8.64630681818182 6.26776966907716e-05 0.00464395304555143 231;1268;2171;5465;5468;8644 AKR1B1;PPARG;PPARA;CNR1;FABP5;AKR1C3 geneontology_Biological_Process

GO:0035357 peroxisome proliferator activated receptor signaling pathway http://amigo.geneontology.org/amigo/term/GO:0035357 15 3 0.0813208477082307 36.8909090909091 6.55174266566139e-05 0.00480982062482774 2171;5468;6256 PPARG;FABP5;RXRA geneontology_Biological_Process

GO:0008544 epidermis development http://amigo.geneontology.org/amigo/term/GO:0008544 259 8 1.40413997042878 5.6974376974377 6.97610153230777e-05 0.0050747967692297 596;836;2171;2260;5465;5467;8644;9817 CASP3;PPARA;FABP5;AKR1C3;KEAP1;BCL2;FGFR1;PPARD geneontology_Biological_Process

GO:1901652 response to peptide http://amigo.geneontology.org/amigo/term/GO:1901652 420 10 2.27698373583046 4.39177489177489 7.29533882792843e-05 0.00525921633343093 231;760;2932;4193;5465;5468;5641;5747;6774;23621 AKR1B1;PPARG;BACE1;PPARA;GSK3B;PTK2;STAT3;CA2;MDM2;LGMN geneontology_Biological_Process

GO:1900034 regulation of cellular response to heat http://amigo.geneontology.org/amigo/term/GO:1900034 42 4 0.227698373583046 17.5670995670996 7.42693208963807e-05 0.00530627773047177 2932;3320;5594;9261 GSK3B;MAPKAPK2;MAPK1;HSP90AA1 geneontology_Biological_Process

GO:0051186 cofactor metabolic process http://amigo.geneontology.org/amigo/term/GO:0051186 339 9 1.83785115820601 4.89702333065165 7.63886223824617e-05 0.00540939607349078 231;1544;3320;3939;5465;6319;6774;8644;9536 AKR1B1;PPARA;AKR1C3;CYP1A2;PTGES;STAT3;HSP90AA1;LDHA;SCD geneontology_Biological_Process

GO:0002065 columnar/cuboidal epithelial cell differentiation http://amigo.geneontology.org/amigo/term/GO:0002065 82 5 0.444553967471661 11.2472283813747 7.73038082695354e-05 0.00542618485765634 2260;2932;5914;6098;6256 GSK3B;RXRA;FGFR1;RARA;ROS1 geneontology_Biological_Process

GO:0010888 negative regulation of lipid storage http://amigo.geneontology.org/amigo/term/GO:0010888 16 3 0.086742237555446 34.5852272727273 8.03274230682938e-05 0.00549384649053408 5465;5467;5468 PPARG;PPARA;PPARD geneontology_Biological_Process

GO:0042759 long-chain fatty acid biosynthetic process http://amigo.geneontology.org/amigo/term/GO:0042759 16 3 0.086742237555446 34.5852272727273 8.03274230682938e-05 0.00549384649053408 239;240;1544 ALOX12;CYP1A2;ALOX5 geneontology_Biological_Process

GO:0033189 response to vitamin A http://amigo.geneontology.org/amigo/term/GO:0033189 16 3 0.086742237555446 34.5852272727273 8.03274230682938e-05 0.00549384649053408 5467;5468;5914 PPARG;PPARD;RARA geneontology_Biological_Process

GO:0009914 hormone transport http://amigo.geneontology.org/amigo/term/GO:0009914 266 8 1.44208969935929 5.54750512645249 8.41150934127732e-05 0.00570414387702552 150;152;1268;2260;5467;5950;5972;6580 SLC22A1;REN;CNR1;ADRA2A;ADRA2C;FGFR1;PPARD;RBP4 geneontology_Biological_Process

GO:0031330 negative regulation of cellular catabolic process http://amigo.geneontology.org/amigo/term/GO:0031330 197 7 1.06801379990143 6.55422242731887 8.62586723102687e-05 0.00580035206577118 596;1268;3357;5465;5747;6774;9261 PPARA;CNR1;PTK2;MAPKAPK2;BCL2;STAT3;HTR2B geneontology_Biological_Process

GO:0003018 vascular process in circulatory system http://amigo.geneontology.org/amigo/term/GO:0003018 136 6 0.737309019221291 8.13770053475936 8.78528488934549e-05 0.00585832080704522 136;150;152;1636;3357;5467 ACE;ADORA2B;ADRA2A;ADRA2C;HTR2B;PPARD geneontology_Biological_Process

GO:0061614 pri-miRNA transcription by RNA polymerase II http://amigo.geneontology.org/amigo/term/GO:0061614 44 4 0.238541153277477 16.7685950413223 8.93545217610647e-05 0.00590921391018215 5465;5467;5468;6774 PPARG;PPARA;STAT3;PPARD geneontology_Biological_Process

GO:0045926 negative regulation of growth http://amigo.geneontology.org/amigo/term/GO:0045926 199 7 1.07885657959586 6.48835084513476 9.1911563221081e-05 0.00602849449914008 596;5465;5467;5468;5641;5747;5950 PPARG;PPARA;PTK2;BCL2;LGMN;PPARD;RBP4 geneontology_Biological_Process

GO:0006691 leukotriene metabolic process http://amigo.geneontology.org/amigo/term/GO:0006691 17 3 0.0921636274026614 32.5508021390374 9.71662404769535e-05 0.00632133541704539 239;240;9261 ALOX12;ALOX5;MAPKAPK2 geneontology_Biological_Process

GO:0097305 response to alcohol http://amigo.geneontology.org/amigo/term/GO:0097305 204 7 1.10596352883194 6.32932263814617 0.000107378384704093 0.00692936963227544 841;1268;5468;5914;5950;6774;8644 PPARG;CNR1;AKR1C3;STAT3;CASP8;RBP4;RARA geneontology_Biological_Process

GO:0007006 mitochondrial membrane organization http://amigo.geneontology.org/amigo/term/GO:0007006 88 5 0.477082306554953 10.4803719008264 0.000108291070840094 0.00693236119089947 596;841;2932;3320;6774 GSK3B;BCL2;STAT3;HSP90AA1;CASP8 geneontology_Biological_Process

GO:0009755 hormone-mediated signaling pathway http://amigo.geneontology.org/amigo/term/GO:0009755 205 7 1.11138491867915 6.29844789356985 0.000110712664471491 0.00702941883884276 5465;5467;5468;5914;5972;6256;7067 REN;PPARG;PPARA;RXRA;PPARD;RARA;THRA geneontology_Biological_Process

GO:0010950 positive regulation of endopeptidase activity http://amigo.geneontology.org/amigo/term/GO:0010950 142 6 0.769837358304584 7.79385403329065 0.000111564133033371 0.00702941883884276 834;836;841;5468;5641;6774 CASP3;PPARG;STAT3;CASP8;CASP1;LGMN geneontology_Biological_Process

GO:0070997 neuron death http://amigo.geneontology.org/amigo/term/GO:0070997 278 8 1.50714637752587 5.3080444735121 0.00011441768410525 0.00704129024338918 596;836;841;2932;5465;5641;6774;23621 CASP3;BACE1;PPARA;GSK3B;BCL2;STAT3;CASP8;LGMN geneontology_Biological_Process

GO:0001516 prostaglandin biosynthetic process http://amigo.geneontology.org/amigo/term/GO:0001516 18 3 0.0975850172498768 30.7424242424242 0.000116152250953183 0.00704129024338918 2171;8644;9536 FABP5;AKR1C3;PTGES geneontology_Biological_Process

GO:0046457 prostanoid biosynthetic process http://amigo.geneontology.org/amigo/term/GO:0046457 18 3 0.0975850172498768 30.7424242424242 0.000116152250953183 0.00704129024338918 2171;8644;9536 FABP5;AKR1C3;PTGES geneontology_Biological_Process

GO:0101023 vascular endothelial cell proliferation http://amigo.geneontology.org/amigo/term/GO:0101023 18 3 0.0975850172498768 30.7424242424242 0.000116152250953183 0.00704129024338918 2260;5468;6774 PPARG;STAT3;FGFR1 geneontology_Biological_Process

GO:1905562 regulation of vascular endothelial cell proliferation http://amigo.geneontology.org/amigo/term/GO:1905562 18 3 0.0975850172498768 30.7424242424242 0.000116152250953183 0.00704129024338918 2260;5468;6774 PPARG;STAT3;FGFR1 geneontology_Biological_Process

GO:0017158 regulation of calcium ion-dependent exocytosis http://amigo.geneontology.org/amigo/term/GO:0017158 91 5 0.493346476096599 10.1348651348651 0.000126976350710795 0.00763958464953217 136;150;1268;2932;23621 BACE1;GSK3B;CNR1;ADORA2B;ADRA2A geneontology_Biological_Process

GO:0071248 cellular response to metal ion http://amigo.geneontology.org/amigo/term/GO:0071248 149 6 0.807787087235091 7.42769981696156 0.000145383948999767 0.00868180865594128 1544;2167;5594;5641;8644;23621 BACE1;AKR1C3;CYP1A2;MAPK1;LGMN;FABP4 geneontology_Biological_Process

GO:0007610 behavior http://amigo.geneontology.org/amigo/term/GO:0007610 457 10 2.47757516017743 4.0362044957231 0.00014693170372615 0.0087092406904937 596;836;1268;3357;5465;5594;5641;5972;6774;7067 CASP3;REN;PPARA;CNR1;BCL2;MAPK1;STAT3;HTR2B;LGMN;THRA geneontology_Biological_Process

GO:0006066 alcohol metabolic process http://amigo.geneontology.org/amigo/term/GO:0006066 216 7 1.17102020699852 5.9776936026936 0.000153291651688203 0.00901941027065443 231;2260;5467;5950;6256;6319;8644 AKR1B1;AKR1C3;RXRA;FGFR1;PPARD;RBP4;SCD geneontology_Biological_Process

GO:0019433 triglyceride catabolic process http://amigo.geneontology.org/amigo/term/GO:0019433 20 3 0.108427796944308 27.6681818181818 0.000161029414417868 0.00940552827862614 2167;2171;11343 MGLL;FABP5;FABP4 geneontology_Biological_Process

GO:0070482 response to oxygen levels http://amigo.geneontology.org/amigo/term/GO:0070482 293 8 1.58846722523411 5.03630158237667 0.000164522877451834 0.00953994250267807 596;836;3939;4193;5328;5465;5467;5468 CASP3;PPARG;PPARA;BCL2;MDM2;LDHA;PLAU;PPARD geneontology_Biological_Process

GO:0060541 respiratory system development http://amigo.geneontology.org/amigo/term/GO:0060541 153 6 0.829472646623953 7.23351158645276 0.00016807985399292 0.00967607907662836 1544;2260;5594;5914;5950;7067 CYP1A2;MAPK1;FGFR1;RBP4;RARA;THRA geneontology_Biological_Process

GO:0010565 regulation of cellular ketone metabolic process http://amigo.geneontology.org/amigo/term/GO:0010565 97 5 0.525874815179892 9.50796626054358 0.000171685052764659 0.0098130270873057 1268;2171;5465;5468;8644 PPARG;PPARA;CNR1;FABP5;AKR1C3 geneontology_Biological_Process

GO:0015874 norepinephrine transport http://amigo.geneontology.org/amigo/term/GO:0015874 21 3 0.113849186791523 26.3506493506493 0.000187147448191149 0.0106209495065644 150;152;6580 SLC22A1;ADRA2A;ADRA2C geneontology_Biological_Process

GO:0046883 regulation of hormone secretion http://amigo.geneontology.org/amigo/term/GO:0046883 224 7 1.21439132577624 5.76420454545455 0.0001919328536093 0.0107401866753959 150;152;1268;2260;5467;5950;5972 REN;CNR1;ADRA2A;ADRA2C;FGFR1;PPARD;RBP4 geneontology_Biological_Process

GO:0071383 cellular response to steroid hormone stimulus http://amigo.geneontology.org/amigo/term/GO:0071383 224 7 1.21439132577624 5.76420454545455 0.0001919328536093 0.0107401866753959 5465;5467;5468;5914;6256;7067;8644 PPARG;PPARA;AKR1C3;RXRA;PPARD;RARA;THRA geneontology_Biological_Process

GO:0010952 positive regulation of peptidase activity http://amigo.geneontology.org/amigo/term/GO:0010952 159 6 0.862000985707245 6.96054888507719 0.000207296107629373 0.0114172913015981 834;836;841;5468;5641;6774 CASP3;PPARG;STAT3;CASP8;CASP1;LGMN geneontology_Biological_Process

GO:0003014 renal system process http://amigo.geneontology.org/amigo/term/GO:0003014 101 5 0.547560374568753 9.13141314131413 0.000207578905445316 0.0114172913015981 231;596;5641;5972;8644 REN;AKR1B1;AKR1C3;BCL2;LGMN geneontology_Biological_Process

GO:0001822 kidney development http://amigo.geneontology.org/amigo/term/GO:0001822 227 7 1.23065549531789 5.68802563075691 0.000208313487882195 0.0114172913015981 231;596;760;1636;2260;5914;5972 ACE;REN;AKR1B1;BCL2;CA2;FGFR1;RARA geneontology_Biological_Process

GO:0046461 neutral lipid catabolic process http://amigo.geneontology.org/amigo/term/GO:0046461 22 3 0.119270576638738 25.1528925619835 0.000215866477280113 0.0114394937165263 2167;2171;11343 MGLL;FABP5;FABP4 geneontology_Biological_Process

GO:0046464 acylglycerol catabolic process http://amigo.geneontology.org/amigo/term/GO:0046464 22 3 0.119270576638738 25.1528925619835 0.000215866477280113 0.0114394937165263 2167;2171;11343 MGLL;FABP5;FABP4 geneontology_Biological_Process

GO:0046320 regulation of fatty acid oxidation http://amigo.geneontology.org/amigo/term/GO:0046320 22 3 0.119270576638738 25.1528925619835 0.000215866477280113 0.0114394937165263 1268;5465;5468 PPARG;PPARA;CNR1 geneontology_Biological_Process

GO:0006692 prostanoid metabolic process http://amigo.geneontology.org/amigo/term/GO:0006692 22 3 0.119270576638738 25.1528925619835 0.000215866477280113 0.0114394937165263 2171;8644;9536 FABP5;AKR1C3;PTGES geneontology_Biological_Process

GO:0006693 prostaglandin metabolic process http://amigo.geneontology.org/amigo/term/GO:0006693 22 3 0.119270576638738 25.1528925619835 0.000215866477280113 0.0114394937165263 2171;8644;9536 FABP5;AKR1C3;PTGES geneontology_Biological_Process

GO:0007423 sensory organ development http://amigo.geneontology.org/amigo/term/GO:0007423 392 9 2.12518482010843 4.23492578849722 0.000229892803133569 0.0121026461228607 43;596;2260;5594;5914;5950;6256;6774;9261 ACHE;MAPKAPK2;BCL2;MAPK1;STAT3;RXRA;FGFR1;RBP4;RARA geneontology_Biological_Process

GO:0030335 positive regulation of cell migration http://amigo.geneontology.org/amigo/term/GO:0030335 393 9 2.13060620995564 4.22414989590562 0.000234304706129063 0.0122542892708808 150;596;2260;4193;5328;5594;5641;5747;6774 ADRA2A;PTK2;BCL2;MAPK1;STAT3;MDM2;FGFR1;PLAU;LGMN geneontology_Biological_Process

GO:0006721 terpenoid metabolic process http://amigo.geneontology.org/amigo/term/GO:0006721 57 4 0.309019221291277 12.9441786283892 0.000246783665139083 0.0127668106436092 1544;5467;5950;8644 AKR1C3;CYP1A2;PPARD;RBP4 geneontology_Biological_Process

GO:0032800 receptor biosynthetic process http://amigo.geneontology.org/amigo/term/GO:0032800 23 3 0.124691966485954 24.0592885375494 0.00024729513243682 0.0127668106436092 43;5465;5468 PPARG;PPARA;ACHE geneontology_Biological_Process

GO:0043406 positive regulation of MAP kinase activity http://amigo.geneontology.org/amigo/term/GO:0043406 234 7 1.2686052242484 5.51787101787102 0.000250964684905375 0.0128732013372616 136;150;2260;3357;5594;9020;9261 ADORA2B;MAP3K14;ADRA2A;MAPKAPK2;MAPK1;FGFR1;HTR2B geneontology_Biological_Process

GO:0019935 cyclic-nucleotide-mediated signaling http://amigo.geneontology.org/amigo/term/GO:0019935 165 6 0.894529324790537 6.70743801652893 0.000253415616618735 0.0129161258865167 136;150;152;3357;5141;5739 ADORA2B;PDE4A;ADRA2A;ADRA2C;HTR2B;PTGIR geneontology_Biological_Process

GO:0043401 steroid hormone mediated signaling pathway http://amigo.geneontology.org/amigo/term/GO:0043401 168 6 0.910793494332183 6.58766233766234 0.000279322010476646 0.0137025693908993 5465;5467;5468;5914;6256;7067 PPARG;PPARA;RXRA;PPARD;RARA;THRA geneontology_Biological_Process

GO:1903532 positive regulation of secretion by cell http://amigo.geneontology.org/amigo/term/GO:1903532 317 8 1.71858058156727 4.655004301692 0.00028153328835101 0.0137025693908993 43;136;834;1268;2260;3357;5467;5950 ACHE;CNR1;ADORA2B;CASP1;FGFR1;HTR2B;PPARD;RBP4 geneontology_Biological_Process

GO:0046902 regulation of mitochondrial membrane permeability http://amigo.geneontology.org/amigo/term/GO:0046902 59 4 0.319862000985707 12.5053929121726 0.000282069123119455 0.0137025693908993 596;841;2932;6774 GSK3B;BCL2;STAT3;CASP8 geneontology_Biological_Process

GO:0032811 negative regulation of epinephrine secretion http://amigo.geneontology.org/amigo/term/GO:0032811 5 2 0.0271069492360769 73.7818181818182 0.000285594782545751 0.0137025693908993 150;152 ADRA2A;ADRA2C geneontology_Biological_Process

GO:0035624 receptor transactivation http://amigo.geneontology.org/amigo/term/GO:0035624 5 2 0.0271069492360769 73.7818181818182 0.000285594782545751 0.0137025693908993 150;152 ADRA2A;ADRA2C geneontology_Biological_Process

GO:0030638 polyketide metabolic process http://amigo.geneontology.org/amigo/term/GO:0030638 5 2 0.0271069492360769 73.7818181818182 0.000285594782545751 0.0137025693908993 231;8644 AKR1B1;AKR1C3 geneontology_Biological_Process

GO:0044597 daunorubicin metabolic process http://amigo.geneontology.org/amigo/term/GO:0044597 5 2 0.0271069492360769 73.7818181818182 0.000285594782545751 0.0137025693908993 231;8644 AKR1B1;AKR1C3 geneontology_Biological_Process

GO:0044598 doxorubicin metabolic process http://amigo.geneontology.org/amigo/term/GO:0044598 5 2 0.0271069492360769 73.7818181818182 0.000285594782545751 0.0137025693908993 231;8644 AKR1B1;AKR1C3 geneontology_Biological_Process

GO:0038171 cannabinoid signaling pathway http://amigo.geneontology.org/amigo/term/GO:0038171 5 2 0.0271069492360769 73.7818181818182 0.000285594782545751 0.0137025693908993 1268;11343 CNR1;MGLL geneontology_Biological_Process

GO:0051222 positive regulation of protein transport http://amigo.geneontology.org/amigo/term/GO:0051222 318 8 1.72400197141449 4.64036592338479 0.000287597668884421 0.0137025693908993 43;834;2932;3357;4193;5467;5594;5950 ACHE;GSK3B;MAPK1;CASP1;MDM2;HTR2B;PPARD;RBP4 geneontology_Biological_Process

GO:2000147 positive regulation of cell motility http://amigo.geneontology.org/amigo/term/GO:2000147 404 9 2.19024149827501 4.10913591359136 0.00028768203669971 0.0137025693908993 150;596;2260;4193;5328;5594;5641;5747;6774 ADRA2A;PTK2;BCL2;MAPK1;STAT3;MDM2;FGFR1;PLAU;LGMN geneontology_Biological_Process

GO:0071241 cellular response to inorganic substance http://amigo.geneontology.org/amigo/term/GO:0071241 170 6 0.921636274026614 6.51016042780749 0.000297720469569551 0.0140967999851808 1544;2167;5594;5641;8644;23621 BACE1;AKR1C3;CYP1A2;MAPK1;LGMN;FABP4 geneontology_Biological_Process

GO:0048384 retinoic acid receptor signaling pathway http://amigo.geneontology.org/amigo/term/GO:0048384 25 3 0.135534746180384 22.1345454545455 0.000318706424519588 0.0149139696433084 5914;6256;8644 AKR1C3;RXRA;RARA geneontology_Biological_Process

GO:0001893 maternal placenta development http://amigo.geneontology.org/amigo/term/GO:0001893 25 3 0.135534746180384 22.1345454545455 0.000318706424519588 0.0149139696433084 1508;5467;6256 CTSB;RXRA;PPARD geneontology_Biological_Process

GO:0009062 fatty acid catabolic process http://amigo.geneontology.org/amigo/term/GO:0009062 61 4 0.330704780680138 12.0953800298063 0.000320841115565407 0.0149265732950836 1268;2166;5465;5467 PPARA;FAAH;CNR1;PPARD geneontology_Biological_Process

GO:0106106 cold-induced thermogenesis http://amigo.geneontology.org/amigo/term/GO:0106106 112 5 0.607195662888122 8.23457792207792 0.000336064415401127 0.0154551002990794 43;2167;2171;6319;7067 ACHE;FABP5;FABP4;SCD;THRA geneontology_Biological_Process

GO:0120161 regulation of cold-induced thermogenesis http://amigo.geneontology.org/amigo/term/GO:0120161 112 5 0.607195662888122 8.23457792207792 0.000336064415401127 0.0154551002990794 43;2167;2171;6319;7067 ACHE;FABP5;FABP4;SCD;THRA geneontology_Biological_Process

GO:0051272 positive regulation of cellular component movement http://amigo.geneontology.org/amigo/term/GO:0051272 413 9 2.23903400689995 4.01959057891261 0.000338552926348323 0.015472700067853 150;596;2260;4193;5328;5594;5641;5747;6774 ADRA2A;PTK2;BCL2;MAPK1;STAT3;MDM2;FGFR1;PLAU;LGMN geneontology_Biological_Process

GO:0015711 organic anion transport http://amigo.geneontology.org/amigo/term/GO:0015711 246 7 1.33366190241498 5.24870657797487 0.000340314322912039 0.015472700067853 760;1636;2167;5465;5467;5468;6256 ACE;PPARG;PPARA;RXRA;CA2;FABP4;PPARD geneontology_Biological_Process

GO:0019318 hexose metabolic process http://amigo.geneontology.org/amigo/term/GO:0019318 175 6 0.948743223262691 6.32415584415584 0.000347905351961053 0.0156546123930698 231;2171;2932;5465;5467;5950 AKR1B1;PPARA;GSK3B;FABP5;PPARD;RBP4 geneontology_Biological_Process

GO:0045471 response to ethanol http://amigo.geneontology.org/amigo/term/GO:0045471 113 5 0.612617052735338 8.16170555108608 0.000350184406193388 0.0156546123930698 841;1268;5914;5950;6774 CNR1;STAT3;CASP8;RBP4;RARA geneontology_Biological_Process

GO:1903364 positive regulation of cellular protein catabolic process http://amigo.geneontology.org/amigo/term/GO:1903364 113 5 0.612617052735338 8.16170555108608 0.000350184406193388 0.0156546123930698 2932;3320;4193;5747;9817 GSK3B;KEAP1;PTK2;HSP90AA1;MDM2 geneontology_Biological_Process

GO:0051953 negative regulation of amine transport http://amigo.geneontology.org/amigo/term/GO:0051953 26 3 0.1409561360276 21.2832167832168 0.000358896962591837 0.0159549638592216 150;152;1268 CNR1;ADRA2A;ADRA2C geneontology_Biological_Process

GO:0032355 response to estradiol http://amigo.geneontology.org/amigo/term/GO:0032355 114 5 0.618038442582553 8.09011164274322 0.000364749604470749 0.01612555986174 836;841;1544;5914;6774 CASP3;CYP1A2;STAT3;CASP8;RARA geneontology_Biological_Process

GO:0045732 positive regulation of protein catabolic process http://amigo.geneontology.org/amigo/term/GO:0045732 178 6 0.965007392804337 6.21756894790603 0.000381059957290208 0.0167540757045948 150;2932;3320;4193;5747;9817 GSK3B;ADRA2A;KEAP1;PTK2;HSP90AA1;MDM2 geneontology_Biological_Process

GO:0006367 transcription initiation from RNA polymerase II promoter http://amigo.geneontology.org/amigo/term/GO:0006367 179 6 0.970428782651553 6.18283392585069 0.000392645136388037 0.0171691059091643 5465;5467;5468;5914;6256;7067 PPARG;PPARA;RXRA;PPARD;RARA;THRA geneontology_Biological_Process

GO:0010677 negative regulation of cellular carbohydrate metabolic process http://amigo.geneontology.org/amigo/term/GO:0010677 27 3 0.146377525874815 20.4949494949495 0.000402212968027449 0.0172277103579159 2932;5465;6774 PPARA;GSK3B;STAT3 geneontology_Biological_Process

GO:0046621 negative regulation of organ growth http://amigo.geneontology.org/amigo/term/GO:0046621 27 3 0.146377525874815 20.4949494949495 0.000402212968027449 0.0172277103579159 5465;5747;5950 PPARA;PTK2;RBP4 geneontology_Biological_Process

GO:0019217 regulation of fatty acid metabolic process http://amigo.geneontology.org/amigo/term/GO:0019217 65 4 0.352390340068999 11.351048951049 0.000409627489401432 0.0172277103579159 1268;2171;5465;5468 PPARG;PPARA;CNR1;FABP5 geneontology_Biological_Process

GO:0042310 vasoconstriction http://amigo.geneontology.org/amigo/term/GO:0042310 65 4 0.352390340068999 11.351048951049 0.000409627489401432 0.0172277103579159 150;152;1636;3357 ACE;ADRA2A;ADRA2C;HTR2B geneontology_Biological_Process

GO:0038034 signal transduction in absence of ligand http://amigo.geneontology.org/amigo/term/GO:0038034 65 4 0.352390340068999 11.351048951049 0.000409627489401432 0.0172277103579159 596;836;2260;2932 CASP3;GSK3B;BCL2;FGFR1 geneontology_Biological_Process

GO:0097192 extrinsic apoptotic signaling pathway in absence of ligand http://amigo.geneontology.org/amigo/term/GO:0097192 65 4 0.352390340068999 11.351048951049 0.000409627489401432 0.0172277103579159 596;836;2260;2932 CASP3;GSK3B;BCL2;FGFR1 geneontology_Biological_Process

GO:0090559 regulation of membrane permeability http://amigo.geneontology.org/amigo/term/GO:0090559 65 4 0.352390340068999 11.351048951049 0.000409627489401432 0.0172277103579159 596;841;2932;6774 GSK3B;BCL2;STAT3;CASP8 geneontology_Biological_Process

GO:1990845 adaptive thermogenesis http://amigo.geneontology.org/amigo/term/GO:1990845 117 5 0.634302612124199 7.88267288267288 0.000411208782599592 0.0172277103579159 43;2167;2171;6319;7067 ACHE;FABP5;FABP4;SCD;THRA geneontology_Biological_Process

GO:0014060 regulation of epinephrine secretion http://amigo.geneontology.org/amigo/term/GO:0014060 6 2 0.0325283390832923 61.4848484848485 0.000426901489302356 0.0174283062350859 150;152 ADRA2A;ADRA2C geneontology_Biological_Process

GO:0048242 epinephrine secretion http://amigo.geneontology.org/amigo/term/GO:0048242 6 2 0.0325283390832923 61.4848484848485 0.000426901489302356 0.0174283062350859 150;152 ADRA2A;ADRA2C geneontology_Biological_Process

GO:0030647 aminoglycoside antibiotic metabolic process http://amigo.geneontology.org/amigo/term/GO:0030647 6 2 0.0325283390832923 61.4848484848485 0.000426901489302356 0.0174283062350859 231;8644 AKR1B1;AKR1C3 geneontology_Biological_Process

GO:0010871 negative regulation of receptor biosynthetic process http://amigo.geneontology.org/amigo/term/GO:0010871 6 2 0.0325283390832923 61.4848484848485 0.000426901489302356 0.0174283062350859 5465;5468 PPARG;PPARA geneontology_Biological_Process

GO:0033500 carbohydrate homeostasis http://amigo.geneontology.org/amigo/term/GO:0033500 182 6 0.986692952193199 6.08091908091908 0.000429064774845278 0.0174283062350859 150;1268;2171;5468;5950;6774 PPARG;CNR1;ADRA2A;FABP5;STAT3;RBP4 geneontology_Biological_Process

GO:0042593 glucose homeostasis http://amigo.geneontology.org/amigo/term/GO:0042593 182 6 0.986692952193199 6.08091908091908 0.000429064774845278 0.0174283062350859 150;1268;2171;5468;5950;6774 PPARG;CNR1;ADRA2A;FABP5;STAT3;RBP4 geneontology_Biological_Process

GO:0055017 cardiac muscle tissue growth http://amigo.geneontology.org/amigo/term/GO:0055017 66 4 0.357811729916215 11.1790633608815 0.000434310592076326 0.0175522896858321 2260;5465;5950;6256 PPARA;RXRA;FGFR1;RBP4 geneontology_Biological_Process

GO:0002444 myeloid leukocyte mediated immunity http://amigo.geneontology.org/amigo/term/GO:0002444 428 9 2.32035485460818 3.87871707731521 0.000439850855066037 0.0176868670464243 136;240;1508;1636;2171;3320;5328;5594;23385 ACE;ADORA2B;FABP5;ALOX5;NCSTN;CTSB;MAPK1;HSP90AA1;PLAU geneontology_Biological_Process

GO:0006775 fat-soluble vitamin metabolic process http://amigo.geneontology.org/amigo/term/GO:0006775 28 3 0.151798915722031 19.762987012987 0.000448753828234394 0.0178505924436014 2260;5467;5641 FGFR1;LGMN;PPARD geneontology_Biological_Process

GO:0046879 hormone secretion http://amigo.geneontology.org/amigo/term/GO:0046879 258 7 1.39871858058157 5.0045806906272 0.000453651743545724 0.0178505924436014 150;152;1268;2260;5467;5950;5972 REN;CNR1;ADRA2A;ADRA2C;FGFR1;PPARD;RBP4 geneontology_Biological_Process

GO:0015849 organic acid transport http://amigo.geneontology.org/amigo/term/GO:0015849 184 6 0.997535731887629 6.01482213438735 0.000454777281848839 0.0178505924436014 1636;2167;5465;5467;5468;6256 ACE;PPARG;PPARA;RXRA;FABP4;PPARD geneontology_Biological_Process

GO:0046942 carboxylic acid transport http://amigo.geneontology.org/amigo/term/GO:0046942 184 6 0.997535731887629 6.01482213438735 0.000454777281848839 0.0178505924436014 1636;2167;5465;5467;5468;6256 ACE;PPARG;PPARA;RXRA;FABP4;PPARD geneontology_Biological_Process

GO:0040017 positive regulation of locomotion http://amigo.geneontology.org/amigo/term/GO:0040017 430 9 2.33119763430261 3.86067653276956 0.000455076338227278 0.0178505924436014 150;596;2260;4193;5328;5594;5641;5747;6774 ADRA2A;PTK2;BCL2;MAPK1;STAT3;MDM2;FGFR1;PLAU;LGMN geneontology_Biological_Process

GO:0048659 smooth muscle cell proliferation http://amigo.geneontology.org/amigo/term/GO:0048659 120 5 0.650566781665845 7.68560606060606 0.000462010457455531 0.0179466392260153 231;4193;5467;5468;5739 AKR1B1;PPARG;MDM2;PPARD;PTGIR geneontology_Biological_Process

GO:0048660 regulation of smooth muscle cell proliferation http://amigo.geneontology.org/amigo/term/GO:0048660 120 5 0.650566781665845 7.68560606060606 0.000462010457455531 0.0179466392260153 231;4193;5467;5468;5739 AKR1B1;PPARG;MDM2;PPARD;PTGIR geneontology_Biological_Process

GO:0051937 catecholamine transport http://amigo.geneontology.org/amigo/term/GO:0051937 68 4 0.368654509610646 10.8502673796791 0.000486835564808108 0.0187291259115119 150;152;1268;6580 SLC22A1;CNR1;ADRA2A;ADRA2C geneontology_Biological_Process

GO:0006720 isoprenoid metabolic process http://amigo.geneontology.org/amigo/term/GO:0006720 68 4 0.368654509610646 10.8502673796791 0.000486835564808108 0.0187291259115119 1544;5467;5950;8644 AKR1C3;CYP1A2;PPARD;RBP4 geneontology_Biological_Process

GO:0051930 regulation of sensory perception of pain http://amigo.geneontology.org/amigo/term/GO:0051930 29 3 0.157220305569246 19.0815047021944 0.000498617139886193 0.0189996873969967 152;2171;11343 ADRA2C;MGLL;FABP5 geneontology_Biological_Process

GO:0071276 cellular response to cadmium ion http://amigo.geneontology.org/amigo/term/GO:0071276 29 3 0.157220305569246 19.0815047021944 0.000498617139886193 0.0189996873969967 1544;5594;8644 AKR1C3;CYP1A2;MAPK1 geneontology_Biological_Process

GO:0051047 positive regulation of secretion http://amigo.geneontology.org/amigo/term/GO:0051047 346 8 1.87580088713652 4.26484498160799 0.000506325722524181 0.0192019830883341 43;136;834;1268;2260;3357;5467;5950 ACHE;CNR1;ADORA2B;CASP1;FGFR1;HTR2B;PPARD;RBP4 geneontology_Biological_Process

GO:0001666 response to hypoxia http://amigo.geneontology.org/amigo/term/GO:0001666 263 7 1.42582552981764 4.90943657103353 0.000508996216814328 0.0192122062591899 596;836;3939;4193;5328;5465;5467 CASP3;PPARA;BCL2;MDM2;LDHA;PLAU;PPARD geneontology_Biological_Process

GO:0062014 negative regulation of small molecule metabolic process http://amigo.geneontology.org/amigo/term/GO:0062014 69 4 0.374075899457861 10.6930171277997 0.000514727811866567 0.0192573931123266 1268;5465;6774;8644 PPARA;CNR1;AKR1C3;STAT3 geneontology_Biological_Process

GO:0038127 ERBB signaling pathway http://amigo.geneontology.org/amigo/term/GO:0038127 123 5 0.666830951207491 7.49815225424982 0.00051741308662212 0.0192573931123266 150;3320;5594;5641;5747 ADRA2A;PTK2;MAPK1;HSP90AA1;LGMN geneontology_Biological_Process

GO:2001056 positive regulation of cysteine-type endopeptidase activity http://amigo.geneontology.org/amigo/term/GO:2001056 123 5 0.666830951207491 7.49815225424982 0.00051741308662212 0.0192573931123266 834;836;841;5468;5641 CASP3;PPARG;CASP8;CASP1;LGMN geneontology_Biological_Process

GO:0005996 monosaccharide metabolic process http://amigo.geneontology.org/amigo/term/GO:0005996 189 6 1.02464268112371 5.85569985569986 0.000524347662753 0.0194251388766181 231;2171;2932;5465;5467;5950 AKR1B1;PPARA;GSK3B;FABP5;PPARD;RBP4 geneontology_Biological_Process

GO:0072329 monocarboxylic acid catabolic process http://amigo.geneontology.org/amigo/term/GO:0072329 70 4 0.379497289305076 10.5402597402597 0.000543740293666217 0.0199656759061832 1268;2166;5465;5467 PPARA;FAAH;CNR1;PPARD geneontology_Biological_Process

GO:0050708 regulation of protein secretion http://amigo.geneontology.org/amigo/term/GO:0050708 350 8 1.89748644652538 4.2161038961039 0.000546423771988769 0.0199656759061832 43;150;152;834;1268;3357;5467;5950 ACHE;CNR1;ADRA2A;ADRA2C;CASP1;HTR2B;PPARD;RBP4 geneontology_Biological_Process

GO:0005975 carbohydrate metabolic process http://amigo.geneontology.org/amigo/term/GO:0005975 350 8 1.89748644652538 4.2161038961039 0.000546423771988769 0.0199656759061832 231;2171;2932;3939;5465;5467;5950;6774 AKR1B1;PPARA;GSK3B;FABP5;STAT3;LDHA;PPARD;RBP4 geneontology_Biological_Process

GO:0051931 regulation of sensory perception http://amigo.geneontology.org/amigo/term/GO:0051931 30 3 0.162641695416461 18.4454545454545 0.000551898727856215 0.0199832290511558 152;2171;11343 ADRA2C;MGLL;FABP5 geneontology_Biological_Process

GO:1904646 cellular response to amyloid-beta http://amigo.geneontology.org/amigo/term/GO:1904646 30 3 0.162641695416461 18.4454545454545 0.000551898727856215 0.0199832290511558 2932;5641;23621 BACE1;GSK3B;LGMN geneontology_Biological_Process

GO:0014065 phosphatidylinositol 3-kinase signaling http://amigo.geneontology.org/amigo/term/GO:0014065 125 5 0.677673730901922 7.37818181818182 0.000557034528336775 0.0200783346655445 2260;3357;5467;5594;5747 PTK2;MAPK1;FGFR1;HTR2B;PPARD geneontology_Biological_Process

GO:0097756 negative regulation of blood vessel diameter http://amigo.geneontology.org/amigo/term/GO:0097756 71 4 0.384918679152292 10.3918053777209 0.000573898403705364 0.0205014956537961 150;152;1636;3357 ACE;ADRA2A;ADRA2C;HTR2B geneontology_Biological_Process

GO:2000300 regulation of synaptic vesicle exocytosis http://amigo.geneontology.org/amigo/term/GO:2000300 71 4 0.384918679152292 10.3918053777209 0.000573898403705364 0.0205014956537961 136;1268;2932;23621 BACE1;GSK3B;CNR1;ADORA2B geneontology_Biological_Process

GO:0000302 response to reactive oxygen species http://amigo.geneontology.org/amigo/term/GO:0000302 193 6 1.04632824051257 5.73433820065944 0.000585763553837326 0.0208115997680707 596;836;3939;4193;5594;8644 CASP3;AKR1C3;BCL2;MAPK1;MDM2;LDHA geneontology_Biological_Process

GO:0010889 regulation of sequestering of triglyceride http://amigo.geneontology.org/amigo/term/GO:0010889 7 2 0.0379497289305076 52.7012987012987 0.00059558314757413 0.0208115997680707 5465;5468 PPARG;PPARA geneontology_Biological_Process

GO:0010966 regulation of phosphate transport http://amigo.geneontology.org/amigo/term/GO:0010966 7 2 0.0379497289305076 52.7012987012987 0.00059558314757413 0.0208115997680707 2260;6098 FGFR1;ROS1 geneontology_Biological_Process

GO:0099541 trans-synaptic signaling by lipid http://amigo.geneontology.org/amigo/term/GO:0099541 7 2 0.0379497289305076 52.7012987012987 0.00059558314757413 0.0208115997680707 1268;2171 CNR1;FABP5 geneontology_Biological_Process

GO:0099542 trans-synaptic signaling by endocannabinoid http://amigo.geneontology.org/amigo/term/GO:0099542 7 2 0.0379497289305076 52.7012987012987 0.00059558314757413 0.0208115997680707 1268;2171 CNR1;FABP5 geneontology_Biological_Process

GO:0060419 heart growth http://amigo.geneontology.org/amigo/term/GO:0060419 72 4 0.390340068999507 10.2474747474747 0.000605227606314207 0.0209045437650698 2260;5465;5950;6256 PPARA;RXRA;FGFR1;RBP4 geneontology_Biological_Process

GO:0046503 glycerolipid catabolic process http://amigo.geneontology.org/amigo/term/GO:0046503 31 3 0.168063085263677 17.8504398826979 0.000608692663991661 0.0209045437650698 2167;2171;11343 MGLL;FABP5;FABP4 geneontology_Biological_Process

GO:0045912 negative regulation of carbohydrate metabolic process http://amigo.geneontology.org/amigo/term/GO:0045912 31 3 0.168063085263677 17.8504398826979 0.000608692663991661 0.0209045437650698 2932;5465;6774 PPARA;GSK3B;STAT3 geneontology_Biological_Process

GO:1901030 positive regulation of mitochondrial outer membrane permeabilization involved in apoptotic signaling pathway http://amigo.geneontology.org/amigo/term/GO:1901030 31 3 0.168063085263677 17.8504398826979 0.000608692663991661 0.0209045437650698 596;841;2932 GSK3B;BCL2;CASP8 geneontology_Biological_Process

GO:0036293 response to decreased oxygen levels http://amigo.geneontology.org/amigo/term/GO:0036293 272 7 1.47461803844258 4.74699197860963 0.000622148342323436 0.0212753463045818 596;836;3939;4193;5328;5465;5467 CASP3;PPARA;BCL2;MDM2;LDHA;PLAU;PPARD geneontology_Biological_Process

GO:1902803 regulation of synaptic vesicle transport http://amigo.geneontology.org/amigo/term/GO:1902803 74 4 0.401182848693938 9.97051597051597 0.000671501464498236 0.0228653392294251 136;1268;2932;23621 BACE1;GSK3B;CNR1;ADORA2B geneontology_Biological_Process

GO:0017156 calcium ion regulated exocytosis http://amigo.geneontology.org/amigo/term/GO:0017156 131 5 0.710202069985214 7.04024982650937 0.000689674218723901 0.0233846317721553 136;150;1268;2932;23621 BACE1;GSK3B;CNR1;ADORA2B;ADRA2A geneontology_Biological_Process

GO:0015844 monoamine transport http://amigo.geneontology.org/amigo/term/GO:0015844 76 4 0.412025628388369 9.70813397129187 0.000742766771864911 0.0249244426225864 150;152;1268;6580 SLC22A1;CNR1;ADRA2A;ADRA2C geneontology_Biological_Process

GO:0003007 heart morphogenesis http://amigo.geneontology.org/amigo/term/GO:0003007 202 6 1.09512074913751 5.47884788478848 0.000744431615445906 0.0249244426225864 3357;4193;5747;5914;5950;6256 PTK2;RXRA;MDM2;HTR2B;RBP4;RARA geneontology_Biological_Process

GO:0045165 cell fate commitment http://amigo.geneontology.org/amigo/term/GO:0045165 202 6 1.09512074913751 5.47884788478848 0.000744431615445906 0.0249244426225864 596;836;2260;5468;5914;6774 CASP3;PPARG;BCL2;STAT3;FGFR1;RARA geneontology_Biological_Process

GO:1903305 regulation of regulated secretory pathway http://amigo.geneontology.org/amigo/term/GO:1903305 134 5 0.726466239526861 6.88263229308005 0.000764249430845521 0.0253295179428862 136;150;1268;2932;23621 BACE1;GSK3B;CNR1;ADORA2B;ADRA2A geneontology_Biological_Process

GO:0046620 regulation of organ growth http://amigo.geneontology.org/amigo/term/GO:0046620 77 4 0.417447018235584 9.58205430932704 0.000780335463004489 0.0253295179428862 2260;5465;5747;5950 PPARA;PTK2;FGFR1;RBP4 geneontology_Biological_Process

GO:2000116 regulation of cysteine-type endopeptidase activity http://amigo.geneontology.org/amigo/term/GO:2000116 204 6 1.10596352883194 5.42513368983957 0.000783828898340166 0.0253295179428862 834;836;841;4193;5468;5641 CASP3;PPARG;CASP8;CASP1;MDM2;LGMN geneontology_Biological_Process

GO:0010700 negative regulation of norepinephrine secretion http://amigo.geneontology.org/amigo/term/GO:0010700 8 2 0.043371118777723 46.1136363636364 0.000791349598315616 0.0253295179428862 150;152 ADRA2A;ADRA2C geneontology_Biological_Process

GO:0002016 regulation of blood volume by renin-angiotensin http://amigo.geneontology.org/amigo/term/GO:0002016 8 2 0.043371118777723 46.1136363636364 0.000791349598315616 0.0253295179428862 1636;5972 ACE;REN geneontology_Biological_Process

GO:0032025 response to cobalt ion http://amigo.geneontology.org/amigo/term/GO:0032025 8 2 0.043371118777723 46.1136363636364 0.000791349598315616 0.0253295179428862 836;841 CASP3;CASP8 geneontology_Biological_Process

GO:0019372 lipoxygenase pathway http://amigo.geneontology.org/amigo/term/GO:0019372 8 2 0.043371118777723 46.1136363636364 0.000791349598315616 0.0253295179428862 239;240 ALOX12;ALOX5 geneontology_Biological_Process

GO:0098917 retrograde trans-synaptic signaling http://amigo.geneontology.org/amigo/term/GO:0098917 8 2 0.043371118777723 46.1136363636364 0.000791349598315616 0.0253295179428862 1268;2171 CNR1;FABP5 geneontology_Biological_Process

GO:0002002 regulation of angiotensin levels in blood http://amigo.geneontology.org/amigo/term/GO:0002002 8 2 0.043371118777723 46.1136363636364 0.000791349598315616 0.0253295179428862 1636;5972 ACE;REN geneontology_Biological_Process

GO:0002003 angiotensin maturation http://amigo.geneontology.org/amigo/term/GO:0002003 8 2 0.043371118777723 46.1136363636364 0.000791349598315616 0.0253295179428862 1636;5972 ACE;REN geneontology_Biological_Process

GO:0042473 outer ear morphogenesis http://amigo.geneontology.org/amigo/term/GO:0042473 8 2 0.043371118777723 46.1136363636364 0.000791349598315616 0.0253295179428862 2260;5594 MAPK1;FGFR1 geneontology_Biological_Process

GO:0031331 positive regulation of cellular catabolic process http://amigo.geneontology.org/amigo/term/GO:0031331 284 7 1.53967471660917 4.54641485275288 0.000803292078694673 0.0255341235279461 150;2932;3320;4193;5465;5747;9817 PPARA;GSK3B;ADRA2A;KEAP1;PTK2;HSP90AA1;MDM2 geneontology_Biological_Process

GO:0072593 reactive oxygen species metabolic process http://amigo.geneontology.org/amigo/term/GO:0072593 205 6 1.11138491867915 5.39866962305987 0.000804123860165262 0.0255341235279461 239;596;1544;3320;6774;8644 ALOX12;AKR1C3;CYP1A2;BCL2;STAT3;HSP90AA1 geneontology_Biological_Process

GO:0030324 lung development http://amigo.geneontology.org/amigo/term/GO:0030324 136 5 0.737309019221291 6.78141711229947 0.000817215121300952 0.0258089447284066 1544;2260;5594;5950;7067 CYP1A2;MAPK1;FGFR1;RBP4;THRA geneontology_Biological_Process

GO:1901216 positive regulation of neuron death http://amigo.geneontology.org/amigo/term/GO:1901216 78 4 0.422868408082799 9.45920745920746 0.000819229187829951 0.0258089447284066 836;841;2932;23621 CASP3;BACE1;GSK3B;CASP8 geneontology_Biological_Process

GO:0007187 G protein-coupled receptor signaling pathway, coupled to cyclic nucleotide second messenger http://amigo.geneontology.org/amigo/term/GO:0007187 206 6 1.11680630852637 5.37246248896734 0.000824823649310025 0.0258832895756032 136;150;152;1268;3357;5739 CNR1;ADORA2B;ADRA2A;ADRA2C;HTR2B;PTGIR geneontology_Biological_Process

GO:0002791 regulation of peptide secretion http://amigo.geneontology.org/amigo/term/GO:0002791 374 8 2.02759980285855 3.94555177442878 0.000844787102202371 0.0263391518116719 43;150;152;834;1268;3357;5467;5950 ACHE;CNR1;ADRA2A;ADRA2C;CASP1;HTR2B;PPARD;RBP4 geneontology_Biological_Process

GO:0031668 cellular response to extracellular stimulus http://amigo.geneontology.org/amigo/term/GO:0031668 207 6 1.12222769837358 5.34650856389987 0.000845933768507834 0.0263391518116719 136;596;4193;5468;5594;8644 PPARG;ADORA2B;AKR1C3;BCL2;MAPK1;MDM2 geneontology_Biological_Process

GO:0043410 positive regulation of MAPK cascade http://amigo.geneontology.org/amigo/term/GO:0043410 470 9 2.54805322819123 3.5321083172147 0.000864025595986195 0.026761603478216 136;150;152;2260;3357;5594;6098;9020;9261 ADORA2B;MAP3K14;ADRA2A;ADRA2C;MAPKAPK2;MAPK1;FGFR1;HTR2B;ROS1 geneontology_Biological_Process

GO:0010883 regulation of lipid storage http://amigo.geneontology.org/amigo/term/GO:0010883 35 3 0.189748644652538 15.8103896103896 0.000872813009284323 0.026761603478216 5465;5467;5468 PPARG;PPARA;PPARD geneontology_Biological_Process

GO:0038083 peptidyl-tyrosine autophosphorylation http://amigo.geneontology.org/amigo/term/GO:0038083 35 3 0.189748644652538 15.8103896103896 0.000872813009284323 0.026761603478216 1636;5747;6098 ACE;PTK2;ROS1 geneontology_Biological_Process

GO:0030323 respiratory tube development http://amigo.geneontology.org/amigo/term/GO:0030323 138 5 0.748151798915722 6.68313570487483 0.000872879093703371 0.026761603478216 1544;2260;5594;5950;7067 CYP1A2;MAPK1;FGFR1;RBP4;THRA geneontology_Biological_Process

GO:0060485 mesenchyme development http://amigo.geneontology.org/amigo/term/GO:0060485 209 6 1.13307047806801 5.29534580252284 0.000889407175975743 0.0271270741092215 596;2260;2932;3357;4193;5594 GSK3B;BCL2;MAPK1;MDM2;FGFR1;HTR2B geneontology_Biological_Process

GO:2000060 positive regulation of ubiquitin-dependent protein catabolic process http://amigo.geneontology.org/amigo/term/GO:2000060 80 4 0.43371118777723 9.22272727272727 0.000901094947367009 0.0271270741092215 2932;4193;5747;9817 GSK3B;KEAP1;PTK2;MDM2 geneontology_Biological_Process

GO:0006919 activation of cysteine-type endopeptidase activity involved in apoptotic process http://amigo.geneontology.org/amigo/term/GO:0006919 80 4 0.43371118777723 9.22272727272727 0.000901094947367009 0.0271270741092215 834;836;841;5468 CASP3;PPARG;CASP8;CASP1 geneontology_Biological_Process

GO:0050796 regulation of insulin secretion http://amigo.geneontology.org/amigo/term/GO:0050796 139 5 0.753573188762937 6.63505559189012 0.000901749776687444 0.0271270741092215 150;152;1268;5467;5950 CNR1;ADRA2A;ADRA2C;PPARD;RBP4 geneontology_Biological_Process

GO:0019933 cAMP-mediated signaling http://amigo.geneontology.org/amigo/term/GO:0019933 139 5 0.753573188762937 6.63505559189012 0.000901749776687444 0.0271270741092215 136;150;152;5141;5739 ADORA2B;PDE4A;ADRA2A;ADRA2C;PTGIR geneontology_Biological_Process

GO:0071902 positive regulation of protein serine/threonine kinase activity http://amigo.geneontology.org/amigo/term/GO:0071902 290 7 1.57220305569246 4.45235109717868 0.000908349006188081 0.0272232537360188 136;150;2260;3357;5594;9020;9261 ADORA2B;MAP3K14;ADRA2A;MAPKAPK2;MAPK1;FGFR1;HTR2B geneontology_Biological_Process

GO:1904645 response to amyloid-beta http://amigo.geneontology.org/amigo/term/GO:1904645 36 3 0.195170034499754 15.3712121212121 0.000948519702345219 0.0283210994707703 2932;5641;23621 BACE1;GSK3B;LGMN geneontology_Biological_Process

GO:0030258 lipid modification http://amigo.geneontology.org/amigo/term/GO:0030258 212 6 1.14933464760966 5.22041166380789 0.000957834251910428 0.0284928984527407 239;1268;2260;5465;5467;5468 PPARG;PPARA;CNR1;ALOX12;FGFR1;PPARD geneontology_Biological_Process

GO:0002446 neutrophil mediated immunity http://amigo.geneontology.org/amigo/term/GO:0002446 382 8 2.07097092163627 3.86292241789624 0.000969341988812378 0.0287284244239876 240;1508;1636;2171;3320;5328;5594;23385 ACE;FABP5;ALOX5;NCSTN;CTSB;MAPK1;HSP90AA1;PLAU geneontology_Biological_Process

GO:0019371 cyclooxygenase pathway http://amigo.geneontology.org/amigo/term/GO:0019371 9 2 0.0487925086249384 40.989898989899 0.00101391292522557 0.0298284236310846 8644;9536 AKR1C3;PTGES geneontology_Biological_Process

GO:0032966 negative regulation of collagen biosynthetic process http://amigo.geneontology.org/amigo/term/GO:0032966 9 2 0.0487925086249384 40.989898989899 0.00101391292522557 0.0298284236310846 5467;5468 PPARG;PPARD geneontology_Biological_Process

GO:1905953 negative regulation of lipid localization http://amigo.geneontology.org/amigo/term/GO:1905953 37 3 0.200591424346969 14.955773955774 0.00102826739262729 0.0298700139412572 5465;5467;5468 PPARG;PPARA;PPARD geneontology_Biological_Process

GO:0034308 primary alcohol metabolic process http://amigo.geneontology.org/amigo/term/GO:0034308 37 3 0.200591424346969 14.955773955774 0.00102826739262729 0.0298700139412572 231;5950;8644 AKR1B1;AKR1C3;RBP4 geneontology_Biological_Process

GO:1901028 regulation of mitochondrial outer membrane permeabilization involved in apoptotic signaling pathway http://amigo.geneontology.org/amigo/term/GO:1901028 37 3 0.200591424346969 14.955773955774 0.00102826739262729 0.0298700139412572 596;841;2932 GSK3B;BCL2;CASP8 geneontology_Biological_Process

GO:0034612 response to tumor necrosis factor http://amigo.geneontology.org/amigo/term/GO:0034612 215 6 1.16559881715131 5.14756871035941 0.00103025791649425 0.0298700139412572 834;836;841;2167;5594;9020 CASP3;MAP3K14;MAPK1;CASP8;CASP1;FABP4 geneontology_Biological_Process

GO:1903522 regulation of blood circulation http://amigo.geneontology.org/amigo/term/GO:1903522 216 6 1.17102020699852 5.12373737373737 0.00105531409548876 0.0303136654475889 140;150;152;1636;4193;7067 ACE;ADORA3;ADRA2A;ADRA2C;MDM2;THRA geneontology_Biological_Process

GO:0007548 sex differentiation http://amigo.geneontology.org/amigo/term/GO:0007548 216 6 1.17102020699852 5.12373737373737 0.00105531409548876 0.0303136654475889 596;836;5914;5950;5972;8644 CASP3;REN;AKR1C3;BCL2;RBP4;RARA geneontology_Biological_Process

GO:0008217 regulation of blood pressure http://amigo.geneontology.org/amigo/term/GO:0008217 144 5 0.780680137999014 6.40467171717172 0.00105692485127185 0.0303136654475889 1268;1636;5465;5468;5972 ACE;REN;PPARG;PPARA;CNR1 geneontology_Biological_Process

GO:0009165 nucleotide biosynthetic process http://amigo.geneontology.org/amigo/term/GO:0009165 218 6 1.18186298669295 5.0767306088407 0.00110683380659704 0.0316317290013911 136;3939;5465;5594;6319;6774 PPARA;ADORA2B;MAPK1;STAT3;LDHA;SCD geneontology_Biological_Process

GO:0034605 cellular response to heat http://amigo.geneontology.org/amigo/term/GO:0034605 85 4 0.460818137013307 8.68021390374332 0.00113075555542963 0.032200377062448 2932;3320;5594;9261 GSK3B;MAPKAPK2;MAPK1;HSP90AA1 geneontology_Biological_Process

GO:0050727 regulation of inflammatory response http://amigo.geneontology.org/amigo/term/GO:0050727 302 7 1.63725973385904 4.27543648404576 0.00115100625869868 0.0326608229861946 136;1268;2167;5465;5467;5468;11343 PPARG;PPARA;CNR1;ADORA2B;MGLL;FABP4;PPARD geneontology_Biological_Process

GO:0048015 phosphatidylinositol-mediated signaling http://amigo.geneontology.org/amigo/term/GO:0048015 147 5 0.79694430754066 6.27396413110699 0.00115908334423753 0.0326918102646712 2260;3357;5467;5594;5747 PTK2;MAPK1;FGFR1;HTR2B;PPARD geneontology_Biological_Process

GO:1901293 nucleoside phosphate biosynthetic process http://amigo.geneontology.org/amigo/term/GO:1901293 220 6 1.19270576638738 5.03057851239669 0.00116026919709655 0.0326918102646712 136;3939;5465;5594;6319;6774 PPARA;ADORA2B;MAPK1;STAT3;LDHA;SCD geneontology_Biological_Process

GO:0050673 epithelial cell proliferation http://amigo.geneontology.org/amigo/term/GO:0050673 303 7 1.64268112370626 4.26132613261326 0.00117332894598432 0.0329437832483036 2260;3357;5467;5468;5594;6774;23385 PPARG;NCSTN;MAPK1;STAT3;FGFR1;HTR2B;PPARD geneontology_Biological_Process

GO:0046928 regulation of neurotransmitter secretion http://amigo.geneontology.org/amigo/term/GO:0046928 86 4 0.466239526860522 8.57928118393235 0.00118117964724385 0.0330482501302282 136;1268;2932;23621 BACE1;GSK3B;CNR1;ADORA2B geneontology_Biological_Process

GO:0006509 membrane protein ectodomain proteolysis http://amigo.geneontology.org/amigo/term/GO:0006509 39 3 0.2114342040414 14.1888111888112 0.0012002134090987 0.0333475961791939 150;23385;23621 BACE1;ADRA2A;NCSTN geneontology_Biological_Process

GO:1902893 regulation of pri-miRNA transcription by RNA polymerase II http://amigo.geneontology.org/amigo/term/GO:1902893 39 3 0.2114342040414 14.1888111888112 0.0012002134090987 0.0333475961791939 5465;5467;6774 PPARA;STAT3;PPARD geneontology_Biological_Process

GO:0043405 regulation of MAP kinase activity http://amigo.geneontology.org/amigo/term/GO:0043405 305 7 1.65352390340069 4.23338301043219 0.00121899764056455 0.0337523152934169 136;150;2260;3357;5594;9020;9261 ADORA2B;MAP3K14;ADRA2A;MAPKAPK2;MAPK1;FGFR1;HTR2B geneontology_Biological_Process

GO:0048017 inositol lipid-mediated signaling http://amigo.geneontology.org/amigo/term/GO:0048017 149 5 0.807787087235091 6.18974984746797 0.00123114680043646 0.033971161024457 2260;3357;5467;5594;5747 PTK2;MAPK1;FGFR1;HTR2B;PPARD geneontology_Biological_Process

GO:0006352 DNA-templated transcription, initiation http://amigo.geneontology.org/amigo/term/GO:0006352 223 6 1.20896993592903 4.96290256828373 0.00124411816341929 0.0341433293656168 5465;5467;5468;5914;6256;7067 PPARG;PPARA;RXRA;PPARD;RARA;THRA geneontology_Biological_Process

GO:0042447 hormone catabolic process http://amigo.geneontology.org/amigo/term/GO:0042447 10 2 0.0542138984721538 36.8909090909091 0.00126298743966791 0.0341433293656168 43;1636 ACE;ACHE geneontology_Biological_Process

GO:0042572 retinol metabolic process http://amigo.geneontology.org/amigo/term/GO:0042572 10 2 0.0542138984721538 36.8909090909091 0.00126298743966791 0.0341433293656168 5950;8644 AKR1C3;RBP4 geneontology_Biological_Process

GO:0030730 sequestering of triglyceride http://amigo.geneontology.org/amigo/term/GO:0030730 10 2 0.0542138984721538 36.8909090909091 0.00126298743966791 0.0341433293656168 5465;5468 PPARG;PPARA geneontology_Biological_Process

GO:0050872 white fat cell differentiation http://amigo.geneontology.org/amigo/term/GO:0050872 10 2 0.0542138984721538 36.8909090909091 0.00126298743966791 0.0341433293656168 2167;5468 PPARG;FABP4 geneontology_Biological_Process

GO:0010713 negative regulation of collagen metabolic process http://amigo.geneontology.org/amigo/term/GO:0010713 10 2 0.0542138984721538 36.8909090909091 0.00126298743966791 0.0341433293656168 5467;5468 PPARG;PPARD geneontology_Biological_Process

GO:0071396 cellular response to lipid http://amigo.geneontology.org/amigo/term/GO:0071396 497 9 2.69443075406604 3.34022315712457 0.00128172476645783 0.0343626497431017 834;5465;5467;5468;5594;5914;6256;7067;8644 PPARG;PPARA;AKR1C3;MAPK1;RXRA;CASP1;PPARD;RARA;THRA geneontology_Biological_Process

GO:0006939 smooth muscle contraction http://amigo.geneontology.org/amigo/term/GO:0006939 88 4 0.477082306554953 8.38429752066116 0.00128672665540641 0.0343626497431017 136;150;152;3357 ADORA2B;ADRA2A;ADRA2C;HTR2B geneontology_Biological_Process

GO:0048640 negative regulation of developmental growth http://amigo.geneontology.org/amigo/term/GO:0048640 88 4 0.477082306554953 8.38429752066116 0.00128672665540641 0.0343626497431017 5465;5641;5747;5950 PPARA;PTK2;LGMN;RBP4 geneontology_Biological_Process

GO:0050999 regulation of nitric-oxide synthase activity http://amigo.geneontology.org/amigo/term/GO:0050999 40 3 0.216855593888615 13.8340909090909 0.00129257155369578 0.0343626497431017 1268;3320;3357 CNR1;HSP90AA1;HTR2B geneontology_Biological_Process

GO:0060038 cardiac muscle cell proliferation http://amigo.geneontology.org/amigo/term/GO:0060038 40 3 0.216855593888615 13.8340909090909 0.00129257155369578 0.0343626497431017 2260;5950;6256 RXRA;FGFR1;RBP4 geneontology_Biological_Process

GO:0002274 myeloid leukocyte activation http://amigo.geneontology.org/amigo/term/GO:0002274 499 9 2.70527353376047 3.32683548916014 0.00131824768688849 0.034929198643979 136;240;1268;1508;2171;3320;5328;5594;23385 CNR1;ADORA2B;FABP5;ALOX5;NCSTN;CTSB;MAPK1;HSP90AA1;PLAU geneontology_Biological_Process

GO:0021761 limbic system development http://amigo.geneontology.org/amigo/term/GO:0021761 89 4 0.482503696402169 8.29009193054137 0.00134190122604405 0.0354385927749323 836;2260;2932;5914 CASP3;GSK3B;FGFR1;RARA geneontology_Biological_Process

GO:0030641 regulation of cellular pH http://amigo.geneontology.org/amigo/term/GO:0030641 41 3 0.22227698373583 13.4966740576497 0.00138929036696023 0.0365694128829465 596;760;5594 BCL2;MAPK1;CA2 geneontology_Biological_Process

GO:0001823 mesonephros development http://amigo.geneontology.org/amigo/term/GO:0001823 90 4 0.487925086249384 8.1979797979798 0.00139871090642851 0.0366966710598064 596;2260;5914;5972 REN;BCL2;FGFR1;RARA geneontology_Biological_Process

GO:0052548 regulation of endopeptidase activity http://amigo.geneontology.org/amigo/term/GO:0052548 313 7 1.69689502217841 4.12518152773744 0.00141585606926253 0.0370250989092771 834;836;841;4193;5468;5641;6774 CASP3;PPARG;STAT3;CASP8;CASP1;MDM2;LGMN geneontology_Biological_Process

GO:0006006 glucose metabolic process http://amigo.geneontology.org/amigo/term/GO:0006006 154 5 0.834894036471168 5.9887839433294 0.00142581646358275 0.037164115119183 2171;2932;5465;5467;5950 PPARA;GSK3B;FABP5;PPARD;RBP4 geneontology_Biological_Process

GO:0006839 mitochondrial transport http://amigo.geneontology.org/amigo/term/GO:0006839 155 5 0.840315426318384 5.95014662756598 0.00146732920941195 0.0381219751094624 596;841;2932;3320;6774 GSK3B;BCL2;STAT3;HSP90AA1;CASP8 geneontology_Biological_Process

GO:0043112 receptor metabolic process http://amigo.geneontology.org/amigo/term/GO:0043112 156 5 0.845736816165599 5.91200466200466 0.00150972835524588 0.0390965899633576 43;3357;5465;5468;5641 PPARG;PPARA;ACHE;HTR2B;LGMN geneontology_Biological_Process

GO:0010745 negative regulation of macrophage derived foam cell differentiation http://amigo.geneontology.org/amigo/term/GO:0010745 11 2 0.0596352883193692 33.5371900826446 0.00153828966568437 0.0392018914165806 5465;5468 PPARG;PPARA geneontology_Biological_Process

GO:0045820 negative regulation of glycolytic process http://amigo.geneontology.org/amigo/term/GO:0045820 11 2 0.0596352883193692 33.5371900826446 0.00153828966568437 0.0392018914165806 5465;6774 PPARA;STAT3 geneontology_Biological_Process

GO:0014842 regulation of skeletal muscle satellite cell proliferation http://amigo.geneontology.org/amigo/term/GO:0014842 11 2 0.0596352883193692 33.5371900826446 0.00153828966568437 0.0392018914165806 5467;6774 STAT3;PPARD geneontology_Biological_Process

GO:0071391 cellular response to estrogen stimulus http://amigo.geneontology.org/amigo/term/GO:0071391 11 2 0.0596352883193692 33.5371900826446 0.00153828966568437 0.0392018914165806 4193;5914 MDM2;RARA geneontology_Biological_Process

GO:0050665 hydrogen peroxide biosynthetic process http://amigo.geneontology.org/amigo/term/GO:0050665 11 2 0.0596352883193692 33.5371900826446 0.00153828966568437 0.0392018914165806 1544;6774 CYP1A2;STAT3 geneontology_Biological_Process

GO:0043299 leukocyte degranulation http://amigo.geneontology.org/amigo/term/GO:0043299 412 8 2.23361261705274 3.58164165931156 0.00157443879967123 0.0399957437300609 136;240;1508;2171;3320;5328;5594;23385 ADORA2B;FABP5;ALOX5;NCSTN;CTSB;MAPK1;HSP90AA1;PLAU geneontology_Biological_Process

GO:0001523 retinoid metabolic process http://amigo.geneontology.org/amigo/term/GO:0001523 43 3 0.233119763430261 12.8689217758985 0.0015961123923226 0.0400379039603933 5467;5950;8644 AKR1C3;PPARD;RBP4 geneontology_Biological_Process

GO:0051972 regulation of telomerase activity http://amigo.geneontology.org/amigo/term/GO:0051972 43 3 0.233119763430261 12.8689217758985 0.0015961123923226 0.0400379039603933 3320;5468;5594 PPARG;MAPK1;HSP90AA1 geneontology_Biological_Process

GO:0048662 negative regulation of smooth muscle cell proliferation http://amigo.geneontology.org/amigo/term/GO:0048662 43 3 0.233119763430261 12.8689217758985 0.0015961123923226 0.0400379039603933 5467;5468;5739 PPARG;PPARD;PTGIR geneontology_Biological_Process

GO:0002067 glandular epithelial cell differentiation http://amigo.geneontology.org/amigo/term/GO:0002067 43 3 0.233119763430261 12.8689217758985 0.0015961123923226 0.0400379039603933 2932;5914;6256 GSK3B;RXRA;RARA geneontology_Biological_Process

GO:0030099 myeloid cell differentiation http://amigo.geneontology.org/amigo/term/GO:0030099 322 7 1.74568753080335 4.0098814229249 0.00166627479284931 0.041667284038688 760;836;841;5468;5914;6774;7067 CASP3;PPARG;STAT3;CASP8;CA2;RARA;THRA geneontology_Biological_Process

GO:0042982 amyloid precursor protein metabolic process http://amigo.geneontology.org/amigo/term/GO:0042982 44 3 0.238541153277477 12.5764462809917 0.00170636296821725 0.042536811438238 43;836;23385 CASP3;ACHE;NCSTN geneontology_Biological_Process

GO:0002275 myeloid cell activation involved in immune response http://amigo.geneontology.org/amigo/term/GO:0002275 418 8 2.26614095613603 3.53023053501522 0.00172548208940859 0.0428798375138123 136;240;1508;2171;3320;5328;5594;23385 ADORA2B;FABP5;ALOX5;NCSTN;CTSB;MAPK1;HSP90AA1;PLAU geneontology_Biological_Process

GO:0016999 antibiotic metabolic process http://amigo.geneontology.org/amigo/term/GO:0016999 96 4 0.520453425332676 7.68560606060606 0.00177534652157096 0.0437930513638811 231;1544;6774;8644 AKR1B1;AKR1C3;CYP1A2;STAT3 geneontology_Biological_Process

GO:1903052 positive regulation of proteolysis involved in cellular protein catabolic process http://amigo.geneontology.org/amigo/term/GO:1903052 96 4 0.520453425332676 7.68560606060606 0.00177534652157096 0.0437930513638811 2932;4193;5747;9817 GSK3B;KEAP1;PTK2;MDM2 geneontology_Biological_Process

GO:0015909 long-chain fatty acid transport http://amigo.geneontology.org/amigo/term/GO:0015909 45 3 0.243962543124692 12.2969696969697 0.00182126897383517 0.0437930513638811 1636;2167;5468 ACE;PPARG;FABP4 geneontology_Biological_Process

GO:0031638 zymogen activation http://amigo.geneontology.org/amigo/term/GO:0031638 45 3 0.243962543124692 12.2969696969697 0.00182126897383517 0.0437930513638811 841;5328;5641 CASP8;PLAU;LGMN geneontology_Biological_Process

GO:0001973 adenosine receptor signaling pathway http://amigo.geneontology.org/amigo/term/GO:0001973 12 2 0.0650566781665845 30.7424242424242 0.00183953832509998 0.0437930513638811 136;140 ADORA2B;ADORA3 geneontology_Biological_Process

GO:0030812 negative regulation of nucleotide catabolic process http://amigo.geneontology.org/amigo/term/GO:0030812 12 2 0.0650566781665845 30.7424242424242 0.00183953832509998 0.0437930513638811 5465;6774 PPARA;STAT3 geneontology_Biological_Process

GO:0051198 negative regulation of coenzyme metabolic process http://amigo.geneontology.org/amigo/term/GO:0051198 12 2 0.0650566781665845 30.7424242424242 0.00183953832509998 0.0437930513638811 5465;6774 PPARA;STAT3 geneontology_Biological_Process

GO:1901661 quinone metabolic process http://amigo.geneontology.org/amigo/term/GO:1901661 12 2 0.0650566781665845 30.7424242424242 0.00183953832509998 0.0437930513638811 231;8644 AKR1B1;AKR1C3 geneontology_Biological_Process

GO:0045986 negative regulation of smooth muscle contraction http://amigo.geneontology.org/amigo/term/GO:0045986 12 2 0.0650566781665845 30.7424242424242 0.00183953832509998 0.0437930513638811 136;152 ADORA2B;ADRA2C geneontology_Biological_Process

GO:0046321 positive regulation of fatty acid oxidation http://amigo.geneontology.org/amigo/term/GO:0046321 12 2 0.0650566781665845 30.7424242424242 0.00183953832509998 0.0437930513638811 5465;5468 PPARG;PPARA geneontology_Biological_Process

GO:0016137 glycoside metabolic process http://amigo.geneontology.org/amigo/term/GO:0016137 12 2 0.0650566781665845 30.7424242424242 0.00183953832509998 0.0437930513638811 231;8644 AKR1B1;AKR1C3 geneontology_Biological_Process

GO:0034505 tooth mineralization http://amigo.geneontology.org/amigo/term/GO:0034505 12 2 0.0650566781665845 30.7424242424242 0.00183953832509998 0.0437930513638811 249;5465 PPARA;ALPL geneontology_Biological_Process

GO:0014841 skeletal muscle satellite cell proliferation http://amigo.geneontology.org/amigo/term/GO:0014841 12 2 0.0650566781665845 30.7424242424242 0.00183953832509998 0.0437930513638811 5467;6774 STAT3;PPARD geneontology_Biological_Process

GO:0014857 regulation of skeletal muscle cell proliferation http://amigo.geneontology.org/amigo/term/GO:0014857 12 2 0.0650566781665845 30.7424242424242 0.00183953832509998 0.0437930513638811 5467;6774 STAT3;PPARD geneontology_Biological_Process

GO:0001952 regulation of cell-matrix adhesion http://amigo.geneontology.org/amigo/term/GO:0001952 97 4 0.525874815179892 7.60637300843486 0.00184432120840139 0.0437930513638811 596;2932;5328;5747 GSK3B;PTK2;BCL2;PLAU geneontology_Biological_Process

GO:1901215 negative regulation of neuron death http://amigo.geneontology.org/amigo/term/GO:1901215 164 5 0.889107934943322 5.62361419068736 0.00188222480228739 0.0443400429741315 596;2932;5465;5641;6774 PPARA;GSK3B;BCL2;STAT3;LGMN geneontology_Biological_Process

GO:0030073 insulin secretion http://amigo.geneontology.org/amigo/term/GO:0030073 164 5 0.889107934943322 5.62361419068736 0.00188222480228739 0.0443400429741315 150;152;1268;5467;5950 CNR1;ADRA2A;ADRA2C;PPARD;RBP4 geneontology_Biological_Process

GO:0060249 anatomical structure homeostasis http://amigo.geneontology.org/amigo/term/GO:0060249 329 7 1.78363725973386 3.92456479690522 0.00188398083119279 0.0443400429741315 231;596;760;1268;3320;5594;5950 AKR1B1;CNR1;BCL2;MAPK1;HSP90AA1;CA2;RBP4 geneontology_Biological_Process

GO:0043279 response to alkaloid http://amigo.geneontology.org/amigo/term/GO:0043279 98 4 0.531296205027107 7.52875695732839 0.00191513595145554 0.0448097014138807 836;1268;4193;5468 CASP3;PPARG;CNR1;MDM2 geneontology_Biological_Process

GO:1902652 secondary alcohol metabolic process http://amigo.geneontology.org/amigo/term/GO:1902652 98 4 0.531296205027107 7.52875695732839 0.00191513595145554 0.0448097014138807 2260;5467;6256;6319 RXRA;FGFR1;PPARD;SCD geneontology_Biological_Process

GO:0097345 mitochondrial outer membrane permeabilization http://amigo.geneontology.org/amigo/term/GO:0097345 46 3 0.249383932971907 12.0296442687747 0.0019409003334947 0.0452801296461358 596;841;2932 GSK3B;BCL2;CASP8 geneontology_Biological_Process

GO:0052547 regulation of peptidase activity http://amigo.geneontology.org/amigo/term/GO:0052547 332 7 1.79990142927551 3.88910186199343 0.0019838074163322 0.0460411568651204 834;836;841;4193;5468;5641;6774 CASP3;PPARG;STAT3;CASP8;CASP1;MDM2;LGMN geneontology_Biological_Process

GO:0090276 regulation of peptide hormone secretion http://amigo.geneontology.org/amigo/term/GO:0090276 166 5 0.899950714637753 5.55585980284775 0.00198502863265015 0.0460411568651204 150;152;1268;5467;5950 CNR1;ADRA2A;ADRA2C;PPARD;RBP4 geneontology_Biological_Process

GO:0006091 generation of precursor metabolites and energy http://amigo.geneontology.org/amigo/term/GO:0006091 333 7 1.80532281912272 3.87742287742288 0.0020179827920892 0.046670226307219 231;1544;2932;3939;5465;5467;6774 AKR1B1;PPARA;GSK3B;CYP1A2;STAT3;LDHA;PPARD geneontology_Biological_Process

GO:0009306 protein secretion http://amigo.geneontology.org/amigo/term/GO:0009306 430 8 2.33119763430261 3.43171247357294 0.00206211793086597 0.0473545405036965 43;150;152;834;1268;3357;5467;5950 ACHE;CNR1;ADRA2A;ADRA2C;CASP1;HTR2B;PPARD;RBP4 geneontology_Biological_Process

GO:0007189 adenylate cyclase-activating G protein-coupled receptor signaling pathway http://amigo.geneontology.org/amigo/term/GO:0007189 100 4 0.542138984721538 7.37818181818182 0.00206238706678774 0.0473545405036965 136;150;152;5739 ADORA2B;ADRA2A;ADRA2C;PTGIR geneontology_Biological_Process

GO:1902110 positive regulation of mitochondrial membrane permeability involved in apoptotic process http://amigo.geneontology.org/amigo/term/GO:1902110 47 3 0.254805322819123 11.7736943907157 0.00206532549809924 0.0473545405036965 596;841;2932 GSK3B;BCL2;CASP8 geneontology_Biological_Process

GO:0009612 response to mechanical stimulus http://amigo.geneontology.org/amigo/term/GO:0009612 168 5 0.910793494332183 5.48971861471861 0.0020918928195448 0.0478266466914215 834;841;5468;9020;23621 PPARG;BACE1;MAP3K14;CASP8;CASP1 geneontology_Biological_Process

GO:0001819 positive regulation of cytokine production http://amigo.geneontology.org/amigo/term/GO:0001819 336 7 1.82158698866437 3.84280303030303 0.00212326648992711 0.0484056366165149 136;150;834;3357;5914;6774;9261 ADORA2B;ADRA2A;MAPKAPK2;STAT3;CASP1;HTR2B;RARA geneontology_Biological_Process

GO:0048762 mesenchymal cell differentiation http://amigo.geneontology.org/amigo/term/GO:0048762 169 5 0.916214884179399 5.45723507261969 0.00214687718120354 0.0485601330263806 596;2260;2932;3357;5594 GSK3B;BCL2;MAPK1;FGFR1;HTR2B geneontology_Biological_Process

GO:2001170 negative regulation of ATP biosynthetic process http://amigo.geneontology.org/amigo/term/GO:2001170 13 2 0.0704780680137999 28.3776223776224 0.00216645432272156 0.0485601330263806 5465;6774 PPARA;STAT3 geneontology_Biological_Process

GO:0014856 skeletal muscle cell proliferation http://amigo.geneontology.org/amigo/term/GO:0014856 13 2 0.0704780680137999 28.3776223776224 0.00216645432272156 0.0485601330263806 5467;6774 STAT3;PPARD geneontology_Biological_Process

GO:0097202 activation of cysteine-type endopeptidase activity http://amigo.geneontology.org/amigo/term/GO:0097202 13 2 0.0704780680137999 28.3776223776224 0.00216645432272156 0.0485601330263806 841;5641 CASP8;LGMN geneontology_Biological_Process

GO:1902894 negative regulation of pri-miRNA transcription by RNA polymerase II http://amigo.geneontology.org/amigo/term/GO:1902894 13 2 0.0704780680137999 28.3776223776224 0.00216645432272156 0.0485601330263806 5465;5467 PPARA;PPARD geneontology_Biological_Process

GO:0017000 antibiotic biosynthetic process http://amigo.geneontology.org/amigo/term/GO:0017000 13 2 0.0704780680137999 28.3776223776224 0.00216645432272156 0.0485601330263806 1544;6774 CYP1A2;STAT3 geneontology_Biological_Process

GO:0016101 diterpenoid metabolic process http://amigo.geneontology.org/amigo/term/GO:0016101 48 3 0.260226712666338 11.5284090909091 0.00219461146137023 0.0486724509567091 5467;5950;8644 AKR1C3;PPARD;RBP4 geneontology_Biological_Process

GO:0019229 regulation of vasoconstriction http://amigo.geneontology.org/amigo/term/GO:0019229 48 3 0.260226712666338 11.5284090909091 0.00219461146137023 0.0486724509567091 150;152;1636 ACE;ADRA2A;ADRA2C geneontology_Biological_Process

GO:0006885 regulation of pH http://amigo.geneontology.org/amigo/term/GO:0006885 48 3 0.260226712666338 11.5284090909091 0.00219461146137023 0.0486724509567091 596;760;5594 BCL2;MAPK1;CA2 geneontology_Biological_Process

GO:0009896 positive regulation of catabolic process http://amigo.geneontology.org/amigo/term/GO:0009896 338 7 1.8324297683588 3.82006455083378 0.00219579540057135 0.0486724509567091 150;2932;3320;4193;5465;5747;9817 PPARA;GSK3B;ADRA2A;KEAP1;PTK2;HSP90AA1;MDM2 geneontology_Biological_Process

GO:0006635 fatty acid beta-oxidation http://amigo.geneontology.org/amigo/term/GO:0006635 49 3 0.265648102513553 11.2931354359926 0.00232882377594223 0.0510554735755884 1268;5465;5467 PPARA;CNR1;PPARD geneontology_Biological_Process

GO:0032768 regulation of monooxygenase activity http://amigo.geneontology.org/amigo/term/GO:0032768 49 3 0.265648102513553 11.2931354359926 0.00232882377594223 0.0510554735755884 1268;3320;3357 CNR1;HSP90AA1;HTR2B geneontology_Biological_Process

GO:1902686 mitochondrial outer membrane permeabilization involved in programmed cell death http://amigo.geneontology.org/amigo/term/GO:1902686 49 3 0.265648102513553 11.2931354359926 0.00232882377594223 0.0510554735755884 596;841;2932 GSK3B;BCL2;CASP8 geneontology_Biological_Process

GO:0055021 regulation of cardiac muscle tissue growth http://amigo.geneontology.org/amigo/term/GO:0055021 49 3 0.265648102513553 11.2931354359926 0.00232882377594223 0.0510554735755884 2260;5465;5950 PPARA;FGFR1;RBP4 geneontology_Biological_Process

GO:0098693 regulation of synaptic vesicle cycle http://amigo.geneontology.org/amigo/term/GO:0098693 104 4 0.563824544110399 7.09440559440559 0.00238008214985563 0.0520366594621442 136;1268;2932;23621 BACE1;GSK3B;CNR1;ADORA2B geneontology_Biological_Process

GO:0032102 negative regulation of response to external stimulus http://amigo.geneontology.org/amigo/term/GO:0032102 254 6 1.37703302119271 4.35719398711525 0.00240950465310408 0.0525363930085527 239;5328;5465;5467;5468;23621 PPARG;BACE1;PPARA;ALOX12;PLAU;PPARD geneontology_Biological_Process

GO:0007188 adenylate cyclase-modulating G protein-coupled receptor signaling pathway http://amigo.geneontology.org/amigo/term/GO:0007188 174 5 0.943321833415476 5.30041797283177 0.00243779771926034 0.0526243430143117 136;150;152;1268;5739 CNR1;ADORA2B;ADRA2A;ADRA2C;PTGIR geneontology_Biological_Process

GO:0048738 cardiac muscle tissue development http://amigo.geneontology.org/amigo/term/GO:0048738 174 5 0.943321833415476 5.30041797283177 0.00243779771926034 0.0526243430143117 2260;5465;5914;5950;6256 PPARA;RXRA;FGFR1;RBP4;RARA geneontology_Biological_Process

GO:0032388 positive regulation of intracellular transport http://amigo.geneontology.org/amigo/term/GO:0032388 174 5 0.943321833415476 5.30041797283177 0.00243779771926034 0.0526243430143117 136;1268;2932;4193;5594 GSK3B;CNR1;ADORA2B;MAPK1;MDM2 geneontology_Biological_Process

GO:0045834 positive regulation of lipid metabolic process http://amigo.geneontology.org/amigo/term/GO:0045834 105 4 0.569245933957615 7.02683982683983 0.00246448403169297 0.0526243430143117 3357;5465;5468;5747 PPARG;PPARA;PTK2;HTR2B geneontology_Biological_Process

GO:0014066 regulation of phosphatidylinositol 3-kinase signaling http://amigo.geneontology.org/amigo/term/GO:0014066 105 4 0.569245933957615 7.02683982683983 0.00246448403169297 0.0526243430143117 2260;5467;5594;5747 PTK2;MAPK1;FGFR1;PPARD geneontology_Biological_Process

GO:0019915 lipid storage http://amigo.geneontology.org/amigo/term/GO:0019915 50 3 0.271069492360769 11.0672727272727 0.00246802656931844 0.0526243430143117 5465;5467;5468 PPARG;PPARA;PPARD geneontology_Biological_Process

GO:1902108 regulation of mitochondrial membrane permeability involved in apoptotic process http://amigo.geneontology.org/amigo/term/GO:1902108 50 3 0.271069492360769 11.0672727272727 0.00246802656931844 0.0526243430143117 596;841;2932 GSK3B;BCL2;CASP8 geneontology_Biological_Process

GO:0051188 cofactor biosynthetic process http://amigo.geneontology.org/amigo/term/GO:0051188 175 5 0.948743223262691 5.27012987012987 0.00249926514182797 0.0526243430143117 1544;3939;5465;6319;6774 PPARA;CYP1A2;STAT3;LDHA;SCD geneontology_Biological_Process

GO:0010721 negative regulation of cell development http://amigo.geneontology.org/amigo/term/GO:0010721 256 6 1.38787580088714 4.32315340909091 0.0025059633650516 0.0526243430143117 596;2932;4193;5465;5747;6774 PPARA;GSK3B;PTK2;BCL2;STAT3;MDM2 geneontology_Biological_Process

GO:0042060 wound healing http://amigo.geneontology.org/amigo/term/GO:0042060 444 8 2.40709709216363 3.32350532350532 0.00251852214249249 0.0526243430143117 150;152;239;836;5328;5465;5467;5594 CASP3;PPARA;ALOX12;ADRA2A;ADRA2C;MAPK1;PLAU;PPARD geneontology_Biological_Process

GO:0071900 regulation of protein serine/threonine kinase activity http://amigo.geneontology.org/amigo/term/GO:0071900 444 8 2.40709709216363 3.32350532350532 0.00251852214249249 0.0526243430143117 136;150;836;2260;3357;5594;9020;9261 CASP3;ADORA2B;MAP3K14;ADRA2A;MAPKAPK2;MAPK1;FGFR1;HTR2B geneontology_Biological_Process

GO:0001991 regulation of systemic arterial blood pressure by circulatory renin-angiotensin http://amigo.geneontology.org/amigo/term/GO:0001991 14 2 0.0758994578610153 26.3506493506494 0.00251876073162727 0.0526243430143117 1636;5972 ACE;REN geneontology_Biological_Process

GO:1902644 tertiary alcohol metabolic process http://amigo.geneontology.org/amigo/term/GO:1902644 14 2 0.0758994578610153 26.3506493506494 0.00251876073162727 0.0526243430143117 231;8644 AKR1B1;AKR1C3 geneontology_Biological_Process

GO:0006817 phosphate ion transport http://amigo.geneontology.org/amigo/term/GO:0006817 14 2 0.0758994578610153 26.3506493506494 0.00251876073162727 0.0526243430143117 2260;6098 FGFR1;ROS1 geneontology_Biological_Process

GO:1905564 positive regulation of vascular endothelial cell proliferation http://amigo.geneontology.org/amigo/term/GO:1905564 14 2 0.0758994578610153 26.3506493506494 0.00251876073162727 0.0526243430143117 2260;6774 STAT3;FGFR1 geneontology_Biological_Process

GO:2001028 positive regulation of endothelial cell chemotaxis http://amigo.geneontology.org/amigo/term/GO:2001028 14 2 0.0758994578610153 26.3506493506494 0.00251876073162727 0.0526243430143117 2260;5641 FGFR1;LGMN geneontology_Biological_Process

GO:0006940 regulation of smooth muscle contraction http://amigo.geneontology.org/amigo/term/GO:0006940 51 3 0.276490882207984 10.8502673796791 0.00261228255968871 0.0540173012901057 136;150;152 ADORA2B;ADRA2A;ADRA2C geneontology_Biological_Process

GO:0033619 membrane protein proteolysis http://amigo.geneontology.org/amigo/term/GO:0033619 51 3 0.276490882207984 10.8502673796791 0.00261228255968871 0.0540173012901057 150;23385;23621 BACE1;ADRA2A;NCSTN geneontology_Biological_Process

GO:0035794 positive regulation of mitochondrial membrane permeability http://amigo.geneontology.org/amigo/term/GO:0035794 51 3 0.276490882207984 10.8502673796791 0.00261228255968871 0.0540173012901057 596;841;2932 GSK3B;BCL2;CASP8 geneontology_Biological_Process

GO:0008406 gonad development http://amigo.geneontology.org/amigo/term/GO:0008406 177 5 0.959586002957122 5.2105803800719 0.00262557906423477 0.0540173012901057 596;836;5914;5972;8644 CASP3;REN;AKR1C3;BCL2;RARA geneontology_Biological_Process

GO:1905952 regulation of lipid localization http://amigo.geneontology.org/amigo/term/GO:1905952 107 4 0.580088713652045 6.89549702633815 0.00263943574161851 0.0540173012901057 5465;5467;5468;5972 REN;PPARG;PPARA;PPARD geneontology_Biological_Process

GO:0003206 cardiac chamber morphogenesis http://amigo.geneontology.org/amigo/term/GO:0003206 107 4 0.580088713652045 6.89549702633815 0.00263943574161851 0.0540173012901057 4193;5914;5950;6256 RXRA;MDM2;RBP4;RARA geneontology_Biological_Process

GO:0001936 regulation of endothelial cell proliferation http://amigo.geneontology.org/amigo/term/GO:0001936 107 4 0.580088713652045 6.89549702633815 0.00263943574161851 0.0540173012901057 2260;3357;5468;6774 PPARG;STAT3;FGFR1;HTR2B geneontology_Biological_Process

GO:0016079 synaptic vesicle exocytosis http://amigo.geneontology.org/amigo/term/GO:0016079 107 4 0.580088713652045 6.89549702633815 0.00263943574161851 0.0540173012901057 136;1268;2932;23621 BACE1;GSK3B;CNR1;ADORA2B geneontology_Biological_Process

GO:0019932 second-messenger-mediated signaling http://amigo.geneontology.org/amigo/term/GO:0019932 350 7 1.89748644652538 3.68909090909091 0.00267229952261949 0.0545503591326561 136;150;152;2932;3357;5141;5739 GSK3B;ADORA2B;PDE4A;ADRA2A;ADRA2C;HTR2B;PTGIR geneontology_Biological_Process

GO:0006164 purine nucleotide biosynthetic process http://amigo.geneontology.org/amigo/term/GO:0006164 178 5 0.965007392804337 5.18130745658836 0.00269044955865505 0.0547811128965845 136;3939;5465;6319;6774 PPARA;ADORA2B;STAT3;LDHA;SCD geneontology_Biological_Process

GO:0009166 nucleotide catabolic process http://amigo.geneontology.org/amigo/term/GO:0009166 108 4 0.585510103499261 6.83164983164983 0.00273003498575075 0.0554460404974048 3939;5141;5465;6774 PPARA;PDE4A;STAT3;LDHA geneontology_Biological_Process

GO:0046686 response to cadmium ion http://amigo.geneontology.org/amigo/term/GO:0046686 52 3 0.2819122720552 10.6416083916084 0.0027616530716118 0.0558049188864587 1544;5594;8644 AKR1C3;CYP1A2;MAPK1 geneontology_Biological_Process

GO:1905710 positive regulation of membrane permeability http://amigo.geneontology.org/amigo/term/GO:1905710 52 3 0.2819122720552 10.6416083916084 0.0027616530716118 0.0558049188864587 596;841;2932 GSK3B;BCL2;CASP8 geneontology_Biological_Process

GO:0018108 peptidyl-tyrosine phosphorylation http://amigo.geneontology.org/amigo/term/GO:0018108 353 7 1.91375061606703 3.65773886170487 0.00280304728780789 0.0564690992251185 150;1636;2260;3320;5747;6098;6774 ACE;ADRA2A;PTK2;STAT3;HSP90AA1;FGFR1;ROS1 geneontology_Biological_Process

GO:0061041 regulation of wound healing http://amigo.geneontology.org/amigo/term/GO:0061041 109 4 0.590931493346476 6.76897414512093 0.00282274927393744 0.0564690992251185 150;239;5328;5467 ALOX12;ADRA2A;PLAU;PPARD geneontology_Biological_Process

GO:0016202 regulation of striated muscle tissue development http://amigo.geneontology.org/amigo/term/GO:0016202 109 4 0.590931493346476 6.76897414512093 0.00282274927393744 0.0564690992251185 596;2260;5465;5950 PPARA;BCL2;FGFR1;RBP4 geneontology_Biological_Process

GO:0043280 positive regulation of cysteine-type endopeptidase activity involved in apoptotic process http://amigo.geneontology.org/amigo/term/GO:0043280 109 4 0.590931493346476 6.76897414512093 0.00282274927393744 0.0564690992251185 834;836;841;5468 CASP3;PPARG;CASP8;CASP1 geneontology_Biological_Process

GO:0008654 phospholipid biosynthetic process http://amigo.geneontology.org/amigo/term/GO:0008654 181 5 0.981271562345983 5.09542943244601 0.00289205934024761 0.0569416574789288 43;2171;2260;3357;5467 ACHE;FABP5;FGFR1;HTR2B;PPARD geneontology_Biological_Process

GO:0018212 peptidyl-tyrosine modification http://amigo.geneontology.org/amigo/term/GO:0018212 355 7 1.92459339576146 3.6371318822023 0.00289291940416792 0.0569416574789288 150;1636;2260;3320;5747;6098;6774 ACE;ADRA2A;PTK2;STAT3;HSP90AA1;FGFR1;ROS1 geneontology_Biological_Process

GO:0031998 regulation of fatty acid beta-oxidation http://amigo.geneontology.org/amigo/term/GO:0031998 15 2 0.0813208477082307 24.5939393939394 0.00289618277854586 0.0569416574789288 1268;5465 PPARA;CNR1 geneontology_Biological_Process

GO:0030809 negative regulation of nucleotide biosynthetic process http://amigo.geneontology.org/amigo/term/GO:0030809 15 2 0.0813208477082307 24.5939393939394 0.00289618277854586 0.0569416574789288 5465;6774 PPARA;STAT3 geneontology_Biological_Process

GO:1900372 negative regulation of purine nucleotide biosynthetic process http://amigo.geneontology.org/amigo/term/GO:1900372 15 2 0.0813208477082307 24.5939393939394 0.00289618277854586 0.0569416574789288 5465;6774 PPARA;STAT3 geneontology_Biological_Process

GO:2000811 negative regulation of anoikis http://amigo.geneontology.org/amigo/term/GO:2000811 15 2 0.0813208477082307 24.5939393939394 0.00289618277854586 0.0569416574789288 596;5747 PTK2;BCL2 geneontology_Biological_Process

GO:0055012 ventricular cardiac muscle cell differentiation http://amigo.geneontology.org/amigo/term/GO:0055012 15 2 0.0813208477082307 24.5939393939394 0.00289618277854586 0.0569416574789288 5914;6256 RXRA;RARA geneontology_Biological_Process

GO:0050433 regulation of catecholamine secretion http://amigo.geneontology.org/amigo/term/GO:0050433 53 3 0.287333661902415 10.4408233276158 0.00291619805156107 0.0569430726067682 150;152;1268 CNR1;ADRA2A;ADRA2C geneontology_Biological_Process

GO:0045833 negative regulation of lipid metabolic process http://amigo.geneontology.org/amigo/term/GO:0045833 53 3 0.287333661902415 10.4408233276158 0.00291619805156107 0.0569430726067682 150;1268;8644 CNR1;ADRA2A;AKR1C3 geneontology_Biological_Process

GO:0062013 positive regulation of small molecule metabolic process http://amigo.geneontology.org/amigo/term/GO:0062013 110 4 0.596352883193692 6.70743801652893 0.00291760307032929 0.0569430726067682 136;5465;5468;6774 PPARG;PPARA;ADORA2B;STAT3 geneontology_Biological_Process

GO:0031669 cellular response to nutrient levels http://amigo.geneontology.org/amigo/term/GO:0031669 182 5 0.986692952193199 5.06743256743257 0.00296163545791428 0.0575218614908497 596;4193;5468;5594;8644 PPARG;AKR1C3;BCL2;MAPK1;MDM2 geneontology_Biological_Process

GO:0045137 development of primary sexual characteristics http://amigo.geneontology.org/amigo/term/GO:0045137 182 5 0.986692952193199 5.06743256743257 0.00296163545791428 0.0575218614908497 596;836;5914;5972;8644 CASP3;REN;AKR1C3;BCL2;RARA geneontology_Biological_Process

GO:0001654 eye development http://amigo.geneontology.org/amigo/term/GO:0001654 265 6 1.43666830951207 4.17632933104631 0.00297657572563681 0.0576720555848565 43;596;5914;5950;6256;6774 ACHE;BCL2;STAT3;RXRA;RBP4;RARA geneontology_Biological_Process

GO:1901861 regulation of muscle tissue development http://amigo.geneontology.org/amigo/term/GO:1901861 111 4 0.601774273040907 6.64701064701065 0.00301462073592906 0.0582681041760975 596;2260;5465;5950 PPARA;BCL2;FGFR1;RBP4 geneontology_Biological_Process

GO:0150063 visual system development http://amigo.geneontology.org/amigo/term/GO:0150063 266 6 1.44208969935929 4.16062884483937 0.00303269565368458 0.058476218363335 43;596;5914;5950;6256;6774 ACHE;BCL2;STAT3;RXRA;RBP4;RARA geneontology_Biological_Process

GO:0002790 peptide secretion http://amigo.geneontology.org/amigo/term/GO:0002790 458 8 2.48299655002464 3.22191345772132 0.00305114336024004 0.0586905028092327 43;150;152;834;1268;3357;5467;5950 ACHE;CNR1;ADRA2A;ADRA2C;CASP1;HTR2B;PPARD;RBP4 geneontology_Biological_Process

GO:0006641 triglyceride metabolic process http://amigo.geneontology.org/amigo/term/GO:0006641 54 3 0.29275505174963 10.2474747474747 0.00307597608333676 0.0590048570815981 2167;2171;11343 MGLL;FABP5;FABP4 geneontology_Biological_Process

GO:0071214 cellular response to abiotic stimulus http://amigo.geneontology.org/amigo/term/GO:0071214 267 6 1.44751108920651 4.14504596527068 0.00308960698790173 0.0590048570815981 231;834;836;841;4193;9020 CASP3;AKR1B1;MAP3K14;CASP8;CASP1;MDM2 geneontology_Biological_Process

GO:0104004 cellular response to environmental stimulus http://amigo.geneontology.org/amigo/term/GO:0104004 267 6 1.44751108920651 4.14504596527068 0.00308960698790173 0.0590048570815981 231;834;836;841;4193;9020 CASP3;AKR1B1;MAP3K14;CASP8;CASP1;MDM2 geneontology_Biological_Process

GO:0017157 regulation of exocytosis http://amigo.geneontology.org/amigo/term/GO:0017157 184 5 0.997535731887629 5.01235177865613 0.0031044192039299 0.0590060866267151 136;150;1268;2932;23621 BACE1;GSK3B;CNR1;ADORA2B;ADRA2A geneontology_Biological_Process

GO:0043281 regulation of cysteine-type endopeptidase activity involved in apoptotic process http://amigo.geneontology.org/amigo/term/GO:0043281 184 5 0.997535731887629 5.01235177865613 0.0031044192039299 0.0590060866267151 834;836;841;4193;5468 CASP3;PPARG;CASP8;CASP1;MDM2 geneontology_Biological_Process

GO:0048634 regulation of muscle organ development http://amigo.geneontology.org/amigo/term/GO:0048634 112 4 0.607195662888122 6.58766233766234 0.00311382652557834 0.0590446442125069 596;2260;5465;5950 PPARA;BCL2;FGFR1;RBP4 geneontology_Biological_Process

GO:0072522 purine-containing compound biosynthetic process http://amigo.geneontology.org/amigo/term/GO:0072522 185 5 1.00295712173484 4.98525798525799 0.00317765092176758 0.0601124413143835 136;3939;5465;6319;6774 PPARA;ADORA2B;STAT3;LDHA;SCD geneontology_Biological_Process

GO:0048880 sensory system development http://amigo.geneontology.org/amigo/term/GO:0048880 269 6 1.45835386890094 4.11422777965529 0.003205831851117 0.0605025152656563 43;596;5914;5950;6256;6774 ACHE;BCL2;STAT3;RXRA;RBP4;RARA geneontology_Biological_Process

GO:1901292 nucleoside phosphate catabolic process http://amigo.geneontology.org/amigo/term/GO:1901292 113 4 0.612617052735338 6.52936444086887 0.00321524458500522 0.0605373815746159 3939;5141;5465;6774 PPARA;PDE4A;STAT3;LDHA geneontology_Biological_Process

GO:0050432 catecholamine secretion http://amigo.geneontology.org/amigo/term/GO:0050432 55 3 0.298176441596846 10.0611570247934 0.00324104440334338 0.0607373239240135 150;152;1268 CNR1;ADRA2A;ADRA2C geneontology_Biological_Process

GO:0060420 regulation of heart growth http://amigo.geneontology.org/amigo/term/GO:0060420 55 3 0.298176441596846 10.0611570247934 0.00324104440334338 0.0607373239240135 2260;5465;5950 PPARA;FGFR1;RBP4 geneontology_Biological_Process

GO:0090596 sensory organ morphogenesis http://amigo.geneontology.org/amigo/term/GO:0090596 186 5 1.00837851158206 4.95845552297165 0.00325212526660446 0.0608025850078711 596;2260;5594;5950;6774 BCL2;MAPK1;STAT3;FGFR1;RBP4 geneontology_Biological_Process

GO:0006656 phosphatidylcholine biosynthetic process http://amigo.geneontology.org/amigo/term/GO:0006656 16 2 0.086742237555446 23.0568181818182 0.00329844782932831 0.0609565347119749 43;2171 ACHE;FABP5 geneontology_Biological_Process

GO:0051195 negative regulation of cofactor metabolic process http://amigo.geneontology.org/amigo/term/GO:0051195 16 2 0.086742237555446 23.0568181818182 0.00329844782932831 0.0609565347119749 5465;6774 PPARA;STAT3 geneontology_Biological_Process

GO:0014061 regulation of norepinephrine secretion http://amigo.geneontology.org/amigo/term/GO:0014061 16 2 0.086742237555446 23.0568181818182 0.00329844782932831 0.0609565347119749 150;152 ADRA2A;ADRA2C geneontology_Biological_Process

GO:0060438 trachea development http://amigo.geneontology.org/amigo/term/GO:0060438 16 2 0.086742237555446 23.0568181818182 0.00329844782932831 0.0609565347119749 5594;5914 MAPK1;RARA geneontology_Biological_Process

GO:0046697 decidualization http://amigo.geneontology.org/amigo/term/GO:0046697 16 2 0.086742237555446 23.0568181818182 0.00329844782932831 0.0609565347119749 1508;5467 CTSB;PPARD geneontology_Biological_Process

GO:0001505 regulation of neurotransmitter levels http://amigo.geneontology.org/amigo/term/GO:0001505 271 6 1.46919664859537 4.08386447500839 0.00332530640072104 0.0613112945128336 43;136;1268;2932;3320;23621 BACE1;ACHE;GSK3B;CNR1;ADORA2B;HSP90AA1 geneontology_Biological_Process

GO:0050795 regulation of behavior http://amigo.geneontology.org/amigo/term/GO:0050795 56 3 0.303597831444061 9.88149350649351 0.00341145891573658 0.0622849043024556 1268;3357;6774 CNR1;STAT3;HTR2B geneontology_Biological_Process

GO:0097194 execution phase of apoptosis http://amigo.geneontology.org/amigo/term/GO:0097194 56 3 0.303597831444061 9.88149350649351 0.00341145891573658 0.0622849043024556 834;836;841 CASP3;CASP8;CASP1 geneontology_Biological_Process

GO:0097164 ammonium ion metabolic process http://amigo.geneontology.org/amigo/term/GO:0097164 115 4 0.623459832429768 6.41581027667984 0.00342481353325175 0.0622849043024556 43;231;2171;8644 AKR1B1;ACHE;FABP5;AKR1C3 geneontology_Biological_Process

GO:0060359 response to ammonium ion http://amigo.geneontology.org/amigo/term/GO:0060359 115 4 0.623459832429768 6.41581027667984 0.00342481353325175 0.0622849043024556 836;1268;4193;5594 CASP3;CNR1;MAPK1;MDM2 geneontology_Biological_Process

GO:0008584 male gonad development http://amigo.geneontology.org/amigo/term/GO:0008584 115 4 0.623459832429768 6.41581027667984 0.00342481353325175 0.0622849043024556 596;5914;5972;8644 REN;AKR1C3;BCL2;RARA geneontology_Biological_Process

GO:0046546 development of primary male sexual characteristics http://amigo.geneontology.org/amigo/term/GO:0046546 115 4 0.623459832429768 6.41581027667984 0.00342481353325175 0.0622849043024556 596;5914;5972;8644 REN;AKR1C3;BCL2;RARA geneontology_Biological_Process

GO:0071216 cellular response to biotic stimulus http://amigo.geneontology.org/amigo/term/GO:0071216 190 5 1.03006407097092 4.85406698564593 0.0035626898970067 0.0646454525075909 834;2932;5467;5594;5914 GSK3B;MAPK1;CASP1;PPARD;RARA geneontology_Biological_Process

GO:0030004 cellular monovalent inorganic cation homeostasis http://amigo.geneontology.org/amigo/term/GO:0030004 57 3 0.309019221291277 9.70813397129186 0.00358727420743632 0.0649442719635869 596;760;5594 BCL2;MAPK1;CA2 geneontology_Biological_Process

GO:0030336 negative regulation of cell migration http://amigo.geneontology.org/amigo/term/GO:0030336 191 5 1.03548546081814 4.8286530223703 0.00364355810179973 0.0658143384437955 140;596;5467;5468;6774 PPARG;ADORA3;BCL2;STAT3;PPARD geneontology_Biological_Process

GO:0043312 neutrophil degranulation http://amigo.geneontology.org/amigo/term/GO:0043312 371 7 2.01133563331691 3.48027444253859 0.0036943871506212 0.0663913887902374 240;1508;2171;3320;5328;5594;23385 FABP5;ALOX5;NCSTN;CTSB;MAPK1;HSP90AA1;PLAU geneontology_Biological_Process

GO:0016049 cell growth http://amigo.geneontology.org/amigo/term/GO:0016049 371 7 2.01133563331691 3.48027444253859 0.0036943871506212 0.0663913887902374 596;2932;3320;5465;5467;5468;6098 PPARG;PPARA;GSK3B;BCL2;HSP90AA1;PPARD;ROS1 geneontology_Biological_Process

GO:0050995 negative regulation of lipid catabolic process http://amigo.geneontology.org/amigo/term/GO:0050995 17 2 0.0921636274026614 21.7005347593583 0.00372528537450845 0.0663913887902374 150;1268 CNR1;ADRA2A geneontology_Biological_Process

GO:0048243 norepinephrine secretion http://amigo.geneontology.org/amigo/term/GO:0048243 17 2 0.0921636274026614 21.7005347593583 0.00372528537450845 0.0663913887902374 150;152 ADRA2A;ADRA2C geneontology_Biological_Process

GO:0002544 chronic inflammatory response http://amigo.geneontology.org/amigo/term/GO:0002544 17 2 0.0921636274026614 21.7005347593583 0.00372528537450845 0.0663913887902374 136;9536 ADORA2B;PTGES geneontology_Biological_Process

GO:0042359 vitamin D metabolic process http://amigo.geneontology.org/amigo/term/GO:0042359 17 2 0.0921636274026614 21.7005347593583 0.00372528537450845 0.0663913887902374 2260;5641 FGFR1;LGMN geneontology_Biological_Process

GO:0002283 neutrophil activation involved in immune response http://amigo.geneontology.org/amigo/term/GO:0002283 372 7 2.01675702316412 3.47091886608016 0.00374959363776028 0.066676107309684 240;1508;2171;3320;5328;5594;23385 FABP5;ALOX5;NCSTN;CTSB;MAPK1;HSP90AA1;PLAU geneontology_Biological_Process

GO:0010608 posttranscriptional regulation of gene expression http://amigo.geneontology.org/amigo/term/GO:0010608 373 7 2.02217841301134 3.46161345357056 0.00380542955810381 0.0675189519377976 1636;5468;5594;5914;6256;6774;9261 ACE;PPARG;MAPKAPK2;MAPK1;STAT3;RXRA;RARA geneontology_Biological_Process

GO:0042542 response to hydrogen peroxide http://amigo.geneontology.org/amigo/term/GO:0042542 119 4 0.64514539181863 6.20015278838808 0.00387154830332181 0.0683888068944396 596;836;3939;4193 CASP3;BCL2;MDM2;LDHA geneontology_Biological_Process

GO:0009408 response to heat http://amigo.geneontology.org/amigo/term/GO:0009408 119 4 0.64514539181863 6.20015278838808 0.00387154830332181 0.0683888068944396 2932;3320;5594;9261 GSK3B;MAPKAPK2;MAPK1;HSP90AA1 geneontology_Biological_Process

GO:0034754 cellular hormone metabolic process http://amigo.geneontology.org/amigo/term/GO:0034754 59 3 0.319862000985707 9.37904468412943 0.00395531897943457 0.06971467505162 231;5950;8644 AKR1B1;AKR1C3;RBP4 geneontology_Biological_Process

GO:0032147 activation of protein kinase activity http://amigo.geneontology.org/amigo/term/GO:0032147 282 6 1.52883193691474 3.92456479690522 0.00404302922100785 0.071103999618692 136;150;152;5594;9020;9261 ADORA2B;MAP3K14;ADRA2A;ADRA2C;MAPKAPK2;MAPK1 geneontology_Biological_Process

GO:1903362 regulation of cellular protein catabolic process http://amigo.geneontology.org/amigo/term/GO:1903362 196 5 1.06259241005421 4.70547309833024 0.00406786324785346 0.0713838633976391 2932;3320;4193;5747;9817 GSK3B;KEAP1;PTK2;HSP90AA1;MDM2 geneontology_Biological_Process

GO:0046824 positive regulation of nucleocytoplasmic transport http://amigo.geneontology.org/amigo/term/GO:0046824 60 3 0.325283390832923 9.22272727272727 0.00414765118070515 0.0724940758647575 2932;4193;5594 GSK3B;MAPK1;MDM2 geneontology_Biological_Process

GO:0042119 neutrophil activation http://amigo.geneontology.org/amigo/term/GO:0042119 379 7 2.05470675209463 3.4068121851763 0.0041539251540158 0.0724940758647575 240;1508;2171;3320;5328;5594;23385 FABP5;ALOX5;NCSTN;CTSB;MAPK1;HSP90AA1;PLAU geneontology_Biological_Process

GO:0071280 cellular response to copper ion http://amigo.geneontology.org/amigo/term/GO:0071280 18 2 0.0975850172498768 20.4949494949495 0.00417642701495291 0.0724940758647575 1544;23621 BACE1;CYP1A2 geneontology_Biological_Process

GO:0002363 alpha-beta T cell lineage commitment http://amigo.geneontology.org/amigo/term/GO:0002363 18 2 0.0975850172498768 20.4949494949495 0.00417642701495291 0.0724940758647575 596;6774 BCL2;STAT3 geneontology_Biological_Process

GO:0046827 positive regulation of protein export from nucleus http://amigo.geneontology.org/amigo/term/GO:0046827 18 2 0.0975850172498768 20.4949494949495 0.00417642701495291 0.0724940758647575 2932;4193 GSK3B;MDM2 geneontology_Biological_Process

GO:0051588 regulation of neurotransmitter transport http://amigo.geneontology.org/amigo/term/GO:0051588 122 4 0.661409561360276 6.04769001490313 0.00423148567458909 0.0727740987199211 136;1268;2932;23621 BACE1;GSK3B;CNR1;ADORA2B geneontology_Biological_Process

GO:0035051 cardiocyte differentiation http://amigo.geneontology.org/amigo/term/GO:0035051 122 4 0.661409561360276 6.04769001490313 0.00423148567458909 0.0727740987199211 5465;5594;5914;6256 PPARA;MAPK1;RXRA;RARA geneontology_Biological_Process

GO:0001935 endothelial cell proliferation http://amigo.geneontology.org/amigo/term/GO:0001935 122 4 0.661409561360276 6.04769001490313 0.00423148567458909 0.0727740987199211 2260;3357;5468;6774 PPARG;STAT3;FGFR1;HTR2B geneontology_Biological_Process

GO:0050714 positive regulation of protein secretion http://amigo.geneontology.org/amigo/term/GO:0050714 198 5 1.07343518974864 4.65794306703398 0.00424712623121759 0.0727740987199211 43;834;3357;5467;5950 ACHE;CASP1;HTR2B;PPARD;RBP4 geneontology_Biological_Process

GO:0090257 regulation of muscle system process http://amigo.geneontology.org/amigo/term/GO:0090257 198 5 1.07343518974864 4.65794306703398 0.00424712623121759 0.0727740987199211 136;150;152;5465;5747 PPARA;ADORA2B;ADRA2A;ADRA2C;PTK2 geneontology_Biological_Process

GO:0071356 cellular response to tumor necrosis factor http://amigo.geneontology.org/amigo/term/GO:0071356 198 5 1.07343518974864 4.65794306703398 0.00424712623121759 0.0727740987199211 834;841;2167;5594;9020 MAP3K14;MAPK1;CASP8;CASP1;FABP4 geneontology_Biological_Process

GO:0010507 negative regulation of autophagy http://amigo.geneontology.org/amigo/term/GO:0010507 61 3 0.330704780680138 9.07153502235469 0.00434558963235032 0.0743021543548446 596;3357;5747 PTK2;BCL2;HTR2B geneontology_Biological_Process

GO:0002262 myeloid cell homeostasis http://amigo.geneontology.org/amigo/term/GO:0002262 123 4 0.666830951207491 5.99852180339985 0.00435632845065526 0.0743269515184293 836;6774;7067;23385 CASP3;NCSTN;STAT3;THRA geneontology_Biological_Process

GO:0030072 peptide hormone secretion http://amigo.geneontology.org/amigo/term/GO:0030072 200 5 1.08427796944308 4.61136363636364 0.00443198511512888 0.0754569040239602 150;152;1268;5467;5950 CNR1;ADRA2A;ADRA2C;PPARD;RBP4 geneontology_Biological_Process

GO:0035264 multicellular organism growth http://amigo.geneontology.org/amigo/term/GO:0035264 124 4 0.672252341054707 5.95014662756598 0.00448364111311372 0.076174301883516 596;5641;5914;6774 BCL2;STAT3;LGMN;RARA geneontology_Biological_Process

GO:0036230 granulocyte activation http://amigo.geneontology.org/amigo/term/GO:0036230 385 7 2.08723509117792 3.35371900826446 0.00452626432394287 0.0764164485015057 240;1508;2171;3320;5328;5594;23385 FABP5;ALOX5;NCSTN;CTSB;MAPK1;HSP90AA1;PLAU geneontology_Biological_Process

GO:0007611 learning or memory http://amigo.geneontology.org/amigo/term/GO:0007611 201 5 1.08969935929029 4.58842152872004 0.00452654293797972 0.0764164485015057 836;1268;5594;5641;7067 CASP3;CNR1;MAPK1;LGMN;THRA geneontology_Biological_Process

GO:0097191 extrinsic apoptotic signaling pathway http://amigo.geneontology.org/amigo/term/GO:0097191 201 5 1.08969935929029 4.58842152872004 0.00452654293797972 0.0764164485015057 596;836;841;2260;2932 CASP3;GSK3B;BCL2;CASP8;FGFR1 geneontology_Biological_Process

GO:0040014 regulation of multicellular organism growth http://amigo.geneontology.org/amigo/term/GO:0040014 62 3 0.336126170527353 8.92521994134897 0.00454918255579528 0.0766369659188923 596;5641;6774 BCL2;STAT3;LGMN geneontology_Biological_Process

GO:2000058 regulation of ubiquitin-dependent protein catabolic process http://amigo.geneontology.org/amigo/term/GO:2000058 125 4 0.677673730901922 5.90254545454545 0.00461344628947002 0.0769052784993682 2932;4193;5747;9817 GSK3B;KEAP1;PTK2;MDM2 geneontology_Biological_Process

GO:0048872 homeostasis of number of cells http://amigo.geneontology.org/amigo/term/GO:0048872 202 5 1.09512074913751 4.56570657065707 0.00462253561114045 0.0769052784993682 596;836;6774;7067;23385 CASP3;NCSTN;BCL2;STAT3;THRA geneontology_Biological_Process

GO:0045932 negative regulation of muscle contraction http://amigo.geneontology.org/amigo/term/GO:0045932 19 2 0.103006407097092 19.4162679425837 0.00465160644759988 0.0769052784993682 136;152 ADORA2B;ADRA2C geneontology_Biological_Process

GO:0051452 intracellular pH reduction http://amigo.geneontology.org/amigo/term/GO:0051452 19 2 0.103006407097092 19.4162679425837 0.00465160644759988 0.0769052784993682 596;760 BCL2;CA2 geneontology_Biological_Process

GO:0007413 axonal fasciculation http://amigo.geneontology.org/amigo/term/GO:0007413 19 2 0.103006407097092 19.4162679425837 0.00465160644759988 0.0769052784993682 836;1268 CASP3;CNR1 geneontology_Biological_Process

GO:0106030 neuron projection fasciculation http://amigo.geneontology.org/amigo/term/GO:0106030 19 2 0.103006407097092 19.4162679425837 0.00465160644759988 0.0769052784993682 836;1268 CASP3;CNR1 geneontology_Biological_Process

GO:0055022 negative regulation of cardiac muscle tissue growth http://amigo.geneontology.org/amigo/term/GO:0055022 19 2 0.103006407097092 19.4162679425837 0.00465160644759988 0.0769052784993682 5465;5950 PPARA;RBP4 geneontology_Biological_Process

GO:0061117 negative regulation of heart growth http://amigo.geneontology.org/amigo/term/GO:0061117 19 2 0.103006407097092 19.4162679425837 0.00465160644759988 0.0769052784993682 5465;5950 PPARA;RBP4 geneontology_Biological_Process

GO:0010869 regulation of receptor biosynthetic process http://amigo.geneontology.org/amigo/term/GO:0010869 19 2 0.103006407097092 19.4162679425837 0.00465160644759988 0.0769052784993682 5465;5468 PPARG;PPARA geneontology_Biological_Process

GO:0046777 protein autophosphorylation http://amigo.geneontology.org/amigo/term/GO:0046777 203 5 1.10054213898472 4.54321540528437 0.00471997506356825 0.0778747225952023 1636;2260;2932;5747;6098 ACE;GSK3B;PTK2;FGFR1;ROS1 geneontology_Biological_Process

GO:0006096 glycolytic process http://amigo.geneontology.org/amigo/term/GO:0006096 63 3 0.341547560374569 8.78354978354978 0.00475847694260945 0.0780273206859852 3939;5465;6774 PPARA;STAT3;LDHA geneontology_Biological_Process

GO:0006757 ATP generation from ADP http://amigo.geneontology.org/amigo/term/GO:0006757 63 3 0.341547560374569 8.78354978354978 0.00475847694260945 0.0780273206859852 3939;5465;6774 PPARA;STAT3;LDHA geneontology_Biological_Process

GO:0019319 hexose biosynthetic process http://amigo.geneontology.org/amigo/term/GO:0019319 63 3 0.341547560374569 8.78354978354978 0.00475847694260945 0.0780273206859852 231;5465;5950 AKR1B1;PPARA;RBP4 geneontology_Biological_Process

GO:0006909 phagocytosis http://amigo.geneontology.org/amigo/term/GO:0006909 204 5 1.10596352883194 4.52094474153298 0.00481887320842322 0.078695149824087 3320;5468;5594;5747;5914 PPARG;PTK2;MAPK1;HSP90AA1;RARA geneontology_Biological_Process

GO:2000146 negative regulation of cell motility http://amigo.geneontology.org/amigo/term/GO:2000146 204 5 1.10596352883194 4.52094474153298 0.00481887320842322 0.078695149824087 140;596;5467;5468;6774 PPARG;ADORA3;BCL2;STAT3;PPARD geneontology_Biological_Process

GO:0046486 glycerolipid metabolic process http://amigo.geneontology.org/amigo/term/GO:0046486 294 6 1.59388861508132 3.76437847866419 0.0049511841504537 0.0806911926108565 43;2167;2171;2260;3357;11343 ACHE;MGLL;FABP5;FGFR1;HTR2B;FABP4 geneontology_Biological_Process

GO:0042866 pyruvate biosynthetic process http://amigo.geneontology.org/amigo/term/GO:0042866 64 3 0.346968950221784 8.64630681818182 0.00497351856862682 0.0808904381832354 3939;5465;6774 PPARA;STAT3;LDHA geneontology_Biological_Process

GO:0001503 ossification http://amigo.geneontology.org/amigo/term/GO:0001503 295 6 1.59931000492854 3.75161787365177 0.00503313337439182 0.0816939822756254 43;249;596;5594;5747;7067 ACHE;PTK2;ALPL;BCL2;MAPK1;THRA geneontology_Biological_Process

GO:0014706 striated muscle tissue development http://amigo.geneontology.org/amigo/term/GO:0014706 296 6 1.60473139477575 3.73894348894349 0.00511608082106663 0.0822203851968829 596;2260;5465;5914;5950;6256 PPARA;BCL2;RXRA;FGFR1;RBP4;RARA geneontology_Biological_Process

GO:0071379 cellular response to prostaglandin stimulus http://amigo.geneontology.org/amigo/term/GO:0071379 20 2 0.108427796944308 18.4454545454545 0.00515055945128684 0.0822203851968829 5468;8644 PPARG;AKR1C3 geneontology_Biological_Process

GO:0035588 G protein-coupled purinergic receptor signaling pathway http://amigo.geneontology.org/amigo/term/GO:0035588 20 2 0.108427796944308 18.4454545454545 0.00515055945128684 0.0822203851968829 136;140 ADORA2B;ADORA3 geneontology_Biological_Process

GO:1903579 negative regulation of ATP metabolic process http://amigo.geneontology.org/amigo/term/GO:1903579 20 2 0.108427796944308 18.4454545454545 0.00515055945128684 0.0822203851968829 5465;6774 PPARA;STAT3 geneontology_Biological_Process

GO:2000209 regulation of anoikis http://amigo.geneontology.org/amigo/term/GO:2000209 20 2 0.108427796944308 18.4454545454545 0.00515055945128684 0.0822203851968829 596;5747 PTK2;BCL2 geneontology_Biological_Process

GO:0043369 CD4-positive or CD8-positive, alpha-beta T cell lineage commitment http://amigo.geneontology.org/amigo/term/GO:0043369 20 2 0.108427796944308 18.4454545454545 0.00515055945128684 0.0822203851968829 596;6774 BCL2;STAT3 geneontology_Biological_Process

GO:2001026 regulation of endothelial cell chemotaxis http://amigo.geneontology.org/amigo/term/GO:2001026 20 2 0.108427796944308 18.4454545454545 0.00515055945128684 0.0822203851968829 2260;5641 FGFR1;LGMN geneontology_Biological_Process

GO:1903034 regulation of response to wounding http://amigo.geneontology.org/amigo/term/GO:1903034 129 4 0.699359290290784 5.71952078928823 0.00515803966118911 0.0822203851968829 150;239;5328;5467 ALOX12;ADRA2A;PLAU;PPARD geneontology_Biological_Process

GO:0046661 male sex differentiation http://amigo.geneontology.org/amigo/term/GO:0046661 129 4 0.699359290290784 5.71952078928823 0.00515803966118911 0.0822203851968829 596;5914;5972;8644 REN;AKR1C3;BCL2;RARA geneontology_Biological_Process

GO:0010594 regulation of endothelial cell migration http://amigo.geneontology.org/amigo/term/GO:0010594 131 4 0.710202069985214 5.63219986120749 0.00544586909158928 0.0861222222745009 2260;5468;5641;5747 PPARG;PTK2;FGFR1;LGMN geneontology_Biological_Process

GO:0046890 regulation of lipid biosynthetic process http://amigo.geneontology.org/amigo/term/GO:0046890 131 4 0.710202069985214 5.63219986120749 0.00544586909158928 0.0861222222745009 2171;3357;6319;8644 FABP5;AKR1C3;HTR2B;SCD geneontology_Biological_Process

GO:0048489 synaptic vesicle transport http://amigo.geneontology.org/amigo/term/GO:0048489 131 4 0.710202069985214 5.63219986120749 0.00544586909158928 0.0861222222745009 136;1268;2932;23621 BACE1;GSK3B;CNR1;ADORA2B geneontology_Biological_Process

GO:0097480 establishment of synaptic vesicle localization http://amigo.geneontology.org/amigo/term/GO:0097480 131 4 0.710202069985214 5.63219986120749 0.00544586909158928 0.0861222222745009 136;1268;2932;23621 BACE1;GSK3B;CNR1;ADORA2B geneontology_Biological_Process

GO:0090066 regulation of anatomical structure size http://amigo.geneontology.org/amigo/term/GO:0090066 399 7 2.16313454903894 3.23604465709729 0.00549356479799568 0.0867051390800028 136;150;152;1636;2932;3357;5467 ACE;GSK3B;ADORA2B;ADRA2A;ADRA2C;HTR2B;PPARD geneontology_Biological_Process

GO:0022408 negative regulation of cell-cell adhesion http://amigo.geneontology.org/amigo/term/GO:0022408 132 4 0.71562345983243 5.58953168044077 0.0055937432225508 0.0879393580881168 239;836;5465;5747 CASP3;PPARA;ALOX12;PTK2 geneontology_Biological_Process

GO:0003205 cardiac chamber development http://amigo.geneontology.org/amigo/term/GO:0003205 132 4 0.71562345983243 5.58953168044077 0.0055937432225508 0.0879393580881168 4193;5914;5950;6256 RXRA;MDM2;RBP4;RARA geneontology_Biological_Process

GO:0006639 acylglycerol metabolic process http://amigo.geneontology.org/amigo/term/GO:0006639 67 3 0.36323311976343 8.25915875169606 0.00565356669730221 0.0879758469555563 2167;2171;11343 MGLL;FABP5;FABP4 geneontology_Biological_Process

GO:0006836 neurotransmitter transport http://amigo.geneontology.org/amigo/term/GO:0006836 212 5 1.14933464760966 4.35034305317324 0.00566399492303438 0.0879758469555563 136;1268;2932;6580;23621 SLC22A1;BACE1;GSK3B;CNR1;ADORA2B geneontology_Biological_Process

GO:0060259 regulation of feeding behavior http://amigo.geneontology.org/amigo/term/GO:0060259 21 2 0.113849186791523 17.5670995670996 0.00567302387266522 0.0879758469555563 1268;6774 CNR1;STAT3 geneontology_Biological_Process

GO:0003081 regulation of systemic arterial blood pressure by renin-angiotensin http://amigo.geneontology.org/amigo/term/GO:0003081 21 2 0.113849186791523 17.5670995670996 0.00567302387266522 0.0879758469555563 1636;5972 ACE;REN geneontology_Biological_Process

GO:0045851 pH reduction http://amigo.geneontology.org/amigo/term/GO:0045851 21 2 0.113849186791523 17.5670995670996 0.00567302387266522 0.0879758469555563 596;760 BCL2;CA2 geneontology_Biological_Process

GO:0002068 glandular epithelial cell development http://amigo.geneontology.org/amigo/term/GO:0002068 21 2 0.113849186791523 17.5670995670996 0.00567302387266522 0.0879758469555563 2932;5914 GSK3B;RARA geneontology_Biological_Process

GO:1902254 negative regulation of intrinsic apoptotic signaling pathway by p53 class mediator http://amigo.geneontology.org/amigo/term/GO:1902254 21 2 0.113849186791523 17.5670995670996 0.00567302387266522 0.0879758469555563 596;4193 BCL2;MDM2 geneontology_Biological_Process

GO:0034404 nucleobase-containing small molecule biosynthetic process http://amigo.geneontology.org/amigo/term/GO:0034404 133 4 0.721044849679645 5.54750512645249 0.00574428559206597 0.0887370141075519 3939;5141;5465;6774 PPARA;PDE4A;STAT3;LDHA geneontology_Biological_Process

GO:0048469 cell maturation http://amigo.geneontology.org/amigo/term/GO:0048469 133 4 0.721044849679645 5.54750512645249 0.00574428559206597 0.0887370141075519 596;2260;5468;5972 REN;PPARG;BCL2;FGFR1 geneontology_Biological_Process

GO:0002793 positive regulation of peptide secretion http://amigo.geneontology.org/amigo/term/GO:0002793 214 5 1.16017742730409 4.30968564146134 0.00589069047994939 0.0903219037381223 43;834;3357;5467;5950 ACHE;CASP1;HTR2B;PPARD;RBP4 geneontology_Biological_Process

GO:0006638 neutral lipid metabolic process http://amigo.geneontology.org/amigo/term/GO:0006638 68 3 0.368654509610646 8.13770053475936 0.00589203121110971 0.0903219037381223 2167;2171;11343 MGLL;FABP5;FABP4 geneontology_Biological_Process

GO:1900371 regulation of purine nucleotide biosynthetic process http://amigo.geneontology.org/amigo/term/GO:1900371 68 3 0.368654509610646 8.13770053475936 0.00589203121110971 0.0903219037381223 136;5465;6774 PPARA;ADORA2B;STAT3 geneontology_Biological_Process

GO:0021766 hippocampus development http://amigo.geneontology.org/amigo/term/GO:0021766 68 3 0.368654509610646 8.13770053475936 0.00589203121110971 0.0903219037381223 836;2932;5914 CASP3;GSK3B;RARA geneontology_Biological_Process

GO:0030808 regulation of nucleotide biosynthetic process http://amigo.geneontology.org/amigo/term/GO:0030808 69 3 0.374075899457861 8.0197628458498 0.00613645409289343 0.0931762915585071 136;5465;6774 PPARA;ADORA2B;STAT3 geneontology_Biological_Process

GO:0014031 mesenchymal cell development http://amigo.geneontology.org/amigo/term/GO:0014031 69 3 0.374075899457861 8.0197628458498 0.00613645409289343 0.0931762915585071 596;3357;5594 BCL2;MAPK1;HTR2B geneontology_Biological_Process

GO:0071260 cellular response to mechanical stimulus http://amigo.geneontology.org/amigo/term/GO:0071260 69 3 0.374075899457861 8.0197628458498 0.00613645409289343 0.0931762915585071 834;841;9020 MAP3K14;CASP8;CASP1 geneontology_Biological_Process

GO:0032436 positive regulation of proteasomal ubiquitin-dependent protein catabolic process http://amigo.geneontology.org/amigo/term/GO:0032436 69 3 0.374075899457861 8.0197628458498 0.00613645409289343 0.0931762915585071 2932;4193;9817 GSK3B;KEAP1;MDM2 geneontology_Biological_Process

GO:0046364 monosaccharide biosynthetic process http://amigo.geneontology.org/amigo/term/GO:0046364 69 3 0.374075899457861 8.0197628458498 0.00613645409289343 0.0931762915585071 231;5465;5950 AKR1B1;PPARA;RBP4 geneontology_Biological_Process

GO:0060537 muscle tissue development http://amigo.geneontology.org/amigo/term/GO:0060537 308 6 1.66978807294234 3.59327036599764 0.00619194362369679 0.0937144150223248 596;2260;5465;5914;5950;6256 PPARA;BCL2;RXRA;FGFR1;RBP4;RARA geneontology_Biological_Process

GO:0030308 negative regulation of cell growth http://amigo.geneontology.org/amigo/term/GO:0030308 136 4 0.737309019221291 5.42513368983957 0.00621213406879573 0.0937144150223248 596;5465;5467;5468 PPARG;PPARA;BCL2;PPARD geneontology_Biological_Process

GO:1900543 negative regulation of purine nucleotide metabolic process http://amigo.geneontology.org/amigo/term/GO:1900543 22 2 0.119270576638738 16.7685950413223 0.00621873961220376 0.0937144150223248 5465;6774 PPARA;STAT3 geneontology_Biological_Process

GO:0060396 growth hormone receptor signaling pathway http://amigo.geneontology.org/amigo/term/GO:0060396 22 2 0.119270576638738 16.7685950413223 0.00621873961220376 0.0937144150223248 5747;6774 PTK2;STAT3 geneontology_Biological_Process

GO:0061564 axon development http://amigo.geneontology.org/amigo/term/GO:0061564 410 7 2.2227698373583 3.14922394678492 0.00635720196926903 0.0948196041897248 596;836;1268;2932;3320;5594;5747 CASP3;GSK3B;CNR1;PTK2;BCL2;MAPK1;HSP90AA1 geneontology_Biological_Process

GO:0002526 acute inflammatory response http://amigo.geneontology.org/amigo/term/GO:0002526 137 4 0.742730409068507 5.38553417385534 0.00637356052296933 0.0948196041897248 1268;5468;6774;9536 PPARG;CNR1;PTGES;STAT3 geneontology_Biological_Process

GO:0009108 coenzyme biosynthetic process http://amigo.geneontology.org/amigo/term/GO:0009108 137 4 0.742730409068507 5.38553417385534 0.00637356052296933 0.0948196041897248 3939;5465;6319;6774 PPARA;STAT3;LDHA;SCD geneontology_Biological_Process

GO:0006644 phospholipid metabolic process http://amigo.geneontology.org/amigo/term/GO:0006644 310 6 1.68063085263677 3.57008797653959 0.00638620795833234 0.0948196041897248 43;2171;2260;3357;5467;5747 ACHE;FABP5;PTK2;FGFR1;HTR2B;PPARD geneontology_Biological_Process

GO:0007517 muscle organ development http://amigo.geneontology.org/amigo/term/GO:0007517 310 6 1.68063085263677 3.57008797653959 0.00638620795833234 0.0948196041897248 43;596;2260;5465;5950;6256 PPARA;ACHE;BCL2;RXRA;FGFR1;RBP4 geneontology_Biological_Process

GO:0046031 ADP metabolic process http://amigo.geneontology.org/amigo/term/GO:0046031 70 3 0.379497289305076 7.9051948051948 0.00638687411375427 0.0948196041897248 3939;5465;6774 PPARA;STAT3;LDHA geneontology_Biological_Process

GO:0050886 endocrine process http://amigo.geneontology.org/amigo/term/GO:0050886 70 3 0.379497289305076 7.9051948051948 0.00638687411375427 0.0948196041897248 1636;2260;5972 ACE;REN;FGFR1 geneontology_Biological_Process

GO:0055024 regulation of cardiac muscle tissue development http://amigo.geneontology.org/amigo/term/GO:0055024 70 3 0.379497289305076 7.9051948051948 0.00638687411375427 0.0948196041897248 2260;5465;5950 PPARA;FGFR1;RBP4 geneontology_Biological_Process

GO:0051146 striated muscle cell differentiation http://amigo.geneontology.org/amigo/term/GO:0051146 219 5 1.18728437654017 4.21129099211291 0.00648543033461624 0.0961044695140725 596;836;5465;5914;6256 CASP3;PPARA;BCL2;RXRA;RARA geneontology_Biological_Process

GO:0050806 positive regulation of synaptic transmission http://amigo.geneontology.org/amigo/term/GO:0050806 138 4 0.748151798915722 5.34650856389987 0.00653775996861916 0.0967008415321452 760;1268;5594;5641 CNR1;MAPK1;CA2;LGMN geneontology_Biological_Process

GO:0002433 immune response-regulating cell surface receptor signaling pathway involved in phagocytosis http://amigo.geneontology.org/amigo/term/GO:0002433 71 3 0.384918679152292 7.79385403329065 0.00664332892449182 0.0979004015723454 3320;5594;5747 PTK2;MAPK1;HSP90AA1 geneontology_Biological_Process

GO:0038096 Fc-gamma receptor signaling pathway involved in phagocytosis http://amigo.geneontology.org/amigo/term/GO:0038096 71 3 0.384918679152292 7.79385403329065 0.00664332892449182 0.0979004015723454 3320;5594;5747 PTK2;MAPK1;HSP90AA1 geneontology_Biological_Process

GO:0042176 regulation of protein catabolic process http://amigo.geneontology.org/amigo/term/GO:0042176 313 6 1.69689502217841 3.5358698809178 0.00668592270363488 0.0983469732986881 150;2932;3320;4193;5747;9817 GSK3B;ADRA2A;KEAP1;PTK2;HSP90AA1;MDM2 geneontology_Biological_Process

GO:0001782 B cell homeostasis http://amigo.geneontology.org/amigo/term/GO:0001782 23 2 0.124691966485954 16.0395256916996 0.0067874486102768 0.0985719850806442 596;836 CASP3;BCL2 geneontology_Biological_Process

GO:0045980 negative regulation of nucleotide metabolic process http://amigo.geneontology.org/amigo/term/GO:0045980 23 2 0.124691966485954 16.0395256916996 0.0067874486102768 0.0985719850806442 5465;6774 PPARA;STAT3 geneontology_Biological_Process

GO:0097066 response to thyroid hormone http://amigo.geneontology.org/amigo/term/GO:0097066 23 2 0.124691966485954 16.0395256916996 0.0067874486102768 0.0985719850806442 231;1508 AKR1B1;CTSB geneontology_Biological_Process

GO:0009299 mRNA transcription http://amigo.geneontology.org/amigo/term/GO:0009299 23 2 0.124691966485954 16.0395256916996 0.0067874486102768 0.0985719850806442 5467;6774 STAT3;PPARD geneontology_Biological_Process

GO:0071295 cellular response to vitamin http://amigo.geneontology.org/amigo/term/GO:0071295 23 2 0.124691966485954 16.0395256916996 0.0067874486102768 0.0985719850806442 4193;5468 PPARG;MDM2 geneontology_Biological_Process

GO:0030878 thyroid gland development http://amigo.geneontology.org/amigo/term/GO:0030878 23 2 0.124691966485954 16.0395256916996 0.0067874486102768 0.0985719850806442 5594;7067 MAPK1;THRA geneontology_Biological_Process

GO:0071378 cellular response to growth hormone stimulus http://amigo.geneontology.org/amigo/term/GO:0071378 23 2 0.124691966485954 16.0395256916996 0.0067874486102768 0.0985719850806442 5747;6774 PTK2;STAT3 geneontology_Biological_Process

GO:0009117 nucleotide metabolic process http://amigo.geneontology.org/amigo/term/GO:0009117 416 7 2.2552981764416 3.10380244755245 0.00686928176418822 0.0991175226800847 136;3939;5141;5465;5594;6319;6774 PPARA;ADORA2B;PDE4A;MAPK1;STAT3;LDHA;SCD geneontology_Biological_Process

GO:0007269 neurotransmitter secretion http://amigo.geneontology.org/amigo/term/GO:0007269 140 4 0.758994578610153 5.27012987012987 0.00687455949605686 0.0991175226800847 136;1268;2932;23621 BACE1;GSK3B;CNR1;ADORA2B geneontology_Biological_Process

GO:0099643 signal release from synapse http://amigo.geneontology.org/amigo/term/GO:0099643 140 4 0.758994578610153 5.27012987012987 0.00687455949605686 0.0991175226800847 136;1268;2932;23621 BACE1;GSK3B;CNR1;ADORA2B geneontology_Biological_Process

GO:0051897 positive regulation of protein kinase B signaling http://amigo.geneontology.org/amigo/term/GO:0051897 140 4 0.758994578610153 5.27012987012987 0.00687455949605686 0.0991175226800847 2260;3320;5747;8644 AKR1C3;PTK2;HSP90AA1;FGFR1 geneontology_Biological_Process

GO:0038094 Fc-gamma receptor signaling pathway http://amigo.geneontology.org/amigo/term/GO:0038094 72 3 0.390340068999507 7.68560606060606 0.0069058550687604 0.0993896623385265 3320;5594;5747 PTK2;MAPK1;HSP90AA1 geneontology_Biological_Process

GO:0097479 synaptic vesicle localization http://amigo.geneontology.org/amigo/term/GO:0097479 141 4 0.764415968457368 5.2327530625403 0.00704719998697279 0.101241820997767 136;1268;2932;23621 BACE1;GSK3B;CNR1;ADORA2B geneontology_Biological_Process

GO:0070542 response to fatty acid http://amigo.geneontology.org/amigo/term/GO:0070542 73 3 0.395761458846723 7.58032378580324 0.00717448799610998 0.102885757965721 5468;6319;8644 PPARG;AKR1C3;SCD geneontology_Biological_Process

GO:2000377 regulation of reactive oxygen species metabolic process http://amigo.geneontology.org/amigo/term/GO:2000377 142 4 0.769837358304584 5.19590268886044 0.00722269428465827 0.103090822896929 596;3320;6774;8644 AKR1C3;BCL2;STAT3;HSP90AA1 geneontology_Biological_Process

GO:0016051 carbohydrate biosynthetic process http://amigo.geneontology.org/amigo/term/GO:0016051 142 4 0.769837358304584 5.19590268886044 0.00722269428465827 0.103090822896929 231;2932;5465;5950 AKR1B1;PPARA;GSK3B;RBP4 geneontology_Biological_Process

GO:0006753 nucleoside phosphate metabolic process http://amigo.geneontology.org/amigo/term/GO:0006753 420 7 2.27698373583046 3.07424242424242 0.00722743709637308 0.103090822896929 136;3939;5141;5465;5594;6319;6774 PPARA;ADORA2B;PDE4A;MAPK1;STAT3;LDHA;SCD geneontology_Biological_Process

GO:0009268 response to pH http://amigo.geneontology.org/amigo/term/GO:0009268 24 2 0.130113356333169 15.3712121212121 0.00737889483334064 0.10371060421248 760;5641 CA2;LGMN geneontology_Biological_Process

GO:0034205 amyloid-beta formation http://amigo.geneontology.org/amigo/term/GO:0034205 24 2 0.130113356333169 15.3712121212121 0.00737889483334064 0.10371060421248 836;23385 CASP3;NCSTN geneontology_Biological_Process

GO:0071880 adenylate cyclase-activating adrenergic receptor signaling pathway http://amigo.geneontology.org/amigo/term/GO:0071880 24 2 0.130113356333169 15.3712121212121 0.00737889483334064 0.10371060421248 150;152 ADRA2A;ADRA2C geneontology_Biological_Process

GO:0045923 positive regulation of fatty acid metabolic process http://amigo.geneontology.org/amigo/term/GO:0045923 24 2 0.130113356333169 15.3712121212121 0.00737889483334064 0.10371060421248 5465;5468 PPARG;PPARA geneontology_Biological_Process

GO:0002360 T cell lineage commitment http://amigo.geneontology.org/amigo/term/GO:0002360 24 2 0.130113356333169 15.3712121212121 0.00737889483334064 0.10371060421248 596;6774 BCL2;STAT3 geneontology_Biological_Process

GO:1900739 regulation of protein insertion into mitochondrial membrane involved in apoptotic signaling pathway http://amigo.geneontology.org/amigo/term/GO:1900739 24 2 0.130113356333169 15.3712121212121 0.00737889483334064 0.10371060421248 596;841 BCL2;CASP8 geneontology_Biological_Process

GO:1900740 positive regulation of protein insertion into mitochondrial membrane involved in apoptotic signaling pathway http://amigo.geneontology.org/amigo/term/GO:1900740 24 2 0.130113356333169 15.3712121212121 0.00737889483334064 0.10371060421248 596;841 BCL2;CASP8 geneontology_Biological_Process

GO:0016486 peptide hormone processing http://amigo.geneontology.org/amigo/term/GO:0016486 24 2 0.130113356333169 15.3712121212121 0.00737889483334064 0.10371060421248 1636;5972 ACE;REN geneontology_Biological_Process

GO:0031647 regulation of protein stability http://amigo.geneontology.org/amigo/term/GO:0031647 226 5 1.22523410547068 4.08085277554304 0.00738753366672251 0.10371060421248 596;836;3320;4193;5594 CASP3;BCL2;MAPK1;HSP90AA1;MDM2 geneontology_Biological_Process

GO:0042594 response to starvation http://amigo.geneontology.org/amigo/term/GO:0042594 143 4 0.775258748151799 5.15956770502225 0.00740106216535907 0.103718562954822 596;5468;5594;8644 PPARG;AKR1C3;BCL2;MAPK1 geneontology_Biological_Process

GO:0006165 nucleoside diphosphate phosphorylation http://amigo.geneontology.org/amigo/term/GO:0006165 74 3 0.401182848693938 7.47788697788698 0.00744926207491048 0.103848423559989 3939;5465;6774 PPARA;STAT3;LDHA geneontology_Biological_Process

GO:0019359 nicotinamide nucleotide biosynthetic process http://amigo.geneontology.org/amigo/term/GO:0019359 74 3 0.401182848693938 7.47788697788698 0.00744926207491048 0.103848423559989 3939;5465;6774 PPARA;STAT3;LDHA geneontology_Biological_Process

GO:0019363 pyridine nucleotide biosynthetic process http://amigo.geneontology.org/amigo/term/GO:0019363 74 3 0.401182848693938 7.47788697788698 0.00744926207491048 0.103848423559989 3939;5465;6774 PPARA;STAT3;LDHA geneontology_Biological_Process

GO:0050890 cognition http://amigo.geneontology.org/amigo/term/GO:0050890 227 5 1.23065549531789 4.06287545054065 0.00752321981390613 0.104683627489313 836;1268;5594;5641;7067 CASP3;CNR1;MAPK1;LGMN;THRA geneontology_Biological_Process

GO:0001558 regulation of cell growth http://amigo.geneontology.org/amigo/term/GO:0001558 321 6 1.74026614095614 3.44774851316907 0.00753533734489431 0.104683627489313 596;2932;5465;5467;5468;6098 PPARG;PPARA;GSK3B;BCL2;PPARD;ROS1 geneontology_Biological_Process

GO:0032963 collagen metabolic process http://amigo.geneontology.org/amigo/term/GO:0032963 75 3 0.406604238541153 7.37818181818182 0.00773021060516121 0.107204757820624 1508;5467;5468 PPARG;CTSB;PPARD geneontology_Biological_Process

GO:0031960 response to corticosteroid http://amigo.geneontology.org/amigo/term/GO:0031960 145 4 0.78610152784623 5.0884012539185 0.00776649691023001 0.10752164061533 249;596;836;8644 CASP3;AKR1C3;ALPL;BCL2 geneontology_Biological_Process

GO:0034504 protein localization to nucleus http://amigo.geneontology.org/amigo/term/GO:0034504 229 5 1.24149827501232 4.02739182215165 0.00779981590575063 0.107796419478094 2932;4193;5594;6774;8644 GSK3B;AKR1C3;MAPK1;STAT3;MDM2 geneontology_Biological_Process

GO:0030168 platelet activation http://amigo.geneontology.org/amigo/term/GO:0030168 146 4 0.791522917693445 5.05354919053549 0.00795360245237031 0.109107077178842 150;152;239;5594 ALOX12;ADRA2A;ADRA2C;MAPK1 geneontology_Biological_Process

GO:0044070 regulation of anion transport http://amigo.geneontology.org/amigo/term/GO:0044070 76 3 0.412025628388369 7.2811004784689 0.00801736583118706 0.109107077178842 760;2260;6098 CA2;FGFR1;ROS1 geneontology_Biological_Process

GO:0002431 Fc receptor mediated stimulatory signaling pathway http://amigo.geneontology.org/amigo/term/GO:0002431 76 3 0.412025628388369 7.2811004784689 0.00801736583118706 0.109107077178842 3320;5594;5747 PTK2;MAPK1;HSP90AA1 geneontology_Biological_Process

GO:0051341 regulation of oxidoreductase activity http://amigo.geneontology.org/amigo/term/GO:0051341 76 3 0.412025628388369 7.2811004784689 0.00801736583118706 0.109107077178842 1268;3320;3357 CNR1;HSP90AA1;HTR2B geneontology_Biological_Process

GO:0046939 nucleotide phosphorylation http://amigo.geneontology.org/amigo/term/GO:0046939 76 3 0.412025628388369 7.2811004784689 0.00801736583118706 0.109107077178842 3939;5465;6774 PPARA;STAT3;LDHA geneontology_Biological_Process

GO:0072525 pyridine-containing compound biosynthetic process http://amigo.geneontology.org/amigo/term/GO:0072525 76 3 0.412025628388369 7.2811004784689 0.00801736583118706 0.109107077178842 3939;5465;6774 PPARA;STAT3;LDHA geneontology_Biological_Process

GO:0045639 positive regulation of myeloid cell differentiation http://amigo.geneontology.org/amigo/term/GO:0045639 76 3 0.412025628388369 7.2811004784689 0.00801736583118706 0.109107077178842 760;841;6774 STAT3;CASP8;CA2 geneontology_Biological_Process

GO:0003300 cardiac muscle hypertrophy http://amigo.geneontology.org/amigo/term/GO:0003300 76 3 0.412025628388369 7.2811004784689 0.00801736583118706 0.109107077178842 3357;5465;5747 PPARA;PTK2;HTR2B geneontology_Biological_Process

GO:0014068 positive regulation of phosphatidylinositol 3-kinase signaling http://amigo.geneontology.org/amigo/term/GO:0014068 76 3 0.412025628388369 7.2811004784689 0.00801736583118706 0.109107077178842 2260;5467;5747 PTK2;FGFR1;PPARD geneontology_Biological_Process

GO:0051271 negative regulation of cellular component movement http://amigo.geneontology.org/amigo/term/GO:0051271 231 5 1.25234105470675 3.99252262888626 0.00808345173410863 0.109819661759486 140;596;5467;5468;6774 PPARG;ADORA3;BCL2;STAT3;PPARD geneontology_Biological_Process

GO:0001667 ameboidal-type cell migration http://amigo.geneontology.org/amigo/term/GO:0001667 326 6 1.76737309019221 3.39486893474624 0.00810452877407164 0.109919388559528 2260;3357;5467;5468;5641;5747 PPARG;PTK2;FGFR1;HTR2B;LGMN;PPARD geneontology_Biological_Process

GO:0016054 organic acid catabolic process http://amigo.geneontology.org/amigo/term/GO:0016054 147 4 0.79694430754066 5.01917130488559 0.0081436589309194 0.110076957373677 1268;2166;5465;5467 PPARA;FAAH;CNR1;PPARD geneontology_Biological_Process

GO:0046395 carboxylic acid catabolic process http://amigo.geneontology.org/amigo/term/GO:0046395 147 4 0.79694430754066 5.01917130488559 0.0081436589309194 0.110076957373677 1268;2166;5465;5467 PPARA;FAAH;CNR1;PPARD geneontology_Biological_Process

GO:0051098 regulation of binding http://amigo.geneontology.org/amigo/term/GO:0051098 329 6 1.78363725973386 3.36391268306162 0.00846064587005579 0.11416878288733 596;1636;2932;5465;5468;5914 ACE;PPARG;PPARA;GSK3B;BCL2;RARA geneontology_Biological_Process

GO:0043583 ear development http://amigo.geneontology.org/amigo/term/GO:0043583 149 4 0.807787087235091 4.95179987797437 0.00853270009346518 0.114509348120004 596;2260;5594;9261 MAPKAPK2;BCL2;MAPK1;FGFR1 geneontology_Biological_Process

GO:0046434 organophosphate catabolic process http://amigo.geneontology.org/amigo/term/GO:0046434 149 4 0.807787087235091 4.95179987797437 0.00853270009346518 0.114509348120004 3939;5141;5465;6774 PPARA;PDE4A;STAT3;LDHA geneontology_Biological_Process

GO:0071453 cellular response to oxygen levels http://amigo.geneontology.org/amigo/term/GO:0071453 149 4 0.807787087235091 4.95179987797437 0.00853270009346518 0.114509348120004 596;4193;5467;5468 PPARG;BCL2;MDM2;PPARD geneontology_Biological_Process

GO:0014897 striated muscle hypertrophy http://amigo.geneontology.org/amigo/term/GO:0014897 78 3 0.422868408082799 7.09440559440559 0.00861042014487723 0.114509348120004 3357;5465;5747 PPARA;PTK2;HTR2B geneontology_Biological_Process

GO:0010743 regulation of macrophage derived foam cell differentiation http://amigo.geneontology.org/amigo/term/GO:0010743 26 2 0.1409561360276 14.1888111888112 0.00862898486832819 0.114509348120004 5465;5468 PPARG;PPARA geneontology_Biological_Process

GO:0007190 activation of adenylate cyclase activity http://amigo.geneontology.org/amigo/term/GO:0007190 26 2 0.1409561360276 14.1888111888112 0.00862898486832819 0.114509348120004 136;140 ADORA2B;ADORA3 geneontology_Biological_Process

GO:0035587 purinergic receptor signaling pathway http://amigo.geneontology.org/amigo/term/GO:0035587 26 2 0.1409561360276 14.1888111888112 0.00862898486832819 0.114509348120004 136;140 ADORA2B;ADORA3 geneontology_Biological_Process

GO:0021955 central nervous system neuron axonogenesis http://amigo.geneontology.org/amigo/term/GO:0021955 26 2 0.1409561360276 14.1888111888112 0.00862898486832819 0.114509348120004 3320;5747 PTK2;HSP90AA1 geneontology_Biological_Process

GO:0071875 adrenergic receptor signaling pathway http://amigo.geneontology.org/amigo/term/GO:0071875 26 2 0.1409561360276 14.1888111888112 0.00862898486832819 0.114509348120004 150;152 ADRA2A;ADRA2C geneontology_Biological_Process

GO:0035767 endothelial cell chemotaxis http://amigo.geneontology.org/amigo/term/GO:0035767 26 2 0.1409561360276 14.1888111888112 0.00862898486832819 0.114509348120004 2260;5641 FGFR1;LGMN geneontology_Biological_Process

GO:0006754 ATP biosynthetic process http://amigo.geneontology.org/amigo/term/GO:0006754 79 3 0.428289797930015 7.00460299194476 0.00891637855550553 0.118127253644297 3939;5465;6774 PPARA;STAT3;LDHA geneontology_Biological_Process

GO:0043123 positive regulation of I-kappaB kinase/NF-kappaB signaling http://amigo.geneontology.org/amigo/term/GO:0043123 152 4 0.824051256776737 4.85406698564593 0.00913886033517364 0.12087464529266 834;841;3357;9020 MAP3K14;CASP8;CASP1;HTR2B geneontology_Biological_Process

GO:0009135 purine nucleoside diphosphate metabolic process http://amigo.geneontology.org/amigo/term/GO:0009135 80 3 0.43371118777723 6.91704545454545 0.00922866233244601 0.120877871234142 3939;5465;6774 PPARA;STAT3;LDHA geneontology_Biological_Process

GO:0009179 purine ribonucleoside diphosphate metabolic process http://amigo.geneontology.org/amigo/term/GO:0009179 80 3 0.43371118777723 6.91704545454545 0.00922866233244601 0.120877871234142 3939;5465;6774 PPARA;STAT3;LDHA geneontology_Biological_Process

GO:0014896 muscle hypertrophy http://amigo.geneontology.org/amigo/term/GO:0014896 80 3 0.43371118777723 6.91704545454545 0.00922866233244601 0.120877871234142 3357;5465;5747 PPARA;PTK2;HTR2B geneontology_Biological_Process

GO:0048010 vascular endothelial growth factor receptor signaling pathway http://amigo.geneontology.org/amigo/term/GO:0048010 80 3 0.43371118777723 6.91704545454545 0.00922866233244601 0.120877871234142 3320;5747;9261 PTK2;MAPKAPK2;HSP90AA1 geneontology_Biological_Process

GO:0060627 regulation of vesicle-mediated transport http://amigo.geneontology.org/amigo/term/GO:0060627 440 7 2.38541153277477 2.9345041322314 0.00923128847349652 0.120877871234142 136;150;1268;2932;5468;5594;23621 PPARG;BACE1;GSK3B;CNR1;ADORA2B;ADRA2A;MAPK1 geneontology_Biological_Process

GO:0008207 C21-steroid hormone metabolic process http://amigo.geneontology.org/amigo/term/GO:0008207 27 2 0.146377525874815 13.6632996632997 0.00928712662035225 0.120877871234142 231;8644 AKR1B1;AKR1C3 geneontology_Biological_Process

GO:0032965 regulation of collagen biosynthetic process http://amigo.geneontology.org/amigo/term/GO:0032965 27 2 0.146377525874815 13.6632996632997 0.00928712662035225 0.120877871234142 5467;5468 PPARG;PPARD geneontology_Biological_Process

GO:0033032 regulation of myeloid cell apoptotic process http://amigo.geneontology.org/amigo/term/GO:0033032 27 2 0.146377525874815 13.6632996632997 0.00928712662035225 0.120877871234142 596;7067 BCL2;THRA geneontology_Biological_Process

GO:1901797 negative regulation of signal transduction by p53 class mediator http://amigo.geneontology.org/amigo/term/GO:1901797 27 2 0.146377525874815 13.6632996632997 0.00928712662035225 0.120877871234142 596;4193 BCL2;MDM2 geneontology_Biological_Process

GO:0010631 epithelial cell migration http://amigo.geneontology.org/amigo/term/GO:0010631 239 5 1.29571217348448 3.85888170406999 0.00929016381017211 0.120877871234142 2260;5467;5468;5641;5747 PPARG;PTK2;FGFR1;LGMN;PPARD geneontology_Biological_Process

GO:1903708 positive regulation of hemopoiesis http://amigo.geneontology.org/amigo/term/GO:1903708 153 4 0.829472646623953 4.82234105763518 0.00934701286501638 0.121420124912112 760;841;5914;6774 STAT3;CASP8;CA2;RARA geneontology_Biological_Process

GO:0090132 epithelium migration http://amigo.geneontology.org/amigo/term/GO:0090132 240 5 1.30113356333169 3.84280303030303 0.00944930222869167 0.12254994559804 2260;5467;5468;5641;5747 PPARG;PTK2;FGFR1;LGMN;PPARD geneontology_Biological_Process

GO:1901655 cellular response to ketone http://amigo.geneontology.org/amigo/term/GO:1901655 81 3 0.439132577624446 6.83164983164983 0.00954729862816539 0.123023323063735 231;5468;8644 AKR1B1;PPARG;AKR1C3 geneontology_Biological_Process

GO:0009185 ribonucleoside diphosphate metabolic process http://amigo.geneontology.org/amigo/term/GO:0009185 81 3 0.439132577624446 6.83164983164983 0.00954729862816539 0.123023323063735 3939;5465;6774 PPARA;STAT3;LDHA geneontology_Biological_Process

GO:1900542 regulation of purine nucleotide metabolic process http://amigo.geneontology.org/amigo/term/GO:1900542 81 3 0.439132577624446 6.83164983164983 0.00954729862816539 0.123023323063735 136;5465;6774 PPARA;ADORA2B;STAT3 geneontology_Biological_Process

GO:0001938 positive regulation of endothelial cell proliferation http://amigo.geneontology.org/amigo/term/GO:0001938 81 3 0.439132577624446 6.83164983164983 0.00954729862816539 0.123023323063735 2260;3357;6774 STAT3;FGFR1;HTR2B geneontology_Biological_Process

GO:0009749 response to glucose http://amigo.geneontology.org/amigo/term/GO:0009749 155 4 0.840315426318384 4.76011730205279 0.00977257365805162 0.125723688764838 150;836;3939;5467 CASP3;ADRA2A;LDHA;PPARD geneontology_Biological_Process

GO:0018105 peptidyl-serine phosphorylation http://amigo.geneontology.org/amigo/term/GO:0018105 243 5 1.31739773287334 3.79536101758324 0.00993804207501814 0.127202465082969 596;2932;3320;5594;9261 GSK3B;MAPKAPK2;BCL2;MAPK1;HSP90AA1 geneontology_Biological_Process

GO:0032228 regulation of synaptic transmission, GABAergic http://amigo.geneontology.org/amigo/term/GO:0032228 28 2 0.151798915722031 13.1753246753247 0.0099670014505151 0.127202465082969 760;1268 CNR1;CA2 geneontology_Biological_Process

GO:0001844 protein insertion into mitochondrial membrane involved in apoptotic signaling pathway http://amigo.geneontology.org/amigo/term/GO:0001844 28 2 0.151798915722031 13.1753246753247 0.0099670014505151 0.127202465082969 596;841 BCL2;CASP8 geneontology_Biological_Process

GO:1902253 regulation of intrinsic apoptotic signaling pathway by p53 class mediator http://amigo.geneontology.org/amigo/term/GO:1902253 28 2 0.151798915722031 13.1753246753247 0.0099670014505151 0.127202465082969 596;4193 BCL2;MDM2 geneontology_Biological_Process

GO:0055026 negative regulation of cardiac muscle tissue development http://amigo.geneontology.org/amigo/term/GO:0055026 28 2 0.151798915722031 13.1753246753247 0.0099670014505151 0.127202465082969 5465;5950 PPARA;RBP4 geneontology_Biological_Process

GO:0090130 tissue migration http://amigo.geneontology.org/amigo/term/GO:0090130 244 5 1.32281912272055 3.77980625931446 0.010104765650198 0.128550611658004 2260;5467;5468;5641;5747 PPARG;PTK2;FGFR1;LGMN;PPARD geneontology_Biological_Process

GO:0040013 negative regulation of locomotion http://amigo.geneontology.org/amigo/term/GO:0040013 244 5 1.32281912272055 3.77980625931446 0.010104765650198 0.128550611658004 140;596;5467;5468;6774 PPARG;ADORA3;BCL2;STAT3;PPARD geneontology_Biological_Process

GO:0006163 purine nucleotide metabolic process http://amigo.geneontology.org/amigo/term/GO:0006163 343 6 1.85953671759487 3.22661012456931 0.0102735167289461 0.130489969627026 136;3939;5141;5465;6319;6774 PPARA;ADORA2B;PDE4A;STAT3;LDHA;SCD geneontology_Biological_Process

GO:0006140 regulation of nucleotide metabolic process http://amigo.geneontology.org/amigo/term/GO:0006140 84 3 0.455396747166092 6.58766233766234 0.0105415794964673 0.132972340711717 136;5465;6774 PPARA;ADORA2B;STAT3 geneontology_Biological_Process

GO:0007631 feeding behavior http://amigo.geneontology.org/amigo/term/GO:0007631 84 3 0.455396747166092 6.58766233766234 0.0105415794964673 0.132972340711717 1268;5972;6774 REN;CNR1;STAT3 geneontology_Biological_Process

GO:1901800 positive regulation of proteasomal protein catabolic process http://amigo.geneontology.org/amigo/term/GO:1901800 84 3 0.455396747166092 6.58766233766234 0.0105415794964673 0.132972340711717 2932;4193;9817 GSK3B;KEAP1;MDM2 geneontology_Biological_Process

GO:0009746 response to hexose http://amigo.geneontology.org/amigo/term/GO:0009746 159 4 0.862000985707245 4.64036592338479 0.0106612047059876 0.132972340711717 150;836;3939;5467 CASP3;ADRA2A;LDHA;PPARD geneontology_Biological_Process

GO:0034694 response to prostaglandin http://amigo.geneontology.org/amigo/term/GO:0034694 29 2 0.157220305569246 12.7210031347962 0.0106683632513025 0.132972340711717 5468;8644 PPARG;AKR1C3 geneontology_Biological_Process

GO:0010039 response to iron ion http://amigo.geneontology.org/amigo/term/GO:0010039 29 2 0.157220305569246 12.7210031347962 0.0106683632513025 0.132972340711717 596;4193 BCL2;MDM2 geneontology_Biological_Process

GO:0010742 macrophage derived foam cell differentiation http://amigo.geneontology.org/amigo/term/GO:0010742 29 2 0.157220305569246 12.7210031347962 0.0106683632513025 0.132972340711717 5465;5468 PPARG;PPARA geneontology_Biological_Process

GO:0090077 foam cell differentiation http://amigo.geneontology.org/amigo/term/GO:0090077 29 2 0.157220305569246 12.7210031347962 0.0106683632513025 0.132972340711717 5465;5468 PPARG;PPARA geneontology_Biological_Process

GO:0045777 positive regulation of blood pressure http://amigo.geneontology.org/amigo/term/GO:0045777 29 2 0.157220305569246 12.7210031347962 0.0106683632513025 0.132972340711717 1268;1636 ACE;CNR1 geneontology_Biological_Process

GO:0032148 activation of protein kinase B activity http://amigo.geneontology.org/amigo/term/GO:0032148 29 2 0.157220305569246 12.7210031347962 0.0106683632513025 0.132972340711717 150;152 ADRA2A;ADRA2C geneontology_Biological_Process

GO:0043029 T cell homeostasis http://amigo.geneontology.org/amigo/term/GO:0043029 29 2 0.157220305569246 12.7210031347962 0.0106683632513025 0.132972340711717 596;836 CASP3;BCL2 geneontology_Biological_Process

GO:0051204 protein insertion into mitochondrial membrane http://amigo.geneontology.org/amigo/term/GO:0051204 29 2 0.157220305569246 12.7210031347962 0.0106683632513025 0.132972340711717 596;841 BCL2;CASP8 geneontology_Biological_Process

GO:0051952 regulation of amine transport http://amigo.geneontology.org/amigo/term/GO:0051952 85 3 0.460818137013307 6.51016042780749 0.0108858779333345 0.134910203288281 150;152;1268 CNR1;ADRA2A;ADRA2C geneontology_Biological_Process

GO:0032368 regulation of lipid transport http://amigo.geneontology.org/amigo/term/GO:0032368 85 3 0.460818137013307 6.51016042780749 0.0108858779333345 0.134910203288281 5465;5468;5972 REN;PPARG;PPARA geneontology_Biological_Process

GO:0051099 positive regulation of binding http://amigo.geneontology.org/amigo/term/GO:0051099 160 4 0.86742237555446 4.61136363636364 0.0108912760965045 0.134910203288281 1636;2932;5468;5914 ACE;PPARG;GSK3B;RARA geneontology_Biological_Process

GO:0002064 epithelial cell development http://amigo.geneontology.org/amigo/term/GO:0002064 160 4 0.86742237555446 4.61136363636364 0.0108912760965045 0.134910203288281 2260;2932;5914;6098 GSK3B;FGFR1;RARA;ROS1 geneontology_Biological_Process

GO:0044282 small molecule catabolic process http://amigo.geneontology.org/amigo/term/GO:0044282 249 5 1.34992607195663 3.70390653523184 0.0109674335272283 0.13563491055007 1268;2166;5465;5467;8644 PPARA;FAAH;CNR1;AKR1C3;PPARD geneontology_Biological_Process

GO:0032386 regulation of intracellular transport http://amigo.geneontology.org/amigo/term/GO:0032386 348 6 1.88664366683095 3.18025078369906 0.0109836818340971 0.13563491055007 136;1268;2932;4193;5594;23621 BACE1;GSK3B;CNR1;ADORA2B;MAPK1;MDM2 geneontology_Biological_Process

GO:0009145 purine nucleoside triphosphate biosynthetic process http://amigo.geneontology.org/amigo/term/GO:0009145 86 3 0.466239526860522 6.43446088794926 0.0112366501605425 0.137907476356843 3939;5465;6774 PPARA;STAT3;LDHA geneontology_Biological_Process

GO:0009206 purine ribonucleoside triphosphate biosynthetic process http://amigo.geneontology.org/amigo/term/GO:0009206 86 3 0.466239526860522 6.43446088794926 0.0112366501605425 0.137907476356843 3939;5465;6774 PPARA;STAT3;LDHA geneontology_Biological_Process

GO:0043467 regulation of generation of precursor metabolites and energy http://amigo.geneontology.org/amigo/term/GO:0043467 86 3 0.466239526860522 6.43446088794926 0.0112366501605425 0.137907476356843 2932;5465;6774 PPARA;GSK3B;STAT3 geneontology_Biological_Process

GO:0001657 ureteric bud development http://amigo.geneontology.org/amigo/term/GO:0001657 86 3 0.466239526860522 6.43446088794926 0.0112366501605425 0.137907476356843 596;2260;5914 BCL2;FGFR1;RARA geneontology_Biological_Process

GO:0006979 response to oxidative stress http://amigo.geneontology.org/amigo/term/GO:0006979 350 6 1.89748644652538 3.16207792207792 0.011277328983333 0.13810685578285 596;836;3939;4193;5594;8644 CASP3;AKR1C3;BCL2;MAPK1;MDM2;LDHA geneontology_Biological_Process

GO:0009152 purine ribonucleotide biosynthetic process http://amigo.geneontology.org/amigo/term/GO:0009152 162 4 0.878265155248891 4.55443322109989 0.0113610312216366 0.13810685578285 3939;5465;6319;6774 PPARA;STAT3;LDHA;SCD geneontology_Biological_Process

GO:0046474 glycerophospholipid biosynthetic process http://amigo.geneontology.org/amigo/term/GO:0046474 162 4 0.878265155248891 4.55443322109989 0.0113610312216366 0.13810685578285 43;2171;2260;3357 ACHE;FABP5;FGFR1;HTR2B geneontology_Biological_Process

GO:0043009 chordate embryonic development http://amigo.geneontology.org/amigo/term/GO:0043009 458 7 2.48299655002464 2.81917427550615 0.0113620095491394 0.13810685578285 841;2260;5594;5914;5950;6256;9817 KEAP1;MAPK1;CASP8;RXRA;FGFR1;RBP4;RARA geneontology_Biological_Process

GO:0019934 cGMP-mediated signaling http://amigo.geneontology.org/amigo/term/GO:0019934 30 2 0.162641695416461 12.2969696969697 0.0113909678601201 0.13810685578285 136;3357 ADORA2B;HTR2B geneontology_Biological_Process

GO:0043276 anoikis http://amigo.geneontology.org/amigo/term/GO:0043276 30 2 0.162641695416461 12.2969696969697 0.0113909678601201 0.13810685578285 596;5747 PTK2;BCL2 geneontology_Biological_Process

GO:0071470 cellular response to osmotic stress http://amigo.geneontology.org/amigo/term/GO:0071470 30 2 0.162641695416461 12.2969696969697 0.0113909678601201 0.13810685578285 231;836 CASP3;AKR1B1 geneontology_Biological_Process

GO:0033028 myeloid cell apoptotic process http://amigo.geneontology.org/amigo/term/GO:0033028 30 2 0.162641695416461 12.2969696969697 0.0113909678601201 0.13810685578285 596;7067 BCL2;THRA geneontology_Biological_Process

GO:0051235 maintenance of location http://amigo.geneontology.org/amigo/term/GO:0051235 252 5 1.36619024149828 3.65981240981241 0.0115086470388697 0.139322531928949 3357;5465;5467;5468;9817 PPARG;PPARA;KEAP1;HTR2B;PPARD geneontology_Biological_Process

GO:0046632 alpha-beta T cell differentiation http://amigo.geneontology.org/amigo/term/GO:0046632 87 3 0.471660916707738 6.36050156739812 0.0115939176162205 0.139720675850899 596;5914;6774 BCL2;STAT3;RARA geneontology_Biological_Process

GO:0072163 mesonephric epithelium development http://amigo.geneontology.org/amigo/term/GO:0072163 87 3 0.471660916707738 6.36050156739812 0.0115939176162205 0.139720675850899 596;2260;5914 BCL2;FGFR1;RARA geneontology_Biological_Process

GO:0072164 mesonephric tubule development http://amigo.geneontology.org/amigo/term/GO:0072164 87 3 0.471660916707738 6.36050156739812 0.0115939176162205 0.139720675850899 596;2260;5914 BCL2;FGFR1;RARA geneontology_Biological_Process

GO:0034284 response to monosaccharide http://amigo.geneontology.org/amigo/term/GO:0034284 164 4 0.889107934943322 4.49889135254989 0.0118437103918456 0.142516346700073 150;836;3939;5467 CASP3;ADRA2A;LDHA;PPARD geneontology_Biological_Process

GO:0018209 peptidyl-serine modification http://amigo.geneontology.org/amigo/term/GO:0018209 254 5 1.37703302119271 3.63099498926271 0.0118794648951557 0.142531931660864 596;2932;3320;5594;9261 GSK3B;MAPKAPK2;BCL2;MAPK1;HSP90AA1 geneontology_Biological_Process

GO:0055086 nucleobase-containing small molecule metabolic process http://amigo.geneontology.org/amigo/term/GO:0055086 462 7 2.5046821094135 2.79476584022039 0.0118806296448133 0.142531931660864 136;3939;5141;5465;5594;6319;6774 PPARA;ADORA2B;PDE4A;MAPK1;STAT3;LDHA;SCD geneontology_Biological_Process

GO:0015837 amine transport http://amigo.geneontology.org/amigo/term/GO:0015837 88 3 0.477082306554953 6.28822314049587 0.0119577008266021 0.143027686120284 150;152;1268 CNR1;ADRA2A;ADRA2C geneontology_Biological_Process

GO:1903409 reactive oxygen species biosynthetic process http://amigo.geneontology.org/amigo/term/GO:1903409 88 3 0.477082306554953 6.28822314049587 0.0119577008266021 0.143027686120284 1544;3320;6774 CYP1A2;STAT3;HSP90AA1 geneontology_Biological_Process

GO:0043434 response to peptide hormone http://amigo.geneontology.org/amigo/term/GO:0043434 355 6 1.92459339576146 3.11754161331626 0.012035877432057 0.14342814403925 760;4193;5465;5468;5747;6774 PPARG;PPARA;PTK2;STAT3;CA2;MDM2 geneontology_Biological_Process

GO:0001894 tissue homeostasis http://amigo.geneontology.org/amigo/term/GO:0001894 165 4 0.894529324790537 4.47162534435262 0.0120899361136471 0.14342814403925 231;596;760;5950 AKR1B1;BCL2;CA2;RBP4 geneontology_Biological_Process

GO:0071222 cellular response to lipopolysaccharide http://amigo.geneontology.org/amigo/term/GO:0071222 165 4 0.894529324790537 4.47162534435262 0.0120899361136471 0.14342814403925 834;5467;5594;5914 MAPK1;CASP1;PPARD;RARA geneontology_Biological_Process

GO:0014072 response to isoquinoline alkaloid http://amigo.geneontology.org/amigo/term/GO:0014072 31 2 0.168063085263677 11.900293255132 0.01213457304606 0.14342814403925 1268;4193 CNR1;MDM2 geneontology_Biological_Process

GO:0043278 response to morphine http://amigo.geneontology.org/amigo/term/GO:0043278 31 2 0.168063085263677 11.900293255132 0.01213457304606 0.14342814403925 1268;4193 CNR1;MDM2 geneontology_Biological_Process

GO:0042987 amyloid precursor protein catabolic process http://amigo.geneontology.org/amigo/term/GO:0042987 31 2 0.168063085263677 11.900293255132 0.01213457304606 0.14342814403925 836;23385 CASP3;NCSTN geneontology_Biological_Process

GO:0071312 cellular response to alkaloid http://amigo.geneontology.org/amigo/term/GO:0071312 31 2 0.168063085263677 11.900293255132 0.01213457304606 0.14342814403925 836;4193 CASP3;MDM2 geneontology_Biological_Process

GO:0010712 regulation of collagen metabolic process http://amigo.geneontology.org/amigo/term/GO:0010712 31 2 0.168063085263677 11.900293255132 0.01213457304606 0.14342814403925 5467;5468 PPARG;PPARD geneontology_Biological_Process

GO:0071375 cellular response to peptide hormone stimulus http://amigo.geneontology.org/amigo/term/GO:0071375 256 5 1.38787580088714 3.60262784090909 0.0122583836261887 0.144677855127967 760;4193;5468;5747;6774 PPARG;PTK2;STAT3;CA2;MDM2 geneontology_Biological_Process

GO:0030218 erythrocyte differentiation http://amigo.geneontology.org/amigo/term/GO:0030218 89 3 0.482503696402169 6.21756894790603 0.0123280194173192 0.144992949921495 836;6774;7067 CASP3;STAT3;THRA geneontology_Biological_Process

GO:0010810 regulation of cell-substrate adhesion http://amigo.geneontology.org/amigo/term/GO:0010810 166 4 0.899950714637753 4.4446878422782 0.0123394400020668 0.144992949921495 596;2932;5328;5747 GSK3B;PTK2;BCL2;PLAU geneontology_Biological_Process

GO:1901617 organic hydroxy compound biosynthetic process http://amigo.geneontology.org/amigo/term/GO:1901617 166 4 0.899950714637753 4.4446878422782 0.0123394400020668 0.144992949921495 231;239;6319;8644 AKR1B1;ALOX12;AKR1C3;SCD geneontology_Biological_Process

GO:1901361 organic cyclic compound catabolic process http://amigo.geneontology.org/amigo/term/GO:1901361 466 7 2.52636766880237 2.77077643386656 0.0124163335103491 0.145682552419081 836;1544;3939;5141;5465;6774;9261 CASP3;PPARA;PDE4A;CYP1A2;MAPKAPK2;STAT3;LDHA geneontology_Biological_Process

GO:0032103 positive regulation of response to external stimulus http://amigo.geneontology.org/amigo/term/GO:0032103 257 5 1.39329719073435 3.58860983374602 0.0124509056663691 0.145874300354737 136;1268;2167;2260;5641 CNR1;ADORA2B;FGFR1;LGMN;FABP4 geneontology_Biological_Process

GO:0048863 stem cell differentiation http://amigo.geneontology.org/amigo/term/GO:0048863 167 4 0.905372104484968 4.41807294501905 0.0125922373699763 0.147314449465717 1636;3357;5594;6774 ACE;MAPK1;STAT3;HTR2B geneontology_Biological_Process

GO:0009132 nucleoside diphosphate metabolic process http://amigo.geneontology.org/amigo/term/GO:0009132 90 3 0.487925086249384 6.14848484848485 0.0127048921245914 0.148199047785685 3939;5465;6774 PPARA;STAT3;LDHA geneontology_Biological_Process

GO:0009201 ribonucleoside triphosphate biosynthetic process http://amigo.geneontology.org/amigo/term/GO:0009201 90 3 0.487925086249384 6.14848484848485 0.0127048921245914 0.148199047785685 3939;5465;6774 PPARA;STAT3;LDHA geneontology_Biological_Process

GO:0002757 immune response-activating signal transduction http://amigo.geneontology.org/amigo/term/GO:0002757 360 6 1.95170034499754 3.07424242424242 0.0128300017481728 0.149440573491818 596;841;3320;5594;5747;9261 PTK2;MAPKAPK2;BCL2;MAPK1;HSP90AA1;CASP8 geneontology_Biological_Process

GO:0001990 regulation of systemic arterial blood pressure by hormone http://amigo.geneontology.org/amigo/term/GO:0001990 32 2 0.173484475110892 11.5284090909091 0.0128989384967487 0.149590298334758 1636;5972 ACE;REN geneontology_Biological_Process

GO:0043368 positive T cell selection http://amigo.geneontology.org/amigo/term/GO:0043368 32 2 0.173484475110892 11.5284090909091 0.0128989384967487 0.149590298334758 596;6774 BCL2;STAT3 geneontology_Biological_Process

GO:0051973 positive regulation of telomerase activity http://amigo.geneontology.org/amigo/term/GO:0051973 32 2 0.173484475110892 11.5284090909091 0.0128989384967487 0.149590298334758 3320;5594 MAPK1;HSP90AA1 geneontology_Biological_Process

GO:0009792 embryo development ending in birth or egg hatching http://amigo.geneontology.org/amigo/term/GO:0009792 470 7 2.54805322819123 2.74719535783366 0.0129694294370655 0.150190122077276 841;2260;5594;5914;5950;6256;9817 KEAP1;MAPK1;CASP8;RXRA;FGFR1;RBP4;RARA geneontology_Biological_Process

GO:0032091 negative regulation of protein binding http://amigo.geneontology.org/amigo/term/GO:0032091 91 3 0.493346476096599 6.08091908091908 0.0130883368063144 0.150918558392315 1636;2932;5465 ACE;PPARA;GSK3B geneontology_Biological_Process

GO:0006090 pyruvate metabolic process http://amigo.geneontology.org/amigo/term/GO:0006090 91 3 0.493346476096599 6.08091908091908 0.0130883368063144 0.150918558392315 3939;5465;6774 PPARA;STAT3;LDHA geneontology_Biological_Process

GO:0010001 glial cell differentiation http://amigo.geneontology.org/amigo/term/GO:0010001 169 4 0.916214884179399 4.36578805809575 0.013107772817128 0.150918558392315 5468;5594;6774;83933 PPARG;MAPK1;STAT3;HDAC10 geneontology_Biological_Process

GO:0099504 synaptic vesicle cycle http://amigo.geneontology.org/amigo/term/GO:0099504 169 4 0.916214884179399 4.36578805809575 0.013107772817128 0.150918558392315 136;1268;2932;23621 BACE1;GSK3B;CNR1;ADORA2B geneontology_Biological_Process

GO:0072521 purine-containing compound metabolic process http://amigo.geneontology.org/amigo/term/GO:0072521 362 6 1.96254312469197 3.0572576594676 0.0131577998706528 0.151276888742764 136;3939;5141;5465;6319;6774 PPARA;ADORA2B;PDE4A;STAT3;LDHA;SCD geneontology_Biological_Process

GO:0050678 regulation of epithelial cell proliferation http://amigo.geneontology.org/amigo/term/GO:0050678 261 5 1.41498275012321 3.53361198188784 0.0132416405761477 0.152022392956003 2260;3357;5467;5468;6774 PPARG;STAT3;FGFR1;HTR2B;PPARD geneontology_Biological_Process

GO:2000278 regulation of DNA biosynthetic process http://amigo.geneontology.org/amigo/term/GO:2000278 92 3 0.498767865943815 6.01482213438735 0.0134783704530491 0.154518510552004 3320;5468;5594 PPARG;MAPK1;HSP90AA1 geneontology_Biological_Process

GO:0043523 regulation of neuron apoptotic process http://amigo.geneontology.org/amigo/term/GO:0043523 171 4 0.927057663873829 4.31472620946305 0.0136366610369069 0.155096280586163 596;836;5641;23621 CASP3;BACE1;BCL2;LGMN geneontology_Biological_Process

GO:0009260 ribonucleotide biosynthetic process http://amigo.geneontology.org/amigo/term/GO:0009260 171 4 0.927057663873829 4.31472620946305 0.0136366610369069 0.155096280586163 3939;5465;6319;6774 PPARA;STAT3;LDHA;SCD geneontology_Biological_Process

GO:0048732 gland development http://amigo.geneontology.org/amigo/term/GO:0048732 365 6 1.97880729423361 3.03212951432129 0.0136605355561086 0.155096280586163 596;2260;5594;5914;6256;7067 BCL2;MAPK1;RXRA;FGFR1;RARA;THRA geneontology_Biological_Process

GO:0046688 response to copper ion http://amigo.geneontology.org/amigo/term/GO:0046688 33 2 0.178905864958107 11.1790633608815 0.0136838258052776 0.155096280586163 1544;23621 BACE1;CYP1A2 geneontology_Biological_Process

GO:0007618 mating http://amigo.geneontology.org/amigo/term/GO:0007618 33 2 0.178905864958107 11.1790633608815 0.0136838258052776 0.155096280586163 1268;7067 CNR1;THRA geneontology_Biological_Process

GO:0014911 positive regulation of smooth muscle cell migration http://amigo.geneontology.org/amigo/term/GO:0014911 33 2 0.178905864958107 11.1790633608815 0.0136838258052776 0.155096280586163 596;4193 BCL2;MDM2 geneontology_Biological_Process

GO:0000060 protein import into nucleus, translocation http://amigo.geneontology.org/amigo/term/GO:0000060 33 2 0.178905864958107 11.1790633608815 0.0136838258052776 0.155096280586163 5594;8644 AKR1C3;MAPK1 geneontology_Biological_Process

GO:0032964 collagen biosynthetic process http://amigo.geneontology.org/amigo/term/GO:0032964 33 2 0.178905864958107 11.1790633608815 0.0136838258052776 0.155096280586163 5467;5468 PPARG;PPARD geneontology_Biological_Process

GO:0043542 endothelial cell migration http://amigo.geneontology.org/amigo/term/GO:0043542 172 4 0.932479053721045 4.28964059196617 0.0139061486318308 0.15717090586428 2260;5468;5641;5747 PPARG;PTK2;FGFR1;LGMN geneontology_Biological_Process

GO:0071219 cellular response to molecule of bacterial origin http://amigo.geneontology.org/amigo/term/GO:0071219 172 4 0.932479053721045 4.28964059196617 0.0139061486318308 0.15717090586428 834;5467;5594;5914 MAPK1;CASP1;PPARD;RARA geneontology_Biological_Process

GO:0050769 positive regulation of neurogenesis http://amigo.geneontology.org/amigo/term/GO:0050769 367 6 1.98965007392804 3.01560564775824 0.0140031280441366 0.158043766726631 152;596;1268;2260;5468;5914 PPARG;CNR1;ADRA2C;BCL2;FGFR1;RARA geneontology_Biological_Process

GO:1903829 positive regulation of cellular protein localization http://amigo.geneontology.org/amigo/term/GO:1903829 265 5 1.43666830951207 3.48027444253859 0.0140658735078822 0.158528337760667 596;841;2932;4193;5594 GSK3B;BCL2;MAPK1;CASP8;MDM2 geneontology_Biological_Process

GO:0003012 muscle system process http://amigo.geneontology.org/amigo/term/GO:0003012 368 6 1.99507146377526 3.00741106719368 0.0141766744021262 0.159354631612429 136;150;152;3357;5465;5747 PPARA;ADORA2B;ADRA2A;ADRA2C;PTK2;HTR2B geneontology_Biological_Process

GO:0002573 myeloid leukocyte differentiation http://amigo.geneontology.org/amigo/term/GO:0002573 173 4 0.93790044356826 4.26484498160799 0.0141790174591414 0.159354631612429 760;841;5468;5914 PPARG;CASP8;CA2;RARA geneontology_Biological_Process

GO:0018107 peptidyl-threonine phosphorylation http://amigo.geneontology.org/amigo/term/GO:0018107 94 3 0.509610645638245 5.88684719535783 0.0142782683323484 0.160020592710716 596;2932;5594 GSK3B;BCL2;MAPK1 geneontology_Biological_Process

GO:0055067 monovalent inorganic cation homeostasis http://amigo.geneontology.org/amigo/term/GO:0055067 94 3 0.509610645638245 5.88684719535783 0.0142782683323484 0.160020592710716 596;760;5594 BCL2;MAPK1;CA2 geneontology_Biological_Process

GO:1903050 regulation of proteolysis involved in cellular protein catabolic process http://amigo.geneontology.org/amigo/term/GO:1903050 174 4 0.943321833415476 4.24033437826541 0.014455281453021 0.16102911896476 2932;4193;5747;9817 GSK3B;KEAP1;PTK2;MDM2 geneontology_Biological_Process

GO:2000273 positive regulation of signaling receptor activity http://amigo.geneontology.org/amigo/term/GO:2000273 34 2 0.184327254805323 10.8502673796791 0.0144889984572141 0.16102911896476 150;152 ADRA2A;ADRA2C geneontology_Biological_Process

GO:0060416 response to growth hormone http://amigo.geneontology.org/amigo/term/GO:0060416 34 2 0.184327254805323 10.8502673796791 0.0144889984572141 0.16102911896476 5747;6774 PTK2;STAT3 geneontology_Biological_Process

GO:0019048 modulation by virus of host morphology or physiology http://amigo.geneontology.org/amigo/term/GO:0019048 34 2 0.184327254805323 10.8502673796791 0.0144889984572141 0.16102911896476 841;6256 CASP8;RXRA geneontology_Biological_Process

GO:0046825 regulation of protein export from nucleus http://amigo.geneontology.org/amigo/term/GO:0046825 34 2 0.184327254805323 10.8502673796791 0.0144889984572141 0.16102911896476 2932;4193 GSK3B;MDM2 geneontology_Biological_Process

GO:0060043 regulation of cardiac muscle cell proliferation http://amigo.geneontology.org/amigo/term/GO:0060043 34 2 0.184327254805323 10.8502673796791 0.0144889984572141 0.16102911896476 2260;5950 FGFR1;RBP4 geneontology_Biological_Process

GO:0008637 apoptotic mitochondrial changes http://amigo.geneontology.org/amigo/term/GO:0008637 95 3 0.515032035485461 5.82488038277512 0.0146881623068551 0.162790408281793 596;841;2932 GSK3B;BCL2;CASP8 geneontology_Biological_Process

GO:0010822 positive regulation of mitochondrion organization http://amigo.geneontology.org/amigo/term/GO:0010822 95 3 0.515032035485461 5.82488038277512 0.0146881623068551 0.162790408281793 596;841;2932 GSK3B;BCL2;CASP8 geneontology_Biological_Process

GO:0045017 glycerolipid biosynthetic process http://amigo.geneontology.org/amigo/term/GO:0045017 175 4 0.948743223262691 4.2161038961039 0.0147349543491893 0.162857879422945 43;2171;2260;3357 ACHE;FABP5;FGFR1;HTR2B geneontology_Biological_Process

GO:0009913 epidermal cell differentiation http://amigo.geneontology.org/amigo/term/GO:0009913 175 4 0.948743223262691 4.2161038961039 0.0147349543491893 0.162857879422945 836;2260;8644;9817 CASP3;AKR1C3;KEAP1;FGFR1 geneontology_Biological_Process

GO:0001701 in utero embryonic development http://amigo.geneontology.org/amigo/term/GO:0001701 269 5 1.45835386890094 3.42852314971274 0.0149242096353577 0.164722104140872 841;2260;5594;6256;9817 KEAP1;MAPK1;CASP8;RXRA;FGFR1 geneontology_Biological_Process

GO:0046390 ribose phosphate biosynthetic process http://amigo.geneontology.org/amigo/term/GO:0046390 176 4 0.954164613109906 4.19214876033058 0.0150180496846091 0.165301834355216 3939;5465;6319;6774 PPARA;STAT3;LDHA;SCD geneontology_Biological_Process

GO:0099003 vesicle-mediated transport in synapse http://amigo.geneontology.org/amigo/term/GO:0099003 176 4 0.954164613109906 4.19214876033058 0.0150180496846091 0.165301834355216 136;1268;2932;23621 BACE1;GSK3B;CNR1;ADORA2B geneontology_Biological_Process

GO:0008203 cholesterol metabolic process http://amigo.geneontology.org/amigo/term/GO:0008203 96 3 0.520453425332676 5.76420454545454 0.0151047047515418 0.165799516353687 5467;6256;6319 RXRA;PPARD;SCD geneontology_Biological_Process

GO:0032675 regulation of interleukin-6 production http://amigo.geneontology.org/amigo/term/GO:0032675 96 3 0.520453425332676 5.76420454545454 0.0151047047515418 0.165799516353687 136;6774;9261 ADORA2B;MAPKAPK2;STAT3 geneontology_Biological_Process

GO:0048705 skeletal system morphogenesis http://amigo.geneontology.org/amigo/term/GO:0048705 177 4 0.959586002957122 4.16846430405752 0.0153045807972187 0.166954227500263 249;2260;5914;7067 ALPL;FGFR1;RARA;THRA geneontology_Biological_Process

GO:0051896 regulation of protein kinase B signaling http://amigo.geneontology.org/amigo/term/GO:0051896 177 4 0.959586002957122 4.16846430405752 0.0153045807972187 0.166954227500263 2260;3320;5747;8644 AKR1C3;PTK2;HSP90AA1;FGFR1 geneontology_Biological_Process

GO:0046470 phosphatidylcholine metabolic process http://amigo.geneontology.org/amigo/term/GO:0046470 35 2 0.189748644652538 10.5402597402597 0.0153142218176947 0.166954227500263 43;2171 ACHE;FABP5 geneontology_Biological_Process

GO:0030810 positive regulation of nucleotide biosynthetic process http://amigo.geneontology.org/amigo/term/GO:0030810 35 2 0.189748644652538 10.5402597402597 0.0153142218176947 0.166954227500263 136;6774 ADORA2B;STAT3 geneontology_Biological_Process

GO:1900373 positive regulation of purine nucleotide biosynthetic process http://amigo.geneontology.org/amigo/term/GO:1900373 35 2 0.189748644652538 10.5402597402597 0.0153142218176947 0.166954227500263 136;6774 ADORA2B;STAT3 geneontology_Biological_Process

GO:0055007 cardiac muscle cell differentiation http://amigo.geneontology.org/amigo/term/GO:0055007 97 3 0.525874815179892 5.70477975632615 0.015527908481642 0.168594740393622 5465;5914;6256 PPARA;RXRA;RARA geneontology_Biological_Process

GO:0009142 nucleoside triphosphate biosynthetic process http://amigo.geneontology.org/amigo/term/GO:0009142 97 3 0.525874815179892 5.70477975632615 0.015527908481642 0.168594740393622 3939;5465;6774 PPARA;STAT3;LDHA geneontology_Biological_Process

GO:0015698 inorganic anion transport http://amigo.geneontology.org/amigo/term/GO:0015698 97 3 0.525874815179892 5.70477975632615 0.015527908481642 0.168594740393622 760;2260;6098 CA2;FGFR1;ROS1 geneontology_Biological_Process

GO:0031589 cell-substrate adhesion http://amigo.geneontology.org/amigo/term/GO:0031589 272 5 1.47461803844258 3.39070855614973 0.0155906971185547 0.168818346877773 596;2932;5328;5467;5747 GSK3B;PTK2;BCL2;PLAU;PPARD geneontology_Biological_Process

GO:0031098 stress-activated protein kinase signaling cascade http://amigo.geneontology.org/amigo/term/GO:0031098 272 5 1.47461803844258 3.39070855614973 0.0155906971185547 0.168818346877773 136;231;5594;9020;9261 AKR1B1;ADORA2B;MAP3K14;MAPKAPK2;MAPK1 geneontology_Biological_Process

GO:0031644 regulation of neurological system process http://amigo.geneontology.org/amigo/term/GO:0031644 98 3 0.531296205027107 5.64656771799629 0.015957785508911 0.17163198876654 152;2171;11343 ADRA2C;MGLL;FABP5 geneontology_Biological_Process

GO:0009127 purine nucleoside monophosphate biosynthetic process http://amigo.geneontology.org/amigo/term/GO:0009127 98 3 0.531296205027107 5.64656771799629 0.015957785508911 0.17163198876654 3939;5465;6774 PPARA;STAT3;LDHA geneontology_Biological_Process

GO:0009168 purine ribonucleoside monophosphate biosynthetic process http://amigo.geneontology.org/amigo/term/GO:0009168 98 3 0.531296205027107 5.64656771799629 0.015957785508911 0.17163198876654 3939;5465;6774 PPARA;STAT3;LDHA geneontology_Biological_Process

GO:0018210 peptidyl-threonine modification http://amigo.geneontology.org/amigo/term/GO:0018210 98 3 0.531296205027107 5.64656771799629 0.015957785508911 0.17163198876654 596;2932;5594 GSK3B;BCL2;MAPK1 geneontology_Biological_Process

GO:0034101 erythrocyte homeostasis http://amigo.geneontology.org/amigo/term/GO:0034101 98 3 0.531296205027107 5.64656771799629 0.015957785508911 0.17163198876654 836;6774;7067 CASP3;STAT3;THRA geneontology_Biological_Process

GO:0001974 blood vessel remodeling http://amigo.geneontology.org/amigo/term/GO:0001974 36 2 0.195170034499754 10.2474747474747 0.0161592631185976 0.173162655478495 1636;4193 ACE;MDM2 geneontology_Biological_Process

GO:0033003 regulation of mast cell activation http://amigo.geneontology.org/amigo/term/GO:0033003 36 2 0.195170034499754 10.2474747474747 0.0161592631185976 0.173162655478495 136;1268 CNR1;ADORA2B geneontology_Biological_Process

GO:0017038 protein import http://amigo.geneontology.org/amigo/term/GO:0017038 180 4 0.975850172498768 4.0989898989899 0.016184919187322 0.173162655478495 3320;5594;6774;8644 AKR1C3;MAPK1;STAT3;HSP90AA1 geneontology_Biological_Process

GO:0042110 T cell activation http://amigo.geneontology.org/amigo/term/GO:0042110 379 6 2.05470675209463 2.92012473015112 0.0161866616218338 0.173162655478495 596;836;841;5914;6774;23385 CASP3;NCSTN;BCL2;STAT3;CASP8;RARA geneontology_Biological_Process

GO:0050728 negative regulation of inflammatory response http://amigo.geneontology.org/amigo/term/GO:0050728 99 3 0.536717594874322 5.58953168044077 0.0163943470519299 0.174451549347796 5465;5467;5468 PPARG;PPARA;PPARD geneontology_Biological_Process

GO:0042476 odontogenesis http://amigo.geneontology.org/amigo/term/GO:0042476 99 3 0.536717594874322 5.58953168044077 0.0163943470519299 0.174451549347796 249;760;5465 PPARA;ALPL;CA2 geneontology_Biological_Process

GO:0046822 regulation of nucleocytoplasmic transport http://amigo.geneontology.org/amigo/term/GO:0046822 99 3 0.536717594874322 5.58953168044077 0.0163943470519299 0.174451549347796 2932;4193;5594 GSK3B;MAPK1;MDM2 geneontology_Biological_Process

GO:0002761 regulation of myeloid leukocyte differentiation http://amigo.geneontology.org/amigo/term/GO:0002761 99 3 0.536717594874322 5.58953168044077 0.0163943470519299 0.174451549347796 760;841;5914 CASP8;CA2;RARA geneontology_Biological_Process

GO:0046660 female sex differentiation http://amigo.geneontology.org/amigo/term/GO:0046660 100 3 0.542138984721538 5.53363636363636 0.0168376035463158 0.178930283635616 596;836;5950 CASP3;BCL2;RBP4 geneontology_Biological_Process

GO:0010632 regulation of epithelial cell migration http://amigo.geneontology.org/amigo/term/GO:0010632 183 4 0.992114342040414 4.03179334326875 0.017096640585934 0.180962060805084 2260;5468;5641;5747 PPARG;PTK2;FGFR1;LGMN geneontology_Biological_Process

GO:0007160 cell-matrix adhesion http://amigo.geneontology.org/amigo/term/GO:0007160 183 4 0.992114342040414 4.03179334326875 0.017096640585934 0.180962060805084 596;2932;5328;5747 GSK3B;PTK2;BCL2;PLAU geneontology_Biological_Process

GO:0009743 response to carbohydrate http://amigo.geneontology.org/amigo/term/GO:0009743 183 4 0.992114342040414 4.03179334326875 0.017096640585934 0.180962060805084 150;836;3939;5467 CASP3;ADRA2A;LDHA;PPARD geneontology_Biological_Process

GO:0016125 sterol metabolic process http://amigo.geneontology.org/amigo/term/GO:0016125 102 3 0.552981764415968 5.42513368983957 0.0177442392774385 0.18682816144482 5467;6256;6319 RXRA;PPARD;SCD geneontology_Biological_Process

GO:0019362 pyridine nucleotide metabolic process http://amigo.geneontology.org/amigo/term/GO:0019362 102 3 0.552981764415968 5.42513368983957 0.0177442392774385 0.18682816144482 3939;5465;6774 PPARA;STAT3;LDHA geneontology_Biological_Process

GO:0046496 nicotinamide nucleotide metabolic process http://amigo.geneontology.org/amigo/term/GO:0046496 102 3 0.552981764415968 5.42513368983957 0.0177442392774385 0.18682816144482 3939;5465;6774 PPARA;STAT3;LDHA geneontology_Biological_Process

GO:0032434 regulation of proteasomal ubiquitin-dependent protein catabolic process http://amigo.geneontology.org/amigo/term/GO:0032434 102 3 0.552981764415968 5.42513368983957 0.0177442392774385 0.18682816144482 2932;4193;9817 GSK3B;KEAP1;MDM2 geneontology_Biological_Process

GO:0006110 regulation of glycolytic process http://amigo.geneontology.org/amigo/term/GO:0006110 38 2 0.206012814194184 9.70813397129187 0.0179078777264901 0.187563923517504 5465;6774 PPARA;STAT3 geneontology_Biological_Process

GO:0060711 labyrinthine layer development http://amigo.geneontology.org/amigo/term/GO:0060711 38 2 0.206012814194184 9.70813397129187 0.0179078777264901 0.187563923517504 841;5594 MAPK1;CASP8 geneontology_Biological_Process

GO:0051453 regulation of intracellular pH http://amigo.geneontology.org/amigo/term/GO:0051453 38 2 0.206012814194184 9.70813397129187 0.0179078777264901 0.187563923517504 596;760 BCL2;CA2 geneontology_Biological_Process

GO:0051205 protein insertion into membrane http://amigo.geneontology.org/amigo/term/GO:0051205 38 2 0.206012814194184 9.70813397129187 0.0179078777264901 0.187563923517504 596;841 BCL2;CASP8 geneontology_Biological_Process

GO:0045666 positive regulation of neuron differentiation http://amigo.geneontology.org/amigo/term/GO:0045666 282 5 1.52883193691474 3.27047066408769 0.0179563391713644 0.187752806103347 152;596;1268;2260;5914 CNR1;ADRA2C;BCL2;FGFR1;RARA geneontology_Biological_Process

GO:0002764 immune response-regulating signaling pathway http://amigo.geneontology.org/amigo/term/GO:0002764 388 6 2.10349926071957 2.85238987816307 0.0179728379749018 0.187752806103347 596;841;3320;5594;5747;9261 PTK2;MAPKAPK2;BCL2;MAPK1;HSP90AA1;CASP8 geneontology_Biological_Process

GO:0010675 regulation of cellular carbohydrate metabolic process http://amigo.geneontology.org/amigo/term/GO:0010675 103 3 0.558403154263184 5.37246248896734 0.0182076355611681 0.189463588765237 2932;5465;6774 PPARA;GSK3B;STAT3 geneontology_Biological_Process

GO:0032635 interleukin-6 production http://amigo.geneontology.org/amigo/term/GO:0032635 103 3 0.558403154263184 5.37246248896734 0.0182076355611681 0.189463588765237 136;6774;9261 ADORA2B;MAPKAPK2;STAT3 geneontology_Biological_Process

GO:0072655 establishment of protein localization to mitochondrion http://amigo.geneontology.org/amigo/term/GO:0072655 103 3 0.558403154263184 5.37246248896734 0.0182076355611681 0.189463588765237 596;841;3320 BCL2;HSP90AA1;CASP8 geneontology_Biological_Process

GO:0046887 positive regulation of hormone secretion http://amigo.geneontology.org/amigo/term/GO:0046887 104 3 0.563824544110399 5.32080419580419 0.0186777609100189 0.193693998544682 2260;5467;5950 FGFR1;PPARD;RBP4 geneontology_Biological_Process

GO:0044262 cellular carbohydrate metabolic process http://amigo.geneontology.org/amigo/term/GO:0044262 188 4 1.01922129127649 3.92456479690522 0.0186867991597719 0.193693998544682 231;2932;5465;6774 AKR1B1;PPARA;GSK3B;STAT3 geneontology_Biological_Process

GO:0045637 regulation of myeloid cell differentiation http://amigo.geneontology.org/amigo/term/GO:0045637 188 4 1.01922129127649 3.92456479690522 0.0186867991597719 0.193693998544682 760;841;5914;6774 STAT3;CASP8;CA2;RARA geneontology_Biological_Process

GO:0030811 regulation of nucleotide catabolic process http://amigo.geneontology.org/amigo/term/GO:0030811 39 2 0.2114342040414 9.45920745920746 0.0188109947166193 0.19397626252885 5465;6774 PPARA;STAT3 geneontology_Biological_Process

GO:0033628 regulation of cell adhesion mediated by integrin http://amigo.geneontology.org/amigo/term/GO:0033628 39 2 0.2114342040414 9.45920745920746 0.0188109947166193 0.19397626252885 5328;5747 PTK2;PLAU geneontology_Biological_Process

GO:0051932 synaptic transmission, GABAergic http://amigo.geneontology.org/amigo/term/GO:0051932 39 2 0.2114342040414 9.45920745920746 0.0188109947166193 0.19397626252885 760;1268 CNR1;CA2 geneontology_Biological_Process

GO:0045600 positive regulation of fat cell differentiation http://amigo.geneontology.org/amigo/term/GO:0045600 39 2 0.2114342040414 9.45920745920746 0.0188109947166193 0.19397626252885 5467;5468 PPARG;PPARD geneontology_Biological_Process

GO:0007596 blood coagulation http://amigo.geneontology.org/amigo/term/GO:0007596 286 5 1.5505174963036 3.22472981563891 0.0189659894287868 0.195071783302252 150;152;239;5328;5594 ALOX12;ADRA2A;ADRA2C;MAPK1;PLAU geneontology_Biological_Process

GO:0042692 muscle cell differentiation http://amigo.geneontology.org/amigo/term/GO:0042692 286 5 1.5505174963036 3.22472981563891 0.0189659894287868 0.195071783302252 596;836;5465;5914;6256 CASP3;PPARA;BCL2;RXRA;RARA geneontology_Biological_Process

GO:0033209 tumor necrosis factor-mediated signaling pathway http://amigo.geneontology.org/amigo/term/GO:0033209 105 3 0.569245933957615 5.27012987012987 0.0191546219946757 0.196506775899224 834;841;9020 MAP3K14;CASP8;CASP1 geneontology_Biological_Process

GO:0034250 positive regulation of cellular amide metabolic process http://amigo.geneontology.org/amigo/term/GO:0034250 105 3 0.569245933957615 5.27012987012987 0.0191546219946757 0.196506775899224 836;5594;6256 CASP3;MAPK1;RXRA geneontology_Biological_Process

GO:0072524 pyridine-containing compound metabolic process http://amigo.geneontology.org/amigo/term/GO:0072524 106 3 0.57466732380483 5.22041166380789 0.0196382247621715 0.200278068253219 3939;5465;6774 PPARA;STAT3;LDHA geneontology_Biological_Process

GO:0045776 negative regulation of blood pressure http://amigo.geneontology.org/amigo/term/GO:0045776 40 2 0.216855593888615 9.22272727272727 0.0197330169883521 0.200278068253219 1268;5465 PPARA;CNR1 geneontology_Biological_Process

GO:0048538 thymus development http://amigo.geneontology.org/amigo/term/GO:0048538 40 2 0.216855593888615 9.22272727272727 0.0197330169883521 0.200278068253219 596;5594 BCL2;MAPK1 geneontology_Biological_Process

GO:0007566 embryo implantation http://amigo.geneontology.org/amigo/term/GO:0007566 40 2 0.216855593888615 9.22272727272727 0.0197330169883521 0.200278068253219 5467;6256 RXRA;PPARD geneontology_Biological_Process

GO:0003044 regulation of systemic arterial blood pressure mediated by a chemical signal http://amigo.geneontology.org/amigo/term/GO:0003044 40 2 0.216855593888615 9.22272727272727 0.0197330169883521 0.200278068253219 1636;5972 ACE;REN geneontology_Biological_Process

GO:2001239 regulation of extrinsic apoptotic signaling pathway in absence of ligand http://amigo.geneontology.org/amigo/term/GO:2001239 40 2 0.216855593888615 9.22272727272727 0.0197330169883521 0.200278068253219 596;2260 BCL2;FGFR1 geneontology_Biological_Process

GO:0008038 neuron recognition http://amigo.geneontology.org/amigo/term/GO:0008038 40 2 0.216855593888615 9.22272727272727 0.0197330169883521 0.200278068253219 836;1268 CASP3;CNR1 geneontology_Biological_Process

GO:0045843 negative regulation of striated muscle tissue development http://amigo.geneontology.org/amigo/term/GO:0045843 40 2 0.216855593888615 9.22272727272727 0.0197330169883521 0.200278068253219 5465;5950 PPARA;RBP4 geneontology_Biological_Process

GO:0050817 coagulation http://amigo.geneontology.org/amigo/term/GO:0050817 289 5 1.56678166584524 3.19125511167034 0.0197474876095713 0.200278068253219 150;152;239;5328;5594 ALOX12;ADRA2A;ADRA2C;MAPK1;PLAU geneontology_Biological_Process

GO:0051962 positive regulation of nervous system development http://amigo.geneontology.org/amigo/term/GO:0051962 397 6 2.1522917693445 2.78772612777651 0.019891239648698 0.201480632492255 152;596;1268;2260;5468;5914 PPARG;CNR1;ADRA2C;BCL2;FGFR1;RARA geneontology_Biological_Process

GO:0034248 regulation of cellular amide metabolic process http://amigo.geneontology.org/amigo/term/GO:0034248 290 5 1.57220305569246 3.18025078369906 0.0200126501105685 0.202306113472759 836;5594;5914;6256;6774 CASP3;MAPK1;STAT3;RXRA;RARA geneontology_Biological_Process

GO:0043122 regulation of I-kappaB kinase/NF-kappaB signaling http://amigo.geneontology.org/amigo/term/GO:0043122 192 4 1.04090685066535 3.84280303030303 0.0200232994089509 0.202306113472759 834;841;3357;9020 MAP3K14;CASP8;CASP1;HTR2B geneontology_Biological_Process

GO:0006733 oxidoreduction coenzyme metabolic process http://amigo.geneontology.org/amigo/term/GO:0006733 107 3 0.580088713652045 5.17162276975361 0.0201285744454567 0.202602330456031 3939;5465;6774 PPARA;STAT3;LDHA geneontology_Biological_Process

GO:0009156 ribonucleoside monophosphate biosynthetic process http://amigo.geneontology.org/amigo/term/GO:0009156 107 3 0.580088713652045 5.17162276975361 0.0201285744454567 0.202602330456031 3939;5465;6774 PPARA;STAT3;LDHA geneontology_Biological_Process

GO:0070585 protein localization to mitochondrion http://amigo.geneontology.org/amigo/term/GO:0070585 107 3 0.580088713652045 5.17162276975361 0.0201285744454567 0.202602330456031 596;841;3320 BCL2;HSP90AA1;CASP8 geneontology_Biological_Process

GO:0007599 hemostasis http://amigo.geneontology.org/amigo/term/GO:0007599 291 5 1.57762444553967 3.16932208684786 0.0202801570427142 0.203871628964572 150;152;239;5328;5594 ALOX12;ADRA2A;ADRA2C;MAPK1;PLAU geneontology_Biological_Process

GO:0043393 regulation of protein binding http://amigo.geneontology.org/amigo/term/GO:0043393 193 4 1.04632824051257 3.82289213377296 0.020366453867273 0.204482263294753 596;1636;2932;5465 ACE;PPARA;GSK3B;BCL2 geneontology_Biological_Process

GO:0010863 positive regulation of phospholipase C activity http://amigo.geneontology.org/amigo/term/GO:0010863 41 2 0.22227698373583 8.99778270509978 0.0206737209176543 0.205504490413751 2260;3357 FGFR1;HTR2B geneontology_Biological_Process

GO:0045981 positive regulation of nucleotide metabolic process http://amigo.geneontology.org/amigo/term/GO:0045981 41 2 0.22227698373583 8.99778270509978 0.0206737209176543 0.205504490413751 136;6774 ADORA2B;STAT3 geneontology_Biological_Process

GO:1900544 positive regulation of purine nucleotide metabolic process http://amigo.geneontology.org/amigo/term/GO:1900544 41 2 0.22227698373583 8.99778270509978 0.0206737209176543 0.205504490413751 136;6774 ADORA2B;STAT3 geneontology_Biological_Process

GO:0044060 regulation of endocrine process http://amigo.geneontology.org/amigo/term/GO:0044060 41 2 0.22227698373583 8.99778270509978 0.0206737209176543 0.205504490413751 2260;5972 REN;FGFR1 geneontology_Biological_Process

GO:0060986 endocrine hormone secretion http://amigo.geneontology.org/amigo/term/GO:0060986 41 2 0.22227698373583 8.99778270509978 0.0206737209176543 0.205504490413751 2260;5972 REN;FGFR1 geneontology_Biological_Process

GO:0060324 face development http://amigo.geneontology.org/amigo/term/GO:0060324 41 2 0.22227698373583 8.99778270509978 0.0206737209176543 0.205504490413751 5594;5914 MAPK1;RARA geneontology_Biological_Process

GO:0048635 negative regulation of muscle organ development http://amigo.geneontology.org/amigo/term/GO:0048635 41 2 0.22227698373583 8.99778270509978 0.0206737209176543 0.205504490413751 5465;5950 PPARA;RBP4 geneontology_Biological_Process

GO:0042743 hydrogen peroxide metabolic process http://amigo.geneontology.org/amigo/term/GO:0042743 41 2 0.22227698373583 8.99778270509978 0.0206737209176543 0.205504490413751 1544;6774 CYP1A2;STAT3 geneontology_Biological_Process

GO:0051402 neuron apoptotic process http://amigo.geneontology.org/amigo/term/GO:0051402 195 4 1.057171020207 3.78368298368298 0.0210636683218617 0.208861801625201 596;836;5641;23621 CASP3;BACE1;BCL2;LGMN geneontology_Biological_Process

GO:0061448 connective tissue development http://amigo.geneontology.org/amigo/term/GO:0061448 195 4 1.057171020207 3.78368298368298 0.0210636683218617 0.208861801625201 2260;5467;5914;7067 FGFR1;PPARD;RARA;THRA geneontology_Biological_Process

GO:2000378 negative regulation of reactive oxygen species metabolic process http://amigo.geneontology.org/amigo/term/GO:2000378 42 2 0.227698373583046 8.78354978354978 0.0216328846719349 0.212860300408035 596;6774 BCL2;STAT3 geneontology_Biological_Process

GO:0090303 positive regulation of wound healing http://amigo.geneontology.org/amigo/term/GO:0090303 42 2 0.227698373583046 8.78354978354978 0.0216328846719349 0.212860300408035 150;5467 ADRA2A;PPARD geneontology_Biological_Process

GO:0044003 modification by symbiont of host morphology or physiology http://amigo.geneontology.org/amigo/term/GO:0044003 42 2 0.227698373583046 8.78354978354978 0.0216328846719349 0.212860300408035 841;6256 CASP8;RXRA geneontology_Biological_Process

GO:1901862 negative regulation of muscle tissue development http://amigo.geneontology.org/amigo/term/GO:1901862 42 2 0.227698373583046 8.78354978354978 0.0216328846719349 0.212860300408035 5465;5950 PPARA;RBP4 geneontology_Biological_Process

GO:0016052 carbohydrate catabolic process http://amigo.geneontology.org/amigo/term/GO:0016052 110 3 0.596352883193692 5.03057851239669 0.0216401468067386 0.212860300408035 3939;5465;6774 PPARA;STAT3;LDHA geneontology_Biological_Process

GO:0001764 neuron migration http://amigo.geneontology.org/amigo/term/GO:0001764 110 3 0.596352883193692 5.03057851239669 0.0216401468067386 0.212860300408035 2260;5747;6774 PTK2;STAT3;FGFR1 geneontology_Biological_Process

GO:0006913 nucleocytoplasmic transport http://amigo.geneontology.org/amigo/term/GO:0006913 296 5 1.60473139477575 3.11578624078624 0.0216531222859461 0.212860300408035 2932;4193;5594;6774;8644 GSK3B;AKR1C3;MAPK1;STAT3;MDM2 geneontology_Biological_Process

GO:0043491 protein kinase B signaling http://amigo.geneontology.org/amigo/term/GO:0043491 198 4 1.07343518974864 3.72635445362718 0.0221368969239573 0.217018966086271 2260;3320;5747;8644 AKR1C3;PTK2;HSP90AA1;FGFR1 geneontology_Biological_Process

GO:0048592 eye morphogenesis http://amigo.geneontology.org/amigo/term/GO:0048592 111 3 0.601774273040907 4.98525798525798 0.0221575225309277 0.217018966086271 596;5950;6774 BCL2;STAT3;RBP4 geneontology_Biological_Process

GO:0022612 gland morphogenesis http://amigo.geneontology.org/amigo/term/GO:0022612 111 3 0.601774273040907 4.98525798525798 0.0221575225309277 0.217018966086271 596;2260;6256 BCL2;RXRA;FGFR1 geneontology_Biological_Process

GO:0051169 nuclear transport http://amigo.geneontology.org/amigo/term/GO:0051169 299 5 1.6209955643174 3.08452417148069 0.0225055109941016 0.220157822707581 2932;4193;5594;6774;8644 GSK3B;AKR1C3;MAPK1;STAT3;MDM2 geneontology_Biological_Process

GO:0006953 acute-phase response http://amigo.geneontology.org/amigo/term/GO:0006953 43 2 0.233119763430261 8.57928118393235 0.0226102881977697 0.220643324583601 1268;6774 CNR1;STAT3 geneontology_Biological_Process

GO:0030850 prostate gland development http://amigo.geneontology.org/amigo/term/GO:0030850 43 2 0.233119763430261 8.57928118393235 0.0226102881977697 0.220643324583601 5914;6256 RXRA;RARA geneontology_Biological_Process

GO:0050954 sensory perception of mechanical stimulus http://amigo.geneontology.org/amigo/term/GO:0050954 112 3 0.607195662888122 4.94074675324675 0.0226816609530933 0.221070220397872 836;2260;23621 CASP3;BACE1;FGFR1 geneontology_Biological_Process

GO:0002521 leukocyte differentiation http://amigo.geneontology.org/amigo/term/GO:0002521 411 6 2.22819122720552 2.69276708692767 0.0231481215548603 0.22514779499134 596;760;841;5468;5914;6774 PPARG;BCL2;STAT3;CASP8;CA2;RARA geneontology_Biological_Process

GO:0009267 cellular response to starvation http://amigo.geneontology.org/amigo/term/GO:0009267 113 3 0.612617052735338 4.89702333065165 0.0232125632176775 0.22514779499134 596;5594;8644 AKR1C3;BCL2;MAPK1 geneontology_Biological_Process

GO:0009124 nucleoside monophosphate biosynthetic process http://amigo.geneontology.org/amigo/term/GO:0009124 113 3 0.612617052735338 4.89702333065165 0.0232125632176775 0.22514779499134 3939;5465;6774 PPARA;STAT3;LDHA geneontology_Biological_Process

GO:0035270 endocrine system development http://amigo.geneontology.org/amigo/term/GO:0035270 113 3 0.612617052735338 4.89702333065165 0.0232125632176775 0.22514779499134 2932;5594;7067 GSK3B;MAPK1;THRA geneontology_Biological_Process

GO:1903827 regulation of cellular protein localization http://amigo.geneontology.org/amigo/term/GO:1903827 412 6 2.23361261705274 2.68623124448367 0.0233937894493448 0.226630875512902 596;841;2932;3320;4193;5594 GSK3B;BCL2;MAPK1;HSP90AA1;CASP8;MDM2 geneontology_Biological_Process

GO:1900274 regulation of phospholipase C activity http://amigo.geneontology.org/amigo/term/GO:1900274 44 2 0.238541153277477 8.38429752066116 0.0236057132087006 0.227988540714038 2260;3357 FGFR1;HTR2B geneontology_Biological_Process

GO:1903727 positive regulation of phospholipid metabolic process http://amigo.geneontology.org/amigo/term/GO:1903727 44 2 0.238541153277477 8.38429752066116 0.0236057132087006 0.227988540714038 3357;5747 PTK2;HTR2B geneontology_Biological_Process

GO:0009410 response to xenobiotic stimulus http://amigo.geneontology.org/amigo/term/GO:0009410 202 4 1.09512074913751 3.65256525652565 0.0236194076795723 0.227988540714038 1268;1544;4193;5468 PPARG;CNR1;CYP1A2;MDM2 geneontology_Biological_Process

GO:0046631 alpha-beta T cell activation http://amigo.geneontology.org/amigo/term/GO:0046631 115 3 0.623459832429768 4.81185770750988 0.0242946606129458 0.233660906520183 596;5914;6774 BCL2;STAT3;RARA geneontology_Biological_Process

GO:0050729 positive regulation of inflammatory response http://amigo.geneontology.org/amigo/term/GO:0050729 115 3 0.623459832429768 4.81185770750988 0.0242946606129458 0.233660906520183 136;1268;2167 CNR1;ADORA2B;FABP4 geneontology_Biological_Process

GO:0030010 establishment of cell polarity http://amigo.geneontology.org/amigo/term/GO:0030010 115 3 0.623459832429768 4.81185770750988 0.0242946606129458 0.233660906520183 2932;3320;5747 GSK3B;PTK2;HSP90AA1 geneontology_Biological_Process

GO:0060047 heart contraction http://amigo.geneontology.org/amigo/term/GO:0060047 204 4 1.10596352883194 3.61675579322638 0.024382904223635 0.234228090753334 140;1636;4193;7067 ACE;ADORA3;MDM2;THRA geneontology_Biological_Process

GO:0072132 mesenchyme morphogenesis http://amigo.geneontology.org/amigo/term/GO:0072132 45 2 0.243962543124692 8.1979797979798 0.0246189431731131 0.235084466910801 2260;4193 MDM2;FGFR1 geneontology_Biological_Process

GO:1904705 regulation of vascular smooth muscle cell proliferation http://amigo.geneontology.org/amigo/term/GO:1904705 45 2 0.243962543124692 8.1979797979798 0.0246189431731131 0.235084466910801 4193;5468 PPARG;MDM2 geneontology_Biological_Process

GO:1990874 vascular smooth muscle cell proliferation http://amigo.geneontology.org/amigo/term/GO:1990874 45 2 0.243962543124692 8.1979797979798 0.0246189431731131 0.235084466910801 4193;5468 PPARG;MDM2 geneontology_Biological_Process

GO:0045058 T cell selection http://amigo.geneontology.org/amigo/term/GO:0045058 45 2 0.243962543124692 8.1979797979798 0.0246189431731131 0.235084466910801 596;6774 BCL2;STAT3 geneontology_Biological_Process

GO:0002763 positive regulation of myeloid leukocyte differentiation http://amigo.geneontology.org/amigo/term/GO:0002763 45 2 0.243962543124692 8.1979797979798 0.0246189431731131 0.235084466910801 760;841 CASP8;CA2 geneontology_Biological_Process

GO:0043271 negative regulation of ion transport http://amigo.geneontology.org/amigo/term/GO:0043271 116 3 0.628881222276984 4.77037617554859 0.0248458548201327 0.236968450859001 150;596;1268 CNR1;ADRA2A;BCL2 geneontology_Biological_Process

GO:0038093 Fc receptor signaling pathway http://amigo.geneontology.org/amigo/term/GO:0038093 117 3 0.634302612124199 4.72960372960373 0.0254038110402452 0.241713788280669 3320;5594;5747 PTK2;MAPK1;HSP90AA1 geneontology_Biological_Process

GO:0009411 response to UV http://amigo.geneontology.org/amigo/term/GO:0009411 117 3 0.634302612124199 4.72960372960373 0.0254038110402452 0.241713788280669 596;836;4193 CASP3;BCL2;MDM2 geneontology_Biological_Process

GO:0015872 dopamine transport http://amigo.geneontology.org/amigo/term/GO:0015872 46 2 0.249383932971907 8.0197628458498 0.0256497633021866 0.242611590950469 1268;6580 SLC22A1;CNR1 geneontology_Biological_Process

GO:0050853 B cell receptor signaling pathway http://amigo.geneontology.org/amigo/term/GO:0050853 46 2 0.249383932971907 8.0197628458498 0.0256497633021866 0.242611590950469 596;5594 BCL2;MAPK1 geneontology_Biological_Process

GO:0043470 regulation of carbohydrate catabolic process http://amigo.geneontology.org/amigo/term/GO:0043470 46 2 0.249383932971907 8.0197628458498 0.0256497633021866 0.242611590950469 5465;6774 PPARA;STAT3 geneontology_Biological_Process

GO:0051926 negative regulation of calcium ion transport http://amigo.geneontology.org/amigo/term/GO:0051926 46 2 0.249383932971907 8.0197628458498 0.0256497633021866 0.242611590950469 150;596 ADRA2A;BCL2 geneontology_Biological_Process

GO:0043525 positive regulation of neuron apoptotic process http://amigo.geneontology.org/amigo/term/GO:0043525 46 2 0.249383932971907 8.0197628458498 0.0256497633021866 0.242611590950469 836;23621 CASP3;BACE1 geneontology_Biological_Process

GO:0050878 regulation of body fluid levels http://amigo.geneontology.org/amigo/term/GO:0050878 422 6 2.28782651552489 2.62257647565704 0.0259484396391331 0.24415525939434 150;152;231;239;5328;5594 AKR1B1;ALOX12;ADRA2A;ADRA2C;MAPK1;PLAU geneontology_Biological_Process

GO:0034655 nucleobase-containing compound catabolic process http://amigo.geneontology.org/amigo/term/GO:0034655 422 6 2.28782651552489 2.62257647565704 0.0259484396391331 0.24415525939434 836;3939;5141;5465;6774;9261 CASP3;PPARA;PDE4A;MAPKAPK2;STAT3;LDHA geneontology_Biological_Process

GO:0006694 steroid biosynthetic process http://amigo.geneontology.org/amigo/term/GO:0006694 119 3 0.64514539181863 4.65011459129106 0.0265400008515011 0.24415525939434 231;6319;8644 AKR1B1;AKR1C3;SCD geneontology_Biological_Process

GO:1903307 positive regulation of regulated secretory pathway http://amigo.geneontology.org/amigo/term/GO:1903307 47 2 0.254805322819123 7.84912959381044 0.0266979605379238 0.24415525939434 136;1268 CNR1;ADORA2B geneontology_Biological_Process

GO:0010720 positive regulation of cell development http://amigo.geneontology.org/amigo/term/GO:0010720 425 6 2.30409068506654 2.60406417112299 0.0267500229814773 0.24415525939434 152;596;1268;2260;5468;5914 PPARG;CNR1;ADRA2C;BCL2;FGFR1;RARA geneontology_Biological_Process

GO:0008291 acetylcholine metabolic process http://amigo.geneontology.org/amigo/term/GO:0008291 5 1 0.0271069492360769 36.8909090909091 0.0268198541624125 0.24415525939434 43 ACHE geneontology_Biological_Process

GO:1900619 acetate ester metabolic process http://amigo.geneontology.org/amigo/term/GO:1900619 5 1 0.0271069492360769 36.8909090909091 0.0268198541624125 0.24415525939434 43 ACHE geneontology_Biological_Process

GO:0110096 cellular response to aldehyde http://amigo.geneontology.org/amigo/term/GO:0110096 5 1 0.0271069492360769 36.8909090909091 0.0268198541624125 0.24415525939434 231 AKR1B1 geneontology_Biological_Process

GO:0034310 primary alcohol catabolic process http://amigo.geneontology.org/amigo/term/GO:0034310 5 1 0.0271069492360769 36.8909090909091 0.0268198541624125 0.24415525939434 8644 AKR1C3 geneontology_Biological_Process

GO:0060087 relaxation of vascular smooth muscle http://amigo.geneontology.org/amigo/term/GO:0060087 5 1 0.0271069492360769 36.8909090909091 0.0268198541624125 0.24415525939434 136 ADORA2B geneontology_Biological_Process

GO:1901374 acetate ester transport http://amigo.geneontology.org/amigo/term/GO:1901374 5 1 0.0271069492360769 36.8909090909091 0.0268198541624125 0.24415525939434 6580 SLC22A1 geneontology_Biological_Process

GO:0002138 retinoic acid biosynthetic process http://amigo.geneontology.org/amigo/term/GO:0002138 5 1 0.0271069492360769 36.8909090909091 0.0268198541624125 0.24415525939434 8644 AKR1C3 geneontology_Biological_Process

GO:0016102 diterpenoid biosynthetic process http://amigo.geneontology.org/amigo/term/GO:0016102 5 1 0.0271069492360769 36.8909090909091 0.0268198541624125 0.24415525939434 8644 AKR1C3 geneontology_Biological_Process

GO:0042574 retinal metabolic process http://amigo.geneontology.org/amigo/term/GO:0042574 5 1 0.0271069492360769 36.8909090909091 0.0268198541624125 0.24415525939434 8644 AKR1C3 geneontology_Biological_Process

GO:0061302 smooth muscle cell-matrix adhesion http://amigo.geneontology.org/amigo/term/GO:0061302 5 1 0.0271069492360769 36.8909090909091 0.0268198541624125 0.24415525939434 5328 PLAU geneontology_Biological_Process

GO:0019740 nitrogen utilization http://amigo.geneontology.org/amigo/term/GO:0019740 5 1 0.0271069492360769 36.8909090909091 0.0268198541624125 0.24415525939434 596 BCL2 geneontology_Biological_Process

GO:0052150 modulation by symbiont of host apoptotic process http://amigo.geneontology.org/amigo/term/GO:0052150 5 1 0.0271069492360769 36.8909090909091 0.0268198541624125 0.24415525939434 841 CASP8 geneontology_Biological_Process

GO:0009820 alkaloid metabolic process http://amigo.geneontology.org/amigo/term/GO:0009820 5 1 0.0271069492360769 36.8909090909091 0.0268198541624125 0.24415525939434 1544 CYP1A2 geneontology_Biological_Process

GO:0014042 positive regulation of neuron maturation http://amigo.geneontology.org/amigo/term/GO:0014042 5 1 0.0271069492360769 36.8909090909091 0.0268198541624125 0.24415525939434 596 BCL2 geneontology_Biological_Process

GO:0035442 dipeptide transmembrane transport http://amigo.geneontology.org/amigo/term/GO:0035442 5 1 0.0271069492360769 36.8909090909091 0.0268198541624125 0.24415525939434 760 CA2 geneontology_Biological_Process

GO:0042938 dipeptide transport http://amigo.geneontology.org/amigo/term/GO:0042938 5 1 0.0271069492360769 36.8909090909091 0.0268198541624125 0.24415525939434 760 CA2 geneontology_Biological_Process

GO:0097264 self proteolysis http://amigo.geneontology.org/amigo/term/GO:0097264 5 1 0.0271069492360769 36.8909090909091 0.0268198541624125 0.24415525939434 5641 LGMN geneontology_Biological_Process

GO:0043416 regulation of skeletal muscle tissue regeneration http://amigo.geneontology.org/amigo/term/GO:0043416 5 1 0.0271069492360769 36.8909090909091 0.0268198541624125 0.24415525939434 5467 PPARD geneontology_Biological_Process

GO:1900084 regulation of peptidyl-tyrosine autophosphorylation http://amigo.geneontology.org/amigo/term/GO:1900084 5 1 0.0271069492360769 36.8909090909091 0.0268198541624125 0.24415525939434 1636 ACE geneontology_Biological_Process

GO:0060509 type I pneumocyte differentiation http://amigo.geneontology.org/amigo/term/GO:0060509 5 1 0.0271069492360769 36.8909090909091 0.0268198541624125 0.24415525939434 7067 THRA geneontology_Biological_Process

GO:0010727 negative regulation of hydrogen peroxide metabolic process http://amigo.geneontology.org/amigo/term/GO:0010727 5 1 0.0271069492360769 36.8909090909091 0.0268198541624125 0.24415525939434 6774 STAT3 geneontology_Biological_Process

GO:0016098 monoterpenoid metabolic process http://amigo.geneontology.org/amigo/term/GO:0016098 5 1 0.0271069492360769 36.8909090909091 0.0268198541624125 0.24415525939434 1544 CYP1A2 geneontology_Biological_Process

GO:0061370 testosterone biosynthetic process http://amigo.geneontology.org/amigo/term/GO:0061370 5 1 0.0271069492360769 36.8909090909091 0.0268198541624125 0.24415525939434 8644 AKR1C3 geneontology_Biological_Process

GO:0016093 polyprenol metabolic process http://amigo.geneontology.org/amigo/term/GO:0016093 5 1 0.0271069492360769 36.8909090909091 0.0268198541624125 0.24415525939434 8644 AKR1C3 geneontology_Biological_Process

GO:0045719 negative regulation of glycogen biosynthetic process http://amigo.geneontology.org/amigo/term/GO:0045719 5 1 0.0271069492360769 36.8909090909091 0.0268198541624125 0.24415525939434 2932 GSK3B geneontology_Biological_Process

GO:0060633 negative regulation of transcription initiation from RNA polymerase II promoter http://amigo.geneontology.org/amigo/term/GO:0060633 5 1 0.0271069492360769 36.8909090909091 0.0268198541624125 0.24415525939434 7067 THRA geneontology_Biological_Process

GO:2000143 negative regulation of DNA-templated transcription, initiation http://amigo.geneontology.org/amigo/term/GO:2000143 5 1 0.0271069492360769 36.8909090909091 0.0268198541624125 0.24415525939434 7067 THRA geneontology_Biological_Process

GO:0045040 protein import into mitochondrial outer membrane http://amigo.geneontology.org/amigo/term/GO:0045040 5 1 0.0271069492360769 36.8909090909091 0.0268198541624125 0.24415525939434 3320 HSP90AA1 geneontology_Biological_Process

GO:0002253 activation of immune response http://amigo.geneontology.org/amigo/term/GO:0002253 426 6 2.30951207491375 2.59795134443022 0.0270208716995286 0.245705699249577 596;841;3320;5594;5747;9261 PTK2;MAPKAPK2;BCL2;MAPK1;HSP90AA1;CASP8 geneontology_Biological_Process

GO:1902107 positive regulation of leukocyte differentiation http://amigo.geneontology.org/amigo/term/GO:1902107 120 3 0.650566781665845 4.61136363636364 0.0271182286022097 0.246311084307471 760;841;5914 CASP8;CA2;RARA geneontology_Biological_Process

GO:0048568 embryonic organ development http://amigo.geneontology.org/amigo/term/GO:0048568 315 5 1.70773780187284 2.92784992784993 0.0274223606546362 0.248791077050339 841;2260;5594;5914;5950 MAPK1;CASP8;FGFR1;RBP4;RARA geneontology_Biological_Process

GO:0030195 negative regulation of blood coagulation http://amigo.geneontology.org/amigo/term/GO:0030195 48 2 0.260226712666338 7.68560606060606 0.0277633235412522 0.250464616659639 239;5328 ALOX12;PLAU geneontology_Biological_Process

GO:0071398 cellular response to fatty acid http://amigo.geneontology.org/amigo/term/GO:0071398 48 2 0.260226712666338 7.68560606060606 0.0277633235412522 0.250464616659639 5468;8644 PPARG;AKR1C3 geneontology_Biological_Process

GO:2001169 regulation of ATP biosynthetic process http://amigo.geneontology.org/amigo/term/GO:2001169 48 2 0.260226712666338 7.68560606060606 0.0277633235412522 0.250464616659639 5465;6774 PPARA;STAT3 geneontology_Biological_Process

GO:0061098 positive regulation of protein tyrosine kinase activity http://amigo.geneontology.org/amigo/term/GO:0061098 48 2 0.260226712666338 7.68560606060606 0.0277633235412522 0.250464616659639 150;1636 ACE;ADRA2A geneontology_Biological_Process

GO:0060964 regulation of gene silencing by miRNA http://amigo.geneontology.org/amigo/term/GO:0060964 48 2 0.260226712666338 7.68560606060606 0.0277633235412522 0.250464616659639 5468;6774 PPARG;STAT3 geneontology_Biological_Process

GO:0006732 coenzyme metabolic process http://amigo.geneontology.org/amigo/term/GO:0006732 213 4 1.15475603745688 3.46393512590696 0.0280040108884489 0.252067598570718 3939;5465;6319;6774 PPARA;STAT3;LDHA;SCD geneontology_Biological_Process

GO:0003015 heart process http://amigo.geneontology.org/amigo/term/GO:0003015 213 4 1.15475603745688 3.46393512590696 0.0280040108884489 0.252067598570718 140;1636;4193;7067 ACE;ADORA3;MDM2;THRA geneontology_Biological_Process

GO:0007162 negative regulation of cell adhesion http://amigo.geneontology.org/amigo/term/GO:0007162 214 4 1.16017742730409 3.44774851316907 0.028425216991468 0.25557144535475 239;836;5465;5747 CASP3;PPARA;ALOX12;PTK2 geneontology_Biological_Process

GO:0050865 regulation of cell activation http://amigo.geneontology.org/amigo/term/GO:0050865 432 6 2.34204041399704 2.56186868686869 0.0286846602067993 0.257327572716803 136;239;596;836;1268;5914 CASP3;CNR1;ADORA2B;ALOX12;BCL2;RARA geneontology_Biological_Process

GO:0042220 response to cocaine http://amigo.geneontology.org/amigo/term/GO:0042220 49 2 0.265648102513553 7.52875695732839 0.0288456426802015 0.257327572716803 1268;4193 CNR1;MDM2 geneontology_Biological_Process

GO:0050819 negative regulation of coagulation http://amigo.geneontology.org/amigo/term/GO:0050819 49 2 0.265648102513553 7.52875695732839 0.0288456426802015 0.257327572716803 239;5328 ALOX12;PLAU geneontology_Biological_Process

GO:1900047 negative regulation of hemostasis http://amigo.geneontology.org/amigo/term/GO:1900047 49 2 0.265648102513553 7.52875695732839 0.0288456426802015 0.257327572716803 239;5328 ALOX12;PLAU geneontology_Biological_Process

GO:0060688 regulation of morphogenesis of a branching structure http://amigo.geneontology.org/amigo/term/GO:0060688 49 2 0.265648102513553 7.52875695732839 0.0288456426802015 0.257327572716803 2260;6256 RXRA;FGFR1 geneontology_Biological_Process

GO:1903036 positive regulation of response to wounding http://amigo.geneontology.org/amigo/term/GO:1903036 49 2 0.265648102513553 7.52875695732839 0.0288456426802015 0.257327572716803 150;5467 ADRA2A;PPARD geneontology_Biological_Process

GO:0010803 regulation of tumor necrosis factor-mediated signaling pathway http://amigo.geneontology.org/amigo/term/GO:0010803 49 2 0.265648102513553 7.52875695732839 0.0288456426802015 0.257327572716803 834;841 CASP8;CASP1 geneontology_Biological_Process

GO:0010212 response to ionizing radiation http://amigo.geneontology.org/amigo/term/GO:0010212 123 3 0.666830951207491 4.49889135254989 0.0288933958191003 0.257466540472651 596;836;4193 CASP3;BCL2;MDM2 geneontology_Biological_Process

GO:1903706 regulation of hemopoiesis http://amigo.geneontology.org/amigo/term/GO:1903706 320 5 1.73484475110892 2.88210227272727 0.0290896066949095 0.258638925302962 760;841;1636;5914;6774 ACE;STAT3;CASP8;CA2;RARA geneontology_Biological_Process

GO:0009150 purine ribonucleotide metabolic process http://amigo.geneontology.org/amigo/term/GO:0009150 320 5 1.73484475110892 2.88210227272727 0.0290896066949095 0.258638925302962 3939;5141;5465;6319;6774 PPARA;PDE4A;STAT3;LDHA;SCD geneontology_Biological_Process

GO:0022407 regulation of cell-cell adhesion http://amigo.geneontology.org/amigo/term/GO:0022407 321 5 1.74026614095614 2.87312376097423 0.0294306552605743 0.2611147875937 239;836;5465;5747;5914 CASP3;PPARA;ALOX12;PTK2;RARA geneontology_Biological_Process

GO:0051592 response to calcium ion http://amigo.geneontology.org/amigo/term/GO:0051592 124 3 0.672252341054707 4.46260997067449 0.0294985963490009 0.2611147875937 5641;8644;9536 AKR1C3;PTGES;LGMN geneontology_Biological_Process

GO:0072073 kidney epithelium development http://amigo.geneontology.org/amigo/term/GO:0072073 124 3 0.672252341054707 4.46260997067449 0.0294985963490009 0.2611147875937 596;2260;5914 BCL2;FGFR1;RARA geneontology_Biological_Process

GO:0090316 positive regulation of intracellular protein transport http://amigo.geneontology.org/amigo/term/GO:0090316 124 3 0.672252341054707 4.46260997067449 0.0294985963490009 0.2611147875937 2932;4193;5594 GSK3B;MAPK1;MDM2 geneontology_Biological_Process

GO:0051196 regulation of coenzyme metabolic process http://amigo.geneontology.org/amigo/term/GO:0051196 50 2 0.271069492360769 7.37818181818182 0.0299447100181551 0.263896001723874 5465;6774 PPARA;STAT3 geneontology_Biological_Process

GO:0097755 positive regulation of blood vessel diameter http://amigo.geneontology.org/amigo/term/GO:0097755 50 2 0.271069492360769 7.37818181818182 0.0299447100181551 0.263896001723874 136;5467 ADORA2B;PPARD geneontology_Biological_Process

GO:0060147 regulation of posttranscriptional gene silencing http://amigo.geneontology.org/amigo/term/GO:0060147 50 2 0.271069492360769 7.37818181818182 0.0299447100181551 0.263896001723874 5468;6774 PPARG;STAT3 geneontology_Biological_Process

GO:0060966 regulation of gene silencing by RNA http://amigo.geneontology.org/amigo/term/GO:0060966 50 2 0.271069492360769 7.37818181818182 0.0299447100181551 0.263896001723874 5468;6774 PPARG;STAT3 geneontology_Biological_Process

GO:0071456 cellular response to hypoxia http://amigo.geneontology.org/amigo/term/GO:0071456 125 3 0.677673730901922 4.42690909090909 0.0301105262684975 0.265065380858655 596;4193;5467 BCL2;MDM2;PPARD geneontology_Biological_Process

GO:0021700 developmental maturation http://amigo.geneontology.org/amigo/term/GO:0021700 219 4 1.18728437654017 3.36903279369033 0.0305882800614834 0.267553910901741 596;2260;5468;5972 REN;PPARG;BCL2;FGFR1 geneontology_Biological_Process

GO:0071300 cellular response to retinoic acid http://amigo.geneontology.org/amigo/term/GO:0071300 51 2 0.276490882207984 7.23351158645276 0.0310603193021741 0.267553910901741 5468;5914 PPARG;RARA geneontology_Biological_Process

GO:0002260 lymphocyte homeostasis http://amigo.geneontology.org/amigo/term/GO:0002260 51 2 0.276490882207984 7.23351158645276 0.0310603193021741 0.267553910901741 596;836 CASP3;BCL2 geneontology_Biological_Process

GO:0010332 response to gamma radiation http://amigo.geneontology.org/amigo/term/GO:0010332 51 2 0.276490882207984 7.23351158645276 0.0310603193021741 0.267553910901741 596;4193 BCL2;MDM2 geneontology_Biological_Process

GO:0010611 regulation of cardiac muscle hypertrophy http://amigo.geneontology.org/amigo/term/GO:0010611 51 2 0.276490882207984 7.23351158645276 0.0310603193021741 0.267553910901741 5465;5747 PPARA;PTK2 geneontology_Biological_Process

GO:0042093 T-helper cell differentiation http://amigo.geneontology.org/amigo/term/GO:0042093 51 2 0.276490882207984 7.23351158645276 0.0310603193021741 0.267553910901741 5914;6774 STAT3;RARA geneontology_Biological_Process

GO:0046700 heterocycle catabolic process http://amigo.geneontology.org/amigo/term/GO:0046700 443 6 2.40167570231641 2.49825569464396 0.0319097343044992 0.267553910901741 836;3939;5141;5465;6774;9261 CASP3;PPARA;PDE4A;MAPKAPK2;STAT3;LDHA geneontology_Biological_Process

GO:0006109 regulation of carbohydrate metabolic process http://amigo.geneontology.org/amigo/term/GO:0006109 128 3 0.693937900443568 4.32315340909091 0.0319866233602863 0.267553910901741 2932;5465;6774 PPARA;GSK3B;STAT3 geneontology_Biological_Process

GO:0032222 regulation of synaptic transmission, cholinergic http://amigo.geneontology.org/amigo/term/GO:0032222 6 1 0.0325283390832923 30.7424242424242 0.0320984446970346 0.267553910901741 43 ACHE geneontology_Biological_Process

GO:0042573 retinoic acid metabolic process http://amigo.geneontology.org/amigo/term/GO:0042573 6 1 0.0325283390832923 30.7424242424242 0.0320984446970346 0.267553910901741 8644 AKR1C3 geneontology_Biological_Process

GO:0035931 mineralocorticoid secretion http://amigo.geneontology.org/amigo/term/GO:0035931 6 1 0.0325283390832923 30.7424242424242 0.0320984446970346 0.267553910901741 5972 REN geneontology_Biological_Process

GO:0035932 aldosterone secretion http://amigo.geneontology.org/amigo/term/GO:0035932 6 1 0.0325283390832923 30.7424242424242 0.0320984446970346 0.267553910901741 5972 REN geneontology_Biological_Process

GO:2000855 regulation of mineralocorticoid secretion http://amigo.geneontology.org/amigo/term/GO:2000855 6 1 0.0325283390832923 30.7424242424242 0.0320984446970346 0.267553910901741 5972 REN geneontology_Biological_Process

GO:2000858 regulation of aldosterone secretion http://amigo.geneontology.org/amigo/term/GO:2000858 6 1 0.0325283390832923 30.7424242424242 0.0320984446970346 0.267553910901741 5972 REN geneontology_Biological_Process

GO:0035360 positive regulation of peroxisome proliferator activated receptor signaling pathway http://amigo.geneontology.org/amigo/term/GO:0035360 6 1 0.0325283390832923 30.7424242424242 0.0320984446970346 0.267553910901741 2171 FABP5 geneontology_Biological_Process

GO:0045759 negative regulation of action potential http://amigo.geneontology.org/amigo/term/GO:0045759 6 1 0.0325283390832923 30.7424242424242 0.0320984446970346 0.267553910901741 1268 CNR1 geneontology_Biological_Process

GO:0031392 regulation of prostaglandin biosynthetic process http://amigo.geneontology.org/amigo/term/GO:0031392 6 1 0.0325283390832923 30.7424242424242 0.0320984446970346 0.267553910901741 2171 FABP5 geneontology_Biological_Process

GO:0019755 one-carbon compound transport http://amigo.geneontology.org/amigo/term/GO:0019755 6 1 0.0325283390832923 30.7424242424242 0.0320984446970346 0.267553910901741 760 CA2 geneontology_Biological_Process

GO:0060331 negative regulation of response to interferon-gamma http://amigo.geneontology.org/amigo/term/GO:0060331 6 1 0.0325283390832923 30.7424242424242 0.0320984446970346 0.267553910901741 5468 PPARG geneontology_Biological_Process

GO:0060336 negative regulation of interferon-gamma-mediated signaling pathway http://amigo.geneontology.org/amigo/term/GO:0060336 6 1 0.0325283390832923 30.7424242424242 0.0320984446970346 0.267553910901741 5468 PPARG geneontology_Biological_Process

GO:1905323 telomerase holoenzyme complex assembly http://amigo.geneontology.org/amigo/term/GO:1905323 6 1 0.0325283390832923 30.7424242424242 0.0320984446970346 0.267553910901741 3320 HSP90AA1 geneontology_Biological_Process

GO:2000169 regulation of peptidyl-cysteine S-nitrosylation http://amigo.geneontology.org/amigo/term/GO:2000169 6 1 0.0325283390832923 30.7424242424242 0.0320984446970346 0.267553910901741 1636 ACE geneontology_Biological_Process

GO:0010571 positive regulation of nuclear cell cycle DNA replication http://amigo.geneontology.org/amigo/term/GO:0010571 6 1 0.0325283390832923 30.7424242424242 0.0320984446970346 0.267553910901741 2260 FGFR1 geneontology_Biological_Process

GO:0044531 modulation of programmed cell death in other organism http://amigo.geneontology.org/amigo/term/GO:0044531 6 1 0.0325283390832923 30.7424242424242 0.0320984446970346 0.267553910901741 841 CASP8 geneontology_Biological_Process

GO:0044532 modulation of apoptotic process in other organism http://amigo.geneontology.org/amigo/term/GO:0044532 6 1 0.0325283390832923 30.7424242424242 0.0320984446970346 0.267553910901741 841 CASP8 geneontology_Biological_Process

GO:0052040 modulation by symbiont of host programmed cell death http://amigo.geneontology.org/amigo/term/GO:0052040 6 1 0.0325283390832923 30.7424242424242 0.0320984446970346 0.267553910901741 841 CASP8 geneontology_Biological_Process

GO:0052248 modulation of programmed cell death in other organism involved in symbiotic interaction http://amigo.geneontology.org/amigo/term/GO:0052248 6 1 0.0325283390832923 30.7424242424242 0.0320984446970346 0.267553910901741 841 CASP8 geneontology_Biological_Process

GO:0052433 modulation by organism of apoptotic process in other organism involved in symbiotic interaction http://amigo.geneontology.org/amigo/term/GO:0052433 6 1 0.0325283390832923 30.7424242424242 0.0320984446970346 0.267553910901741 841 CASP8 geneontology_Biological_Process

GO:0045852 pH elevation http://amigo.geneontology.org/amigo/term/GO:0045852 6 1 0.0325283390832923 30.7424242424242 0.0320984446970346 0.267553910901741 596 BCL2 geneontology_Biological_Process

GO:0051454 intracellular pH elevation http://amigo.geneontology.org/amigo/term/GO:0051454 6 1 0.0325283390832923 30.7424242424242 0.0320984446970346 0.267553910901741 596 BCL2 geneontology_Biological_Process

GO:0048743 positive regulation of skeletal muscle fiber development http://amigo.geneontology.org/amigo/term/GO:0048743 6 1 0.0325283390832923 30.7424242424242 0.0320984446970346 0.267553910901741 596 BCL2 geneontology_Biological_Process

GO:0060440 trachea formation http://amigo.geneontology.org/amigo/term/GO:0060440 6 1 0.0325283390832923 30.7424242424242 0.0320984446970346 0.267553910901741 5594 MAPK1 geneontology_Biological_Process

GO:0042661 regulation of mesodermal cell fate specification http://amigo.geneontology.org/amigo/term/GO:0042661 6 1 0.0325283390832923 30.7424242424242 0.0320984446970346 0.267553910901741 2260 FGFR1 geneontology_Biological_Process

GO:1905770 regulation of mesodermal cell differentiation http://amigo.geneontology.org/amigo/term/GO:1905770 6 1 0.0325283390832923 30.7424242424242 0.0320984446970346 0.267553910901741 2260 FGFR1 geneontology_Biological_Process

GO:1905902 regulation of mesoderm formation http://amigo.geneontology.org/amigo/term/GO:1905902 6 1 0.0325283390832923 30.7424242424242 0.0320984446970346 0.267553910901741 2260 FGFR1 geneontology_Biological_Process

GO:0035672 oligopeptide transmembrane transport http://amigo.geneontology.org/amigo/term/GO:0035672 6 1 0.0325283390832923 30.7424242424242 0.0320984446970346 0.267553910901741 760 CA2 geneontology_Biological_Process

GO:1902949 positive regulation of tau-protein kinase activity http://amigo.geneontology.org/amigo/term/GO:1902949 6 1 0.0325283390832923 30.7424242424242 0.0320984446970346 0.267553910901741 3320 HSP90AA1 geneontology_Biological_Process

GO:2000288 positive regulation of myoblast proliferation http://amigo.geneontology.org/amigo/term/GO:2000288 6 1 0.0325283390832923 30.7424242424242 0.0320984446970346 0.267553910901741 5467 PPARD geneontology_Biological_Process

GO:0036015 response to interleukin-3 http://amigo.geneontology.org/amigo/term/GO:0036015 6 1 0.0325283390832923 30.7424242424242 0.0320984446970346 0.267553910901741 2932 GSK3B geneontology_Biological_Process

GO:0036016 cellular response to interleukin-3 http://amigo.geneontology.org/amigo/term/GO:0036016 6 1 0.0325283390832923 30.7424242424242 0.0320984446970346 0.267553910901741 2932 GSK3B geneontology_Biological_Process

GO:0060484 lung-associated mesenchyme development http://amigo.geneontology.org/amigo/term/GO:0060484 6 1 0.0325283390832923 30.7424242424242 0.0320984446970346 0.267553910901741 2260 FGFR1 geneontology_Biological_Process

GO:0070989 oxidative demethylation http://amigo.geneontology.org/amigo/term/GO:0070989 6 1 0.0325283390832923 30.7424242424242 0.0320984446970346 0.267553910901741 1544 CYP1A2 geneontology_Biological_Process

GO:1902033 regulation of hematopoietic stem cell proliferation http://amigo.geneontology.org/amigo/term/GO:1902033 6 1 0.0325283390832923 30.7424242424242 0.0320984446970346 0.267553910901741 1636 ACE geneontology_Biological_Process

GO:0021869 forebrain ventricular zone progenitor cell division http://amigo.geneontology.org/amigo/term/GO:0021869 6 1 0.0325283390832923 30.7424242424242 0.0320984446970346 0.267553910901741 2260 FGFR1 geneontology_Biological_Process

GO:0051964 negative regulation of synapse assembly http://amigo.geneontology.org/amigo/term/GO:0051964 6 1 0.0325283390832923 30.7424242424242 0.0320984446970346 0.267553910901741 5747 PTK2 geneontology_Biological_Process

GO:0010513 positive regulation of phosphatidylinositol biosynthetic process http://amigo.geneontology.org/amigo/term/GO:0010513 6 1 0.0325283390832923 30.7424242424242 0.0320984446970346 0.267553910901741 3357 HTR2B geneontology_Biological_Process

GO:0015959 diadenosine polyphosphate metabolic process http://amigo.geneontology.org/amigo/term/GO:0015959 6 1 0.0325283390832923 30.7424242424242 0.0320984446970346 0.267553910901741 5594 MAPK1 geneontology_Biological_Process

GO:0070874 negative regulation of glycogen metabolic process http://amigo.geneontology.org/amigo/term/GO:0070874 6 1 0.0325283390832923 30.7424242424242 0.0320984446970346 0.267553910901741 2932 GSK3B geneontology_Biological_Process

GO:2000465 regulation of glycogen (starch) synthase activity http://amigo.geneontology.org/amigo/term/GO:2000465 6 1 0.0325283390832923 30.7424242424242 0.0320984446970346 0.267553910901741 2932 GSK3B geneontology_Biological_Process

GO:0007008 outer mitochondrial membrane organization http://amigo.geneontology.org/amigo/term/GO:0007008 6 1 0.0325283390832923 30.7424242424242 0.0320984446970346 0.267553910901741 3320 HSP90AA1 geneontology_Biological_Process

GO:1904683 regulation of metalloendopeptidase activity http://amigo.geneontology.org/amigo/term/GO:1904683 6 1 0.0325283390832923 30.7424242424242 0.0320984446970346 0.267553910901741 6774 STAT3 geneontology_Biological_Process

GO:1903749 positive regulation of establishment of protein localization to mitochondrion http://amigo.geneontology.org/amigo/term/GO:1903749 52 2 0.2819122720552 7.09440559440559 0.0321922659513967 0.267961309248314 596;841 BCL2;CASP8 geneontology_Biological_Process

GO:0044270 cellular nitrogen compound catabolic process http://amigo.geneontology.org/amigo/term/GO:0044270 444 6 2.40709709216363 2.49262899262899 0.0322142938636438 0.267961309248314 836;3939;5141;5465;6774;9261 CASP3;PPARA;PDE4A;MAPKAPK2;STAT3;LDHA geneontology_Biological_Process

GO:0009259 ribonucleotide metabolic process http://amigo.geneontology.org/amigo/term/GO:0009259 329 5 1.78363725973386 2.80326056921802 0.032251122697676 0.267989079778612 3939;5141;5465;6319;6774 PPARA;PDE4A;STAT3;LDHA;SCD geneontology_Biological_Process

GO:0007249 I-kappaB kinase/NF-kappaB signaling http://amigo.geneontology.org/amigo/term/GO:0007249 223 4 1.20896993592903 3.30860171218916 0.0323874746680947 0.268842917317524 834;841;3357;9020 MAP3K14;CASP8;CASP1;HTR2B geneontology_Biological_Process

GO:0001959 regulation of cytokine-mediated signaling pathway http://amigo.geneontology.org/amigo/term/GO:0001959 129 3 0.699359290290784 4.28964059196617 0.0326253991626457 0.270537247771493 834;841;5468 PPARG;CASP8;CASP1 geneontology_Biological_Process

GO:0051384 response to glucocorticoid http://amigo.geneontology.org/amigo/term/GO:0051384 130 3 0.704780680137999 4.25664335664336 0.0332708658727738 0.274192658847023 249;596;836 CASP3;ALPL;BCL2 geneontology_Biological_Process

GO:0015893 drug transport http://amigo.geneontology.org/amigo/term/GO:0015893 130 3 0.704780680137999 4.25664335664336 0.0332708658727738 0.274192658847023 760;1268;6580 SLC22A1;CNR1;CA2 geneontology_Biological_Process

GO:0032024 positive regulation of insulin secretion http://amigo.geneontology.org/amigo/term/GO:0032024 53 2 0.287333661902415 6.96054888507719 0.0333403470455078 0.274192658847023 5467;5950 PPARD;RBP4 geneontology_Biological_Process

GO:0050891 multicellular organismal water homeostasis http://amigo.geneontology.org/amigo/term/GO:0050891 53 2 0.287333661902415 6.96054888507719 0.0333403470455078 0.274192658847023 231;239 AKR1B1;ALOX12 geneontology_Biological_Process

GO:0014743 regulation of muscle hypertrophy http://amigo.geneontology.org/amigo/term/GO:0014743 53 2 0.287333661902415 6.96054888507719 0.0333403470455078 0.274192658847023 5465;5747 PPARA;PTK2 geneontology_Biological_Process

GO:0002287 alpha-beta T cell activation involved in immune response http://amigo.geneontology.org/amigo/term/GO:0002287 53 2 0.287333661902415 6.96054888507719 0.0333403470455078 0.274192658847023 5914;6774 STAT3;RARA geneontology_Biological_Process

GO:0002293 alpha-beta T cell differentiation involved in immune response http://amigo.geneontology.org/amigo/term/GO:0002293 53 2 0.287333661902415 6.96054888507719 0.0333403470455078 0.274192658847023 5914;6774 STAT3;RARA geneontology_Biological_Process

GO:0002294 CD4-positive, alpha-beta T cell differentiation involved in immune response http://amigo.geneontology.org/amigo/term/GO:0002294 53 2 0.287333661902415 6.96054888507719 0.0333403470455078 0.274192658847023 5914;6774 STAT3;RARA geneontology_Biological_Process

GO:0019439 aromatic compound catabolic process http://amigo.geneontology.org/amigo/term/GO:0019439 448 6 2.42878265155249 2.47037337662338 0.0334517007925581 0.274825985361448 836;3939;5141;5465;6774;9261 CASP3;PPARA;PDE4A;MAPKAPK2;STAT3;LDHA geneontology_Biological_Process

GO:0050768 negative regulation of neurogenesis http://amigo.geneontology.org/amigo/term/GO:0050768 226 4 1.22523410547068 3.26468222043443 0.0337771590375823 0.277215206788444 2932;4193;5747;6774 GSK3B;PTK2;STAT3;MDM2 geneontology_Biological_Process

GO:0045931 positive regulation of mitotic cell cycle http://amigo.geneontology.org/amigo/term/GO:0045931 131 3 0.710202069985214 4.22414989590562 0.0339230142348328 0.27812700810157 2260;4193;5641 MDM2;FGFR1;LGMN geneontology_Biological_Process

GO:0030888 regulation of B cell proliferation http://amigo.geneontology.org/amigo/term/GO:0030888 54 2 0.29275505174963 6.83164983164983 0.0345043613132836 0.282314825387419 596;836 CASP3;BCL2 geneontology_Biological_Process

GO:0045576 mast cell activation http://amigo.geneontology.org/amigo/term/GO:0045576 54 2 0.29275505174963 6.83164983164983 0.0345043613132836 0.282314825387419 136;1268 CNR1;ADORA2B geneontology_Biological_Process

GO:0032943 mononuclear cell proliferation http://amigo.geneontology.org/amigo/term/GO:0032943 228 4 1.23607688516511 3.23604465709729 0.0347228573969285 0.283522760092063 596;836;1636;23385 CASP3;ACE;NCSTN;BCL2 geneontology_Biological_Process

GO:0043010 camera-type eye development http://amigo.geneontology.org/amigo/term/GO:0043010 228 4 1.23607688516511 3.23604465709729 0.0347228573969285 0.283522760092063 43;5914;5950;6256 ACHE;RXRA;RBP4;RARA geneontology_Biological_Process

GO:2001233 regulation of apoptotic signaling pathway http://amigo.geneontology.org/amigo/term/GO:2001233 336 5 1.82158698866437 2.74485930735931 0.0348546743815583 0.284308974924801 596;841;2260;2932;4193 GSK3B;BCL2;CASP8;MDM2;FGFR1 geneontology_Biological_Process

GO:0042063 gliogenesis http://amigo.geneontology.org/amigo/term/GO:0042063 229 4 1.24149827501232 3.22191345772132 0.0352014892456606 0.285722821050479 5468;5594;6774;83933 PPARG;MAPK1;STAT3;HDAC10 geneontology_Biological_Process

GO:0006937 regulation of muscle contraction http://amigo.geneontology.org/amigo/term/GO:0006937 133 3 0.721044849679645 4.16062884483937 0.0352473163885985 0.285722821050479 136;150;152 ADORA2B;ADRA2A;ADRA2C geneontology_Biological_Process

GO:0030003 cellular cation homeostasis http://amigo.geneontology.org/amigo/term/GO:0030003 454 6 2.46131099063578 2.43772527032439 0.0353657408024319 0.285722821050479 596;760;1268;3357;5594;5739 CNR1;BCL2;MAPK1;CA2;HTR2B;PTGIR geneontology_Biological_Process

GO:0007005 mitochondrion organization http://amigo.geneontology.org/amigo/term/GO:0007005 338 5 1.8324297683588 2.72861753630984 0.035622028867083 0.285722821050479 596;841;2932;3320;6774 GSK3B;BCL2;STAT3;HSP90AA1;CASP8 geneontology_Biological_Process

GO:0010518 positive regulation of phospholipase activity http://amigo.geneontology.org/amigo/term/GO:0010518 55 2 0.298176441596846 6.70743801652893 0.0356841091212059 0.285722821050479 2260;3357 FGFR1;HTR2B geneontology_Biological_Process

GO:0046888 negative regulation of hormone secretion http://amigo.geneontology.org/amigo/term/GO:0046888 55 2 0.298176441596846 6.70743801652893 0.0356841091212059 0.285722821050479 150;152 ADRA2A;ADRA2C geneontology_Biological_Process

GO:0048645 animal organ formation http://amigo.geneontology.org/amigo/term/GO:0048645 55 2 0.298176441596846 6.70743801652893 0.0356841091212059 0.285722821050479 2260;5594 MAPK1;FGFR1 geneontology_Biological_Process

GO:0014074 response to purine-containing compound http://amigo.geneontology.org/amigo/term/GO:0014074 134 3 0.726466239526861 4.12957937584803 0.0359194491881707 0.285722821050479 3939;5468;5972 REN;PPARG;LDHA geneontology_Biological_Process

GO:0034614 cellular response to reactive oxygen species http://amigo.geneontology.org/amigo/term/GO:0034614 134 3 0.726466239526861 4.12957937584803 0.0359194491881707 0.285722821050479 4193;5594;8644 AKR1C3;MAPK1;MDM2 geneontology_Biological_Process

GO:0048771 tissue remodeling http://amigo.geneontology.org/amigo/term/GO:0048771 134 3 0.726466239526861 4.12957937584803 0.0359194491881707 0.285722821050479 760;1636;4193 ACE;CA2;MDM2 geneontology_Biological_Process

GO:0036294 cellular response to decreased oxygen levels http://amigo.geneontology.org/amigo/term/GO:0036294 134 3 0.726466239526861 4.12957937584803 0.0359194491881707 0.285722821050479 596;4193;5467 BCL2;MDM2;PPARD geneontology_Biological_Process

GO:0006606 protein import into nucleus http://amigo.geneontology.org/amigo/term/GO:0006606 134 3 0.726466239526861 4.12957937584803 0.0359194491881707 0.285722821050479 5594;6774;8644 AKR1C3;MAPK1;STAT3 geneontology_Biological_Process

GO:0034599 cellular response to oxidative stress http://amigo.geneontology.org/amigo/term/GO:0034599 231 4 1.25234105470675 3.19401810310901 0.0361703331615004 0.285722821050479 596;4193;5594;8644 AKR1C3;BCL2;MAPK1;MDM2 geneontology_Biological_Process

GO:0051348 negative regulation of transferase activity http://amigo.geneontology.org/amigo/term/GO:0051348 231 4 1.25234105470675 3.19401810310901 0.0361703331615004 0.285722821050479 836;2167;2932;5468 CASP3;PPARG;GSK3B;FABP4 geneontology_Biological_Process

GO:0043588 skin development http://amigo.geneontology.org/amigo/term/GO:0043588 231 4 1.25234105470675 3.19401810310901 0.0361703331615004 0.285722821050479 239;596;836;8644 CASP3;ALOX12;AKR1C3;BCL2 geneontology_Biological_Process

GO:0072594 establishment of protein localization to organelle http://amigo.geneontology.org/amigo/term/GO:0072594 457 6 2.47757516017743 2.42172269743386 0.0363490461039742 0.285722821050479 596;841;3320;5594;6774;8644 AKR1C3;BCL2;MAPK1;STAT3;HSP90AA1;CASP8 geneontology_Biological_Process

GO:0042446 hormone biosynthetic process http://amigo.geneontology.org/amigo/term/GO:0042446 56 2 0.303597831444061 6.58766233766234 0.0368793924621492 0.285722821050479 231;8644 AKR1B1;AKR1C3 geneontology_Biological_Process

GO:0071242 cellular response to ammonium ion http://amigo.geneontology.org/amigo/term/GO:0071242 56 2 0.303597831444061 6.58766233766234 0.0368793924621492 0.285722821050479 836;5594 CASP3;MAPK1 geneontology_Biological_Process

GO:0033627 cell adhesion mediated by integrin http://amigo.geneontology.org/amigo/term/GO:0033627 56 2 0.303597831444061 6.58766233766234 0.0368793924621492 0.285722821050479 5328;5747 PTK2;PLAU geneontology_Biological_Process

GO:0030104 water homeostasis http://amigo.geneontology.org/amigo/term/GO:0030104 56 2 0.303597831444061 6.58766233766234 0.0368793924621492 0.285722821050479 231;239 AKR1B1;ALOX12 geneontology_Biological_Process

GO:0034637 cellular carbohydrate biosynthetic process http://amigo.geneontology.org/amigo/term/GO:0034637 56 2 0.303597831444061 6.58766233766234 0.0368793924621492 0.285722821050479 231;2932 AKR1B1;GSK3B geneontology_Biological_Process

GO:0002292 T cell differentiation involved in immune response http://amigo.geneontology.org/amigo/term/GO:0002292 56 2 0.303597831444061 6.58766233766234 0.0368793924621492 0.285722821050479 5914;6774 STAT3;RARA geneontology_Biological_Process

GO:0042108 positive regulation of cytokine biosynthetic process http://amigo.geneontology.org/amigo/term/GO:0042108 56 2 0.303597831444061 6.58766233766234 0.0368793924621492 0.285722821050479 6774;9261 MAPKAPK2;STAT3 geneontology_Biological_Process

GO:0031670 cellular response to nutrient http://amigo.geneontology.org/amigo/term/GO:0031670 56 2 0.303597831444061 6.58766233766234 0.0368793924621492 0.285722821050479 4193;5468 PPARG;MDM2 geneontology_Biological_Process

GO:0019693 ribose phosphate metabolic process http://amigo.geneontology.org/amigo/term/GO:0019693 342 5 1.85411532774766 2.69670388091441 0.0371882600792441 0.285722821050479 3939;5141;5465;6319;6774 PPARA;PDE4A;STAT3;LDHA;SCD geneontology_Biological_Process

GO:2001236 regulation of extrinsic apoptotic signaling pathway http://amigo.geneontology.org/amigo/term/GO:2001236 136 3 0.737309019221291 4.06885026737968 0.0372836221423372 0.285722821050479 596;841;2260 BCL2;CASP8;FGFR1 geneontology_Biological_Process

GO:0060405 regulation of penile erection http://amigo.geneontology.org/amigo/term/GO:0060405 7 1 0.0379497289305076 26.3506493506494 0.0373489216219447 0.285722821050479 1268 CNR1 geneontology_Biological_Process

GO:0035333 Notch receptor processing, ligand-dependent http://amigo.geneontology.org/amigo/term/GO:0035333 7 1 0.0379497289305076 26.3506493506494 0.0373489216219447 0.285722821050479 23385 NCSTN geneontology_Biological_Process

GO:0044557 relaxation of smooth muscle http://amigo.geneontology.org/amigo/term/GO:0044557 7 1 0.0379497289305076 26.3506493506494 0.0373489216219447 0.285722821050479 136 ADORA2B geneontology_Biological_Process

GO:0010753 positive regulation of cGMP-mediated signaling http://amigo.geneontology.org/amigo/term/GO:0010753 7 1 0.0379497289305076 26.3506493506494 0.0373489216219447 0.285722821050479 136 ADORA2B geneontology_Biological_Process

GO:0061179 negative regulation of insulin secretion involved in cellular response to glucose stimulus http://amigo.geneontology.org/amigo/term/GO:0061179 7 1 0.0379497289305076 26.3506493506494 0.0373489216219447 0.285722821050479 150 ADRA2A geneontology_Biological_Process

GO:0043651 linoleic acid metabolic process http://amigo.geneontology.org/amigo/term/GO:0043651 7 1 0.0379497289305076 26.3506493506494 0.0373489216219447 0.285722821050479 239 ALOX12 geneontology_Biological_Process

GO:0070166 enamel mineralization http://amigo.geneontology.org/amigo/term/GO:0070166 7 1 0.0379497289305076 26.3506493506494 0.0373489216219447 0.285722821050479 5465 PPARA geneontology_Biological_Process

GO:1904781 positive regulation of protein localization to centrosome http://amigo.geneontology.org/amigo/term/GO:1904781 7 1 0.0379497289305076 26.3506493506494 0.0373489216219447 0.285722821050479 2932 GSK3B geneontology_Biological_Process

GO:0071455 cellular response to hyperoxia http://amigo.geneontology.org/amigo/term/GO:0071455 7 1 0.0379497289305076 26.3506493506494 0.0373489216219447 0.285722821050479 5468 PPARG geneontology_Biological_Process

GO:2000074 regulation of type B pancreatic cell development http://amigo.geneontology.org/amigo/term/GO:2000074 7 1 0.0379497289305076 26.3506493506494 0.0373489216219447 0.285722821050479 2932 GSK3B geneontology_Biological_Process

GO:1905461 positive regulation of vascular associated smooth muscle cell apoptotic process http://amigo.geneontology.org/amigo/term/GO:1905461 7 1 0.0379497289305076 26.3506493506494 0.0373489216219447 0.285722821050479 5468 PPARG geneontology_Biological_Process

GO:0051001 negative regulation of nitric-oxide synthase activity http://amigo.geneontology.org/amigo/term/GO:0051001 7 1 0.0379497289305076 26.3506493506494 0.0373489216219447 0.285722821050479 1268 CNR1 geneontology_Biological_Process

GO:0045924 regulation of female receptivity http://amigo.geneontology.org/amigo/term/GO:0045924 7 1 0.0379497289305076 26.3506493506494 0.0373489216219447 0.285722821050479 7067 THRA geneontology_Biological_Process

GO:0060180 female mating behavior http://amigo.geneontology.org/amigo/term/GO:0060180 7 1 0.0379497289305076 26.3506493506494 0.0373489216219447 0.285722821050479 7067 THRA geneontology_Biological_Process

GO:0008627 intrinsic apoptotic signaling pathway in response to osmotic stress http://amigo.geneontology.org/amigo/term/GO:0008627 7 1 0.0379497289305076 26.3506493506494 0.0373489216219447 0.285722821050479 836 CASP3 geneontology_Biological_Process

GO:0051902 negative regulation of mitochondrial depolarization http://amigo.geneontology.org/amigo/term/GO:0051902 7 1 0.0379497289305076 26.3506493506494 0.0373489216219447 0.285722821050479 596 BCL2 geneontology_Biological_Process

GO:0070305 response to cGMP http://amigo.geneontology.org/amigo/term/GO:0070305 7 1 0.0379497289305076 26.3506493506494 0.0373489216219447 0.285722821050479 5972 REN geneontology_Biological_Process

GO:0030853 negative regulation of granulocyte differentiation http://amigo.geneontology.org/amigo/term/GO:0030853 7 1 0.0379497289305076 26.3506493506494 0.0373489216219447 0.285722821050479 5914 RARA geneontology_Biological_Process

GO:0060687 regulation of branching involved in prostate gland morphogenesis http://amigo.geneontology.org/amigo/term/GO:0060687 7 1 0.0379497289305076 26.3506493506494 0.0373489216219447 0.285722821050479 6256 RXRA geneontology_Biological_Process

GO:0006857 oligopeptide transport http://amigo.geneontology.org/amigo/term/GO:0006857 7 1 0.0379497289305076 26.3506493506494 0.0373489216219447 0.285722821050479 760 CA2 geneontology_Biological_Process

GO:2001225 regulation of chloride transport http://amigo.geneontology.org/amigo/term/GO:2001225 7 1 0.0379497289305076 26.3506493506494 0.0373489216219447 0.285722821050479 760 CA2 geneontology_Biological_Process

GO:0015697 quaternary ammonium group transport http://amigo.geneontology.org/amigo/term/GO:0015697 7 1 0.0379497289305076 26.3506493506494 0.0373489216219447 0.285722821050479 6580 SLC22A1 geneontology_Biological_Process

GO:1903265 positive regulation of tumor necrosis factor-mediated signaling pathway http://amigo.geneontology.org/amigo/term/GO:1903265 7 1 0.0379497289305076 26.3506493506494 0.0373489216219447 0.285722821050479 834 CASP1 geneontology_Biological_Process

GO:0001554 luteolysis http://amigo.geneontology.org/amigo/term/GO:0001554 7 1 0.0379497289305076 26.3506493506494 0.0373489216219447 0.285722821050479 836 CASP3 geneontology_Biological_Process

GO:0045630 positive regulation of T-helper 2 cell differentiation http://amigo.geneontology.org/amigo/term/GO:0045630 7 1 0.0379497289305076 26.3506493506494 0.0373489216219447 0.285722821050479 5914 RARA geneontology_Biological_Process

GO:0035095 behavioral response to nicotine http://amigo.geneontology.org/amigo/term/GO:0035095 7 1 0.0379497289305076 26.3506493506494 0.0373489216219447 0.285722821050479 5465 PPARA geneontology_Biological_Process

GO:0031622 positive regulation of fever generation http://amigo.geneontology.org/amigo/term/GO:0031622 7 1 0.0379497289305076 26.3506493506494 0.0373489216219447 0.285722821050479 1268 CNR1 geneontology_Biological_Process

GO:1905050 positive regulation of metallopeptidase activity http://amigo.geneontology.org/amigo/term/GO:1905050 7 1 0.0379497289305076 26.3506493506494 0.0373489216219447 0.285722821050479 6774 STAT3 geneontology_Biological_Process

GO:0097267 omega-hydroxylase P450 pathway http://amigo.geneontology.org/amigo/term/GO:0097267 7 1 0.0379497289305076 26.3506493506494 0.0373489216219447 0.285722821050479 1544 CYP1A2 geneontology_Biological_Process

GO:0071838 cell proliferation in bone marrow http://amigo.geneontology.org/amigo/term/GO:0071838 7 1 0.0379497289305076 26.3506493506494 0.0373489216219447 0.285722821050479 1636 ACE geneontology_Biological_Process

GO:0035766 cell chemotaxis to fibroblast growth factor http://amigo.geneontology.org/amigo/term/GO:0035766 7 1 0.0379497289305076 26.3506493506494 0.0373489216219447 0.285722821050479 2260 FGFR1 geneontology_Biological_Process

GO:0035768 endothelial cell chemotaxis to fibroblast growth factor http://amigo.geneontology.org/amigo/term/GO:0035768 7 1 0.0379497289305076 26.3506493506494 0.0373489216219447 0.285722821050479 2260 FGFR1 geneontology_Biological_Process

GO:1904847 regulation of cell chemotaxis to fibroblast growth factor http://amigo.geneontology.org/amigo/term/GO:1904847 7 1 0.0379497289305076 26.3506493506494 0.0373489216219447 0.285722821050479 2260 FGFR1 geneontology_Biological_Process

GO:2000544 regulation of endothelial cell chemotaxis to fibroblast growth factor http://amigo.geneontology.org/amigo/term/GO:2000544 7 1 0.0379497289305076 26.3506493506494 0.0373489216219447 0.285722821050479 2260 FGFR1 geneontology_Biological_Process

GO:0060693 regulation of branching involved in salivary gland morphogenesis http://amigo.geneontology.org/amigo/term/GO:0060693 7 1 0.0379497289305076 26.3506493506494 0.0373489216219447 0.285722821050479 2260 FGFR1 geneontology_Biological_Process

GO:0014827 intestine smooth muscle contraction http://amigo.geneontology.org/amigo/term/GO:0014827 7 1 0.0379497289305076 26.3506493506494 0.0373489216219447 0.285722821050479 3357 HTR2B geneontology_Biological_Process

GO:0060534 trachea cartilage development http://amigo.geneontology.org/amigo/term/GO:0060534 7 1 0.0379497289305076 26.3506493506494 0.0373489216219447 0.285722821050479 5914 RARA geneontology_Biological_Process

GO:0060059 embryonic retina morphogenesis in camera-type eye http://amigo.geneontology.org/amigo/term/GO:0060059 7 1 0.0379497289305076 26.3506493506494 0.0373489216219447 0.285722821050479 5950 RBP4 geneontology_Biological_Process

GO:0006776 vitamin A metabolic process http://amigo.geneontology.org/amigo/term/GO:0006776 7 1 0.0379497289305076 26.3506493506494 0.0373489216219447 0.285722821050479 5467 PPARD geneontology_Biological_Process

GO:0006873 cellular ion homeostasis http://amigo.geneontology.org/amigo/term/GO:0006873 462 6 2.5046821094135 2.39551357733176 0.0380271584450413 0.29063354525045 596;760;1268;3357;5594;5739 CNR1;BCL2;MAPK1;CA2;HTR2B;PTGIR geneontology_Biological_Process

GO:0060350 endochondral bone morphogenesis http://amigo.geneontology.org/amigo/term/GO:0060350 57 2 0.309019221291277 6.47208931419458 0.0380900149441388 0.290836163724235 249;5914 ALPL;RARA geneontology_Biological_Process

GO:0031349 positive regulation of defense response http://amigo.geneontology.org/amigo/term/GO:0031349 345 5 1.87037949728931 2.67325428194993 0.0383906338134272 0.292852098927593 136;841;1268;2167;9261 CNR1;ADORA2B;MAPKAPK2;CASP8;FABP4 geneontology_Biological_Process

GO:0000187 activation of MAPK activity http://amigo.geneontology.org/amigo/term/GO:0000187 138 3 0.748151798915722 4.0098814229249 0.0386742579689956 0.294454245735397 136;5594;9261 ADORA2B;MAPKAPK2;MAPK1 geneontology_Biological_Process

GO:0010821 regulation of mitochondrion organization http://amigo.geneontology.org/amigo/term/GO:0010821 138 3 0.748151798915722 4.0098814229249 0.0386742579689956 0.294454245735397 596;841;2932 GSK3B;BCL2;CASP8 geneontology_Biological_Process

GO:0045844 positive regulation of striated muscle tissue development http://amigo.geneontology.org/amigo/term/GO:0045844 58 2 0.314440611138492 6.36050156739812 0.0393157817791789 0.29848660891555 596;2260 BCL2;FGFR1 geneontology_Biological_Process

GO:0048636 positive regulation of muscle organ development http://amigo.geneontology.org/amigo/term/GO:0048636 58 2 0.314440611138492 6.36050156739812 0.0393157817791789 0.29848660891555 596;2260 BCL2;FGFR1 geneontology_Biological_Process

GO:2001021 negative regulation of response to DNA damage stimulus http://amigo.geneontology.org/amigo/term/GO:2001021 58 2 0.314440611138492 6.36050156739812 0.0393157817791789 0.29848660891555 596;4193 BCL2;MDM2 geneontology_Biological_Process

GO:0060759 regulation of response to cytokine stimulus http://amigo.geneontology.org/amigo/term/GO:0060759 139 3 0.753573188762937 3.98103335513407 0.0393794676404471 0.298686729913609 834;841;5468 PPARG;CASP8;CASP1 geneontology_Biological_Process

GO:0010506 regulation of autophagy http://amigo.geneontology.org/amigo/term/GO:0010506 238 4 1.29029078363726 3.10007639419404 0.0396831341814808 0.300704961856259 596;836;3357;5747 CASP3;PTK2;BCL2;HTR2B geneontology_Biological_Process

GO:1901863 positive regulation of muscle tissue development http://amigo.geneontology.org/amigo/term/GO:1901863 59 2 0.319862000985707 6.25269645608629 0.0405564997721525 0.305521612692464 596;2260 BCL2;FGFR1 geneontology_Biological_Process

GO:0045682 regulation of epidermis development http://amigo.geneontology.org/amigo/term/GO:0045682 59 2 0.319862000985707 6.25269645608629 0.0405564997721525 0.305521612692464 5467;9817 KEAP1;PPARD geneontology_Biological_Process

GO:2000573 positive regulation of DNA biosynthetic process http://amigo.geneontology.org/amigo/term/GO:2000573 59 2 0.319862000985707 6.25269645608629 0.0405564997721525 0.305521612692464 3320;5594 MAPK1;HSP90AA1 geneontology_Biological_Process

GO:0048639 positive regulation of developmental growth http://amigo.geneontology.org/amigo/term/GO:0048639 141 3 0.764415968457368 3.92456479690522 0.0408096029493785 0.305521612692464 596;2260;5467 BCL2;FGFR1;PPARD geneontology_Biological_Process

GO:0070661 leukocyte proliferation http://amigo.geneontology.org/amigo/term/GO:0070661 241 4 1.30655495317891 3.06148623161071 0.0412467534394171 0.305521612692464 596;836;1636;23385 CASP3;ACE;NCSTN;BCL2 geneontology_Biological_Process

GO:0051961 negative regulation of nervous system development http://amigo.geneontology.org/amigo/term/GO:0051961 242 4 1.31197634302612 3.0488354620586 0.041775720208007 0.305521612692464 2932;4193;5747;6774 GSK3B;PTK2;STAT3;MDM2 geneontology_Biological_Process

GO:0006094 gluconeogenesis http://amigo.geneontology.org/amigo/term/GO:0006094 60 2 0.325283390832923 6.14848484848485 0.0418119773097886 0.305521612692464 5465;5950 PPARA;RBP4 geneontology_Biological_Process

GO:1903747 regulation of establishment of protein localization to mitochondrion http://amigo.geneontology.org/amigo/term/GO:1903747 60 2 0.325283390832923 6.14848484848485 0.0418119773097886 0.305521612692464 596;841 BCL2;CASP8 geneontology_Biological_Process

GO:0050866 negative regulation of cell activation http://amigo.geneontology.org/amigo/term/GO:0050866 143 3 0.775258748151799 3.86967577876669 0.0422659310749746 0.305521612692464 239;836;1268 CASP3;CNR1;ALOX12 geneontology_Biological_Process

GO:0021543 pallium development http://amigo.geneontology.org/amigo/term/GO:0021543 143 3 0.775258748151799 3.86967577876669 0.0422659310749746 0.305521612692464 836;2932;5914 CASP3;GSK3B;RARA geneontology_Biological_Process

GO:0048511 rhythmic process http://amigo.geneontology.org/amigo/term/GO:0048511 243 4 1.31739773287334 3.03628881406659 0.0423085683775968 0.305521612692464 836;2932;5465;5468 CASP3;PPARG;PPARA;GSK3B geneontology_Biological_Process

GO:0051090 regulation of DNA-binding transcription factor activity http://amigo.geneontology.org/amigo/term/GO:0051090 355 5 1.92459339576146 2.59795134443022 0.0425712674262286 0.305521612692464 140;5468;5594;6774;9817 PPARG;ADORA3;KEAP1;MAPK1;STAT3 geneontology_Biological_Process

GO:0070472 regulation of uterine smooth muscle contraction http://amigo.geneontology.org/amigo/term/GO:0070472 8 1 0.043371118777723 23.0568181818182 0.0425714319110344 0.305521612692464 152 ADRA2C geneontology_Biological_Process

GO:0060020 Bergmann glial cell differentiation http://amigo.geneontology.org/amigo/term/GO:0060020 8 1 0.043371118777723 23.0568181818182 0.0425714319110344 0.305521612692464 5594 MAPK1 geneontology_Biological_Process

GO:0045741 positive regulation of epidermal growth factor-activated receptor activity http://amigo.geneontology.org/amigo/term/GO:0045741 8 1 0.043371118777723 23.0568181818182 0.0425714319110344 0.305521612692464 150 ADRA2A geneontology_Biological_Process

GO:0035655 interleukin-18-mediated signaling pathway http://amigo.geneontology.org/amigo/term/GO:0035655 8 1 0.043371118777723 23.0568181818182 0.0425714319110344 0.305521612692464 240 ALOX5 geneontology_Biological_Process

GO:0071351 cellular response to interleukin-18 http://amigo.geneontology.org/amigo/term/GO:0071351 8 1 0.043371118777723 23.0568181818182 0.0425714319110344 0.305521612692464 240 ALOX5 geneontology_Biological_Process

GO:0097011 cellular response to granulocyte macrophage colony-stimulating factor stimulus http://amigo.geneontology.org/amigo/term/GO:0097011 8 1 0.043371118777723 23.0568181818182 0.0425714319110344 0.305521612692464 5594 MAPK1 geneontology_Biological_Process

GO:0097012 response to granulocyte macrophage colony-stimulating factor http://amigo.geneontology.org/amigo/term/GO:0097012 8 1 0.043371118777723 23.0568181818182 0.0425714319110344 0.305521612692464 5594 MAPK1 geneontology_Biological_Process

GO:2001279 regulation of unsaturated fatty acid biosynthetic process http://amigo.geneontology.org/amigo/term/GO:2001279 8 1 0.043371118777723 23.0568181818182 0.0425714319110344 0.305521612692464 2171 FABP5 geneontology_Biological_Process

GO:0016114 terpenoid biosynthetic process http://amigo.geneontology.org/amigo/term/GO:0016114 8 1 0.043371118777723 23.0568181818182 0.0425714319110344 0.305521612692464 8644 AKR1C3 geneontology_Biological_Process

GO:0009414 response to water deprivation http://amigo.geneontology.org/amigo/term/GO:0009414 8 1 0.043371118777723 23.0568181818182 0.0425714319110344 0.305521612692464 231 AKR1B1 geneontology_Biological_Process

GO:0042415 norepinephrine metabolic process http://amigo.geneontology.org/amigo/term/GO:0042415 8 1 0.043371118777723 23.0568181818182 0.0425714319110344 0.305521612692464 231 AKR1B1 geneontology_Biological_Process

GO:0071472 cellular response to salt stress http://amigo.geneontology.org/amigo/term/GO:0071472 8 1 0.043371118777723 23.0568181818182 0.0425714319110344 0.305521612692464 231 AKR1B1 geneontology_Biological_Process

GO:0071474 cellular hyperosmotic response http://amigo.geneontology.org/amigo/term/GO:0071474 8 1 0.043371118777723 23.0568181818182 0.0425714319110344 0.305521612692464 231 AKR1B1 geneontology_Biological_Process

GO:0060099 regulation of phagocytosis, engulfment http://amigo.geneontology.org/amigo/term/GO:0060099 8 1 0.043371118777723 23.0568181818182 0.0425714319110344 0.305521612692464 5468 PPARG geneontology_Biological_Process

GO:0044362 negative regulation of molecular function in other organism http://amigo.geneontology.org/amigo/term/GO:0044362 8 1 0.043371118777723 23.0568181818182 0.0425714319110344 0.305521612692464 841 CASP8 geneontology_Biological_Process

GO:0052204 negative regulation of molecular function in other organism involved in symbiotic interaction http://amigo.geneontology.org/amigo/term/GO:0052204 8 1 0.043371118777723 23.0568181818182 0.0425714319110344 0.305521612692464 841 CASP8 geneontology_Biological_Process

GO:0071287 cellular response to manganese ion http://amigo.geneontology.org/amigo/term/GO:0071287 8 1 0.043371118777723 23.0568181818182 0.0425714319110344 0.305521612692464 23621 BACE1 geneontology_Biological_Process

GO:0072488 ammonium transmembrane transport http://amigo.geneontology.org/amigo/term/GO:0072488 8 1 0.043371118777723 23.0568181818182 0.0425714319110344 0.305521612692464 6580 SLC22A1 geneontology_Biological_Process

GO:0046007 negative regulation of activated T cell proliferation http://amigo.geneontology.org/amigo/term/GO:0046007 8 1 0.043371118777723 23.0568181818182 0.0425714319110344 0.305521612692464 836 CASP3 geneontology_Biological_Process

GO:0033210 leptin-mediated signaling pathway http://amigo.geneontology.org/amigo/term/GO:0033210 8 1 0.043371118777723 23.0568181818182 0.0425714319110344 0.305521612692464 6774 STAT3 geneontology_Biological_Process

GO:0014041 regulation of neuron maturation http://amigo.geneontology.org/amigo/term/GO:0014041 8 1 0.043371118777723 23.0568181818182 0.0425714319110344 0.305521612692464 596 BCL2 geneontology_Biological_Process

GO:1903431 positive regulation of cell maturation http://amigo.geneontology.org/amigo/term/GO:1903431 8 1 0.043371118777723 23.0568181818182 0.0425714319110344 0.305521612692464 596 BCL2 geneontology_Biological_Process

GO:0033689 negative regulation of osteoblast proliferation http://amigo.geneontology.org/amigo/term/GO:0033689 8 1 0.043371118777723 23.0568181818182 0.0425714319110344 0.305521612692464 596 BCL2 geneontology_Biological_Process

GO:1904180 negative regulation of membrane depolarization http://amigo.geneontology.org/amigo/term/GO:1904180 8 1 0.043371118777723 23.0568181818182 0.0425714319110344 0.305521612692464 596 BCL2 geneontology_Biological_Process

GO:0002866 positive regulation of acute inflammatory response to antigenic stimulus http://amigo.geneontology.org/amigo/term/GO:0002866 8 1 0.043371118777723 23.0568181818182 0.0425714319110344 0.305521612692464 1268 CNR1 geneontology_Biological_Process

GO:0060100 positive regulation of phagocytosis, engulfment http://amigo.geneontology.org/amigo/term/GO:0060100 8 1 0.043371118777723 23.0568181818182 0.0425714319110344 0.305521612692464 5468 PPARG geneontology_Biological_Process

GO:1905155 positive regulation of membrane invagination http://amigo.geneontology.org/amigo/term/GO:1905155 8 1 0.043371118777723 23.0568181818182 0.0425714319110344 0.305521612692464 5468 PPARG geneontology_Biological_Process

GO:0061052 negative regulation of cell growth involved in cardiac muscle cell development http://amigo.geneontology.org/amigo/term/GO:0061052 8 1 0.043371118777723 23.0568181818182 0.0425714319110344 0.305521612692464 5465 PPARA geneontology_Biological_Process

GO:0007172 signal complex assembly http://amigo.geneontology.org/amigo/term/GO:0007172 8 1 0.043371118777723 23.0568181818182 0.0425714319110344 0.305521612692464 5747 PTK2 geneontology_Biological_Process

GO:1902947 regulation of tau-protein kinase activity http://amigo.geneontology.org/amigo/term/GO:1902947 8 1 0.043371118777723 23.0568181818182 0.0425714319110344 0.305521612692464 3320 HSP90AA1 geneontology_Biological_Process

GO:0031620 regulation of fever generation http://amigo.geneontology.org/amigo/term/GO:0031620 8 1 0.043371118777723 23.0568181818182 0.0425714319110344 0.305521612692464 1268 CNR1 geneontology_Biological_Process

GO:2001223 negative regulation of neuron migration http://amigo.geneontology.org/amigo/term/GO:2001223 8 1 0.043371118777723 23.0568181818182 0.0425714319110344 0.305521612692464 6774 STAT3 geneontology_Biological_Process

GO:0042738 exogenous drug catabolic process http://amigo.geneontology.org/amigo/term/GO:0042738 8 1 0.043371118777723 23.0568181818182 0.0425714319110344 0.305521612692464 1544 CYP1A2 geneontology_Biological_Process

GO:0021873 forebrain neuroblast division http://amigo.geneontology.org/amigo/term/GO:0021873 8 1 0.043371118777723 23.0568181818182 0.0425714319110344 0.305521612692464 2260 FGFR1 geneontology_Biological_Process

GO:0044351 macropinocytosis http://amigo.geneontology.org/amigo/term/GO:0044351 8 1 0.043371118777723 23.0568181818182 0.0425714319110344 0.305521612692464 9261 MAPKAPK2 geneontology_Biological_Process

GO:0035278 miRNA mediated inhibition of translation http://amigo.geneontology.org/amigo/term/GO:0035278 8 1 0.043371118777723 23.0568181818182 0.0425714319110344 0.305521612692464 6774 STAT3 geneontology_Biological_Process

GO:0040033 negative regulation of translation, ncRNA-mediated http://amigo.geneontology.org/amigo/term/GO:0040033 8 1 0.043371118777723 23.0568181818182 0.0425714319110344 0.305521612692464 6774 STAT3 geneontology_Biological_Process

GO:0045974 regulation of translation, ncRNA-mediated http://amigo.geneontology.org/amigo/term/GO:0045974 8 1 0.043371118777723 23.0568181818182 0.0425714319110344 0.305521612692464 6774 STAT3 geneontology_Biological_Process

GO:0060638 mesenchymal-epithelial cell signaling http://amigo.geneontology.org/amigo/term/GO:0060638 8 1 0.043371118777723 23.0568181818182 0.0425714319110344 0.305521612692464 2260 FGFR1 geneontology_Biological_Process

GO:0045722 positive regulation of gluconeogenesis http://amigo.geneontology.org/amigo/term/GO:0045722 8 1 0.043371118777723 23.0568181818182 0.0425714319110344 0.305521612692464 5465 PPARA geneontology_Biological_Process

GO:0010511 regulation of phosphatidylinositol biosynthetic process http://amigo.geneontology.org/amigo/term/GO:0010511 8 1 0.043371118777723 23.0568181818182 0.0425714319110344 0.305521612692464 3357 HTR2B geneontology_Biological_Process

GO:0014831 gastro-intestinal system smooth muscle contraction http://amigo.geneontology.org/amigo/term/GO:0014831 8 1 0.043371118777723 23.0568181818182 0.0425714319110344 0.305521612692464 3357 HTR2B geneontology_Biological_Process

GO:0098926 postsynaptic signal transduction http://amigo.geneontology.org/amigo/term/GO:0098926 8 1 0.043371118777723 23.0568181818182 0.0425714319110344 0.305521612692464 6774 STAT3 geneontology_Biological_Process

GO:0099527 postsynapse to nucleus signaling pathway http://amigo.geneontology.org/amigo/term/GO:0099527 8 1 0.043371118777723 23.0568181818182 0.0425714319110344 0.305521612692464 6774 STAT3 geneontology_Biological_Process

GO:0038114 interleukin-21-mediated signaling pathway http://amigo.geneontology.org/amigo/term/GO:0038114 8 1 0.043371118777723 23.0568181818182 0.0425714319110344 0.305521612692464 6774 STAT3 geneontology_Biological_Process

GO:0098756 response to interleukin-21 http://amigo.geneontology.org/amigo/term/GO:0098756 8 1 0.043371118777723 23.0568181818182 0.0425714319110344 0.305521612692464 6774 STAT3 geneontology_Biological_Process

GO:0098757 cellular response to interleukin-21 http://amigo.geneontology.org/amigo/term/GO:0098757 8 1 0.043371118777723 23.0568181818182 0.0425714319110344 0.305521612692464 6774 STAT3 geneontology_Biological_Process

GO:0002429 immune response-activating cell surface receptor signaling pathway http://amigo.geneontology.org/amigo/term/GO:0002429 244 4 1.32281912272055 3.02384500745156 0.0428452984230535 0.307211539409744 596;3320;5594;5747 PTK2;BCL2;MAPK1;HSP90AA1 geneontology_Biological_Process

GO:0060193 positive regulation of lipase activity http://amigo.geneontology.org/amigo/term/GO:0060193 61 2 0.330704780680138 6.04769001490313 0.0430820243497018 0.308080749639244 2260;3357 FGFR1;HTR2B geneontology_Biological_Process

GO:0010660 regulation of muscle cell apoptotic process http://amigo.geneontology.org/amigo/term/GO:0010660 61 2 0.330704780680138 6.04769001490313 0.0430820243497018 0.308080749639244 239;5468 PPARG;ALOX12 geneontology_Biological_Process

GO:0032755 positive regulation of interleukin-6 production http://amigo.geneontology.org/amigo/term/GO:0032755 61 2 0.330704780680138 6.04769001490313 0.0430820243497018 0.308080749639244 136;6774 ADORA2B;STAT3 geneontology_Biological_Process

GO:0006417 regulation of translation http://amigo.geneontology.org/amigo/term/GO:0006417 245 4 1.32824051256777 3.01150278293135 0.0433859106464093 0.309976836600506 5594;5914;6256;6774 MAPK1;STAT3;RXRA;RARA geneontology_Biological_Process

GO:0048167 regulation of synaptic plasticity http://amigo.geneontology.org/amigo/term/GO:0048167 145 3 0.78610152784623 3.81630094043887 0.0437483319372066 0.311731212966632 5594;5641;5914 MAPK1;LGMN;RARA geneontology_Biological_Process

GO:0051100 negative regulation of binding http://amigo.geneontology.org/amigo/term/GO:0051100 145 3 0.78610152784623 3.81630094043887 0.0437483319372066 0.311731212966632 1636;2932;5465 ACE;PPARA;GSK3B geneontology_Biological_Process

GO:0030278 regulation of ossification http://amigo.geneontology.org/amigo/term/GO:0030278 145 3 0.78610152784623 3.81630094043887 0.0437483319372066 0.311731212966632 596;5594;5747 PTK2;BCL2;MAPK1 geneontology_Biological_Process

GO:0007204 positive regulation of cytosolic calcium ion concentration http://amigo.geneontology.org/amigo/term/GO:0007204 246 4 1.33366190241498 2.99926090169993 0.0439304051776801 0.312472090872708 596;1268;3357;5739 CNR1;BCL2;HTR2B;PTGIR geneontology_Biological_Process

GO:0006650 glycerophospholipid metabolic process http://amigo.geneontology.org/amigo/term/GO:0006650 246 4 1.33366190241498 2.99926090169993 0.0439304051776801 0.312472090872708 43;2171;2260;3357 ACHE;FABP5;FGFR1;HTR2B geneontology_Biological_Process

GO:1903578 regulation of ATP metabolic process http://amigo.geneontology.org/amigo/term/GO:1903578 62 2 0.336126170527353 5.95014662756598 0.0443664524094987 0.31473435477022 5465;6774 PPARA;STAT3 geneontology_Biological_Process

GO:0021954 central nervous system neuron development http://amigo.geneontology.org/amigo/term/GO:0021954 62 2 0.336126170527353 5.95014662756598 0.0443664524094987 0.31473435477022 3320;5747 PTK2;HSP90AA1 geneontology_Biological_Process

GO:0007004 telomere maintenance via telomerase http://amigo.geneontology.org/amigo/term/GO:0007004 62 2 0.336126170527353 5.95014662756598 0.0443664524094987 0.31473435477022 3320;5594 MAPK1;HSP90AA1 geneontology_Biological_Process

GO:0051650 establishment of vesicle localization http://amigo.geneontology.org/amigo/term/GO:0051650 248 4 1.34450468210941 2.97507331378299 0.0450310408289093 0.319165977602243 136;1268;2932;23621 BACE1;GSK3B;CNR1;ADORA2B geneontology_Biological_Process

GO:0048729 tissue morphogenesis http://amigo.geneontology.org/amigo/term/GO:0048729 484 6 2.62395268605224 2.28662659654395 0.0460033685232486 0.325574537067848 596;760;2260;4193;5914;6256 BCL2;RXRA;CA2;MDM2;FGFR1;RARA geneontology_Biological_Process

GO:0061136 regulation of proteasomal protein catabolic process http://amigo.geneontology.org/amigo/term/GO:0061136 148 3 0.802365697387876 3.73894348894349 0.0460205408261292 0.325574537067848 2932;4193;9817 GSK3B;KEAP1;MDM2 geneontology_Biological_Process

GO:0006914 autophagy http://amigo.geneontology.org/amigo/term/GO:0006914 363 5 1.96796451453918 2.54069621838217 0.0461085430386785 0.325574537067848 596;836;3320;3357;5747 CASP3;PTK2;BCL2;HSP90AA1;HTR2B geneontology_Biological_Process

GO:0061919 process utilizing autophagic mechanism http://amigo.geneontology.org/amigo/term/GO:0061919 363 5 1.96796451453918 2.54069621838217 0.0461085430386785 0.325574537067848 596;836;3320;3357;5747 CASP3;PTK2;BCL2;HSP90AA1;HTR2B geneontology_Biological_Process

GO:0031099 regeneration http://amigo.geneontology.org/amigo/term/GO:0031099 149 3 0.807787087235091 3.71384990848078 0.0467908389233755 0.325574537067848 596;5467;5468 PPARG;BCL2;PPARD geneontology_Biological_Process

GO:0060411 cardiac septum morphogenesis http://amigo.geneontology.org/amigo/term/GO:0060411 64 2 0.346968950221784 5.76420454545454 0.0469777053942548 0.325574537067848 4193;5914 MDM2;RARA geneontology_Biological_Process

GO:0006970 response to osmotic stress http://amigo.geneontology.org/amigo/term/GO:0006970 64 2 0.346968950221784 5.76420454545454 0.0469777053942548 0.325574537067848 231;836 CASP3;AKR1B1 geneontology_Biological_Process

GO:0043367 CD4-positive, alpha-beta T cell differentiation http://amigo.geneontology.org/amigo/term/GO:0043367 64 2 0.346968950221784 5.76420454545454 0.0469777053942548 0.325574537067848 5914;6774 STAT3;RARA geneontology_Biological_Process

GO:0006278 RNA-dependent DNA biosynthetic process http://amigo.geneontology.org/amigo/term/GO:0006278 64 2 0.346968950221784 5.76420454545454 0.0469777053942548 0.325574537067848 3320;5594 MAPK1;HSP90AA1 geneontology_Biological_Process

GO:0031652 positive regulation of heat generation http://amigo.geneontology.org/amigo/term/GO:0031652 9 1 0.0487925086249384 20.4949494949495 0.0477661217842607 0.325574537067848 1268 CNR1 geneontology_Biological_Process

GO:0070471 uterine smooth muscle contraction http://amigo.geneontology.org/amigo/term/GO:0070471 9 1 0.0487925086249384 20.4949494949495 0.0477661217842607 0.325574537067848 152 ADRA2C geneontology_Biological_Process

GO:0002676 regulation of chronic inflammatory response http://amigo.geneontology.org/amigo/term/GO:0002676 9 1 0.0487925086249384 20.4949494949495 0.0477661217842607 0.325574537067848 136 ADORA2B geneontology_Biological_Process

GO:0031284 positive regulation of guanylate cyclase activity http://amigo.geneontology.org/amigo/term/GO:0031284 9 1 0.0487925086249384 20.4949494949495 0.0477661217842607 0.325574537067848 136 ADORA2B geneontology_Biological_Process

GO:0032000 positive regulation of fatty acid beta-oxidation http://amigo.geneontology.org/amigo/term/GO:0032000 9 1 0.0487925086249384 20.4949494949495 0.0477661217842607 0.325574537067848 5465 PPARA geneontology_Biological_Process

GO:0042538 hyperosmotic salinity response http://amigo.geneontology.org/amigo/term/GO:0042538 9 1 0.0487925086249384 20.4949494949495 0.0477661217842607 0.325574537067848 231 AKR1B1 geneontology_Biological_Process

GO:0035358 regulation of peroxisome proliferator activated receptor signaling pathway http://amigo.geneontology.org/amigo/term/GO:0035358 9 1 0.0487925086249384 20.4949494949495 0.0477661217842607 0.325574537067848 2171 FABP5 geneontology_Biological_Process

GO:1905153 regulation of membrane invagination http://amigo.geneontology.org/amigo/term/GO:1905153 9 1 0.0487925086249384 20.4949494949495 0.0477661217842607 0.325574537067848 5468 PPARG geneontology_Biological_Process

GO:0044359 modulation of molecular function in other organism http://amigo.geneontology.org/amigo/term/GO:0044359 9 1 0.0487925086249384 20.4949494949495 0.0477661217842607 0.325574537067848 841 CASP8 geneontology_Biological_Process

GO:0052205 modulation of molecular function in other organism involved in symbiotic interaction http://amigo.geneontology.org/amigo/term/GO:0052205 9 1 0.0487925086249384 20.4949494949495 0.0477661217842607 0.325574537067848 841 CASP8 geneontology_Biological_Process

GO:1902004 positive regulation of amyloid-beta formation http://amigo.geneontology.org/amigo/term/GO:1902004 9 1 0.0487925086249384 20.4949494949495 0.0477661217842607 0.325574537067848 836 CASP3 geneontology_Biological_Process

GO:2001269 positive regulation of cysteine-type endopeptidase activity involved in apoptotic signaling pathway http://amigo.geneontology.org/amigo/term/GO:2001269 9 1 0.0487925086249384 20.4949494949495 0.0477661217842607 0.325574537067848 841 CASP8 geneontology_Biological_Process

GO:0060019 radial glial cell differentiation http://amigo.geneontology.org/amigo/term/GO:0060019 9 1 0.0487925086249384 20.4949494949495 0.0477661217842607 0.325574537067848 6774 STAT3 geneontology_Biological_Process

GO:0036462 TRAIL-activated apoptotic signaling pathway http://amigo.geneontology.org/amigo/term/GO:0036462 9 1 0.0487925086249384 20.4949494949495 0.0477661217842607 0.325574537067848 841 CASP8 geneontology_Biological_Process

GO:1905288 vascular associated smooth muscle cell apoptotic process http://amigo.geneontology.org/amigo/term/GO:1905288 9 1 0.0487925086249384 20.4949494949495 0.0477661217842607 0.325574537067848 5468 PPARG geneontology_Biological_Process

GO:1905459 regulation of vascular associated smooth muscle cell apoptotic process http://amigo.geneontology.org/amigo/term/GO:1905459 9 1 0.0487925086249384 20.4949494949495 0.0477661217842607 0.325574537067848 5468 PPARG geneontology_Biological_Process

GO:0043497 regulation of protein heterodimerization activity http://amigo.geneontology.org/amigo/term/GO:0043497 9 1 0.0487925086249384 20.4949494949495 0.0477661217842607 0.325574537067848 596 BCL2 geneontology_Biological_Process

GO:0048742 regulation of skeletal muscle fiber development http://amigo.geneontology.org/amigo/term/GO:0048742 9 1 0.0487925086249384 20.4949494949495 0.0477661217842607 0.325574537067848 596 BCL2 geneontology_Biological_Process

GO:1902969 mitotic DNA replication http://amigo.geneontology.org/amigo/term/GO:1902969 9 1 0.0487925086249384 20.4949494949495 0.0477661217842607 0.325574537067848 2260 FGFR1 geneontology_Biological_Process

GO:0050966 detection of mechanical stimulus involved in sensory perception of pain http://amigo.geneontology.org/amigo/term/GO:0050966 9 1 0.0487925086249384 20.4949494949495 0.0477661217842607 0.325574537067848 23621 BACE1 geneontology_Biological_Process

GO:0071550 death-inducing signaling complex assembly http://amigo.geneontology.org/amigo/term/GO:0071550 9 1 0.0487925086249384 20.4949494949495 0.0477661217842607 0.325574537067848 841 CASP8 geneontology_Biological_Process

GO:0072584 caveolin-mediated endocytosis http://amigo.geneontology.org/amigo/term/GO:0072584 9 1 0.0487925086249384 20.4949494949495 0.0477661217842607 0.325574537067848 5594 MAPK1 geneontology_Biological_Process

GO:0009214 cyclic nucleotide catabolic process http://amigo.geneontology.org/amigo/term/GO:0009214 9 1 0.0487925086249384 20.4949494949495 0.0477661217842607 0.325574537067848 5141 PDE4A geneontology_Biological_Process

GO:0033004 negative regulation of mast cell activation http://amigo.geneontology.org/amigo/term/GO:0033004 9 1 0.0487925086249384 20.4949494949495 0.0477661217842607 0.325574537067848 1268 CNR1 geneontology_Biological_Process

GO:0099550 trans-synaptic signaling, modulating synaptic transmission http://amigo.geneontology.org/amigo/term/GO:0099550 9 1 0.0487925086249384 20.4949494949495 0.0477661217842607 0.325574537067848 1268 CNR1 geneontology_Biological_Process

GO:1904338 regulation of dopaminergic neuron differentiation http://amigo.geneontology.org/amigo/term/GO:1904338 9 1 0.0487925086249384 20.4949494949495 0.0477661217842607 0.325574537067848 2932 GSK3B geneontology_Biological_Process

GO:0019373 epoxygenase P450 pathway http://amigo.geneontology.org/amigo/term/GO:0019373 9 1 0.0487925086249384 20.4949494949495 0.0477661217842607 0.325574537067848 1544 CYP1A2 geneontology_Biological_Process

GO:0038007 netrin-activated signaling pathway http://amigo.geneontology.org/amigo/term/GO:0038007 9 1 0.0487925086249384 20.4949494949495 0.0477661217842607 0.325574537067848 5747 PTK2 geneontology_Biological_Process

GO:0045410 positive regulation of interleukin-6 biosynthetic process http://amigo.geneontology.org/amigo/term/GO:0045410 9 1 0.0487925086249384 20.4949494949495 0.0477661217842607 0.325574537067848 6774 STAT3 geneontology_Biological_Process

GO:0051974 negative regulation of telomerase activity http://amigo.geneontology.org/amigo/term/GO:0051974 9 1 0.0487925086249384 20.4949494949495 0.0477661217842607 0.325574537067848 5468 PPARG geneontology_Biological_Process

GO:0060117 auditory receptor cell development http://amigo.geneontology.org/amigo/term/GO:0060117 9 1 0.0487925086249384 20.4949494949495 0.0477661217842607 0.325574537067848 2260 FGFR1 geneontology_Biological_Process

GO:1900402 regulation of carbohydrate metabolic process by regulation of transcription from RNA polymerase II promoter http://amigo.geneontology.org/amigo/term/GO:1900402 9 1 0.0487925086249384 20.4949494949495 0.0477661217842607 0.325574537067848 5465 PPARA geneontology_Biological_Process

GO:0045713 low-density lipoprotein particle receptor biosynthetic process http://amigo.geneontology.org/amigo/term/GO:0045713 9 1 0.0487925086249384 20.4949494949495 0.0477661217842607 0.325574537067848 5468 PPARG geneontology_Biological_Process

GO:0038113 interleukin-9-mediated signaling pathway http://amigo.geneontology.org/amigo/term/GO:0038113 9 1 0.0487925086249384 20.4949494949495 0.0477661217842607 0.325574537067848 6774 STAT3 geneontology_Biological_Process

GO:0071355 cellular response to interleukin-9 http://amigo.geneontology.org/amigo/term/GO:0071355 9 1 0.0487925086249384 20.4949494949495 0.0477661217842607 0.325574537067848 6774 STAT3 geneontology_Biological_Process

GO:0038155 interleukin-23-mediated signaling pathway http://amigo.geneontology.org/amigo/term/GO:0038155 9 1 0.0487925086249384 20.4949494949495 0.0477661217842607 0.325574537067848 6774 STAT3 geneontology_Biological_Process

GO:0010517 regulation of phospholipase activity http://amigo.geneontology.org/amigo/term/GO:0010517 65 2 0.352390340068999 5.67552447552448 0.0483041610573124 0.328402631079536 2260;3357 FGFR1;HTR2B geneontology_Biological_Process

GO:0014032 neural crest cell development http://amigo.geneontology.org/amigo/term/GO:0014032 65 2 0.352390340068999 5.67552447552448 0.0483041610573124 0.328402631079536 3357;5594 MAPK1;HTR2B geneontology_Biological_Process

GO:0010657 muscle cell apoptotic process http://amigo.geneontology.org/amigo/term/GO:0010657 65 2 0.352390340068999 5.67552447552448 0.0483041610573124 0.328402631079536 239;5468 PPARG;ALOX12 geneontology_Biological_Process

GO:0031348 negative regulation of defense response http://amigo.geneontology.org/amigo/term/GO:0031348 151 3 0.818629866929522 3.66465984346779 0.0483506728828773 0.328439800007457 5465;5467;5468 PPARG;PPARA;PPARD geneontology_Biological_Process

GO:0051216 cartilage development http://amigo.geneontology.org/amigo/term/GO:0051216 152 3 0.824051256776737 3.64055023923445 0.0491401724643747 0.332954834936432 2260;5914;7067 FGFR1;RARA;THRA geneontology_Biological_Process

GO:2001235 positive regulation of apoptotic signaling pathway http://amigo.geneontology.org/amigo/term/GO:2001235 152 3 0.824051256776737 3.64055023923445 0.0491401724643747 0.332954834936432 596;841;2932 GSK3B;BCL2;CASP8 geneontology_Biological_Process

GO:0051170 import into nucleus http://amigo.geneontology.org/amigo/term/GO:0051170 152 3 0.824051256776737 3.64055023923445 0.0491401724643747 0.332954834936432 5594;6774;8644 AKR1C3;MAPK1;STAT3 geneontology_Biological_Process

GO:0018958 phenol-containing compound metabolic process http://amigo.geneontology.org/amigo/term/GO:0018958 66 2 0.357811729916215 5.58953168044077 0.0496442591951411 0.335234904708455 231;596 AKR1B1;BCL2 geneontology_Biological_Process

GO:0048708 astrocyte differentiation http://amigo.geneontology.org/amigo/term/GO:0048708 66 2 0.357811729916215 5.58953168044077 0.0496442591951411 0.335234904708455 5594;6774 MAPK1;STAT3 geneontology_Biological_Process

GO:0051193 regulation of cofactor metabolic process http://amigo.geneontology.org/amigo/term/GO:0051193 66 2 0.357811729916215 5.58953168044077 0.0496442591951411 0.335234904708455 5465;6774 PPARA;STAT3 geneontology_Biological_Process

GO:0071277 cellular response to calcium ion http://amigo.geneontology.org/amigo/term/GO:0071277 66 2 0.357811729916215 5.58953168044077 0.0496442591951411 0.335234904708455 5641;8644 AKR1C3;LGMN geneontology_Biological_Process

GO:0050679 positive regulation of epithelial cell proliferation http://amigo.geneontology.org/amigo/term/GO:0050679 153 3 0.829472646623953 3.61675579322638 0.0499360356563618 0.336920874639298 2260;3357;6774 STAT3;FGFR1;HTR2B geneontology_Biological_Process

GO:0090575 RNA polymerase II transcription factor complex http://amigo.geneontology.org/amigo/term/GO:0090575 155 7 0.707281553398058 9.89704873026767 5.79217562812762e-06 0.00579217562812762 5465;5467;5468;5914;6256;6774;7067 PPARG;PPARA;STAT3;RXRA;PPARD;RARA;THRA geneontology_Cellular_Component

GO:0044798 nuclear transcription factor complex http://amigo.geneontology.org/amigo/term/GO:0044798 187 7 0.853300970873786 8.20343611332347 1.9778214586319e-05 0.0098891072931595 5465;5467;5468;5914;6256;6774;7067 PPARG;PPARA;STAT3;RXRA;PPARD;RARA;THRA geneontology_Cellular_Component

GO:0000323 lytic vacuole http://amigo.geneontology.org/amigo/term/GO:0000323 451 9 2.05796116504854 4.37326036703307 0.000168574833340873 0.0421437083352183 231;1508;1636;2171;3320;5594;5641;23385;23621 ACE;AKR1B1;BACE1;FABP5;NCSTN;CTSB;MAPK1;HSP90AA1;LGMN geneontology_Cellular_Component

GO:0005764 lysosome http://amigo.geneontology.org/amigo/term/GO:0005764 451 9 2.05796116504854 4.37326036703307 0.000168574833340873 0.0421437083352183 231;1508;1636;2171;3320;5594;5641;23385;23621 ACE;AKR1B1;BACE1;FABP5;NCSTN;CTSB;MAPK1;HSP90AA1;LGMN geneontology_Cellular_Component

GO:0005775 vacuolar lumen http://amigo.geneontology.org/amigo/term/GO:0005775 125 5 0.570388349514563 8.76595744680851 0.000247821210560106 0.0495642421120213 1508;2171;3320;5594;5641 FABP5;CTSB;MAPK1;HSP90AA1;LGMN geneontology_Cellular_Component

GO:0005641 nuclear envelope lumen http://amigo.geneontology.org/amigo/term/GO:0005641 7 2 0.0319417475728155 62.6139817629179 0.000421804568738526 0.0703007614564211 240;9536 PTGES;ALOX5 geneontology_Cellular_Component

GO:0005667 transcription factor complex http://amigo.geneontology.org/amigo/term/GO:0005667 322 7 1.46932038834951 4.7641073080481 0.000586295846026763 0.0837565494323947 5465;5467;5468;5914;6256;6774;7067 PPARG;PPARA;STAT3;RXRA;PPARD;RARA;THRA geneontology_Cellular_Component

GO:0031264 death-inducing signaling complex http://amigo.geneontology.org/amigo/term/GO:0031264 9 2 0.0410679611650485 48.6997635933806 0.00071889344439835 0.0898616805497937 836;841 CASP3;CASP8 geneontology_Cellular_Component

GO:1904813 ficolin-1-rich granule lumen http://amigo.geneontology.org/amigo/term/GO:1904813 108 4 0.492815533980583 8.11662726556344 0.00144163493746396 0.160181659718218 240;1508;3320;5594 ALOX5;CTSB;MAPK1;HSP90AA1 geneontology_Cellular_Component

GO:0036019 endolysosome http://amigo.geneontology.org/amigo/term/GO:0036019 18 2 0.0821359223300971 24.3498817966903 0.00297637169670883 0.277570800849376 1508;5641 CTSB;LGMN geneontology_Cellular_Component

GO:0043202 lysosomal lumen http://amigo.geneontology.org/amigo/term/GO:0043202 64 3 0.292038834951456 10.2726063829787 0.00305327880934314 0.277570800849376 1508;3320;5641 CTSB;HSP90AA1;LGMN geneontology_Cellular_Component

GO:0043209 myelin sheath http://amigo.geneontology.org/amigo/term/GO:0043209 136 4 0.620582524271845 6.44555694618273 0.003342018389771 0.278501532480916 231;596;760;3320 AKR1B1;BCL2;HSP90AA1;CA2 geneontology_Cellular_Component

GO:0101002 ficolin-1-rich granule http://amigo.geneontology.org/amigo/term/GO:0101002 152 4 0.693592233009709 5.76707726763718 0.00496875624616955 0.371762589052702 240;1508;3320;5594 ALOX5;CTSB;MAPK1;HSP90AA1 geneontology_Cellular_Component

GO:0044437 vacuolar part http://amigo.geneontology.org/amigo/term/GO:0044437 355 6 1.61990291262136 3.70392568175007 0.0052313478132523 0.371762589052702 1508;2171;3320;5594;5641;23385 FABP5;NCSTN;CTSB;MAPK1;HSP90AA1;LGMN geneontology_Cellular_Component

GO:0030424 axon http://amigo.geneontology.org/amigo/term/GO:0030424 495 7 2.25873786407767 3.09907586503331 0.0067173347292091 0.371762589052702 152;760;1268;2932;3320;5594;23621 BACE1;GSK3B;CNR1;ADRA2C;MAPK1;HSP90AA1;CA2 geneontology_Cellular_Component

GO:0045121 membrane raft http://amigo.geneontology.org/amigo/term/GO:0045121 273 5 1.24572815533981 4.01371677967423 0.00779339460031525 0.371762589052702 836;841;1268;5594;23621 CASP3;BACE1;CNR1;MAPK1;CASP8 geneontology_Cellular_Component

GO:0098857 membrane microdomain http://amigo.geneontology.org/amigo/term/GO:0098857 274 5 1.25029126213592 3.99906817828855 0.00791146955392852 0.371762589052702 836;841;1268;5594;23621 CASP3;BACE1;CNR1;MAPK1;CASP8 geneontology_Cellular_Component

GO:0060205 cytoplasmic vesicle lumen http://amigo.geneontology.org/amigo/term/GO:0060205 278 5 1.26854368932039 3.94152762896066 0.00839630858898044 0.371762589052702 240;2171;3320;5594;23621 BACE1;FABP5;ALOX5;MAPK1;HSP90AA1 geneontology_Cellular_Component

GO:0031983 vesicle lumen http://amigo.geneontology.org/amigo/term/GO:0031983 279 5 1.27310679611651 3.92740028978876 0.00852068615563373 0.371762589052702 240;2171;3320;5594;23621 BACE1;FABP5;ALOX5;MAPK1;HSP90AA1 geneontology_Cellular_Component

GO:0042470 melanosome http://amigo.geneontology.org/amigo/term/GO:0042470 93 3 0.424368932038835 7.06932052161977 0.00867320179537145 0.371762589052702 1508;3320;23385 NCSTN;CTSB;HSP90AA1 geneontology_Cellular_Component

GO:0048770 pigment granule http://amigo.geneontology.org/amigo/term/GO:0048770 93 3 0.424368932038835 7.06932052161977 0.00867320179537145 0.371762589052702 1508;3320;23385 NCSTN;CTSB;HSP90AA1 geneontology_Cellular_Component

GO:0031904 endosome lumen http://amigo.geneontology.org/amigo/term/GO:0031904 31 2 0.141456310679612 14.1386410432395 0.00871133590890238 0.371762589052702 1508;5641 CTSB;LGMN geneontology_Cellular_Component

GO:0098589 membrane region http://amigo.geneontology.org/amigo/term/GO:0098589 281 5 1.28223300970874 3.89944726281517 0.00877328858340187 0.371762589052702 836;841;1268;5594;23621 CASP3;BACE1;CNR1;MAPK1;CASP8 geneontology_Cellular_Component

GO:0000790 nuclear chromatin http://amigo.geneontology.org/amigo/term/GO:0000790 285 5 1.3004854368932 3.84471817842479 0.00929406472631755 0.371762589052702 5467;5914;6256;6774;55193 STAT3;RXRA;PBRM1;PPARD;RARA geneontology_Cellular_Component

GO:0098984 neuron to neuron synapse http://amigo.geneontology.org/amigo/term/GO:0098984 285 5 1.3004854368932 3.84471817842479 0.00929406472631755 0.371762589052702 152;2171;5594;6774;23621 BACE1;ADRA2C;FABP5;MAPK1;STAT3 geneontology_Cellular_Component

GO:0098978 glutamatergic synapse http://amigo.geneontology.org/amigo/term/GO:0098978 300 5 1.36893203883495 3.65248226950355 0.0114382501795204 0.439932699212322 136;152;1268;2932;6774 GSK3B;CNR1;ADORA2B;ADRA2C;STAT3 geneontology_Cellular_Component

GO:0033293 monocarboxylic acid binding http://amigo.geneontology.org/amigo/term/GO:0033293 45 7 0.251298128735182 27.8553606237817 4.45835812712403e-09 3.04865656956347e-06 2167;2171;5465;5467;5468;5914;6256 PPARG;PPARA;FABP5;RXRA;FABP4;PPARD;RARA geneontology_Molecular_Function

GO:0004879 nuclear receptor activity http://amigo.geneontology.org/amigo/term/GO:0004879 47 7 0.262466934456745 26.6700261291527 6.12590067561314e-09 3.04865656956347e-06 5465;5467;5468;5914;6256;6774;7067 PPARG;PPARA;STAT3;RXRA;PPARD;RARA;THRA geneontology_Molecular_Function

GO:0098531 transcription factor activity, direct ligand regulated sequence-specific DNA binding http://amigo.geneontology.org/amigo/term/GO:0098531 47 7 0.262466934456745 26.6700261291527 6.12590067561314e-09 3.04865656956347e-06 5465;5467;5468;5914;6256;6774;7067 PPARG;PPARA;STAT3;RXRA;PPARD;RARA;THRA geneontology_Molecular_Function

GO:0005504 fatty acid binding http://amigo.geneontology.org/amigo/term/GO:0005504 24 5 0.134025668658764 37.3062865497076 1.77970770454117e-07 6.64275900719991e-05 2167;2171;5465;5467;5468 PPARG;PPARA;FABP5;FABP4;PPARD geneontology_Molecular_Function

GO:0003707 steroid hormone receptor activity http://amigo.geneontology.org/amigo/term/GO:0003707 51 6 0.284804545899873 21.0670794633643 3.435027866594e-07 9.05809134437904e-05 5465;5467;5468;5914;6256;7067 PPARG;PPARA;RXRA;PPARD;RARA;THRA geneontology_Molecular_Function

GO:0004175 endopeptidase activity http://amigo.geneontology.org/amigo/term/GO:0004175 283 11 1.58038600960125 6.96032484036948 3.64022425092259e-07 9.05809134437904e-05 834;836;841;1508;1636;5328;5550;5641;5972;23385;23621 CASP3;ACE;REN;BACE1;PREP;NCSTN;CTSB;CASP8;CASP1;PLAU;LGMN geneontology_Molecular_Function

GO:0005501 retinoid binding http://amigo.geneontology.org/amigo/term/GO:0005501 21 4 0.117272460076418 34.1086048454469 4.87331306420202e-06 0.00103940805783623 2171;5914;5950;6256 FABP5;RXRA;RBP4;RARA geneontology_Molecular_Function

GO:0019840 isoprenoid binding http://amigo.geneontology.org/amigo/term/GO:0019840 22 4 0.1228568629372 32.5582137161085 5.93155895500797e-06 0.00110697718997836 2171;5914;5950;6256 FABP5;RXRA;RBP4;RARA geneontology_Molecular_Function

GO:0070011 peptidase activity, acting on L-amino acid peptides http://amigo.geneontology.org/amigo/term/GO:0070011 392 11 2.18908592142647 5.0249283924096 8.74319876431606e-06 0.00135354960007552 834;836;841;1508;1636;5328;5550;5641;5972;23385;23621 CASP3;ACE;REN;BACE1;PREP;NCSTN;CTSB;CASP8;CASP1;PLAU;LGMN geneontology_Molecular_Function

GO:0097199 cysteine-type endopeptidase activity involved in apoptotic signaling pathway http://amigo.geneontology.org/amigo/term/GO:0097199 8 3 0.0446752228862545 67.1513157894737 9.06597186922653e-06 0.00135354960007552 834;836;841 CASP3;CASP8;CASP1 geneontology_Molecular_Function

GO:0031406 carboxylic acid binding http://amigo.geneontology.org/amigo/term/GO:0031406 138 7 0.770647594787891 9.08326976862446 1.06671496538091e-05 0.0014466277751375 2167;2171;5465;5467;5468;5914;6256 PPARG;PPARA;FABP5;RXRA;FABP4;PPARD;RARA geneontology_Molecular_Function

GO:0008233 peptidase activity http://amigo.geneontology.org/amigo/term/GO:0008233 404 11 2.25609875575585 4.87567309362515 1.16272828544206e-05 0.0014466277751375 834;836;841;1508;1636;5328;5550;5641;5972;23385;23621 CASP3;ACE;REN;BACE1;PREP;NCSTN;CTSB;CASP8;CASP1;PLAU;LGMN geneontology_Molecular_Function

GO:0043177 organic acid binding http://amigo.geneontology.org/amigo/term/GO:0043177 146 7 0.815322817674145 8.58555635664504 1.54219109727016e-05 0.00177114716017258 2167;2171;5465;5467;5468;5914;6256 PPARG;PPARA;FABP5;RXRA;FABP4;PPARD;RARA geneontology_Molecular_Function

GO:0036041 long-chain fatty acid binding http://amigo.geneontology.org/amigo/term/GO:0036041 10 3 0.0558440286078182 53.7210526315789 1.92733341044526e-05 0.00183113684868945 2167;5467;5468 PPARG;FABP4;PPARD geneontology_Molecular_Function

GO:0001972 retinoic acid binding http://amigo.geneontology.org/amigo/term/GO:0001972 10 3 0.0558440286078182 53.7210526315789 1.92733341044526e-05 0.00183113684868945 2171;5914;6256 FABP5;RXRA;RARA geneontology_Molecular_Function

GO:0030374 nuclear receptor transcription coactivator activity http://amigo.geneontology.org/amigo/term/GO:0030374 60 5 0.335064171646909 14.922514619883 1.96237036698133e-05 0.00183113684868945 5465;5467;5468;5914;7067 PPARG;PPARA;PPARD;RARA;THRA geneontology_Molecular_Function

GO:0001103 RNA polymerase II repressing transcription factor binding http://amigo.geneontology.org/amigo/term/GO:0001103 31 4 0.173116488684236 23.1058290888512 2.45775962799399e-05 0.00215849124976178 5465;5467;5468;6774 PPARG;PPARA;STAT3;PPARD geneontology_Molecular_Function

GO:0019902 phosphatase binding http://amigo.geneontology.org/amigo/term/GO:0019902 159 7 0.887920054864309 7.88359262937217 2.68157301859251e-05 0.00222421584264368 596;5465;5468;5594;5747;6098;6774 PPARG;PPARA;PTK2;BCL2;MAPK1;STAT3;ROS1 geneontology_Molecular_Function

GO:0097153 cysteine-type endopeptidase activity involved in apoptotic process http://amigo.geneontology.org/amigo/term/GO:0097153 14 3 0.0781816400509454 38.3721804511278 5.75412710832612e-05 0.00452153251196363 834;836;841 CASP3;CASP8;CASP1 geneontology_Molecular_Function

GO:0004197 cysteine-type endopeptidase activity http://amigo.geneontology.org/amigo/term/GO:0004197 81 5 0.452336631723327 11.0537145332467 8.42095564320688e-05 0.00596013957692932 834;836;841;1508;5641 CASP3;CTSB;CASP8;CASP1;LGMN geneontology_Molecular_Function

GO:0004190 aspartic-type endopeptidase activity http://amigo.geneontology.org/amigo/term/GO:0004190 16 3 0.0893504457725091 33.5756578947368 8.78252315421602e-05 0.00596013957692932 836;5972;23621 CASP3;REN;BACE1 geneontology_Molecular_Function

GO:0070001 aspartic-type peptidase activity http://amigo.geneontology.org/amigo/term/GO:0070001 16 3 0.0893504457725091 33.5756578947368 8.78252315421602e-05 0.00596013957692932 836;5972;23621 CASP3;REN;BACE1 geneontology_Molecular_Function

GO:0052689 carboxylic ester hydrolase activity http://amigo.geneontology.org/amigo/term/GO:0052689 57 4 0.318310963064564 12.5663281009541 0.000276998718745425 0.0179808298733443 43;760;2166;11343 ACHE;FAAH;MGLL;CA2 geneontology_Molecular_Function

GO:0070491 repressing transcription factor binding http://amigo.geneontology.org/amigo/term/GO:0070491 62 4 0.346232977368473 11.5529145444256 0.000383168224857511 0.0238362566546777 5465;5467;5468;6774 PPARG;PPARA;STAT3;PPARD geneontology_Molecular_Function

GO:0001609 G protein-coupled adenosine receptor activity http://amigo.geneontology.org/amigo/term/GO:0001609 6 2 0.0335064171646909 59.6900584795322 0.00045305515269789 0.0260158208837673 136;140 ADORA2B;ADORA3 geneontology_Molecular_Function

GO:0004936 alpha-adrenergic receptor activity http://amigo.geneontology.org/amigo/term/GO:0004936 6 2 0.0335064171646909 59.6900584795322 0.00045305515269789 0.0260158208837673 150;152 ADRA2A;ADRA2C geneontology_Molecular_Function

GO:0097718 disordered domain specific binding http://amigo.geneontology.org/amigo/term/GO:0097718 28 3 0.156363280101891 19.1860902255639 0.000489916863415596 0.0270905880399809 3320;4193;9817 KEAP1;HSP90AA1;MDM2 geneontology_Molecular_Function

GO:0019903 protein phosphatase binding http://amigo.geneontology.org/amigo/term/GO:0019903 121 5 0.6757127461546 7.39959402638828 0.000551480267196425 0.028090381189143 596;5468;5747;6098;6774 PPARG;PTK2;BCL2;STAT3;ROS1 geneontology_Molecular_Function

GO:0004032 alditol:NADP+ 1-oxidoreductase activity http://amigo.geneontology.org/amigo/term/GO:0004032 7 2 0.0390908200254727 51.1629072681704 0.000632001695993534 0.028090381189143 231;8644 AKR1B1;AKR1C3 geneontology_Molecular_Function

GO:0008106 alcohol dehydrogenase (NADP+) activity http://amigo.geneontology.org/amigo/term/GO:0008106 7 2 0.0390908200254727 51.1629072681704 0.000632001695993534 0.028090381189143 231;8644 AKR1B1;AKR1C3 geneontology_Molecular_Function

GO:0051379 epinephrine binding http://amigo.geneontology.org/amigo/term/GO:0051379 7 2 0.0390908200254727 51.1629072681704 0.000632001695993534 0.028090381189143 150;152 ADRA2A;ADRA2C geneontology_Molecular_Function

GO:0003708 retinoic acid receptor activity http://amigo.geneontology.org/amigo/term/GO:0003708 7 2 0.0390908200254727 51.1629072681704 0.000632001695993534 0.028090381189143 5914;6256 RXRA;RARA geneontology_Molecular_Function

GO:0050693 LBD domain binding http://amigo.geneontology.org/amigo/term/GO:0050693 7 2 0.0390908200254727 51.1629072681704 0.000632001695993534 0.028090381189143 5468;6256 PPARG;RXRA geneontology_Molecular_Function

GO:0001085 RNA polymerase II transcription factor binding http://amigo.geneontology.org/amigo/term/GO:0001085 125 5 0.698050357597727 7.16280701754386 0.000639700576310021 0.028090381189143 2932;5465;5467;5468;6774 PPARG;PPARA;GSK3B;STAT3;PPARD geneontology_Molecular_Function

GO:0005506 iron ion binding http://amigo.geneontology.org/amigo/term/GO:0005506 72 4 0.402077005976291 9.94834307992203 0.000677999738558643 0.0289215317048016 239;240;1544;6319 ALOX12;CYP1A2;ALOX5;SCD geneontology_Molecular_Function

GO:0017171 serine hydrolase activity http://amigo.geneontology.org/amigo/term/GO:0017171 132 5 0.7371411776232 6.78296119085593 0.000819064050088247 0.0339684062994931 43;1508;1636;5328;5550 ACE;ACHE;PREP;CTSB;PLAU geneontology_Molecular_Function

GO:0031490 chromatin DNA binding http://amigo.geneontology.org/amigo/term/GO:0031490 81 4 0.452336631723327 8.84297162659736 0.00105640149473463 0.0419363471505731 5914;6256;6774;7067 STAT3;RXRA;RARA;THRA geneontology_Molecular_Function

GO:0008234 cysteine-type peptidase activity http://amigo.geneontology.org/amigo/term/GO:0008234 140 5 0.781816400509454 6.3953634085213 0.00106736851421418 0.0419363471505731 834;836;841;1508;5641 CASP3;CTSB;CASP8;CASP1;LGMN geneontology_Molecular_Function

GO:0046982 protein heterodimerization activity http://amigo.geneontology.org/amigo/term/GO:0046982 384 8 2.14441069854022 3.73062865497076 0.00122568885976104 0.0469218837852112 150;152;596;5467;5468;5914;5950;6256 PPARG;ADRA2A;ADRA2C;BCL2;RXRA;PPARD;RBP4;RARA geneontology_Molecular_Function

GO:0004033 aldo-keto reductase (NADP) activity http://amigo.geneontology.org/amigo/term/GO:0004033 11 2 0.0614284314686 32.5582137161085 0.00163163916383124 0.0609009317900012 231;8644 AKR1B1;AKR1C3 geneontology_Molecular_Function

GO:0048156 tau protein binding http://amigo.geneontology.org/amigo/term/GO:0048156 45 3 0.251298128735182 11.9380116959064 0.00198420393596865 0.0722540604000293 2932;3320;5641 GSK3B;HSP90AA1;LGMN geneontology_Molecular_Function

GO:0008656 cysteine-type endopeptidase activator activity involved in apoptotic process http://amigo.geneontology.org/amigo/term/GO:0008656 14 2 0.0781816400509454 25.5814536340852 0.00267073593813549 0.0927304361775882 834;836 CASP3;CASP1 geneontology_Molecular_Function

GO:0005123 death receptor binding http://amigo.geneontology.org/amigo/term/GO:0005123 14 2 0.0781816400509454 25.5814536340852 0.00267073593813549 0.0927304361775882 836;841 CASP3;CASP8 geneontology_Molecular_Function

GO:0004935 adrenergic receptor activity http://amigo.geneontology.org/amigo/term/GO:0004935 15 2 0.0837660429117272 23.8760233918129 0.00307059658963194 0.104190925189102 150;152 ADRA2A;ADRA2C geneontology_Molecular_Function

GO:0008227 G protein-coupled amine receptor activity http://amigo.geneontology.org/amigo/term/GO:0008227 53 3 0.295973351621436 10.1360476663357 0.00317400368310672 0.105306388863963 150;152;3357 ADRA2A;ADRA2C;HTR2B geneontology_Molecular_Function

GO:0016616 oxidoreductase activity, acting on the CH-OH group of donors, NAD or NADP as acceptor http://amigo.geneontology.org/amigo/term/GO:0016616 54 3 0.301557754482218 9.94834307992203 0.00334750135354089 0.106542514856765 231;3939;8644 AKR1B1;AKR1C3;LDHA geneontology_Molecular_Function

GO:0016701 oxidoreductase activity, acting on single donors with incorporation of molecular oxygen http://amigo.geneontology.org/amigo/term/GO:0016701 16 2 0.0893504457725091 22.3837719298246 0.00349670678364467 0.106542514856765 239;240 ALOX12;ALOX5 geneontology_Molecular_Function

GO:0016702 oxidoreductase activity, acting on single donors with incorporation of molecular oxygen, incorporation of two atoms of oxygen http://amigo.geneontology.org/amigo/term/GO:0016702 16 2 0.0893504457725091 22.3837719298246 0.00349670678364467 0.106542514856765 239;240 ALOX12;ALOX5 geneontology_Molecular_Function

GO:0016505 peptidase activator activity involved in apoptotic process http://amigo.geneontology.org/amigo/term/GO:0016505 16 2 0.0893504457725091 22.3837719298246 0.00349670678364467 0.106542514856765 834;836 CASP3;CASP1 geneontology_Molecular_Function

GO:0097110 scaffold protein binding http://amigo.geneontology.org/amigo/term/GO:0097110 57 3 0.318310963064564 9.42474607571561 0.00390251636976147 0.1133752999069 841;3320;4193 HSP90AA1;CASP8;MDM2 geneontology_Molecular_Function

GO:0016614 oxidoreductase activity, acting on CH-OH group of donors http://amigo.geneontology.org/amigo/term/GO:0016614 57 3 0.318310963064564 9.42474607571561 0.00390251636976147 0.1133752999069 231;3939;8644 AKR1B1;AKR1C3;LDHA geneontology_Molecular_Function

GO:0031690 adrenergic receptor binding http://amigo.geneontology.org/amigo/term/GO:0031690 17 2 0.0949348486332909 21.0670794633643 0.00394877132964422 0.1133752999069 150;152 ADRA2A;ADRA2C geneontology_Molecular_Function

GO:1901338 catecholamine binding http://amigo.geneontology.org/amigo/term/GO:1901338 18 2 0.100519251494073 19.8966861598441 0.00442649741497914 0.124693596991771 150;152 ADRA2A;ADRA2C geneontology_Molecular_Function

GO:0003713 transcription coactivator activity http://amigo.geneontology.org/amigo/term/GO:0003713 282 6 1.57480160674047 3.8100037327361 0.00469318312424349 0.129757822305473 5465;5467;5468;5914;6256;7067 PPARG;PPARA;RXRA;PPARD;RARA;THRA geneontology_Molecular_Function

GO:0008238 exopeptidase activity http://amigo.geneontology.org/amigo/term/GO:0008238 62 3 0.346232977368473 8.66468590831919 0.00494596825334293 0.134260556404382 1636;5550;23621 ACE;BACE1;PREP geneontology_Molecular_Function

GO:0035586 purinergic receptor activity http://amigo.geneontology.org/amigo/term/GO:0035586 20 2 0.111688057215636 17.9070175438596 0.00545777474294029 0.145508173057319 136;140 ADORA2B;ADORA3 geneontology_Molecular_Function

GO:0008289 lipid binding http://amigo.geneontology.org/amigo/term/GO:0008289 491 8 2.74194180464387 2.91764033301176 0.00560525267831846 0.146818285065429 2167;2171;5465;5467;5468;5914;5950;6256 PPARG;PPARA;FABP5;RXRA;FABP4;PPARD;RBP4;RARA geneontology_Molecular_Function

GO:0008236 serine-type peptidase activity http://amigo.geneontology.org/amigo/term/GO:0008236 130 4 0.725972371901636 5.50985155195682 0.00589373051806807 0.151235333512077 1508;1636;5328;5550 ACE;PREP;CTSB;PLAU geneontology_Molecular_Function

GO:0016491 oxidoreductase activity http://amigo.geneontology.org/amigo/term/GO:0016491 393 7 2.19467032428725 3.18954510959332 0.00597648002492468 0.151235333512077 231;239;240;1544;3939;6319;8644 AKR1B1;ALOX12;AKR1C3;CYP1A2;ALOX5;LDHA;SCD geneontology_Molecular_Function

GO:0016705 oxidoreductase activity, acting on paired donors, with incorporation or reduction of molecular oxygen http://amigo.geneontology.org/amigo/term/GO:0016705 69 3 0.385323797393945 7.78565980167811 0.00666606480086962 0.165873912461639 1544;6319;8644 AKR1C3;CYP1A2;SCD geneontology_Molecular_Function

GO:1901618 organic hydroxy compound transmembrane transporter activity http://amigo.geneontology.org/amigo/term/GO:1901618 23 2 0.128441265797982 15.5713196033562 0.00718996685754303 0.175977385546094 5950;6580 SLC22A1;RBP4 geneontology_Molecular_Function

GO:0051393 alpha-actinin binding http://amigo.geneontology.org/amigo/term/GO:0051393 25 2 0.139610071519545 14.3256140350877 0.00846499858468275 0.20384262720857 5468;5914 PPARG;RARA geneontology_Molecular_Function

GO:0003682 chromatin binding http://amigo.geneontology.org/amigo/term/GO:0003682 421 7 2.35103360438914 2.97741384339709 0.00860813209601774 0.203999066973881 5468;5914;6256;6774;7067;8019;55193 PPARG;STAT3;RXRA;BRD3;PBRM1;RARA;THRA geneontology_Molecular_Function

GO:0051059 NF-kappaB binding http://amigo.geneontology.org/amigo/term/GO:0051059 26 2 0.145194474380327 13.774628879892 0.00913775564514807 0.21316670590947 2932;5467 GSK3B;PPARD geneontology_Molecular_Function

GO:0051019 mitogen-activated protein kinase binding http://amigo.geneontology.org/amigo/term/GO:0051019 27 2 0.150778877241109 13.264457439896 0.00983364323124403 0.225871220680728 1636;9261 ACE;MAPKAPK2 geneontology_Molecular_Function

GO:0016903 oxidoreductase activity, acting on the aldehyde or oxo group of donors http://amigo.geneontology.org/amigo/term/GO:0016903 28 2 0.156363280101891 12.7907268170426 0.0105523914133847 0.238707884548233 231;8644 AKR1B1;AKR1C3 geneontology_Molecular_Function

GO:0042805 actinin binding http://amigo.geneontology.org/amigo/term/GO:0042805 31 2 0.173116488684236 11.5529145444256 0.0128431331626955 0.286191012117975 5468;5914 PPARG;RARA geneontology_Molecular_Function

GO:0004713 protein tyrosine kinase activity http://amigo.geneontology.org/amigo/term/GO:0004713 165 4 0.921426472029 4.34109516214779 0.013383139913355 0.293838645450575 2260;3320;5747;6098 PTK2;HSP90AA1;FGFR1;ROS1 geneontology_Molecular_Function

GO:0042169 SH2 domain binding http://amigo.geneontology.org/amigo/term/GO:0042169 32 2 0.178700891545018 11.1918859649123 0.013650668221998 0.295048938936741 2260;5747 PTK2;FGFR1 geneontology_Molecular_Function

GO:0042277 peptide binding http://amigo.geneontology.org/amigo/term/GO:0042277 256 5 1.42960713236014 3.49746436403509 0.01383350684901 0.295048938936741 43;5468;6256;9536;23621 PPARG;BACE1;ACHE;PTGES;RXRA geneontology_Molecular_Function

GO:0016504 peptidase activator activity http://amigo.geneontology.org/amigo/term/GO:0016504 33 2 0.1842852944058 10.8527379053695 0.0144797469367623 0.30072641348827 834;836 CASP3;CASP1 geneontology_Molecular_Function

GO:0051427 hormone receptor binding http://amigo.geneontology.org/amigo/term/GO:0051427 169 4 0.943764083472127 4.23834734765909 0.014502546397291 0.30072641348827 2167;5468;6256;6774 PPARG;STAT3;RXRA;FABP4 geneontology_Molecular_Function

GO:0031625 ubiquitin protein ligase binding http://amigo.geneontology.org/amigo/term/GO:0031625 260 5 1.45194474380327 3.44365721997301 0.0147087935732934 0.300825052122287 596;841;2932;3320;4193 GSK3B;BCL2;HSP90AA1;CASP8;MDM2 geneontology_Molecular_Function

GO:0004497 monooxygenase activity http://amigo.geneontology.org/amigo/term/GO:0004497 34 2 0.189869697266582 10.5335397316821 0.0153301123534653 0.309295374915184 1544;8644 AKR1C3;CYP1A2 geneontology_Molecular_Function

GO:0042826 histone deacetylase binding http://amigo.geneontology.org/amigo/term/GO:0042826 99 3 0.5528558832174 5.42636895268474 0.0177457215968821 0.353258164588601 3320;5914;83933 HSP90AA1;RARA;HDAC10 geneontology_Molecular_Function

GO:0044389 ubiquitin-like protein ligase binding http://amigo.geneontology.org/amigo/term/GO:0044389 275 5 1.535710786715 3.25582137161085 0.0183228600989835 0.359947764839242 596;841;2932;3320;4193 GSK3B;BCL2;HSP90AA1;CASP8;MDM2 geneontology_Molecular_Function

GO:0043028 cysteine-type endopeptidase regulator activity involved in apoptotic process http://amigo.geneontology.org/amigo/term/GO:0043028 38 2 0.212207308709709 9.42474607571561 0.0189393754968135 0.367227111905747 834;836 CASP3;CASP1 geneontology_Molecular_Function

GO:0051018 protein kinase A binding http://amigo.geneontology.org/amigo/term/GO:0051018 40 2 0.223376114431273 8.95350877192982 0.0208652001522838 0.399381331119996 2932;5914 GSK3B;RARA geneontology_Molecular_Function

GO:0033218 amide binding http://amigo.geneontology.org/amigo/term/GO:0033218 289 5 1.61389242676594 3.09809992108298 0.0221891994118496 0.419347781289765 43;5468;6256;9536;23621 PPARG;BACE1;ACHE;PTGES;RXRA geneontology_Molecular_Function

GO:0032813 tumor necrosis factor receptor superfamily binding http://amigo.geneontology.org/amigo/term/GO:0032813 42 2 0.234544920152836 8.52715121136174 0.0228692119045004 0.426796667167739 836;841 CASP3;CASP8 geneontology_Molecular_Function

GO:0042165 neurotransmitter binding http://amigo.geneontology.org/amigo/term/GO:0042165 43 2 0.240129323013618 8.32884536923705 0.0238999379643847 0.440526017047239 43;3357 ACHE;HTR2B geneontology_Molecular_Function

GO:0002020 protease binding http://amigo.geneontology.org/amigo/term/GO:0002020 115 3 0.642206328989909 4.67139588100687 0.0262478121538413 0.448179887853968 596;836;2932 CASP3;GSK3B;BCL2 geneontology_Molecular_Function

GO:0004252 serine-type endopeptidase activity http://amigo.geneontology.org/amigo/term/GO:0004252 117 3 0.653375134711472 4.59154295996401 0.0274397106291542 0.448179887853968 1508;5328;5550 PREP;CTSB;PLAU geneontology_Molecular_Function

GO:0051380 norepinephrine binding http://amigo.geneontology.org/amigo/term/GO:0051380 5 1 0.0279220143039091 35.8140350877193 0.0276172469407669 0.448179887853968 150 ADRA2A geneontology_Molecular_Function

GO:0016803 ether hydrolase activity http://amigo.geneontology.org/amigo/term/GO:0016803 5 1 0.0279220143039091 35.8140350877193 0.0276172469407669 0.448179887853968 239 ALOX12 geneontology_Molecular_Function

GO:0050542 icosanoid binding http://amigo.geneontology.org/amigo/term/GO:0050542 5 1 0.0279220143039091 35.8140350877193 0.0276172469407669 0.448179887853968 5468 PPARG geneontology_Molecular_Function

GO:0050544 arachidonic acid binding http://amigo.geneontology.org/amigo/term/GO:0050544 5 1 0.0279220143039091 35.8140350877193 0.0276172469407669 0.448179887853968 5468 PPARG geneontology_Molecular_Function

GO:0015616 DNA translocase activity http://amigo.geneontology.org/amigo/term/GO:0015616 5 1 0.0279220143039091 35.8140350877193 0.0276172469407669 0.448179887853968 55193 PBRM1 geneontology_Molecular_Function

GO:0015651 quaternary ammonium group transmembrane transporter activity http://amigo.geneontology.org/amigo/term/GO:0015651 5 1 0.0279220143039091 35.8140350877193 0.0276172469407669 0.448179887853968 6580 SLC22A1 geneontology_Molecular_Function

GO:0051525 NFAT protein binding http://amigo.geneontology.org/amigo/term/GO:0051525 5 1 0.0279220143039091 35.8140350877193 0.0276172469407669 0.448179887853968 5465 PPARA geneontology_Molecular_Function

GO:0005007 fibroblast growth factor-activated receptor activity http://amigo.geneontology.org/amigo/term/GO:0005007 5 1 0.0279220143039091 35.8140350877193 0.0276172469407669 0.448179887853968 2260 FGFR1 geneontology_Molecular_Function

GO:0004457 lactate dehydrogenase activity http://amigo.geneontology.org/amigo/term/GO:0004457 5 1 0.0279220143039091 35.8140350877193 0.0276172469407669 0.448179887853968 3939 LDHA geneontology_Molecular_Function

GO:0043621 protein self-association http://amigo.geneontology.org/amigo/term/GO:0043621 47 2 0.262466934456745 7.62000746547219 0.0282088142166175 0.452857630380751 43;5468 PPARG;ACHE geneontology_Molecular_Function

GO:0051213 dioxygenase activity http://amigo.geneontology.org/amigo/term/GO:0051213 50 2 0.279220143039091 7.16280701754386 0.0316292831667302 0.480560210290857 239;240 ALOX12;ALOX5 geneontology_Molecular_Function

GO:0004704 NF-kappaB-inducing kinase activity http://amigo.geneontology.org/amigo/term/GO:0004704 6 1 0.0335064171646909 29.8450292397661 0.0330500852983807 0.480560210290857 9020 MAP3K14 geneontology_Molecular_Function

GO:0050543 icosatetraenoic acid binding http://amigo.geneontology.org/amigo/term/GO:0050543 6 1 0.0335064171646909 29.8450292397661 0.0330500852983807 0.480560210290857 5468 PPARG geneontology_Molecular_Function

GO:0008242 omega peptidase activity http://amigo.geneontology.org/amigo/term/GO:0008242 6 1 0.0335064171646909 29.8450292397661 0.0330500852983807 0.480560210290857 23621 BACE1 geneontology_Molecular_Function

GO:0099626 voltage-gated calcium channel activity involved in regulation of presynaptic cytosolic calcium levels http://amigo.geneontology.org/amigo/term/GO:0099626 6 1 0.0335064171646909 29.8450292397661 0.0330500852983807 0.480560210290857 1268 CNR1 geneontology_Molecular_Function

GO:0070330 aromatase activity http://amigo.geneontology.org/amigo/term/GO:0070330 6 1 0.0335064171646909 29.8450292397661 0.0330500852983807 0.480560210290857 1544 CYP1A2 geneontology_Molecular_Function

GO:0004955 prostaglandin receptor activity http://amigo.geneontology.org/amigo/term/GO:0004955 6 1 0.0335064171646909 29.8450292397661 0.0330500852983807 0.480560210290857 5468 PPARG geneontology_Molecular_Function

GO:0019788 NEDD8 transferase activity http://amigo.geneontology.org/amigo/term/GO:0019788 6 1 0.0335064171646909 29.8450292397661 0.0330500852983807 0.480560210290857 4193 MDM2 geneontology_Molecular_Function

GO:0008504 monoamine transmembrane transporter activity http://amigo.geneontology.org/amigo/term/GO:0008504 6 1 0.0335064171646909 29.8450292397661 0.0330500852983807 0.480560210290857 6580 SLC22A1 geneontology_Molecular_Function

GO:0001664 G protein-coupled receptor binding http://amigo.geneontology.org/amigo/term/GO:0001664 218 4 1.21739982365044 3.28569129245131 0.0331531826255581 0.480560210290857 150;152;1636;6774 ACE;ADRA2A;ADRA2C;STAT3 geneontology_Molecular_Function

GO:0005518 collagen binding http://amigo.geneontology.org/amigo/term/GO:0005518 55 2 0.307142157343 6.51164274322169 0.0376717475404711 0.499221540465595 43;1508 ACHE;CTSB geneontology_Molecular_Function

GO:0016788 hydrolase activity, acting on ester bonds http://amigo.geneontology.org/amigo/term/GO:0016788 449 6 2.50739688449104 2.39291993904583 0.03832224773699 0.499221540465595 43;249;760;2166;5141;11343 ACHE;FAAH;PDE4A;MGLL;ALPL;CA2 geneontology_Molecular_Function

GO:0099511 voltage-gated calcium channel activity involved in regulation of cytosolic calcium levels http://amigo.geneontology.org/amigo/term/GO:0099511 7 1 0.0390908200254727 25.5814536340852 0.0384530992321122 0.499221540465595 1268 CNR1 geneontology_Molecular_Function

GO:0004089 carbonate dehydratase activity http://amigo.geneontology.org/amigo/term/GO:0004089 7 1 0.0390908200254727 25.5814536340852 0.0384530992321122 0.499221540465595 760 CA2 geneontology_Molecular_Function

GO:0016229 steroid dehydrogenase activity http://amigo.geneontology.org/amigo/term/GO:0016229 7 1 0.0390908200254727 25.5814536340852 0.0384530992321122 0.499221540465595 8644 AKR1C3 geneontology_Molecular_Function

GO:0033764 steroid dehydrogenase activity, acting on the CH-OH group of donors, NAD or NADP as acceptor http://amigo.geneontology.org/amigo/term/GO:0033764 7 1 0.0390908200254727 25.5814536340852 0.0384530992321122 0.499221540465595 8644 AKR1C3 geneontology_Molecular_Function

GO:0070008 serine-type exopeptidase activity http://amigo.geneontology.org/amigo/term/GO:0070008 7 1 0.0390908200254727 25.5814536340852 0.0384530992321122 0.499221540465595 5550 PREP geneontology_Molecular_Function

GO:0016805 dipeptidase activity http://amigo.geneontology.org/amigo/term/GO:0016805 7 1 0.0390908200254727 25.5814536340852 0.0384530992321122 0.499221540465595 1636 ACE geneontology_Molecular_Function

GO:0004887 thyroid hormone receptor activity http://amigo.geneontology.org/amigo/term/GO:0004887 7 1 0.0390908200254727 25.5814536340852 0.0384530992321122 0.499221540465595 7067 THRA geneontology_Molecular_Function

GO:0004954 prostanoid receptor activity http://amigo.geneontology.org/amigo/term/GO:0004954 7 1 0.0390908200254727 25.5814536340852 0.0384530992321122 0.499221540465595 5468 PPARG geneontology_Molecular_Function

GO:0031730 CCR5 chemokine receptor binding http://amigo.geneontology.org/amigo/term/GO:0031730 7 1 0.0390908200254727 25.5814536340852 0.0384530992321122 0.499221540465595 6774 STAT3 geneontology_Molecular_Function

GO:0097371 MDM2/MDM4 family protein binding http://amigo.geneontology.org/amigo/term/GO:0097371 7 1 0.0390908200254727 25.5814536340852 0.0384530992321122 0.499221540465595 5465 PPARA geneontology_Molecular_Function
