# Supplementary figures and images for: A Network Pharmacology Study: Reveal the Mechanisms of Palovarotene Against Heterotopic Ossification
Source: Front Med (Lausanne). 2022 May 13;9:897392. doi: 10.3389/fmed.2022.897392 (PMC9136101; doi:10.3389/fmed.2022.897392)

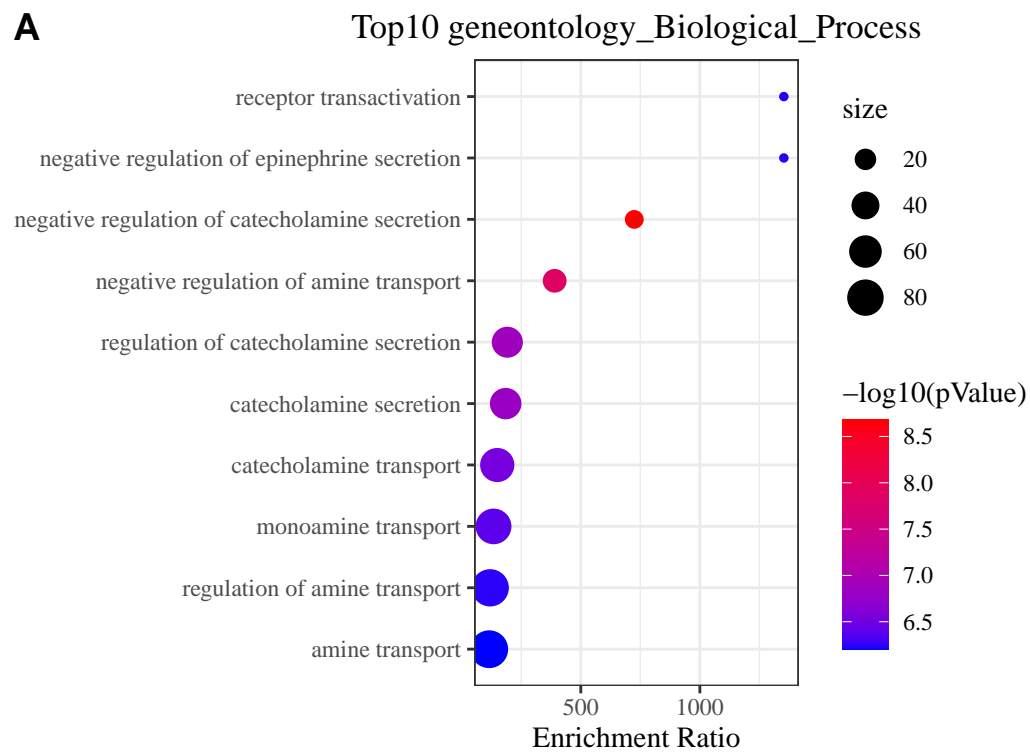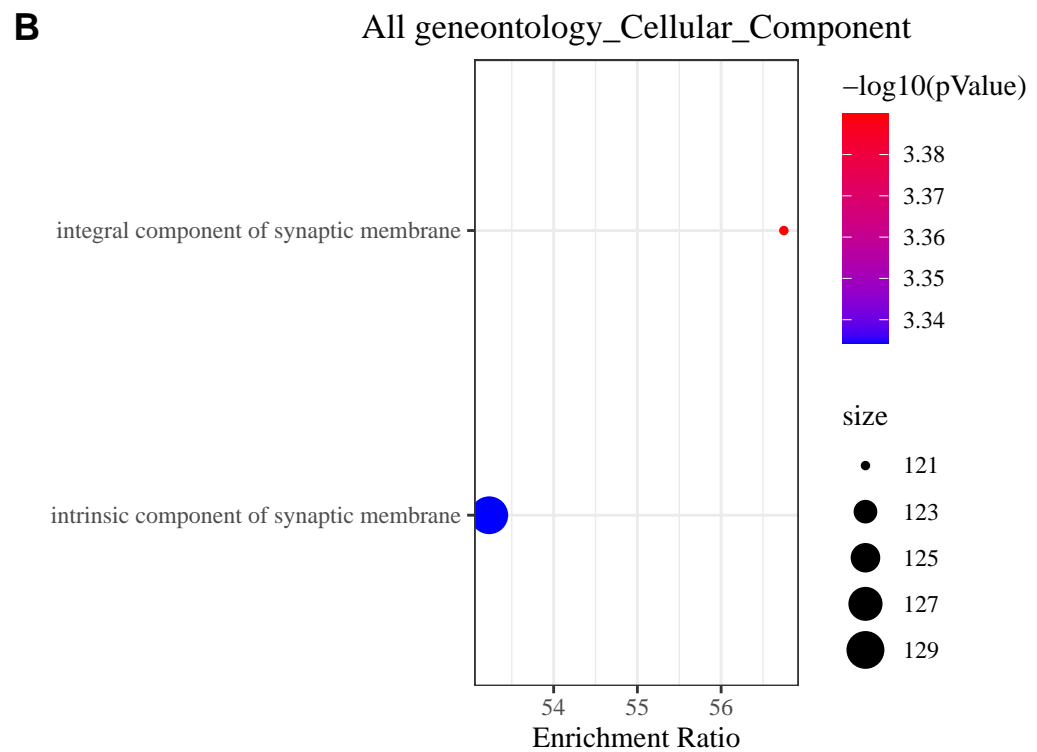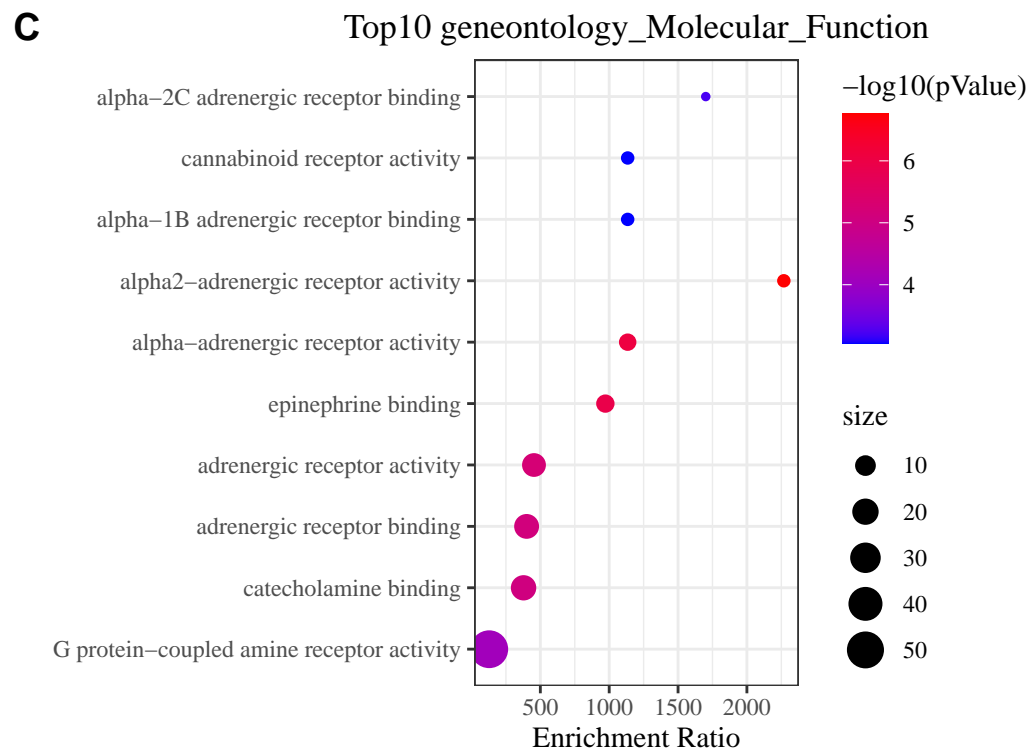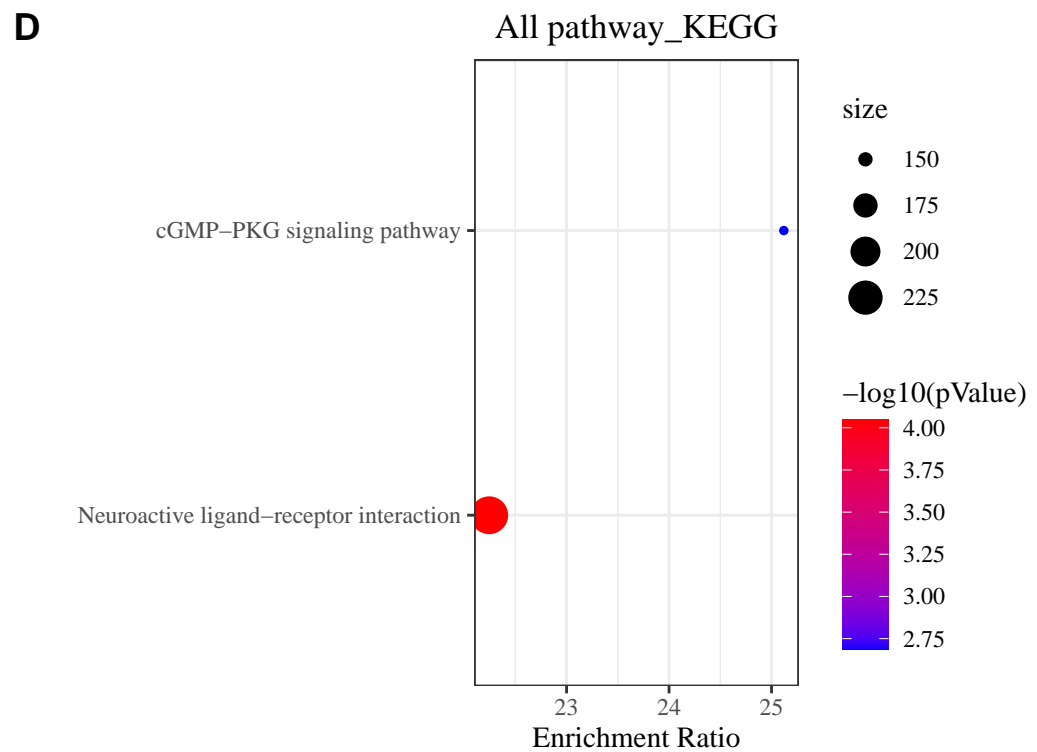

Supplement: Supplementary file 3 [file Supplementary_Material_3.zip › data sheet 12.pdf]

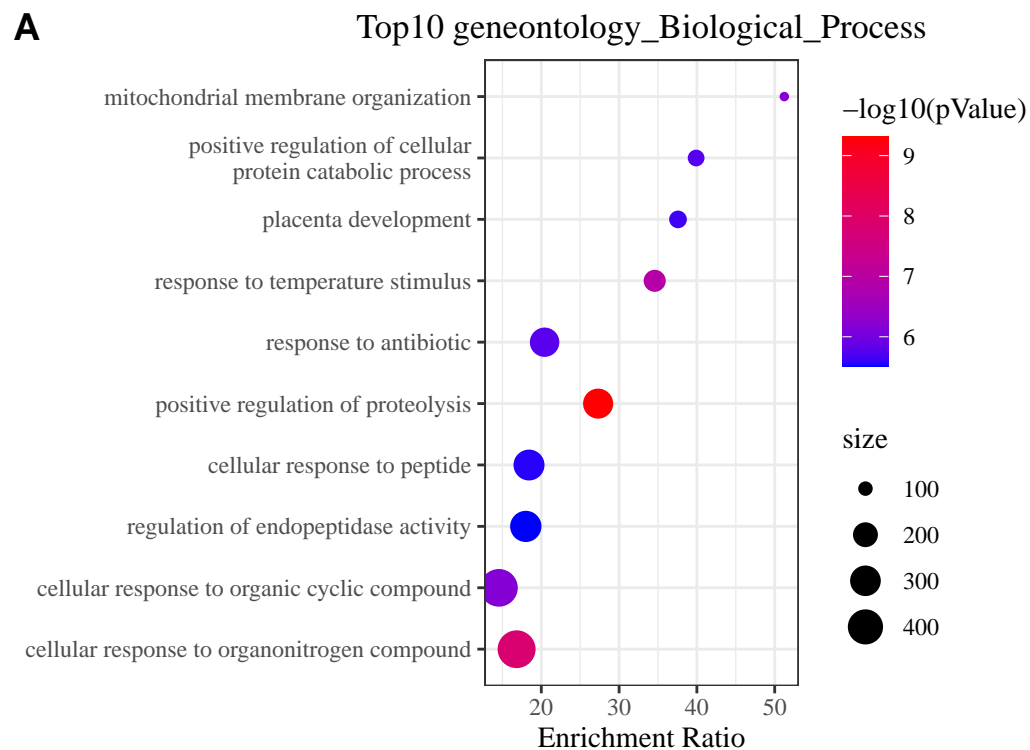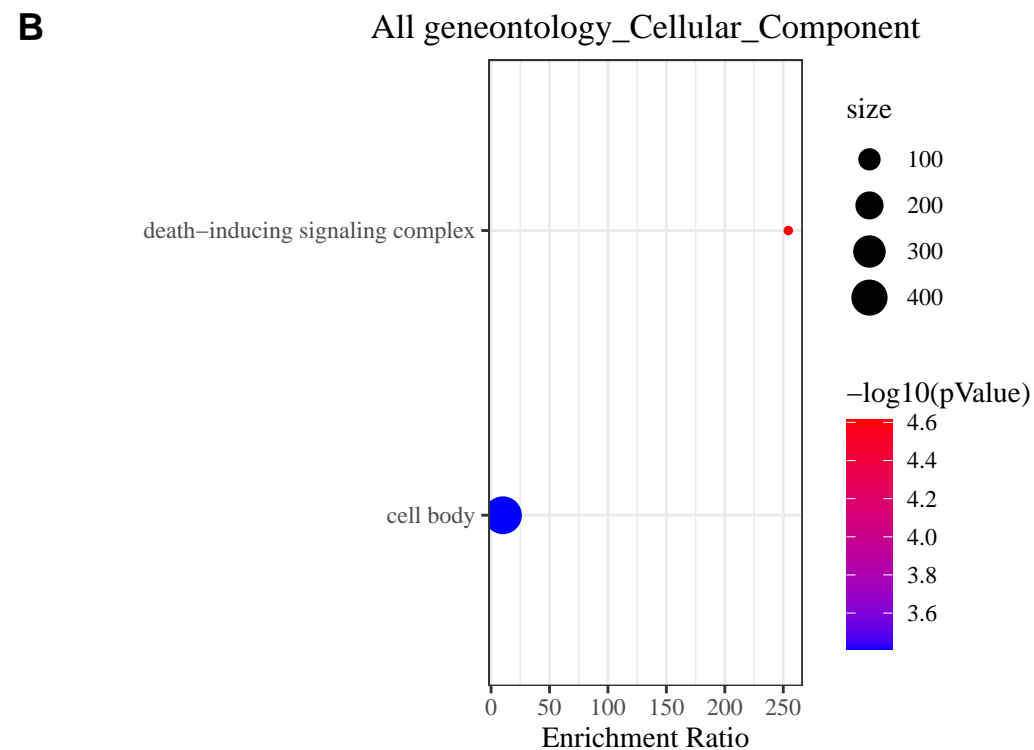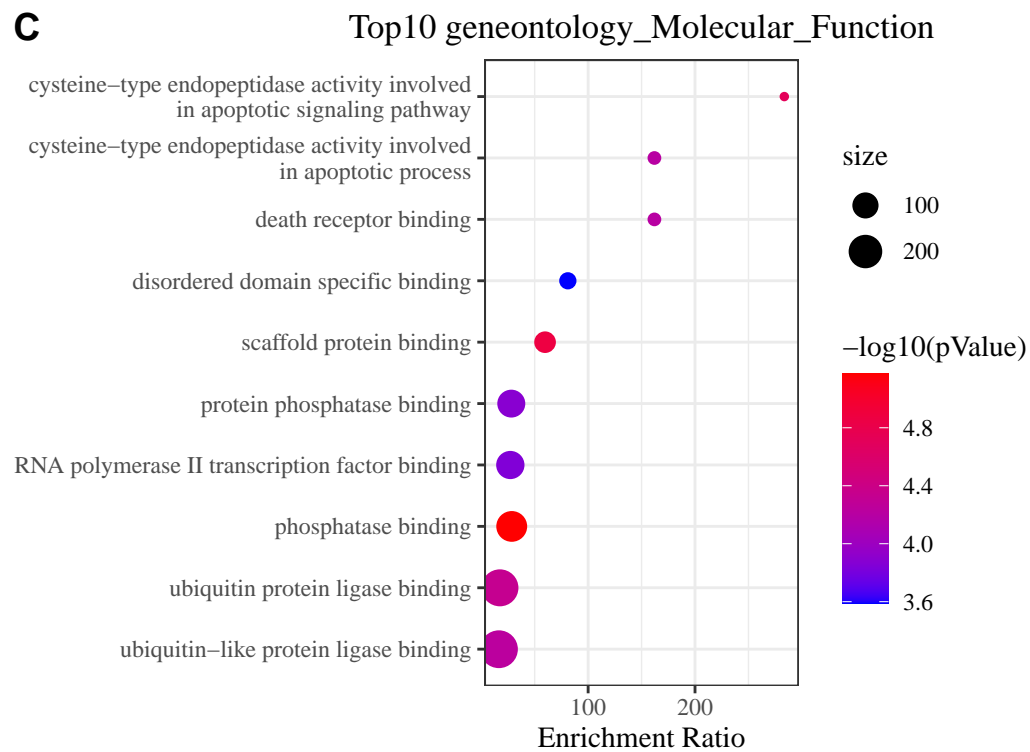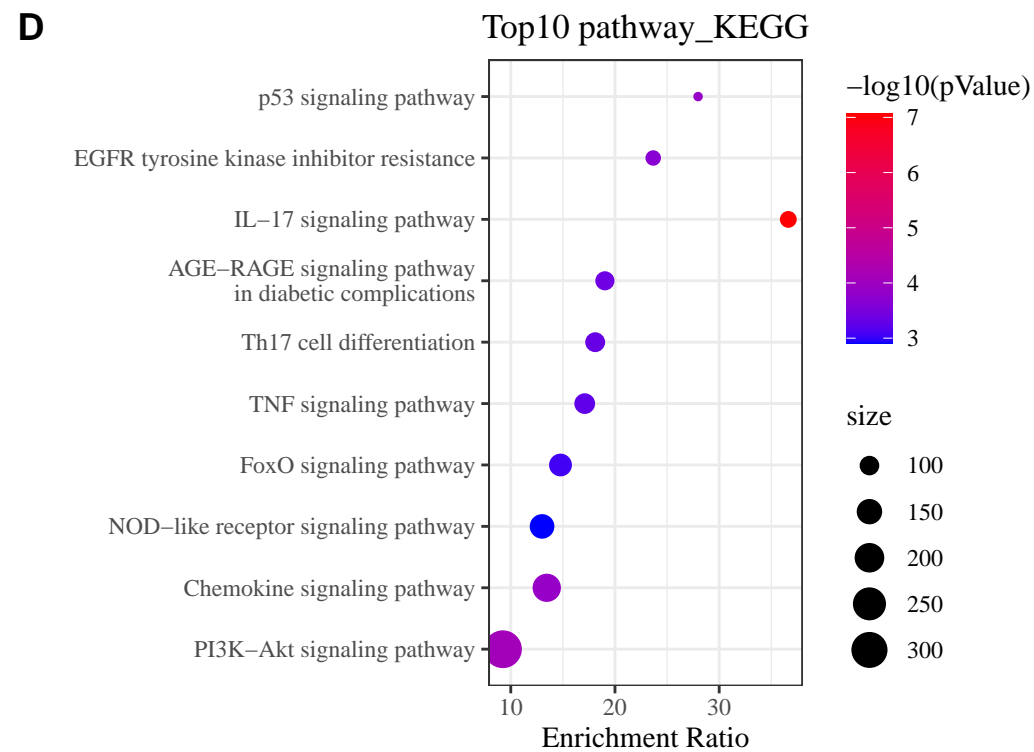

Supplement: Supplementary file 3 [file Supplementary_Material_3.zip › data sheet 9.pdf]

A

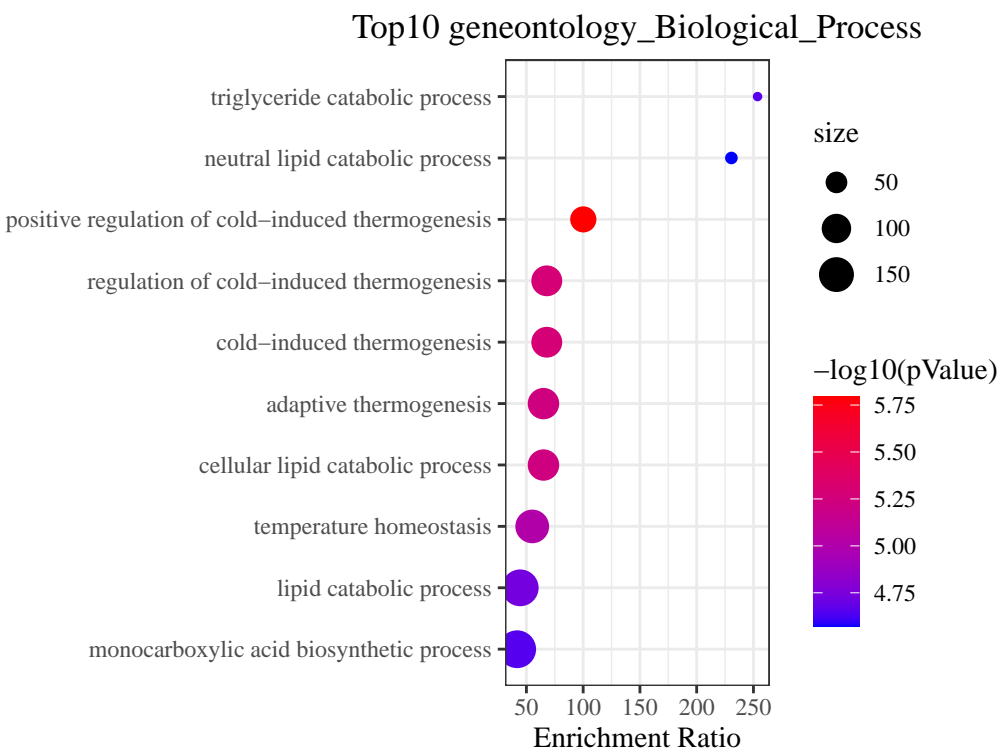

B

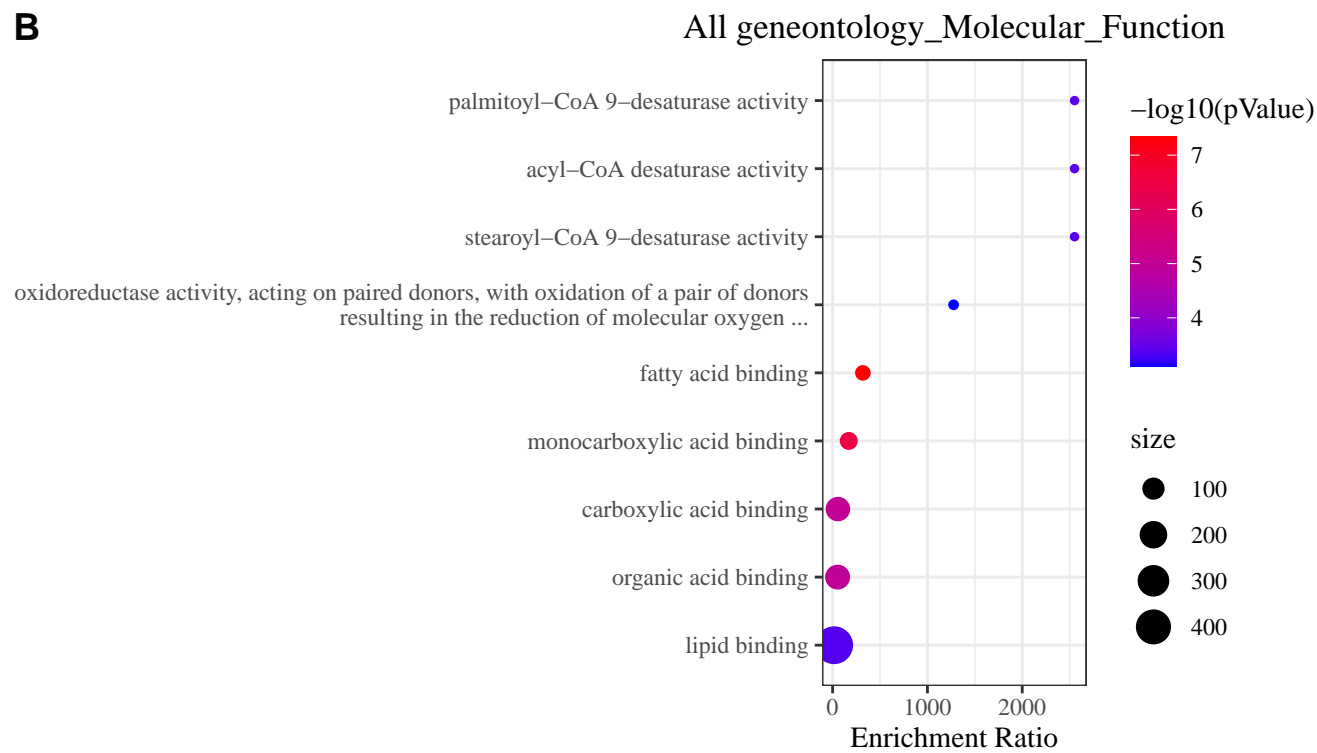

C

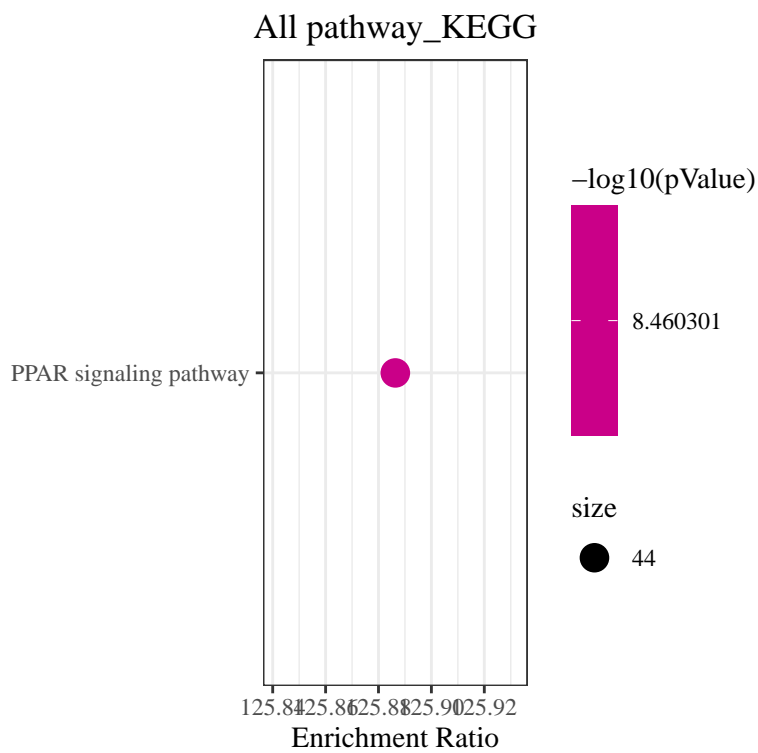

Supplement: Supplementary file 3 [file Supplementary_Material_3.zip › data sheet 10.pdf]

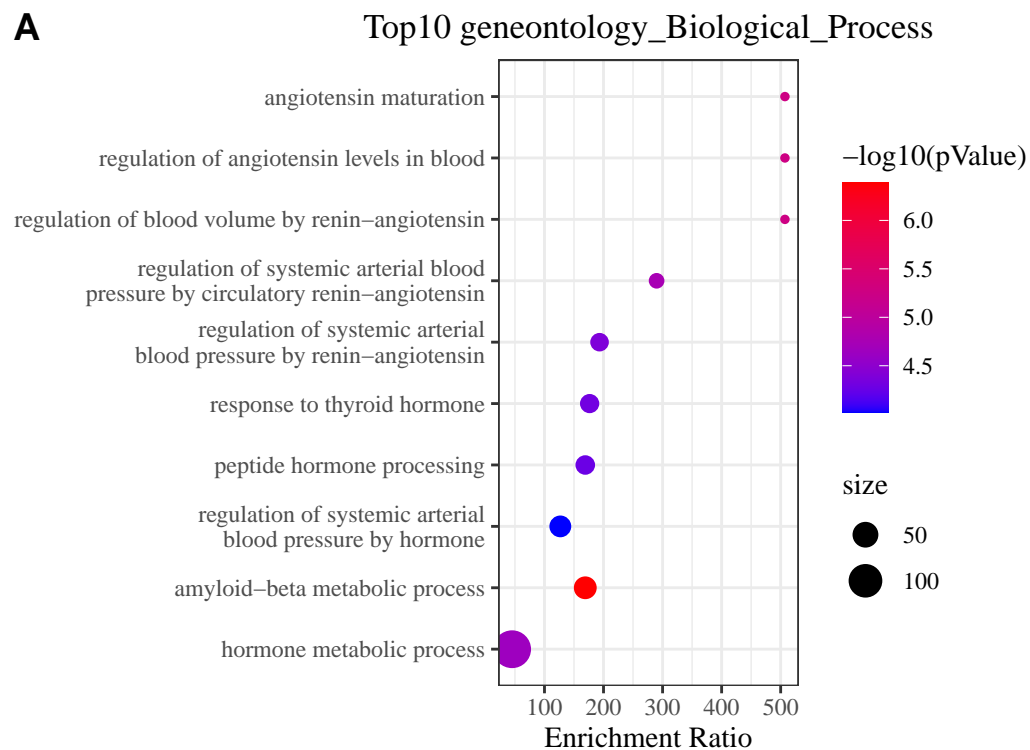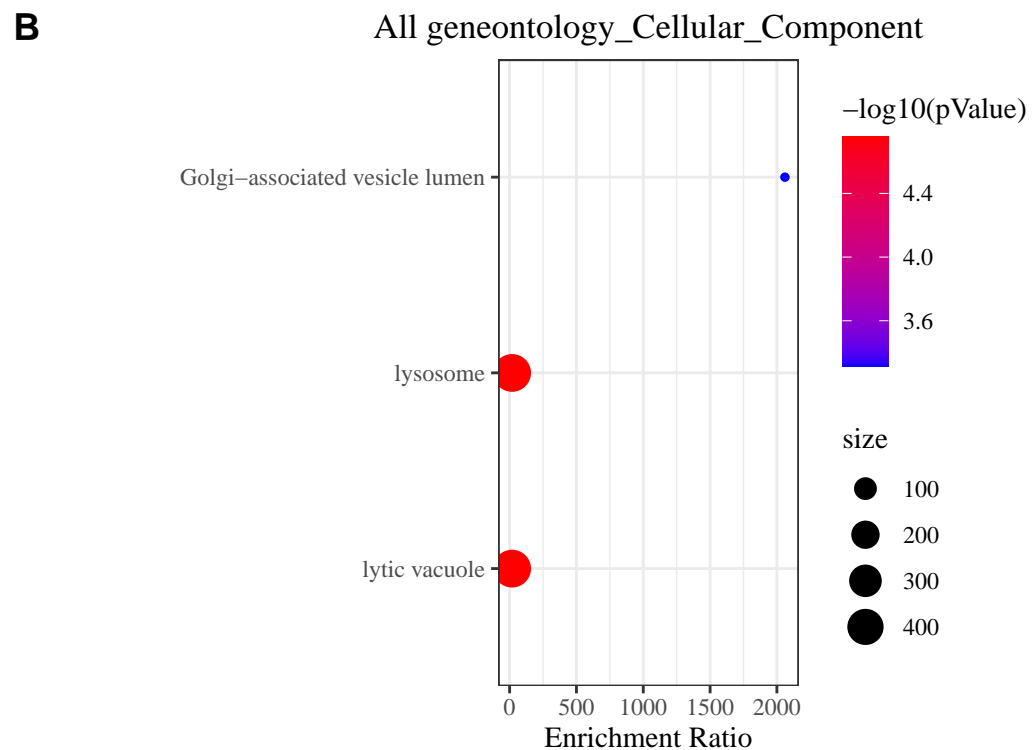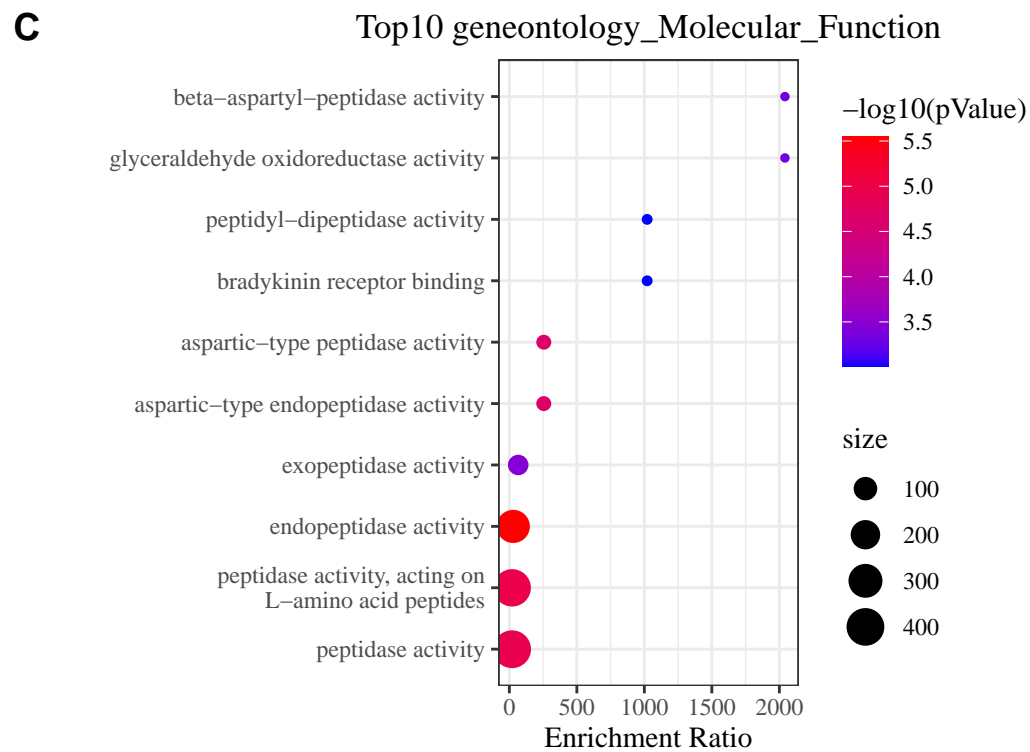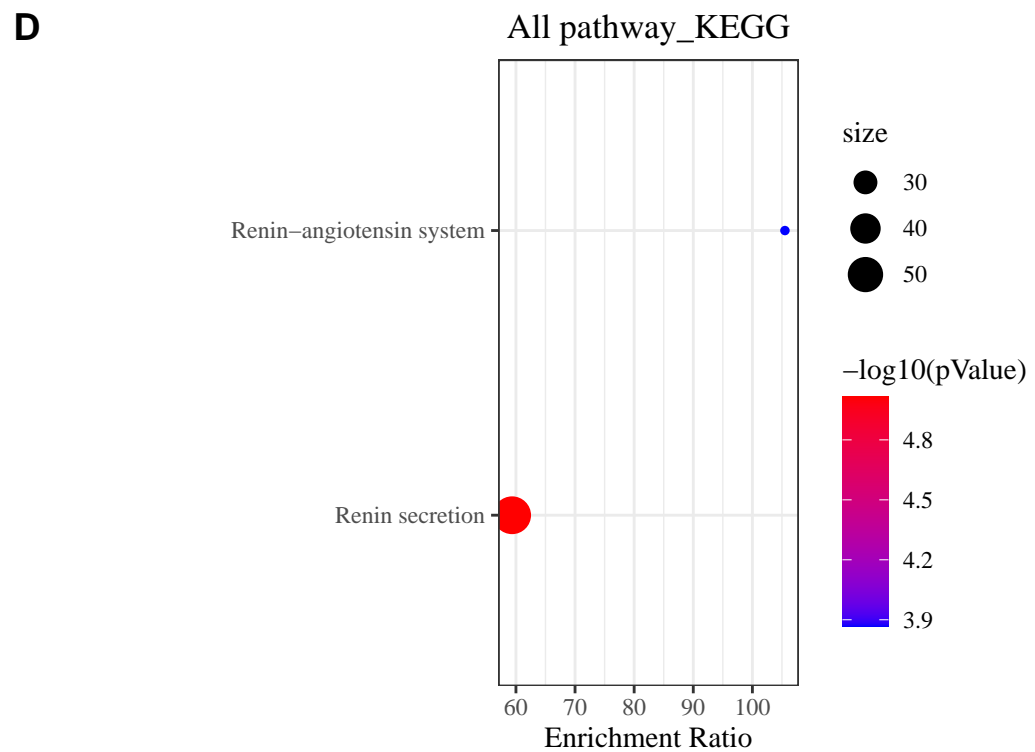

Supplement: Supplementary file 3 [file Supplementary_Material_3.zip › data sheet 11.pdf]
